# Integrated *in silico* and *in vivo* larvicidal evaluation of compounds targeting juvenile hormone for malaria vector control

**DOI:** 10.1371/journal.pone.0352147

**Published:** 2026-06-26

**Authors:** Eric Kibagendi Osoro, Adeniyi Ayinde Abdulwahab, Florence Ezinwa Nkemehule, Adedoyin John-Joy Owolade, Micheal Abimbola Oladosu, Wilberforce K. Ndarawit, Daramola Oluwasegun Oluwatobiloba, Moses Adondua Abah, Fidelis Ngugi, Njogu M. Kimani, Damilola Samuel Bodun

**Affiliations:** 1 Chemoinformatics Academy, Nigeria; 2 Department of Basic Sciences, Tharaka University, Kenya; 3 Faculty of Pharmaceutical Sciences, Bayero University, Kano, Nigeria; 4 Department of Pharmacognosy, Faculty of Pharmacy, University of Lagos, Nigeria; 5 Department of Pharmaceutical Chemistry, Faculty of Pharmacy, Obafemi Awolowo University, Ile Ife, Osun State, Nigeria; 6 Department of Biochemistry, Faculty of Basic Medical Sciences, College of medicine, University of Lagos Idi-Araba, Lagos, Nigeria; 7 Department of Physical Sciences, University of Embu, Kenya; 8 Natural Product Chemistry and Computational Drug Discovery Laboratory, Embu, Kenya; 9 University of Lagos, Nigeria; 10 Department of Biochemistry, Faculty of Biosciences, Federal University Wukari, Wukari, Taraba State, Nigeria; Universidade Federal do Para, BRAZIL

## Abstract

Malaria remains a major global health challenge, particularly in sub-Saharan Africa, where it is a leading cause of morbidity and mortality. Inhibition of the mosquito juvenile hormone binding protein (MJHBP) is essential for developing selective and effective insecticides against malaria vectors. We performed virtual screening on 2,874 compounds from Agrochemical Insecticides Library using Python-based workflow in RDKit and Pandas libraries. The resultant compounds were subjected to molecular docking study against MJHBP (pdb:5V13). Further evaluation was performed through molecular mechanics generalized Born surface area (MM-GBSA) analysis, insecticide-likeness assessment, toxicity and environmental hazard predictions and Density Functional Theory (DFT) studies. The top-ranked compounds, together with the co-crystallized ligand, were subsequently subjected to molecular dynamics (MD) simulations. The molecular docking and MM-GBSA analyses identified fifteen lead compounds as potent inhibitors, exhibiting binding affinities ranging from –49.84 kcal/mol to –89.51 kcal/mol, values comparable to that of the co-crystallized ligand (–75.83 kcal/mol). The DFT results of the analysed compounds indicated that the top candidates, based on docking scores and binding energies, possessed lower HOMO-LUMO energy gap values, 1.52 eV for F3023-0929 and 2.36 eV for F2708-0061, compared to the co-crystallized ligand, which had a gap of 3.43 eV. All the top compounds displayed significant insecticidal potential with minimal environmental hazards. The stability of the most promising compounds (F2708-0061 and F3023-0929), identified based on binding free energy, was further corroborated through MD simulation. Further in vivo experiments revealed that F3023-0929 and F2708-0061 exhibited potent larvicidal activity, with LC₅₀ values of 37.75 ppm and 33.49 ppm, respectively, thereby supporting their potential as promising insecticidal candidates for mosquito control.

## 1. Introduction

Malaria continues to pose a significant public health challenge globally and especially in sub-Saharan Africa, where it remains a leading cause of morbidity and mortality [1]. The primary vectors of this disease are female *Anopheles* mosquitoes, which transmit the Plasmodium pathogen responsible for the illness [2]. In this regard, malaria transmission rates are better reduced by mosquito control which involves vector elimination and minimizing human-vector contact. Traditional control strategies, such as insecticide-treated nets (ITNs) and indoor residual spraying (IRS), have been instrumental in reducing transmission rates [3]. However, the effectiveness of these interventions is increasingly undermined by the development of insecticide resistance among mosquito populations [4]. Studies have documented significant resistance to pyrethroids, organophosphates, carbamates and organochlorines, the primary insecticides used in ITNs and IRS, leading to decreased mortality rates in mosquito populations and compromised control efforts [5,6]. This necessitates the discovery of new effective chemical compounds for vector control.

Pyrethroids and organochlorines cause a knockdown effect on mosquitoes by disrupting the insect’s voltage-gated sodium channels in neuronal membranes while carbamates and organophosphate work by inhibiting acetylcholinesterase (AChE) enzyme, preventing breakdown of the neurotransmitter acetylcholine, which leads to the death of the insect [5]. The growing resistance to these insecticides points to likely alterations of these molecular targets, and hence identifying and developing novel chemical compounds that target new bilogical pathways is necessary [7]. Juvenile hormone binding protein (JHBP) is one of those potential targets being considered for insecticide development. The juvenile hormone system plays a pivotal role in mosquito development which includes, regulating metamorphosis, reproduction, and physiological adaptations [8]. In mosquitoes, the binding of juvenile hormone to its receptor proteins is crucial for maintaining the larval stage and preventing premature pupation. Interfering with this hormone signaling mechanism can lead to developmental abnormalities and mortality [9]. Thus, targeting the mosquito juvenile hormone binding protein (MJHBP), which mediates juvenile hormone action, offers a promising avenue for developing specific and effective insecticides.

The recent advancements in computational techniques have made it possible to accelerate identification of potential insecticidal compounds through *in silico* approaches [10]. Deployment of virtual screening strategies has revolutionized the identification of new bioactive molecules by evaluating *in silico* huge compound libraries against a bioreceptor, favoring the analysis of their chemical space, pharmacodynamics, and their pharmacokinetic properties [11]. The integration of chemoinformatics into insecticide development represents a paradigm shift towards more efficient, targeted, and environmentally responsible pest control solutions [12]. By harnessing computational tools, researchers can accelerate the discovery of novel insecticides, optimize their efficacy, and ensure their safety, thereby addressing the challenges posed by pest resistance and environmental concerns [10,13]. This has reduced the time, costs, and infrastructure required to find new chemical entities [11]. Thus, integration of cutting-edge chemoinformatics tools such as virtual screening and molecular docking not only accelerate the discovery of potent insecticidal compounds, but also enhances selectivity, cost-efficiency, and environmental safety, signaling a decisive shift toward smarter and more sustainable pest control strategies.

Virtual screening of huge compound databases against molecular targets has facilitated the exploration of extensive chemical spaces for chemicals with desirable properties [14]. For a successful screening process, it is important to start the process with compounds that have ideal attributes which could significantly increase the chances getting the right leads and subsequently right final products. Thus, a number of descriptors connected to oral bioavailability or membrane permeability such as log P, molecular weight, hydrogen donors and number of acceptors are used as filters during the initial stages of the screening process. The Tice’s rules, inspired by the Lipinski’s rule of five, provides guidelines of the physicochemical properties of compounds ideal for application as insecticides [15,16]. The Tice’s rules specify ranges for molecular mass, log P, rotatable bonds and hydrogen-bond donors and acceptors which confer insecticide-likeness to a given molecule. According to Tice’s rules, the guidelines for agrochemicals (insecticides and herbicides) and pharmaceuticals (Lipiski’s rule R05) are similar except that the former require fewer hydrogen bonds than the latter (Table 1) [15]. By following these recommendations, virtual screening becomes simpler and more focused. Herein, we report the identification and study of potential larvicidal compounds targeting mosquito juvenile hormone binding protein (MJHBP) through virtual screening, molecular docking and dynamics simulations.

**Table 1:** Rule based filters for pharmaceuticals and agrochemicals.

Descriptor (s)	Lipinski	Tice	
	*Drugs*	*Herbicides*	*Insecticides*
Molecular Mass	≤500	150-500	150-500
mlogP	≤5	≤3.5	0-5
H- bond donors	≤5	≤3	≤2
H-bond acceptors	≤10	2-12	1-8
Rotatable bonds	–	≤12	≤12

## 2. Materials and methods

### 2.1. Software and tools

In this study, we conducted the virtual screening of insecticidal compounds using a cheminformatics approach. A python-based workflow utilizing the RDKit and Pandas libraries was used to filter these insecticidal compounds obtained from Agrochemical Insecticides library based on their physicochemical properties in line with Tice rule (Table 1) [17]. The Tice rule is a set of guidelines, analogous to Lipinski’s Rule of Five for drugs, that helps predict the potential of a chemical compound to have insecticidal or herbicidal properties [15]. Briefly, the molecular dataset (SDF file) was imported into a Pandas DataFrame using RDKit’s Chem.SDMolSupplier and PandasTools.LoadSDF. Subsequently, molecular descriptors were computed with the RDKit Descriptors module, including molecular weight (MW), octanol–water partition coefficient (MolLogP), number of hydrogen bond donors (Donor), number of hydrogen bond acceptors (Acceptor), and rotatable bond count (RotBonds). The compounds were then filtered according to the guidelines based on the Tice rule for insecticides as discussed by Avram and coworkers and presented in Table 1 [15].

The defined criteria in Table 1 having been applied systematically, the last set of filtered molecules were exported in two formats for use in the subsequent study and analysis: a CSV file and an SDF file. In the SDF output, important properties such as `IDNUMBER` and `Chemical_Name” were retained by setting them as molecular properties using RDKit’s SDWriter module, making the SDF output compatible with downstream cheminformatics tools. This computational workflow presents a reproducible and efficient approach to prioritize agrochemicals compounds relevant for further biological assessment.

### 2.2. Ligand preparation

Ligand preparation of the Tice rule-filtered compounds was performed using LigPrep module in the Schrodinger Suite (Release 2021-2), to process the SDF file containing the screened molecules. The workflow generated multiple stereoisomers and tautomeric forms for each compound, with ionization states calculated at physiological pH 7.0 using Epik. Conformational sampling was conducted to generate low-energy 3D structures, with chirality retained from the original SDF when specified, or enumerated when undefined.

### 2.3. Protein preparation

Target protein target (pdb:5V13) structure was retrieved from the Protein Data Bank (https://www.rcsb.org/) (S1 File) [18]. Protein preparation was conducted using the Protein Preparation Wizard within Maestro, following a standardized protocol to ensure structural integrity and accuracy. Crystal structures underwent initial pre-processing to remove water molecules beyond 5 Å from heteroatoms, delete co-crystallized ligands, and eliminate non-essential ions. Missing side chains and loops were modelled using Prime, while hydrogen atoms were added and optimized using PROPKA at pH 7.4. Protonation states of ionizable residues were assigned based on the local electrostatic environment, and the overall structure was subjected to restrained energy minimization using the OPLS4 force field with heavy atom RMSD convergence criterion of 0.30 Å.

### 2.4. Receptor grid generation

Receptor grid generation was accomplished using the Receptor Grid Generation panel, with the binding site defined by co-crystallized ligand coordinates. The Grid boxes were centered on the identified binding sites with dimensions of 20 × 20 × 20 Å to accommodate ligand flexibility while maintaining computational efficiency. Van der Waals scaling factors were set to 1.0 for receptor atoms and 0.8 for ligand atoms to account for minor conformational adjustments during docking.

### 2.5. Molecular docking study

In this study, insecticidal compounds were systematically screened using a series of computational approaches to identify hit molecules with enhanced binding affinities relative to the co-crystallized ligand. A total of 2,874 compounds from the insecticide category of the agrochemicals’ library were initially filtered according to Tice’s rule, yielding 2,466 preliminary hits (Table 1) [17]. These hits were subsequently subjected to High-Throughput Virtual Screening (HTVS), which identified 1,607 promising lead compounds. The selected leads were further evaluated through molecular docking using the Glide protocol in Standard Precision (SP) mode, producing 39 top candidates. Glide SP employed systematic conformational sampling with the OPLS4 force field, generating up to 10,000 poses per ligand with post-docking energy minimization. The 39 top compounds based on docking scores (GlideScore ≤ −9.9 kcal/mol) were further subjected to the Extra Precision (XP) docking mode for enhanced accuracy and selectivity. Lastly, fifteen (15) highest ranking compounds (GlideScore ≤ −11.0 kcal/mol) were then selected for MMGBSA calculations. Validation of the docking protocol was carried out by extracting, preparing and re-docking of the co-crystalized ligand into the original active site of the target protein (5V13) to validate the reliability of the docking procedure.

### 2.6. Post docking analysis

Post-docking analysis included visual inspection using Maestro’s molecular visualization tools to evaluate binding poses, intermolecular interactions, and structural complementarity.

Binding affinity results were analyzed using the Schrödinger Suite’s integrated analysis tools, with compounds ranked according to Glide Score in comparison with the reference molecule. Interaction fingerprints were generated using the Interaction Fingerprint panel to characterize binding patterns and identify critical residues involved in ligand recognition. Top ranked molecules with better or close to reference molecule binding affinity were further subjected for free energy calculation, such as MMGBSA and MD simulations.

### 2.7. Molecular mechanics/generalized born surface area (MM/GBSA) calculations

MM/GBSA continuum solvent was employed in determining the best docked protein-ligand complex binding free energy. Rotamer search techniques from prime were used in conjunction with the OPLSE force field and the VSGB solvent model.


$$\begin{array}{c} G_{bind}\;=\;\Delta G_{complex}-\;\left(\Delta G_{protein}+\Delta G_{ligand}\right)\; \end{array}$$

$$\begin{array}{c} where:\;\Delta G_{complex}=Gibbs\;free\;energy\;of\;the\;protein-ligand\;complex. \end{array}$$

$$\begin{array}{c} \Delta G_{protein}\;=\;Gibs\;free\;energy\;of\;the\;protein.\; \end{array}$$

$$\begin{array}{c} \Delta G_{ligand}\;=Gibbs\;free\;energy\;of\;the\;ligand. \end{array}$$

### 2.8. Pesticide-likeness analysis (CoPLA)

Physicochemical properties of each of the hit compounds were obtained from the Agrochemicals library to assess their compliance with Tice’s rules of insecticide-likeness. Six key molecular descriptors were evaluated to determine their desirability, including molecular weight (MW), calculated lipophilicity (clogP), number of hydrogen bond donors (HBD), number of hydrogen bond acceptors (HBA), number of rotatable bonds (RB), and topological polar surface area (TPSA).

### 2.9. Toxicity and environmental hazard predictions

The crucial metrices essential for regulatory assessment of chemical risks including Bioconcentration factor (BCF), Biomagnification factor (BMF), Biodegradability, lethal concentrations of 50% fathead minnow (LC50−96 h) and daphnia magna (LC50−48 h) for the top compounds were evaluated using Alvascience prediction software based on QSAR [19].

### 2.10. Density functional theory (DFT) studies

Density Functional Theory (DFT) calculations were performed for the top-ranking compound based on the docking score (F3023-0929), MMGBSA values (F2708-0061) and the co-crystallized ligand. Molecular geometry optimization and property calculations of the selected compounds were performed using ORCA 5.0.4 program [20]. All the selected compounds were optimized using the DFT method with Perdew-Burke-Ernzerhof (PBE) functional and a basis set of def2-SVP. Initial optimization of all the selected compounds was performed in the gas phase by Avogadro v 4.2.1 software and saved as XYZ files in each of their dedicated folders. The ORCA input file was then created in each of the corresponding folders which specified the PBE functional, a geometry optimization step, a vibrational frequency and LARGEPRINT output. With the ORCA input file, the job was submitted in command prompt. ORCA read the geometry, performed the optimization under PBE, and then immediately executed the analysis to generate the ORCA output file in 2–3 hours. Highest occupied molecular orbital (HOMO) and lowest unoccupied molecular orbital (LUMO) were extracted from ORCA output file directly and IboView, Avogadro and Chemissian were used to visualize the structure of the molecules in detail. The HOMO-LUMO gap was calculated according to the following equation.


$$\begin{array}{c} \Delta \varepsilon_{Gap}=\varepsilon_{LUMO\;}-{\;\varepsilon }_{HOMO\;} \end{array}$$

Molecular electronic potential (MESP) maps of compounds F3023-0929, F2708-0061 and the co-crystallized were also generated by orca. Briefly, electron density of each of the molecules was calculated by orca leading to generation of a cube file of the MESP. The output MESP file was then visualized in Jmol viewer (Jmol). Finally, for each of the selected compounds, hardness (η), and softness (S), were calculated from the energies of frontier HOMO and LUMO according to Koopmans’ theorem on the correlation of ionization potential (*I*) and electron affinities (*A*) with HOMO and LUMO energy (*ε*) [21,22]. Hardness, softness, electronegativity (χ) and electrophilicity (ω) were calculated using the following equations;


$$\begin{array}{c} \eta \;=\;\frac{\text{1}}{\text{2}}(\varepsilon_{LUMO}-\varepsilon_{HOMO});\text{ S}=\frac{\text{1}}{\eta } \end{array}$$

$$\begin{array}{c} \;\chi =\;\frac{\text{1}}{\text{2}}(\varepsilon_{LUMO}+\varepsilon_{HOMO});\;\omega =\frac{\chi^{\text{2}}}{\text{2}\eta } \end{array}$$

### 2.11 Molecular dynamic simulations

The top-ranked docked molecules identified through molecular docking and MMGBSA calculation, along with the co-crystalized ligand, were subjected to 200 ns MD simulations performed with the Desmond module in the Schrodinger software suite. The simulation employed an explicit solvent model, incorporating Na^+^ and Cl^-^ ions to neutralize the system. Additionally, the system was carried out at 300º K and 1.01325 bar. An orthorhombic simulation box with a 10 × 10 × 10 Å buffer and 0.15M salt concentration was used to mimic physiological conditions, utilizing the transferable intermolecular potential with 4 points (TIP4P) water model. Following MD simulations, trajectories data for root mean square deviation (RMSD), root mean square fluctuation (RMSF), and the protein interaction were evaluated to determine the system/ligand-protein complex stability.

### 2.12. Larvicidal test of lead compounds against *Anopheles Arabiensis*

#### 2.12.1. Materials and chemicals.

The lead compounds, F3023-0929 and F2708-0061, were procured from the Life Chemicals online shop (https://lifechemicals.com). Analytical grade ethanol, used for sample preparation, was obtained from Sigma-Aldrich. Mosquito colonies were obtained from Kenya Medical Research Institute (KEMRI) and their larvae reared at the University of Nairobi Insectary.

#### 2.12.2. Larvicidal assay.

The larvicidal activity was tested based on the recommendations of the WHO guidelines for laboratory and field-testing of larvicides [23]. Both compounds were tested at 100 ppm, 50 ppm and 25 ppm, to get the activity range. Briefly, batches of twenty-five (25) third and fourth instar larvae were transferred into 100 ml beakers containing an appropriate concentration of the test solution and distilled water. Ethanol in distilled water was used as negative control. The experiment was conducted for 72 h period under optimal conditions (24–32°C temperature and 85% relative humidity) in the insectary. Each experiment was conducted in triplicates. The number of dead larvae were counted after a 24, 48 and 72 -hour exposure and the mortality rate calculated per replicate. Progression of the surviving larvae was monitored by tracking development milestones and behaviors for two weeks (14 days).

#### 2.12.3 Statistical analysis.

All experiments were done in triplicates, and mortality rates reported as mean values. The results were expressed as mean ± SE (standard error). The results were subjected to one-way ANOVA using Minitab 22.4.0.

## 3. Results and discussion

### 3.1. Molecular docking and MMGBSA analyses

**Fig 1:**
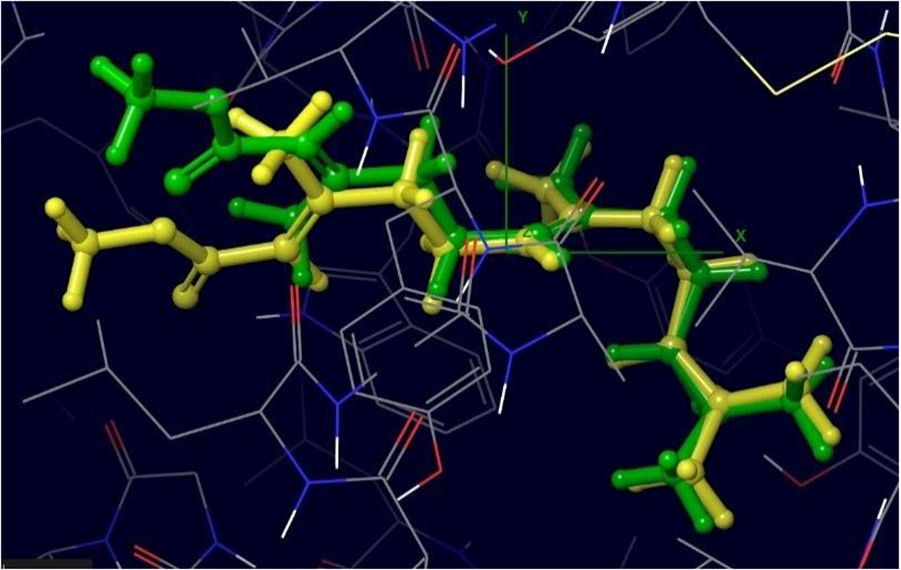
5V13 co-crystalized ligand overlapped at its binding domain.

Molecular docking, a widely recognized virtual screening technique, was employed in this study to predict potential drug targets and to analyze the interactions between ligands and the target protein at the atomic level [24]. The docking protocol was validated by redocking the co-crystallized ligand into the active site of the target protein. Fig 1 illustrates the superimposition of the re-docked ligand which exhibited a deviation of 1.6 Å in RMSD, demonstrating the accuracy and reliability of the docking protocol [24]. As presented in Table 2, the molecular docking analysis identified fifteen insecticidal compounds (15) with docking scores ranging from –11.2 to –11.8 kcal/mol. Docking score is a scoring function used to predict the binding affinity of the interaction between a ligand and the target protein [25]. The negative score signifies a favorable interaction, with more negative values indicating stronger binding. Therefore, the lowest (most negative) binding score suggests the highest likelihood of a stable and strong interaction between the protein and the ligand [26]. As shown in Table 2, all the hit molecules exhibited more negative binding scores than the co-crystallized ligand (–9.352 kcal/mol), indicating that these compounds have a stronger interaction potential with the mosquito juvenile hormone binding protein (MJHBP).This enhanced binding affinity may contribute to stronger stabilization of the active site and improved inhibition efficiency, thereby positioning them as promising, novel, and effective insecticides, necessary for vector control.

**Table 2:** Molecular docking and MMGBSA results of top 15 molecules from the agrochemical insecticides library and co-crystalized ligand, on Mosquito Juvenile Hormone-binding protein.

Molecule IDs	Chemical Name	Chemical class	Docking Score (kcal/mol)	MMGBSA (dG Bind) (kcal/mol)
F3023-0929	3-{[4-(dimethylamino)phenyl]amino}-1-(naphthalen-1-yl)pyrrolidine-2,5-dione	Succinimide derivative	−11.878	−64.26
F0466-0008	3-(2,5-dioxopyrrolidin-1-yl)-N-{naphtho[2,1-d] [1,3]thiazol-2-yl}benzamide	Benzothiazole–benzamide–succinimide hybrid	−11.749	−63.77
F1813-0484	N-(5,7-dimethyl-1,3-benzothiazol-2-yl)-3-(2,5-dioxopyrrolidin-1-yl)benzamide	Benzothiazole–benzamide–succinimide hybrid	−11.706	−76.57
F1813-0480	N-(7-chloro-4-methyl-1,3-benzothiazol-2-yl)-3-(2,5-dioxopyrrolidin-1-yl)benzamide	Benzothiazole–benzamide–succinimide hybrid	−11.677	−81.32
F2744-0810	N-(4-chloro-3-nitrophenyl)-1-[(3-methylphenyl)methyl]-6-oxo-1,6-dihydropyridine-3-carboxamide	Dihydropyridinone carboxamide	−11.672	−78.66
F1813-0481	N-(4,7-dimethyl-1,3-benzothiazol-2-yl)-3-(2,5-dioxopyrrolidin-1-yl)benzamide	Benzothiazole–benzamide–succinimide hybrid	−11.657	−75.33
F1813-0482	3-(2,5-dioxopyrrolidin-1-yl)-N-(4-methoxy-7-methyl-1,3-benzothiazol-2-yl)benzamide	Benzothiazole–benzamide–succinimide hybrid	−11.653	−79.2
F1065-0366	1-benzyl-3-{[4-(piperidin-1-yl)phenyl]amino}pyrrolidine-2,5-dione	Succinimide derivative	−11.643	−68.83
F2744-1400	N-(4-chloro-3-nitrophenyl)-1-[(2-fluorophenyl)methyl]-6-oxo-1,6-dihydropyridine-3-carboxamide	Dihydropyridinone carboxamide	−11.595	−78.67
F1813-0479	N-(4,7-dichloro-1,3-benzothiazol-2-yl)-3-(2,5-dioxopyrrolidin-1-yl)benzamide	Benzothiazole–benzamide–succinimide hybrid	−11.558	−85.75
F1065-0552	1-(3-chlorophenyl)-3-{[4-(morpholin-4-yl)phenyl]amino}pyrrolidine-2,5-dione	Succinimide derivative	−11.509	−66.72
F2744-1046	N-(4-chloro-3-nitrophenyl)-1-[(2,5-dimethylphenyl)methyl]-6-oxo-1,6-dihydropyridine-3-carboxamide	Dihydropyridinone carboxamide	−11.491	−75.16
F2708-0061	N-(7-chloro-4-methoxy-1,3-benzothiazol-2-yl)-3-(2,5-dioxopyrrolidin-1-yl)benzamide	Benzothiazole–benzamide–succinimide hybrid	−11.439	−89.51
F1751-0027	1-(naphthalen-1-yl)-3-[2-(4-nitrophenyl)hydrazin-1-yl]pyrrolidine-2,5-dione	Succinimide derivative	−11.289	−49.84
F2744-0456	N-(4-chloro-3-nitrophenyl)-1-[(2-methylphenyl)methyl]-6-oxo-1,6-dihydropyridine-3-carboxamide	Dihydropyridinone carboxamide	−11.206	−80.39
cocrystalized ligand	methyl (2E,6E)-9-[(2R)-3,3-dimethyloxiran-2-yl]-3,7-dimethylnona-2,6-dienoate	Epoxide-containing ester (terpenoid derivative)	−9.352	−75.83

The identified hit compounds belong mainly to three chemical classes which include succinimide derivatives, benzothiazole–benzamide–succinimide hybrids, and dihydropyridinone carboxamides. Succinimide derivatives are cyclic imides with two carbonyl groups bound to a nitrogen atom [27]. The benzothiazole–benzamide–succinimide hybrids are a class of compounds that integrates three structural units, the benzothiazole, benzamide, and succinimide within a single structure [28]. Dihydropyridinone carboxamides, on the other hand, are amide-substituted nitrogen heterocycles derived from partially saturated pyridine rings [29]. Collectively, these classes of Nitrogen containing compounds are unified by the presence of amide-type linkages and heterocyclic nitrogen atoms, features commonly associated with strong biological activity and target-binding potential [30]. Accordingly, most of these hit compounds exhibited a stronger binding interaction and higher affinity for the mosquito juvenile hormone compared to the co-crystallized ligand, as reflected by their superior docking scores and MMGBSA values, suggesting enhanced biological activity and binding potential (Table 2).

**Fig 2:**
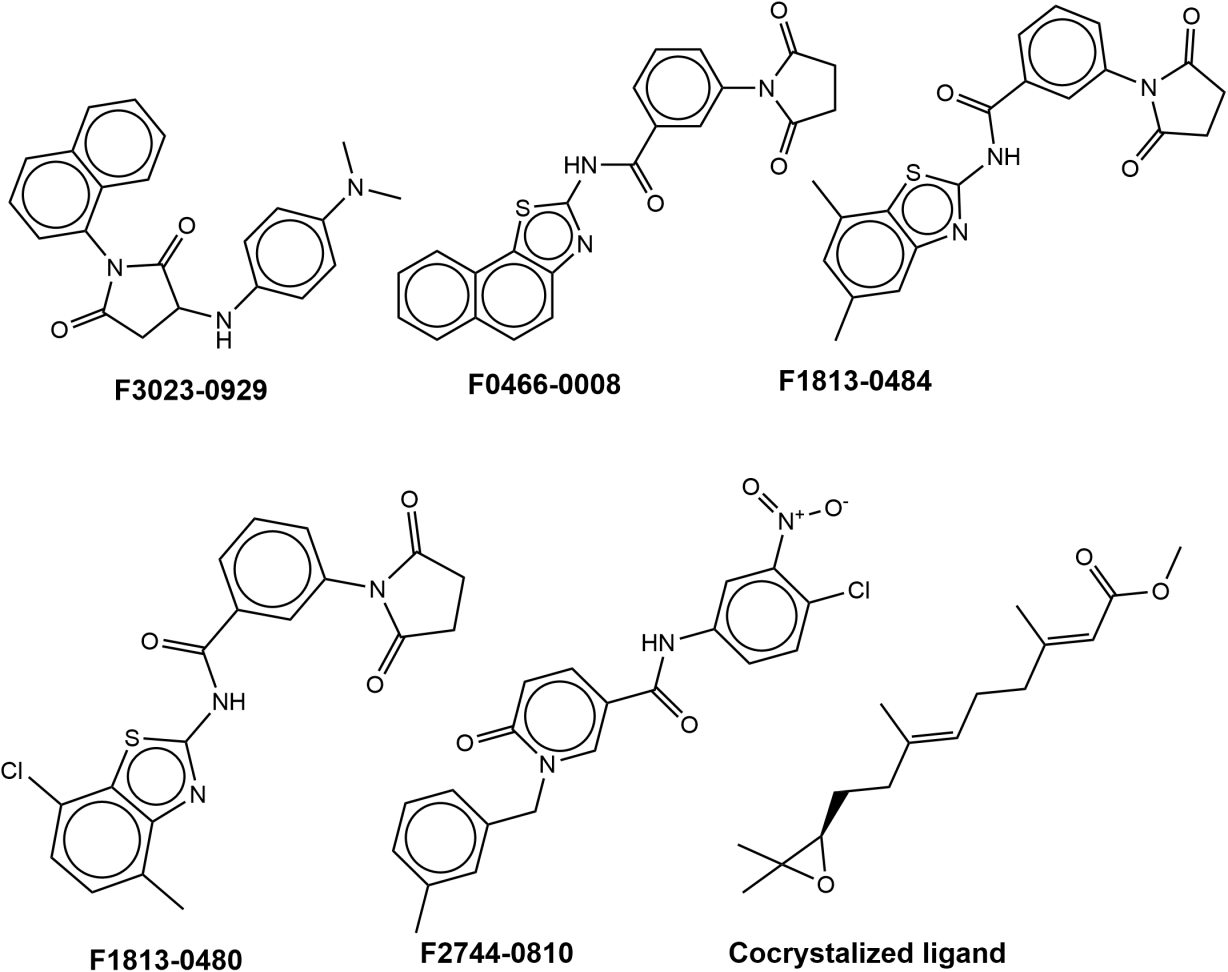
Chemical structures of the co-crystallized ligand and the top five compounds based on docking scores.

Among the identified hit compounds, F3023-0929 exhibited the highest docking score of −11.878kcal/mol followed by compounds F0466-0008, F1813-0484, F1813-0480 and F2744-0810 in that order as presented in Table 2. The chemical structures of these compounds are shown in Fig 2. Although compound F3023-0929 exhibited the highest docking score, its MM-GBSA binding free energy value was comparatively lower than that of other top compounds (Table 2). MMGBSA estimates how stable the ligand-protein interaction is under near physiological conditions while taking into account solvent effects, entropic contributions and protein flexibility, unlike docking score which only estimates how well the ligand fits into the active site without considering solvation and energetic balance [28,29]. Thus, the discrepancy suggests that, despite a strong initial docking interaction, the overall binding stability or energetic favorability of F3023-0929 within the active site may be relatively weaker when solvent effects and energy factors are considered. A more negative MMGBSA values indicate more stable and stronger binding suggesting higher binding affinity [31]. Accordingly, compound F2708-0061 exhibited the highest binding affinity with MMGBSA value of (−89.51 kcal/mol), followed by compounds F1813-0479, F1813-0480, F2744-0456 and F1813-0482.

### 3.2 Post-docking Analysis

**Fig 3:**
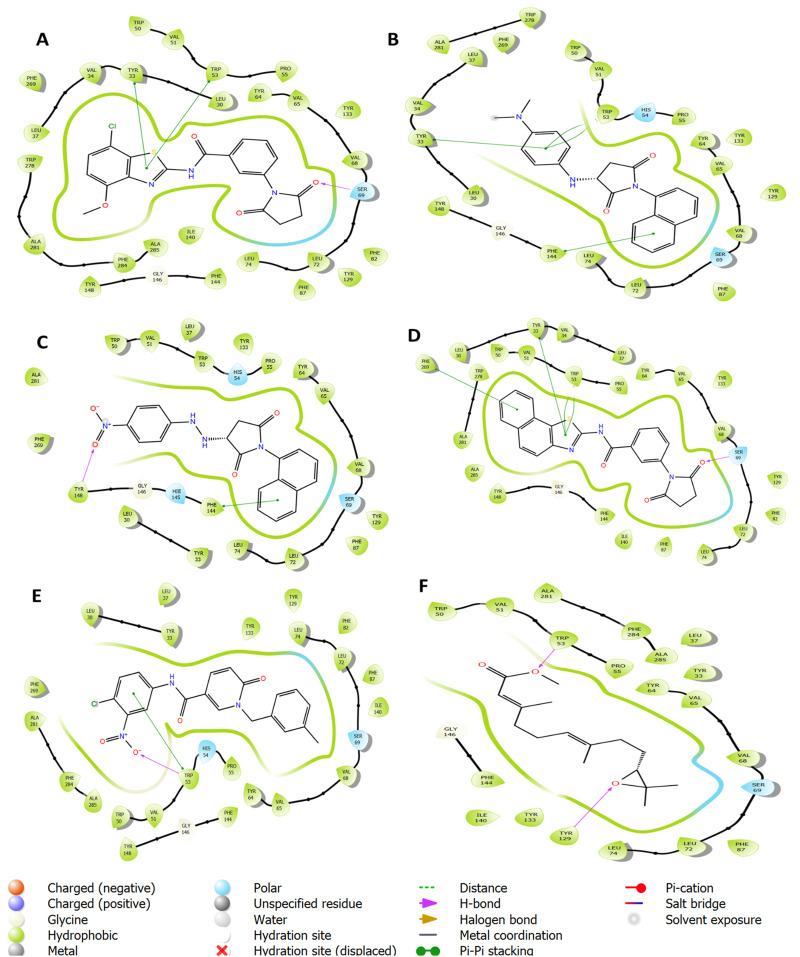
2D-Molecular interactions of amino-acid residues of 5V13 with the top docking compounds F3023-0929 (A), F0466-0008 (B), F1813-0484 (C), F1813-0480 (D), F2744-0810 (E) and the co-crystalized ligand (F).

The post-docking analysis identified significant interactions between the hit molecules and the protein target, including hydrogen bonds, hydrophobic interactions, and electrostatic interactions. The hydrogen bonds and hydrophobic interactions of the top five hit compounds with the target protein are shown in Fig 3 and Table 3. The top compound F3023-0929 (based on docking score) did not form hydrogen bonding interactions but instead formed π−π stacking interactions with TRY33, TRP53, and PHE144 in addition to hydrophobic interactions with the amino acid residues at the active site of the mosquito juvenile hormone-binding protein (Table 3). Compound F0466-0008 with the second highest score on the other hand formed one hydrogen bonding interactions with SER69, π−π  stacking interactions with TYR33, TRP53 and PHE269 in addition to hydrophobic interactions with amino acid residues. The C=O group that is joined to the five-membered heterocyclic ring (succinimide) in the chemical structure of F0466-0008 which plays a role of a hydrogen bond acceptor, because of the available lone pairs on the electronegative oxygen atom (Fig 3). The partial positive charge on the donor hydrogen and the partial negative charge on the carbonyl oxygen often stabilize this binding relationship, which positively affects the protein-ligand complex’s total binding energy and stability [32]. The 2D-molecular interactions of amino-acid residues of 5V13 with compounds F3023-0929 and F0466-0008 are shown in Fig 3.

**Table 3:** Intermolecular interactions of the top hit compounds and the co-crystallized ligand with the amino acids at the active sites of 5V13.

Molecule IDs	H-bond	Hydrophobic interacting amino acids	Other interactions
F3023-0929	None	ALA 281, TRP 278, PHE 269, LEU 37, VAL 34, TYR 33, LEU 30, TYR 148, PHE 144, LEU 74, LEU 72, PHE 87, VAL 68, TYR 129, VAL 65, TYR 64, TYR 133, PRO 55, TRP 53, VAL 51, TRP 50	Pi-Pi Stacking: PHE 144, TYR 33, TRP 53 (2)
F0466-0008	SER 69	LEU 30, TYR 33, VAL 34, LEU 37, PHE 269, TRP 278, ALA 281, TRP 50, VAL 51, TRP 53, PRO 55, TYR 64, VAL 65, TYR 133, VAL 68, TYR 129, PHE 82, LEU 72, PHE 87, LEU 74, ILE 140, PHE 144, TYR 148, ALA 285	Pi-Pi Stacking: TRP 53 (2), TYR 33, PHE 269
F1813-0484	None	LEU 30, TYR 33, VAL 34, LEU 37, TRP 50, VAL 51, TRP 53, PRO 55, TYR 64, VAL 65, TYR 133, TYR 129, VAL 65, VAL 68, LEU 72, LEU 74, PHE 87, ILE 140, PHE 144, TYR 148, ALA 285, VAL 282, ALA 281, TRP 278	Pi-Pi Stacking: TRP 53 (2), TYR 33
F1813-0480	SER 69	PHE 269, LEU 37, VAL 34, TYR 33, LEU 30, TYR 64, VAL 65, TYR 133, VAL 68, TYR 129, LEU 72, LEU 74, PHE 82, PHE 87, PHE 144, ILE 140, TYR 148, PRO 55, TRP 53, VAL 51, TRP 50, ALA 285, PHE 284, ALA 281, TRP 278	Pi-Pi Stacking: TRP 53 (2), TYR 33
F2744-0810	TRP53	LEU30, TYR33, LEU37, TYR133, TYR129, LEU74, PHE82, LEU72, PHE87, ILE140, VAL68, VAL65, TYR64, PHE144, GLY146, TYR148, PHE269, ALA281, PHE284, ALA285, TRP50, VAL51, TRP53, PRO55	Pi-Pi Stacking: TRP 53
Co-crystallized ligand	TYR53, TYR129	ALA281, PHE284, ALA285, LEU37, TYR33, TRP50, VAL51, TRP53, PRO55, TYR64, VAL65, VAL68, PHE87, LEU72, LEU74, TYR129, TYR133, ILE140, PHE144, GLY146	None

Compound F1813-0484 exhibited a binding profile dominated by hydrophobic contacts with residues LEU30, TRP53, PHE144, and TYR148, and notable π–π stacking with TRP53 and TYR33, despite the absence of hydrogen bonds. This indicates that van der Waals and aromatic interactions are key contributors to its stability within the binding cavity. Compound F1813-0480 formed a hydrogen bond with SER69, alongside strong hydrophobic interactions with PHE269, TYR33, TRP53, and TYR148. The dual π–π stacking interactions with TRP53 and TYR33 suggest effective aromatic stabilization similar to F0466-0008, reflecting a comparable binding mode. Compound F2744-0810 displayed a hydrogen bond with TRP53, supported by extensive hydrophobic interactions with key residues such as TYR133, TYR148, PHE144, and ALA285. The π–π stacking with TRP53 reinforces its stable binding within the active site pocket. In comparison, the co-crystallized ligand formed hydrogen bonds with TYR53 and TYR129, and maintained hydrophobic contacts with residues ALA281, TRP50, TYR64, PHE87, and PHE144, indicating a well-conserved interaction network within the active site. Overall, the top hit compounds mimic several crucial hydrophobic and aromatic interactions observed in the co-crystallized ligand, suggesting strong binding complementarity. Particularly, interactions involving TRP53, TYR33, PHE144, and TYR148 appear to be critical for ligand stabilization and may serve as anchor points for structure-based optimization.

### 3.3. Pesticide-likeness Analysis (CoPLA)

Based on Tice’s rule for insecticide-likeness, which suggests that potent insecticidal molecules should possess a MW ≤ 500 Da, clogP ≤ 5, hydrogen bond acceptors (HBA) ≤ 8, hydrogen bond donors (HBD) ≤ 2, rotatable bonds (RotBonds) ≤ 12, and a topological polar surface area (TPSA) below 140 Å² [15], the top hit compounds from the agrochemical database met the required physicochemical thresholds (Table 4). The molecular weights of the selected compounds range between 359.42 and 420.27 Da, indicating moderate molecular size favorable for membrane permeability. Their clogP values (2.42–4.29) fall within the optimal lipophilicity range for effective bioavailability and insecticidal activity. Similarly, all compounds possess acceptable numbers of hydrogen bond donors (1–2) and acceptors (4–5), supporting good molecular interactions with biological targets. The number of rotatable bonds (3–5) suggests adequate molecular flexibility without compromising structural stability, while the TPSA values (52.65–116.84 Å²) remain within the preferred range for efficient absorption and transport across insect membranes. Collectively, these attributes demonstrate that the screened molecules conform well to Tice’s rule, making them suitable candidates with promising insecticidal potential.

**Table 4:** Pesticide likeness analysis (CoPLA) of the top compounds using the Tice rule.

Molecule IDs	MW	clogP	HBA	HBD	RotBonds	TPSA
F3023-0929	359.42	3.83	4	2	4	52.65
F0466-0008	401.44	2.8	4	1	3	107.61
F1813-0484	379.43	2.62	4	1	3	107.61
F1813-0480	399.85	2.84	4	1	3	107.61
F2744-0810	397.81	3.85	4	1	5	92.55
F1813-0481	379.43	2.62	4	1	3	107.61
F1813-0482	395.43	2.42	5	1	4	116.84
F1065-0366	363.45	4.07	4	2	5	52.65
F2744-1400	401.78	3.49	4	1	5	92.55
F1813-0479	420.27	3.06	4	1	3	107.61
F1065-0552	385.84	2.94	5	2	4	61.88
F2744-1046	411.84	4.29	4	1	5	92.55
F2708-0061	415.85	2.61	5	1	4	116.84
F1751-0027	376.37	3.62	5	2	5	104.58
F2744-0456	397.81	3.8	4	1	5	92.55

### 3.4 Toxicity and environmental hazard predictions

The toxicity and environmental hazard predictions obtained from the Alvascience platform(https://www.alvascience.com/alva/qsar/) [19] revealed that all the screened compounds were non-readily biodegradable (NRB), indicating potential environmental persistence. All the hit compounds demonstrated low bioaccumulation potential, with bioconcentration factor (BCF) values (4.055–54.954 L/kg) compared to the regulatory thresholds (≥ 2000 L/kg for *bioaccumulative*, and reported Biomagnification Factor (BMF) values that are substantially below unity (0.000174–0.0006839), clearly indicating that the compounds under investigation do not biomagnify through the food chain (Table 5). Bioconcentration and biomagnification both describe the accumulation of chemicals in organisms, but they refer to different uptake pathways and ecological scales. While BCF measures uptake directly from water, BMF quantifies the increase of a chemical’s concentration in a predator compared to its prey, representing a step-by-step increase through the food chain [33]. According to different environmental regulatory bodies, chemicals with BCF values *<*2000 L/kg are not considered significant for bioaccumulation potential [34]. Also, a BMF > 1 indicates that the chemical’s concentration in the predator is higher than that in its prey, signifying that the chemical has been concentrated through the food chain [35]. These results imply that although the compounds may not degrade readily, their low potential for bioaccumulation and trophic transfer strengthens their ecological safety profile.

**Table 5:** Biodegradability, bioconcentration factor (BCF), Biomagnification Factor (BMF), LC₅₀–96 h and LC₅₀–48 h for the top compounds aspredicted by Alvascience prediction software.

Molecule IDs	RB	LC50-96h (mg/L)	LC50-48h (mg/L)	BMF	BCF (L/kg)
F6482-2348	NRB	3.131	9.145	0.000174	4.055
F1813-0481	NRB	2.993	0.083	0.0002061	15.101
F1813-0484	NRB	2.993	0.047	0.0002032	15.276
F2708-0061	NRB	2.582	0.020	0.0002223	19.011
F1813-0480	NRB	0.855	0.044	0.0003243	31.046
F1813-0482	NRB	5.472	0.080	0.0006808	10.740
F1813-0479	NRB	0.309	0.023	0.0002443	54.954
F1813-0483	NRB	7.300	0.060	0.000156	6.653
F1065-0552	NRB	4.310	0.917	0.0006839	17.258
F0466-0008	NRB	2.268	0.021	0.0001435	18.836
F3023-0929	NRB	3.074	0.173	0.0002339	13.614
F1065-0366	NRB	3.536	1.293	0.0003162	12.078
F2744-0456	NRB	1.124	0.587	0.0004467	30.269

### 3.5. Density functional theory (DFT) studies

**Fig 4:**
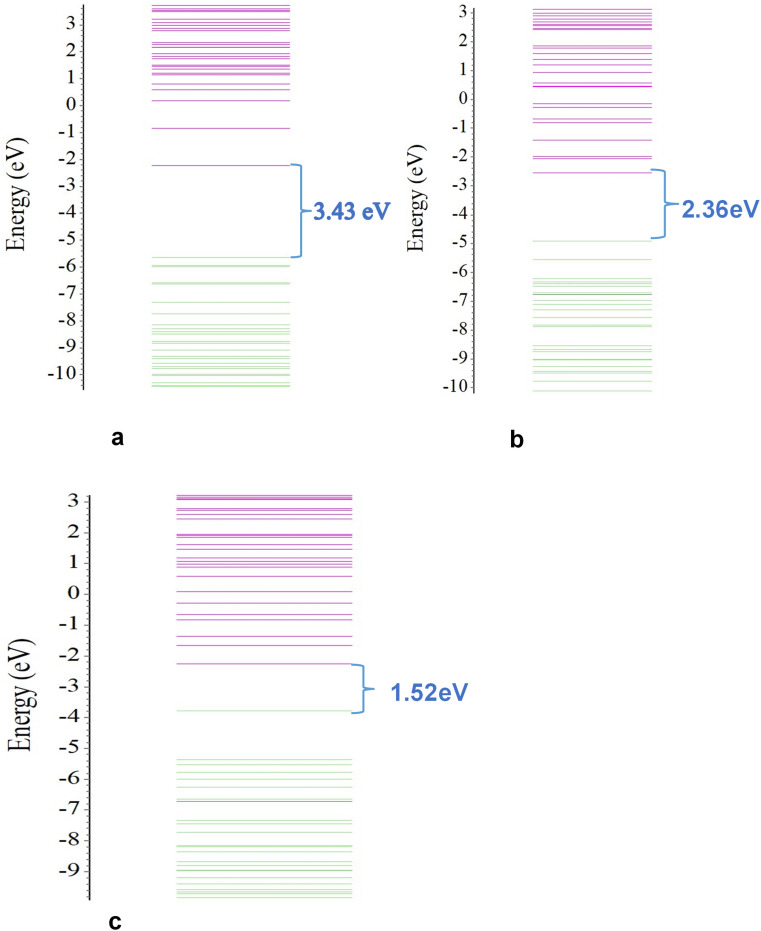
The HOMO-LUMO band gap for the co-crystallized ligand (a), F2708-0061 (b) and F3023-0929 (c) visualized in Chemissian.

The frontier molecular orbitals’ (FMO’s) energy gap (ΔE_HOMO-LUMO_) provides important information about a compound’s electron donating/receiving ability, chemical reactivity and consequently kinetic stability of its structure [35]. Lower energy gap values indicate that the molecule under study is highly chemically reactive, biologically active, and polarizable [36]. From our DFT results shown in Table 6 and Fig 4, F3023-0929 had the smallest HOMO-LUMO energy gap (1.52 eV), which implied greater chemical reactivity, followed by F2708-0061 (2.36 eV) and finally the co-crystallized ligand. The electrophilicity index followed the same trend, with F3023-0929 having the highest value (5.96), followed by F2708-0061 (5.93), and the co-crystallized ligand (4.51). Higher electrophilicity index indicates that the molecule possesses a higher binding capacity with biomolecules and a good electrophilic property [36]. Additionally, the lowest hardness (η) and largest softness (σ) correspond to the lowest Δε_gap_ values as recorded in Table 6 [22]. In our study F3023-0929 had the lowest hardness and highest softness when compared to the other compounds under study. The DFT results relating to energy gap, electrophilicity, hardness and softness of the studied molecules compliment the docking scores where F3023-0929 with the smallest energy gap had the highest docking score, followed by F2708-0061 and the co-crystallized ligand in that order. These DFT results offer insights into molecular reactivity and stability of the top candidates but do not directly correlate with binding affinity or insecticidal potency.

**Table 6:** Energy of HOMO, LUMO, εGap, G-E(1e), hardness and softness of the F3023-0929 (highest docking score), F2708-0061 (highest MMGSA) and the co-crystalized ligand.

Molecule IDs	εHOMO (eV)	εLUMO (eV)	GAP (eV)	η	S	ω	G-E(1e) (Kcal/mol)
F3023-0929	−3.77	−2.25	1.52	0.76	1.32	5.96	206.90
F2708-0061	−4.92	−2.56	2.36	1.18	0.85	5.93	153.93
Co-crystallized ligand	−5.65	−2.22	3.43	1.72	0.58	4.51	191.88

Additional insights from Table 6 indicate that molecule F3023-0929 exhibited the highest Gibbs free energy minus electronic energy (G–E(1e)) value (206.90 kcal/mol), followed by the co-crystallized ligand (191.88 kcal/mol) and then F2708-0061 (153.93 kcal/mol). According to Orca’s description [37] and thermodynamics, G–E(1e) is defined as the non-electronic contribution to Gibbs free energy of a molecule/ligand in its ground state [38]. It reflects how much the molecular Gibbs energy is increased due to nuclear motion and entropy. The higher G–E(1e) value for F3023-0929 suggests that this ligand possesses greater conformational flexibility and higher entropy in its unbound state compared to the other two compounds. Upon binding, such flexible ligands experience a significant loss of entropy, which contributes unfavorably to the overall binding free energy [39,40]. This entropic penalty can diminish binding stability, resulting in lower MMGBSA binding free energy estimates despite favorable initial docking scores. Conversely, F2708-0061, which exhibited the lowes]t G–E(1e) value, is likely more rigid and conformationally constrained. This reduced flexibility limits entropy loss upon binding, allowing the molecule to maintain stronger and more stable interactions with the protein target and this is reflected in its more favorable MMGBSA value (–89.51 kcal/mol). In contrast, F3023-0929, despite achieving the highest initial docking score (–11.878), showed a less favorable MMGBSA binding free energy (–64.26 kcal/mol) compared to the co-crystallized ligand (–75.83) and F2708-0061 (−89.51), highlighting the importance of entropic contributions in realistic binding energy predictions. Although useful for understanding molecular stability and thermodynamic behavior, G–E(1e) values do not directly predict biological activity.

**Fig 5:**
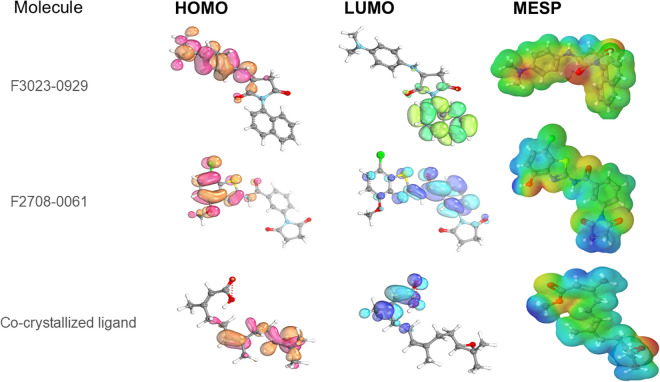
Images of HOMO, LUMO (Visualized in Iboview) and MESP (Visualized in Jmol).

The resultant HOMO, LUMO and molecular electronic potential (MESP) maps of compounds F3023-0929, F2708-0061 and the co-crystallized ligand are demonstrated in Fig 5. A molecule’s interactions with other molecules can be reliably predicted by using the MESP, which is based on characterization of the molecule in terms of electron-rich and -deficient areas [41]. In the MESP visualized in Jmol viewer, red typically represents negative potential, blue represents positive potential, while green represents neutral potential. According to MESP maps of compounds F3023-0929, F2708-0061 and the co-crystallized ligand in Fig 5, the negative potential regions are localized over the electronegative atoms (oxygen and nitrogen) while the positive potential regions are localized over the hydrogen atoms. The negative potentials regions near the oxygen and nitrogen atoms are possible hydrogen bond (HB) acceptor sites of the molecule whereas the positive potential regions are probable HB donor sites [42]. Finally, a closer look at the frontier molecular orbitals reveals that the HOMO orbitals of both F3023-0929 and F2708-0061are mostly located around the heteroatoms while the LUMO orbitals are located mostly around the hydrocarbon part of the molecule.

### 3.6. Molecular dynamics (MD) simulations

In this study, we used a 200-nanosecond simulation to investigate the atomic-level interactions between the target protein and the top compound predicted by the molecular docking study. This interaction was compared with the co-crystalized ligand-protein interaction. The simulation made it possible to evaluate the flexibility and conformational changes of both complexes, increasing the likelihood of more precise drug binding site prediction and the ability to refine and optimize drug candidates for better therapeutic outcomes [43]. The atomic deviations, fluctuations, radius gyrations, solvent accessible surface area (SASA), molecular surface area (MolSA) polar surface area (PSA) and protein-ligand interactions of the top ligand were compared to the co-crystallized-protein interaction in this study.

#### 3.6.1. Root mean square deviation (RMSD).

The RMSD is the rate of the mean distance between the atoms (usually the backbone atoms) of superimposed molecules, the RMSD examines the equilibration and the structural stability of the target protein in the presence of a docked ligand [44]. The RMSD examines the dynamics and function and understands how the proteins bind with small molecules [45]. The value is an indication of the differences in the protein backbone and the stability of the protein backbone and protein-ligand complex [25]. Important metrics for evaluating the quality and stability of molecular interactions are ligand RMSD and protein RMSD. The stability of the ligand’s location and orientation within the protein’s binding site is reflected in the ligand RMSD, whereas the protein RMSD shows how much the protein structure deviates from its initial configuration over time [46].

**Fig 6:**
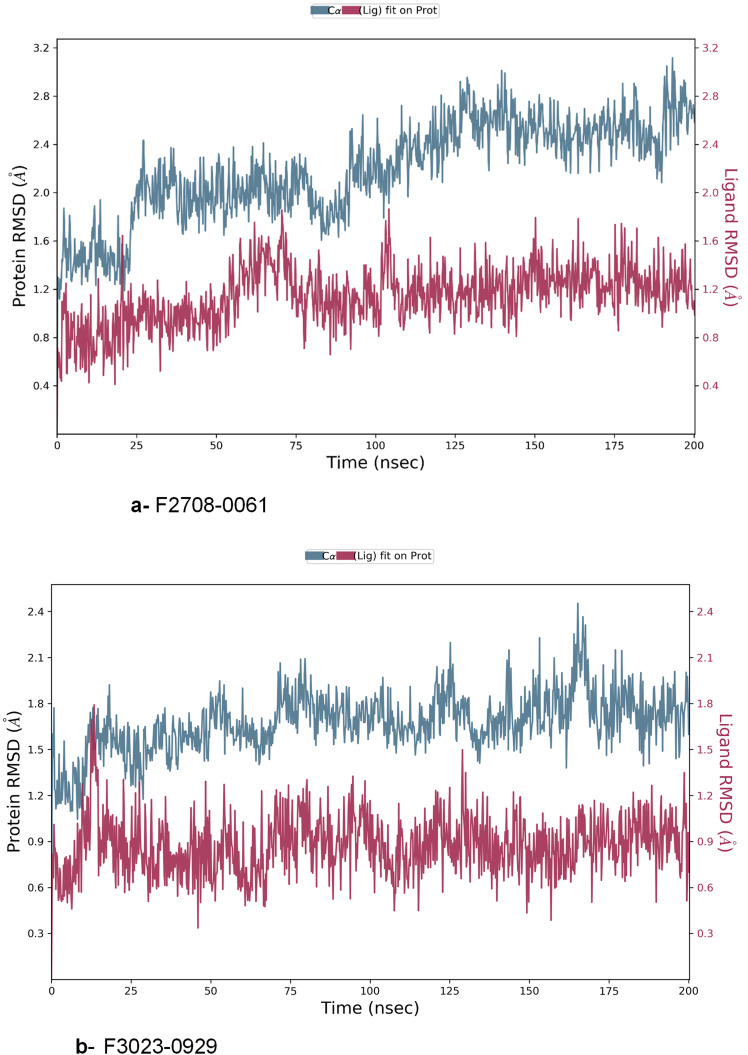
Schematic illustration of 200 ns MD Simulation graph of root-mean-square deviation (RMSD) of compounds F2708-0061 (a) and F3023-0929 (b).

The left y-axis of the Fig 6 represents the protein’s RMSD, while the right y-axis displays the ligand’s RMSD profile matched with the protein backbone. The ligand’s RMSD values in relation to the protein backbone are referred to as “Lig Fit Prot” and offer further details regarding the ligand’s conformational changes and structural compatibility with the protein. A ligand’s flexibility or mobility in relation to the protein backbone is indicated by “Lig Fit Prot” values, which are often marginally higher than the protein RMSD. A much higher number, on the other hand, can indicate significant alterations in the ligand’s posture in comparison to its original docked position. Such variances might point to substantial conformational changes inside the binding pocket and a less stable interaction between the ligand and the protein, both of which could affect the ligand’s capacity to attach to its target [47]. The RMSD profile of F2708-0061, F3023-0929, and the reference ligand demonstrated the overall structural stability of the protein-ligand complexes during the 200 ns simulation period.

In Fig 6(a), the protein RMSD (Cα) of the ligand F2708-0061 showed an initial stabilization between 0 ns to 25 ns; RMSD increased from around 1.5 Å to approximately 2.2 Å, suggesting that the protein was equilibrated in the solvent. Between 50 and 150 ns, the RMSD plateaued and varied between 2.1 and 2.6 Å, indicating stable structure during the middle phase (Fig 6). A modest rise and minor convergence with the ligand RMSD (2.8 Å) were also seen in the last phase between 185 and 200 ns, which may indicate conformational convergence or pocket rearrangement. The protein’s overall structural integrity is maintained throughout the simulation, and its RMSD values fall within the range that is predicted for a well-folded protein (<3.0 Å) (Fig 6).

According to Fig 6a, the RMSD of ligand F2708-0061 remained relatively stable with moderate oscillations raging between 0.4 Å to 1.2 Å throughout the simulation time frame. This indicate that the compound maintained a consistent orientation within the active binding pocket. Similarly, compound F3023-0929 displayed stable RMSD behavior during the simulation period. After an initial adaptation phase, of oscillation between 1.2 Å to 1.5 Å, the protein -ligand complex achieved equilibrium and maintained a relatively constant RMSD trajectory oscillating between 1.6 Å and 1.8 Å throughout the simulation period. The ligand RMSD exhibited initial oscillation from 0.6 Å to 1.8 Å, from 0 ns to around 20 ns. However, after initial adaptation phase, the ligand’s RMSD remained closely aligned with the protein RMSD, oscillating around 0.9 Å all through the simulation time. This indicates that ligand remained tightly associated with the binding site rather than diffusing away from the receptor. This observation demonstrates a stable binding mode and supports the potential inhibitory capability of the compounds.

**Fig 7:**
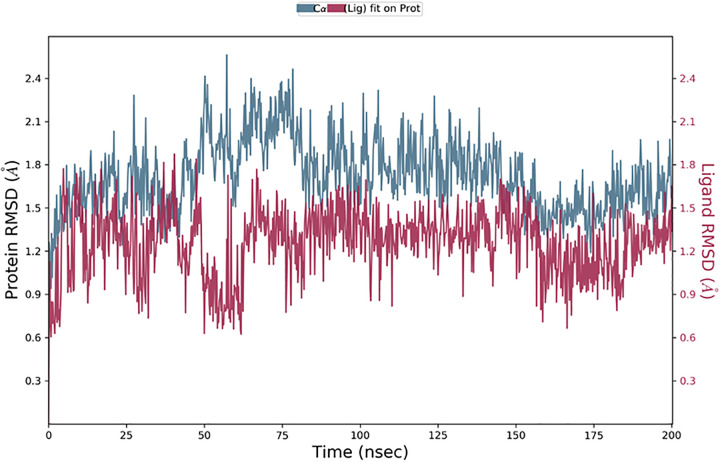
Schematic illustration of 200 ns MD Simulation graph of root-mean-square deviation (RMSD) of the reference ligand.

Comparing the protein and ligand RMSD plots of the top ligand with the reference ligand, Fig 7 presents an overview of the reference ligand’s protein RMSD, which had an initial phase from zero (0) ns to 50 ns, with protein RMSD values oscillating between 0.9 Å and 2.1 Å. This was anticipated during the early equilibration. We also observed a few tiny peaks and troughs in the range of 2.1 Å to 2.4 Å. This shows that the protein is in a stable folded state with no significant conformational changes and greater structural stability because the RMSD values are below the 3.0 Å threshold. For the ligand RMSD, the values increased from 0.6 Å to 1.5 Å, suggesting early repositioning within the binding site. Additionally, we noticed a major stabilization between 60 ns and 200 ns, with the ligand RMSD values stabilizing between 0.9 Å to 1.5 Å. The fluctuations were moderate indicating stable binding with slight conformational adjustments. The reference ligand demonstrated stable RMSD behaviour throughout the simulation, consistent with the expected stability of a validated co-crystallized ligand. Overall, the comparable RMSD stability observed for F2708-0061 and F3023-0929 suggests that both investigated compounds exhibit dynamic stability similar to that of the standard ligand. This finding is highly significant, as it indicates that the compounds are capable of maintaining prolonged occupancy within the active site under simulated physiological conditions

#### 3.6.2. Root mean square fluctuation (RMSF).

**Fig 8:**
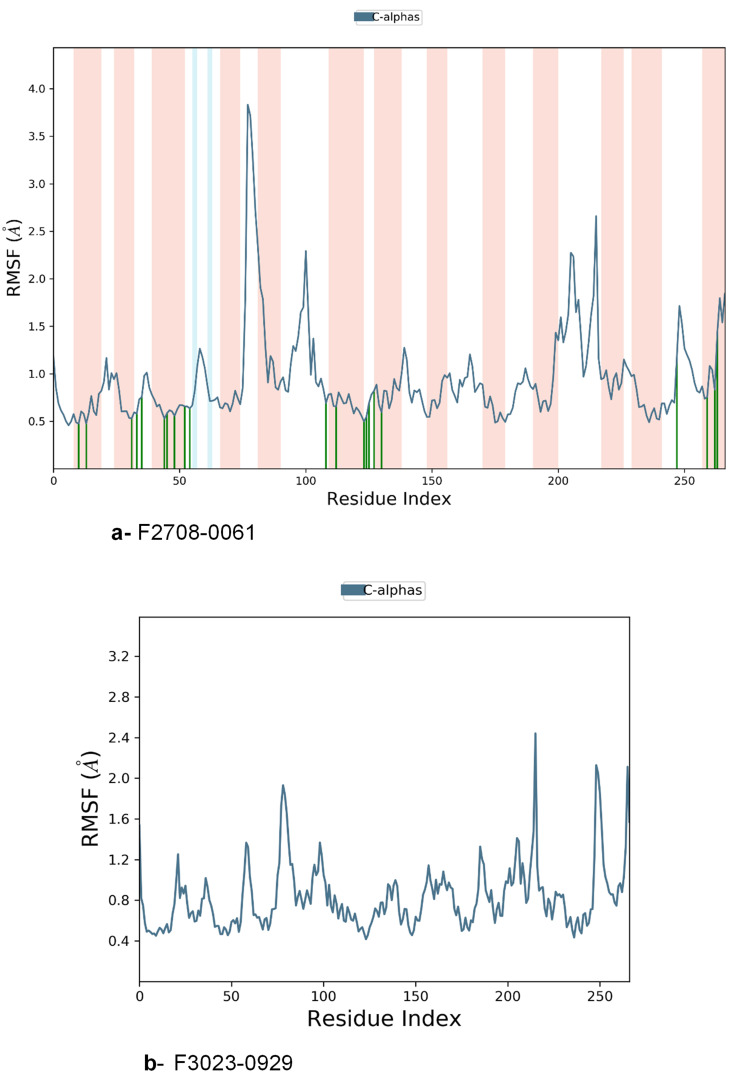
Schematic illustration of 200 ns MD Simulation graph of root-mean-square fluctuation (RMSF) of compounds F2708-0061(a) and F3023-0929(b).

The root mean square fluctuation (RMSF) is a metric used to quantify the average variation of each atom’s location from its mean over many configurations or throughout a simulation. It provides insights into the mobility and flexibility of residues; more mobility or flexibility is indicated by higher RMSF value [47]. In the case of ligand F2708-0061 (Fig 8(a)), the RMSF revealed generally low fluctuation across most residues, indicating that the protein maintained structural integrity during ligand binding. Notable fluctuation was observed with residue index around 75 fluctuating up to 3.8 Å, 210, 220, and 250 residues fluctuated between 1.2 Å to 2.6 Å. However, majority of the residues, exhibited minimal fluctuation of below 1.0 Å suggesting that ligand binding stabilized the catalytic pocket and reduced local mobility. Therefore, low fluctuations suggest that the protein region is relatively rigid and stable during a simulation.

Ligand F3023-0929 (Fig 8(b)) also demonstrated acceptable RMSF behaviour with moderate fluctuations observed primarily in loop and terminal regions rather than in the catalytic domain. This include residue index 80 with fluctuation of up to 2.0 Å, residue 210,250 and 270 with fluctuation rising up to 2.5 Å, 2.4 Å and 2.3 Å respectively. However, all the other residues fluctuated below 1.5 Å indicating that the ligand effectively stabilized the active site architecture while allowing limited flexibility in peripheral protein regions. Such flexibility is often beneficial because it permits adaptive fitting of the ligand without disrupting the core protein structure. Overall, the RMSF analyses indicate that both F2708-0061 and F3023-0929 maintained the structural stability of the protein throughout the simulation. Although localized fluctuations were observed mainly within loop and peripheral regions, the catalytic domain remained comparatively stable, with most residues exhibiting low fluctuation values.

**Fig 9:**
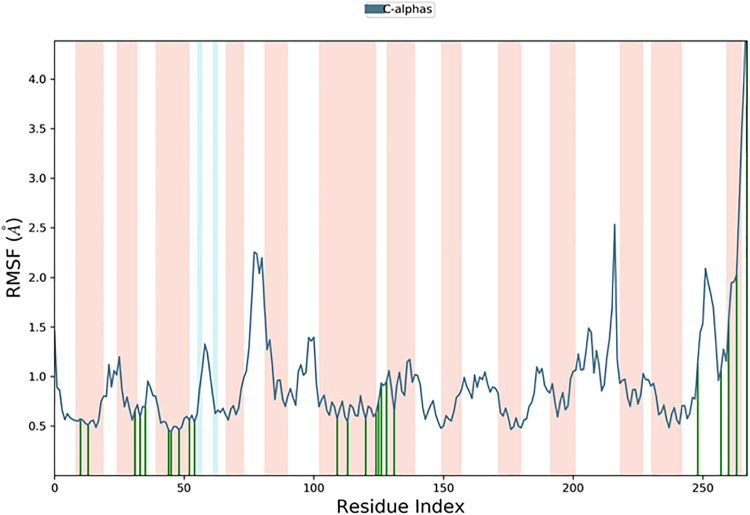
Schematic illustration of 200 ns MD Simulation graph of root-mean-square fluctuation (RMSF) of Reference ligand.

The RMSF analysis of the reference ligand (Fig 9) showed a pattern comparable to those of F2708-0061 and F3023-0929, with fluctuations around residues 30, 60, 80, 100, 200, 210, 250, and 270 reaching up to 2.5 Å. Most core regions remained stable below 1.2 Å, indicating limited conformational flexibility, while moderate fluctuations (1.2–2.0 Å) were observed around residues 40–50, 90–110, 150–170, and 240–255, likely corresponding to dynamic loop regions involved in ligand recognition. Compared with the two top ligands (Fig 8), the reference ligand exhibited low-to-moderate fluctuations with peaks around residues 75, 95, 210, and 260, whereas the top ligands showed modest fluctuations with more pronounced peaks between residues 75 and 250. Nevertheless, RMSF values remained below 3.0 Å, indicating overall protein stability and limited flexibility favorable for ligand binding. The results further suggest that both ligands preserved the rigidity of key secondary structural elements, including α-helices and β-strands, thereby supporting stable protein–ligand interactions

#### 3.6.3. Radius of gyration and surface accessibility.

**Fig 10:**
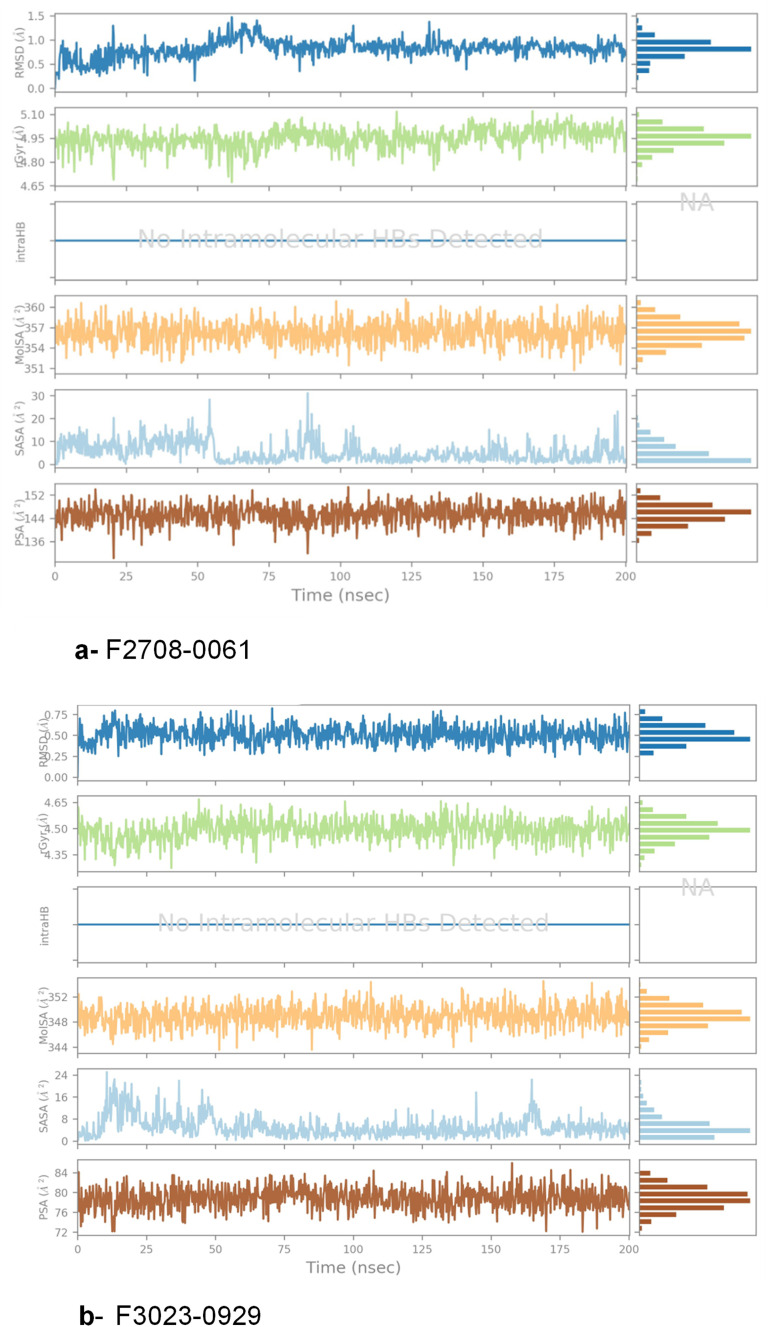
Schematic illustration of 200 ns MD Simulation graph of the radius of gyration, solvent accessible surface area (SASA), polar surface area (PSA) and molecular surface area (MolSA) of compound F2708-0061 and F3023-0929.

**Fig 11:**
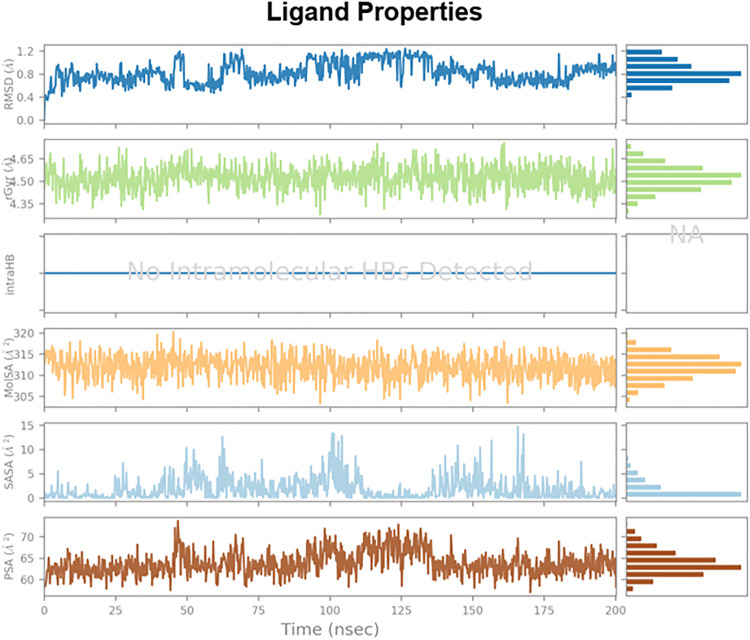
Schematic illustration of 200 ns MD Simulation graph of the radius of gyration, solvent accessible surface area (SASA), polar surface area (PSA) and molecular surface area (MolSA) of the reference compound.

Structural dynamics and surface property analyses, including solvent accessible surface area (SASA), polar surface area (PSA), molecular surface area (MolSA) and radius of gyration (Rg), were used to evaluate ligand conformational behaviour during the MD simulation. Radius of gyration (Rg) provides information regarding the compactness and folding stability of the protein-ligand complex. The Rg profiles for both ligands remained relatively stable throughout the simulation, indicating maintenance of overall structural compactness (Fig 10). However, F2708-0061 displayed slightly higher Rg values (4.9–5.0 Å) compared to F3023-0929 (4.4–4.6 Å). This suggests that F2708-0061 adopts a comparatively more expanded conformation, whereas F3023-0929 maintains a more compact molecular arrangement. The reference ligand maintained a relatively stable Rg around 4.4–4.6 Å throughout the simulation, indicating a compact and conformationally stable structure (Fig 11). This behaviour is very similar to F3023-0929, whose Rg values also remained within the same range with relatively small fluctuations. Therefore, in terms of compactness, the reference ligand and F3023-0929 appear more structurally compact than F2708-0061.

SASA analysis further demonstrated stable solvent exposure patterns for both compounds. Moderate SASA values indicate that the complexes maintained proper folding and solvent accessibility without excessive exposure of hydrophobic regions. Excessive increases in SASA typically indicate protein unfolding or instability, however neither complex showed this behaviour (Fig 10). Although F3023-0929 showed higher SASA values during the early stages of the simulation (<50 ns), both ligands exhibit comparable SASA profiles after equilibration, indicating similar solvent exposure and binding pocket accommodation in the later simulation period. The reference ligand showed comparatively lower SASA values, mostly below 5 Å² with occasional spikes. Overall, the SASA profiles indicate that both F2708-0061 and F3023-0929 attained comparable solvent-accessible conformations after equilibration and exhibit behavior similar to the reference ligand, suggesting stable accommodation within the binding pocket throughout the latter stages of the MD simulation.

The reference ligand showed the lowest PSA values (60–70 Å²), indicating a comparatively lower polar character. F3023-0929 exhibited moderately higher PSA values (76–82 Å²), while F2708-0061 showed substantially higher PSA values (140–150 Å²). The elevated PSA of F2708-0061 suggests a stronger capacity for polar and hydrogen-bonding interactions relative to both the reference ligand and F3023-0929. The reference ligand’s lower PSA may favour hydrophobic interactions within the receptor binding pocket. The differences notwithstanding, the PSA profiles remained relatively stable during the simulation period. Stable PSA values indicate consistent interactions between the ligand and surrounding residues, which contributes significantly to binding affinity and specificity. The heteroatoms present in both compounds likely contributed to sustained polar contacts and hydrogen bonding interactions throughout the simulation.

The MolSA profiles of both ligands and the reference ligand remained relatively stable throughout the 200 ns simulation, indicating preservation of their overall molecular surface characteristics during complex formation. F2708-0061 exhibited the highest MolSA values (354–360 Å²), indicating greater molecular surface exposure and comparatively lower compactness. This observation is consistent with the Rg profile, which suggested that F2708-0061 adopts a more expanded conformation during the simulation. F3023-0929 and the reference ligand exhibited MolSA values of about 344–352 Å² and 305–318 Å² respectively. Despite the differences in values, the MolSA trajectories observed for F2708-0061, F3023-0929, and the reference ligand display only minor fluctuations over the simulation period, suggesting that their molecular surface geometries remain structurally stable during ligand–protein interactions.

Collectively, the RMSD, RMSF, Rg, SASA, PSA, and MolSA analyses demonstrate that both F2708-0061 and F3023-0929 formed stable complexes with the target protein throughout the 200 ns MD simulation, exhibiting binding behaviours comparable to the reference ligand. F3023-0929 displayed structural compactness and conformational stability closely resembling that of the reference ligand, while F2708-0061 exhibited a slightly more expanded conformation characterized by higher polar and molecular surface areas, suggesting an enhanced capacity for polar interactions within the binding pocket. Despite these differences, the relatively stable trajectory profiles observed for all three ligands indicate sustained ligand accommodation and favourable binding stability over the course of the simulation.

#### 3.6.4. Protein-ligand Contacts.

**Fig 12:**
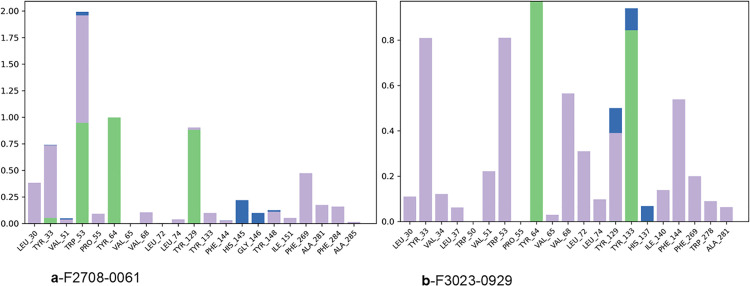
Schematic illustration of 200 ns MD Simulation graph of protein-ligand contacts of compounds F2708-0061 (a) and F3023-0929 (b).

Protein- ligand contacts provide detailed insights into the interactions between a ligand and its target protein during MD simulations. In this study water bridges, hydrophobic interactions, and hydrogen bonds (H-bonds) were used to quantify these interactions [47]. Hydrogen bonds were defined by a donor–acceptor distance ≤ 3.5 Å and a donor–hydrogen–acceptor angle ≥ 120°. Hydrophobic contacts were identified when nonpolar atoms were within 4.5 Å, while water bridges were assigned when a water molecule simultaneously formed hydrogen bonds with both the ligand and protein residues within the same geometric criteria. Various amino acid residues engage in these interactions, which is necessary in determining and understanding the binding mechanism and stability of the protein-ligand complex [48]. In a protein-ligand contact plot from an MD simulation, the amino acid residues are normally shown by the x-axis, while the y-axis shows the fraction of time the interaction is maintained during the simulation. Color bars are displayed in Fig 12; light purple indicates hydrophobic contacts, green indicates hydrogen bond interactions, and blue indicates water bridges.

The MD analysis revealed that several residues play critical roles in ligand stabilization. As shown in Fig 12, for compound F2708-0061, residues TYR33, TRP53, TYR64, and TYR129 exhibited high occupancy values (>50% of simulation time), indicating persistent interactions. TYR33 and TRP53 participated in π–π stacking and hydrophobic interactions, whereas TYR64 contributed through hydrogen bonding, collectively maintaining the ligand within the binding pocket. Other residues such as LEU30 and PHE269 showed moderate occupancy (30–50%), providing additional stabilization via hydrogen bonds and hydrophobic contacts. This implies that the ligand is stabilized by the amino acid residues across a number of nodes. Residues, PRO55, VAL68, TYR133, LEU74, ALA281, and PHE284 contributed mainly through hydrophobic interactions with lower occupancy (<30%), reflecting transient contacts important for conformational adaptability. As seen in Fig 12, the water-mediated interactions for VAL51, HIS145, GLY146, TYR 148, HIS 145, and GLY 146 suggest that the binding area is flexible or partially exposed to solvent. The combination of high-occupancy interactions and persistent hydrophobic contacts highlights key anchoring points that directly contribute to ligand binding strength and stability.

Ligand F3023-0929 formed a stable interaction network within the binding pocket during the MD simulation, with residues LEU30, TRP53, TYR64, VAL68, TYR129, TYR133, and PHE144 showing the highest interaction occupancies. Moderate occupancies (~20%) were observed for VAL51, LEU72, PHE269, and ILE140, indicating supportive roles in ligand stabilization. PHE144 contributed through π–π stacking and hydrophobic interactions, while TYR64 and TYR133 were involved in hydrogen bonding, highlighting the importance of both hydrophobic and polar contacts in maintaining ligand stability. Additionally, water-mediated interactions involving TYR129, TYR133, and HIS139 further stabilized the complex through indirect hydrogen-bonding networks. Collectively, these interactions indicate that F3023-0929 is favourably accommodated within the active site through a combination of hydrophobic, aromatic, hydrogen bonding, and water-bridged interactions, thereby supporting the stable binding behaviour observed during the simulation trajectory.

**Fig 13:**
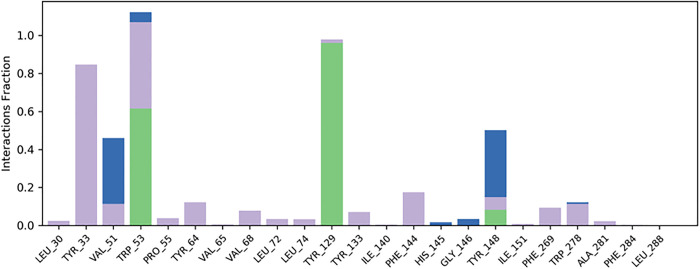
Schematic illustration of 200 ns MD Simulation graph of the protein-ligand contacts of the reference ligand.

Fig 12 shows the protein-ligand contacts for the reference ligand, dominant interactions with amino acid residues like TYR 53, TRP53, and TYR 129 showed high interaction fractions, with fraction times exceeding 0.5. These residues are essential for both specificity and ligand anchoring. Other significant residues PR O55, TYR 64, VAL 68, LEU72, LEU 74, TYR 133, PHE 144, PHE 269, and ALA 281 show notable hydrophobic interactions, suggesting their involvement in non-polar stabilization. VAL 51, HIS 145, GLY 146, TYR 148 and TRP 278 contribute clear water-bridged interactions suggesting that parts of the ligand or binding pocket may be partially solvent-exposed or flexible. In Fig 13, the majority of residues have interaction percentages that are less than 0.4, suggesting that only a small number of important residues play a significant role in binding. Additionally, no residue surpasses 1.0, indicating that no residue is consistently involved in various contact types throughout the whole simulation.

### 3.7. Larvicidal test and analysis

**Fig 14:**
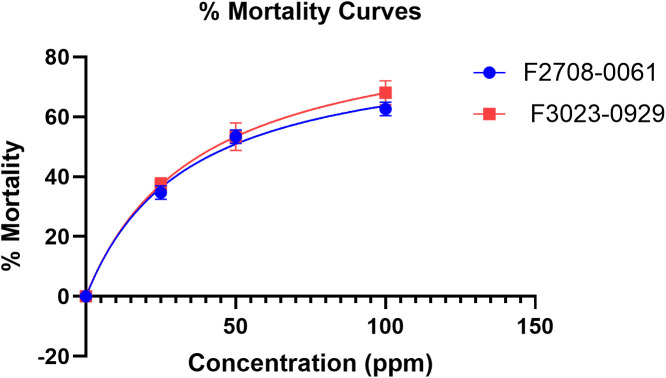
Dose dependent percentage mortality curves for compounds F3023-0929 and F2708-0061.

The predicted docking scores and binding energies of the lead compounds, F3023-0929 and F2708-0061, were validated through laboratory bioassays, with results summarized in **Table 7**. Both compounds exhibited significant dose-dependent larvicidal activity against *Anopheles arabiensis*, with 100 ppm producing the strongest effect (Fig 14). At this concentration, measured 72 hours post-exposure, F3023-0929 caused an average mortality of 68%, compared to 62.67% for F2708-0061. However, the difference was not statistically significant (ANOVA, p = 0.116), indicating comparable performance. A similar trend was observed at 50 ppm, where the larvicidal effects of the two compounds did not differ significantly (ANOVA, p = 1.00). At 25 ppm, F3023-0929 again showed a slightly higher mean mortality (37.33%) compared to F2708-0061 (34.67%), but the difference remained statistically insignificant (ANOVA, p = 0.23). Overall, F3023-0929 and F2708-0061 exhibited potent larvicidal activity with LC_50_ values of 37.75 ppm (95% CI: 25.74–55.93) and 33.49 ppm (95% CI: 23.99–46.78), respectively, indicating their potential as promising insecticidal candidates for mosquito control. Interestingly, none of the surviving larvae developed into pupae during the 14 days monitoring period, suggesting that both compounds may interfere with juvenile hormone–mediated metamorphosis, thereby disrupting normal mosquito development. While a direct comparison with established insect growth regulators could not be done, since LC₅₀ values measure larval mortality, whereas these regulators are usually assessed by inhibition of emergence (IE₅₀), well-known agents like pyriproxyfen and methoprene disrupt mosquito development at very low concentrations [49]. Likewise, the tested compounds seem to interfere with metamorphosis, as indicated by the surviving larvae’s inability to progress to the pupal stage.

**Table 7:** Mean percent larval mortality rate of *An. arabiensis* following 72h after exposure to compounds F3023-0929 and F2708-0061.

Compound	Concentration (ppm)	% Mean mortality rate ± SEM		LC_50_ (ppm)	95% CI
		24 hours	72 hours		
F3023-0929	25	20.00 ± 2.31	37.33 ± 1.33^a^	37.75	25.74-55.93
	50	45.33 ± 3.53	53.33 ± 2.67^b^		
	100	56.00 ± 6.11	68.00 ± 2.31^c^		
F2708-0061	25	16.00 ± 2.31	34.67 ± 1.33^a^	33.49	23.99-46.78
	50	37.33 ± 5.81	53.33 ± 1.33^b^		
	100	36.00 ± 2.31	62.67 ± 1.33^c^		
Negative control	0.00	0.00	0.00		

Mean values followed by the same letters (a, b or c) are not statistically significantly different (one way ANOVA Tukey post-hoc test; p-value < 0.05)

## Conclusion

The mosquito juvenile hormone binding protein (MJHBP) is a key regulator of development, metamorphosis, and reproduction in Anopheles mosquitoes. Disrupting this hormone signaling impairs normal growth and survival, making MJHBP a potential avenue for developing selective and effective insecticides against malaria vectors. This study utilized integrate *in silico* techniques including molecular docking, molecular mechanics generalized Born surface area (MM-GBSA), insecticide-likeness assessment, toxicity and environmental hazard predictions, Density Functional Theory (DFT) studies and molecular dynamics (MD) simulations and larvicidal test for discovery of novel inhibitors targeting MJHBP. We identified fifteen (15) promising inhibitors of MJHBP exhibiting docking scores ranging from −11.878 kcal/mol and −11.206 kcal/mol, and MMGBSA scores ranging between –49.84 kcal/mol to –89.51 kcal/mol). The DFT results of the analysed compounds indicated that the top candidates, based on docking scores and binding energies, possessed lower HOMO-LUMO energy gap values, 1.52 eV for F3023-0929 and 2.36 eV for F2708-0061, compared to the co-crystallized ligand, which had a gap of 3.43 eV. MD simulation analysis demonstrated that both F2708-0061 and F3023-0929 formed stable complexes with the target protein throughout the 200 ns MD simulation, exhibiting binding behaviours comparable to the reference ligand. All the top compounds displayed significant insecticidal potential with minimal environmental hazards. F3023-0929 and F2708-0061 exhibited potent larvicidal activity with IC_50_ values of 37.75 ppm and 33.49 ppm, respectively in a larvicidal in vivo test, supporting their potential as promising insecticidal candidates for mosquito control.

## Supporting information

S1 File Supplementary figure and table. Contains the crystal structure of mosquito juvenile hormone-binding protein (PDB ID: 5V13) and a list of the titles and chemical names of compounds obtained from the Agrochemical Insecticide Screening Library.
(DOCX)
