# Supplementary material for: Integrated in silico and in vivo larvicidal evaluation of compounds targeting juvenile hormone for malaria vector control
Source: PLoS One. 2026 Jun 26;21(6):e0352147. doi: 10.1371/journal.pone.0352147 (PMC13308820; doi:10.1371/journal.pone.0352147)
Supplement: S1 File — Contains the crystal structure of mosquito juvenile hormone-binding protein (PDB ID: 5V13) and a list of the titles and chemical names of compounds obtained from the Agrochemical Insecticide Screening Library. (DOCX) [file pone.0352147.s001.docx]

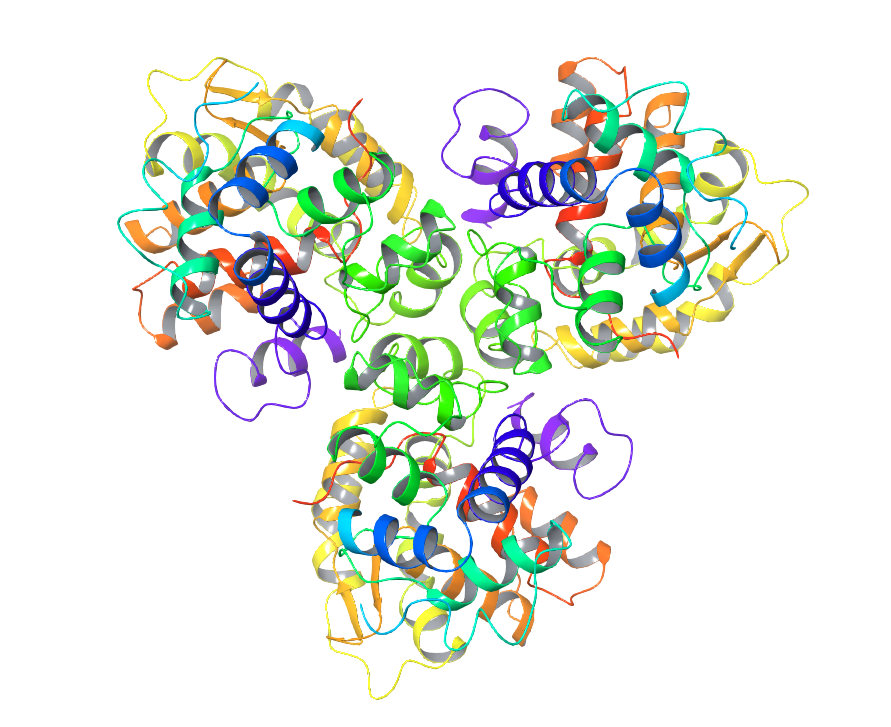


Supplementary Fig. 1: Crystal structure of mosquito juvenile hormone-binding protein (PDB ID: 5V13)

Table 1: The titles and chemical names of the compounds obtained from the Agrochemical Insecticide Screening Library

| Title | Chemical_Name |
| --- | --- |
| F6845-0033 | 5'-(propan-2-yl)-8-azaspiro[bicyclo[3.2.1]octane-3,2'-[1,5]oxazinan]-6'-one hydrochloride |
| F6482-2348 | 1,3-dimethyl-N-(octahydro-1,4-benzodioxin-6-yl)-1H-pyrazole-5-carboxamide |
| F3395-1848 | 1-[2-(pyridin-3-yl)piperidin-1-yl]ethan-1-one |
| F6546-1184 | 7-[(pyridin-3-yl)methyl]-7-azabicyclo[2.2.1]heptane |
| F6541-1260 | 3-[(pyrrolidin-1-yl)methyl]pyridine dihydrochloride |
| F2644-0496 | 8-amino-1,3-dimethyl-7-(3-methylbutyl)-2,3,6,7-tetrahydro-1H-purine-2,6-dione |
| F6617-2271 | 2-[3-(propan-2-yl)-1,2,4-oxadiazol-5-yl]pyridine |
| F6617-2272 | 4-[3-(propan-2-yl)-1,2,4-oxadiazol-5-yl]pyridine |
| F6561-4597 | 1-{3-methylidene-8-azabicyclo[3.2.1]octan-8-yl}pent-4-en-1-one |
| F6617-2251 | 2-(3-cyclopropyl-1,2,4-oxadiazol-5-yl)pyridine |
| F6617-2260 | 3-(3-cyclopropyl-1,2,4-oxadiazol-5-yl)pyridine |
| F6619-8932 | 4-(phenoxymethyl)oxane |
| F6617-2252 | 4-(3-cyclopropyl-1,2,4-oxadiazol-5-yl)pyridine |
| F6617-2213 | 2-methyl-5-(3-methyl-1,2,4-oxadiazol-5-yl)pyridine |
| F8889-4371 | 4-(cyclohexyloxy)phenol |
| F6473-3523 | 1-[1-(4-methylthiophene-2-carbonyl)azetidin-3-yl]pyrrolidine-2,5-dione |
| F6617-5673 | 2-chloro-5-(3-methyl-1,2,4-oxadiazol-5-yl)pyridine |
| F6822-6691 | N-cyclopentyl-4-(trifluoromethyl)pyridine-3-carboxamide |
| F6822-6716 | 1-methyl-4-[4-(trifluoromethyl)pyridine-3-carbonyl]piperazine |
| F6617-2225 | 3-fluoro-5-(3-methyl-1,2,4-oxadiazol-5-yl)pyridine |
| F1906-0442 | 4-phenoxybutan-1-amine |
| F6617-2496 | 4-(5-methyl-1,2,4-oxadiazol-3-yl)pyridine |
| F6525-0009 | N-({2-methyl-2H,4H,5H,6H-cyclopenta[c]pyrazol-3-yl}methyl)-2-(trifluoromethyl)benzamide |
| F6477-5333 | 1-({2-[(pyridin-2-yl)amino]-1,3-thiazol-4-yl}methyl)pyrrolidine-2,5-dione hydrochloride |
| F6617-3584 | 3-[3-(pyridin-3-yl)-1,2,4-oxadiazol-5-yl]propanoic acid |
| F8880-8338 | N-[1-(pyridin-3-yl)ethyl]cyclopropanamine |
| F0634-0003 | 7-ethyl-1,3-dimethyl-8-[(piperidin-1-yl)methyl]-2,3,6,7-tetrahydro-1H-purine-2,6-dione |
| F2158-0913 | 3-(piperidin-2-yl)pyridine |
| F6511-9314 | 2-[1-(3-bromophenyl)-1H-1,2,3-triazol-4-yl]ethan-1-ol |
| F6511-9321 | 2-[1-(4-bromophenyl)-1H-1,2,3-triazol-4-yl]ethan-1-ol |
| F0481-0378 | 3-(piperidin-2-yl)pyridine hydrochloride |
| F6477-5328 | 1-({2-[(3-methylpyridin-2-yl)amino]-1,3-thiazol-4-yl}methyl)pyrrolidine-2,5-dione hydrochloride |
| F6481-0809 | 1-{2-[7-(2-chlorophenyl)-1,4-thiazepan-4-yl]-2-oxoethyl}pyrrolidine-2,5-dione |
| F6525-0231 | 3,5-dimethyl-N-({2-methyl-2H,4H,5H,6H-cyclopenta[c]pyrazol-3-yl}methyl)benzamide |
| F3259-0263 | 1,3-dimethyl-7-(2-methylpropyl)-2,3,6,7-tetrahydro-1H-purine-2,6-dione |
| F6511-9308 | 2-{1-[2-chloro-5-(trifluoromethyl)phenyl]-1H-1,2,3-triazol-4-yl}ethan-1-ol |
| F6572-1379 | 2-[1-(2-chlorophenyl)-1H-1,2,3-triazol-4-yl]ethan-1-ol |
| F6525-0151 | 2-(3-methoxyphenyl)-N-({2-methyl-2H,4H,5H,6H-cyclopenta[c]pyrazol-3-yl}methyl)acetamide |
| F8889-6491 | 4-(ethoxycarbonyl)benzoic acid |
| F6477-5330 | 1-({2-[(pyridin-3-yl)amino]-1,3-thiazol-4-yl}methyl)pyrrolidine-2,5-dione hydrochloride |
| F6477-5326 | 1-({2-[(4-methylpyridin-3-yl)amino]-1,3-thiazol-4-yl}methyl)pyrrolidine-2,5-dione hydrochloride |
| F3259-0270 | 7-[(2E)-but-2-en-1-yl]-1,3-dimethyl-2,3,6,7-tetrahydro-1H-purine-2,6-dione |
| F6451-0590 | 2-(2,5-dioxopyrrolidin-1-yl)-N-[2-methoxy-2-(5-methylthiophen-2-yl)ethyl]acetamide |
| F6440-2748 | N-[(1-cyclopentyl-4,5,6,7-tetrahydro-1H-indazol-3-yl)methyl]-2-(2,5-dioxopyrrolidin-1-yl)acetamide |
| F6359-1029 | 1-(2-{4-[(3-chloropyridin-2-yl)oxy]piperidin-1-yl}-2-oxoethyl)pyrrolidine-2,5-dione |
| F6473-3588 | 1-{1-[2-(trifluoromethyl)benzoyl]azetidin-3-yl}pyrrolidine-2,5-dione |
| F2168-0005 | 2-(methylsulfanyl)pyridine-3-carboxylic acid |
| F6525-0198 | N-({2-methyl-2H,4H,5H,6H-cyclopenta[c]pyrazol-3-yl}methyl)-2-(trifluoromethoxy)benzamide |
| F6473-3564 | 1-[1-(2-chloro-4-fluorobenzoyl)azetidin-3-yl]pyrrolidine-2,5-dione |
| F6374-1422 | 1-[2-(4-{[(1-methyl-1H-imidazol-2-yl)sulfanyl]methyl}piperidin-1-yl)-2-oxoethyl]pyrrolidine-2,5-dione |
| F3260-0883 | 1-ethyl-3,7-dimethyl-8-(pyrrolidin-1-yl)-2,3,6,7-tetrahydro-1H-purine-2,6-dione |
| F6617-2074 | 4-cyclopropyl-1-(4-methylphenyl)-1H-1,2,3-triazole |
| F6617-2428 | 4-cyclopropyl-1-phenyl-1H-1,2,3-triazole |
| F6473-3650 | 1-{1-[3-(2-bromophenyl)propanoyl]azetidin-3-yl}pyrrolidine-2,5-dione |
| F6473-3772 | 1-[1-(1H-indole-3-carbonyl)azetidin-3-yl]pyrrolidine-2,5-dione |
| F6473-3536 | 1-{1-[2-(4-fluorophenyl)acetyl]azetidin-3-yl}pyrrolidine-2,5-dione |
| F6451-1112 | 2-(2,5-dioxopyrrolidin-1-yl)-N-[2-(3-fluorophenyl)-2-methoxyethyl]acetamide |
| F6359-5407 | 1-(2-{4-[(6-methylpyridin-2-yl)oxy]piperidin-1-yl}-2-oxoethyl)pyrrolidine-2,5-dione |
| F6473-3845 | N-(3,4-dichlorophenyl)-3-(2,5-dioxopyrrolidin-1-yl)azetidine-1-carboxamide |
| F6617-2177 | 1-phenyl-4-(propan-2-yl)-1H-1,2,3-triazole |
| F6473-3893 | 1-[1-(cyclohexanesulfonyl)azetidin-3-yl]pyrrolidine-2,5-dione |
| F6473-3555 | 1-{1-[2-(2-methylphenoxy)acetyl]azetidin-3-yl}pyrrolidine-2,5-dione |
| F6473-3854 | N-(4-tert-butylphenyl)-3-(2,5-dioxopyrrolidin-1-yl)azetidine-1-carboxamide |
| F2477-0096 | 1,3-dimethyl-8-(methylamino)-7-(2-methylpropyl)-2,3,6,7-tetrahydro-1H-purine-2,6-dione |
| F9994-5206 | 4-(3-methylphenyl)-4-oxobutanoic acid |
| F6184-0072 | 1-{2-oxo-2-[4-(thiophen-2-yl)piperidin-1-yl]ethyl}pyrrolidine-2,5-dione |
| F6477-5320 | 1-({2-[(2-fluorophenyl)amino]-1,3-thiazol-4-yl}methyl)pyrrolidine-2,5-dione hydrochloride |
| F6473-3532 | 1-[1-(2-phenoxyacetyl)azetidin-3-yl]pyrrolidine-2,5-dione |
| F6473-3558 | 1-{1-[2-(2-fluorophenoxy)acetyl]azetidin-3-yl}pyrrolidine-2,5-dione |
| F6525-0002 | 4-(diethylamino)-N-({2-methyl-2H,4H,5H,6H-cyclopenta[c]pyrazol-3-yl}methyl)benzamide |
| F6452-0068 | 2-(2,5-dioxopyrrolidin-1-yl)-N-[2-(2-fluorophenyl)-2-methoxypropyl]acetamide |
| F2477-0097 | 8-(dimethylamino)-1,3-dimethyl-7-(2-methylpropyl)-2,3,6,7-tetrahydro-1H-purine-2,6-dione |
| F6477-5337 | 1-{[2-(benzylamino)-1,3-thiazol-4-yl]methyl}pyrrolidine-2,5-dione hydrochloride |
| F6619-8934 | 3-(phenoxymethyl)oxolane |
| F1909-1624 | [3-(2-methoxyphenoxy)propyl](methyl)amine hydrochloride |
| F6477-5322 | 1-({2-[(3,4-difluorophenyl)amino]-1,3-thiazol-4-yl}methyl)pyrrolidine-2,5-dione hydrochloride |
| F5577-0010 | 6-fluoro-1-methyl-4-oxo-7-(piperidin-1-yl)-1,4-dihydroquinoline-3-carboxylic acid |
| F6473-3602 | 1-{1-[2-(4-chlorophenoxy)-2-methylpropanoyl]azetidin-3-yl}pyrrolidine-2,5-dione |
| F6374-0166 | 1-(2-oxo-2-{4-[(pyridin-2-ylsulfanyl)methyl]piperidin-1-yl}ethyl)pyrrolidine-2,5-dione |
| F6619-8931 | 3-(phenoxymethyl)oxane |
| F6473-3572 | 1-{1-[2-(4-ethoxyphenyl)acetyl]azetidin-3-yl}pyrrolidine-2,5-dione |
| F0001-1304 | 3,5-dimethoxyphenol |
| F2147-1619 | 4-(1H-indol-3-yl)pyrrolidin-2-one |
| F6442-1092 | 1-{2-[3-(cyclohexanesulfonyl)azetidin-1-yl]-2-oxoethyl}pyrrolidine-2,5-dione |
| F6477-5331 | 1-({2-[(4-methylphenyl)amino]-1,3-thiazol-4-yl}methyl)pyrrolidine-2,5-dione hydrochloride |
| F2189-0857 | 2-amino-1-(4-ethylphenyl)ethan-1-ol |
| F5577-0021 | 6-fluoro-1-methyl-7-(morpholin-4-yl)-4-oxo-1,4-dihydroquinoline-3-carboxylic acid |
| F6473-3573 | 1-[1-(adamantane-1-carbonyl)azetidin-3-yl]pyrrolidine-2,5-dione |
| F5822-0970 | 1-[2-oxo-2-(4-{[2-(propan-2-yl)-1H-imidazol-1-yl]methyl}piperidin-1-yl)ethyl]pyrrolidine-2,5-dione |
| F6359-5009 | 1-(2-{4-[(3-bromopyridin-2-yl)oxy]piperidin-1-yl}-2-oxoethyl)pyrrolidine-2,5-dione |
| F6473-3860 | N-[(4-chlorophenyl)methyl]-3-(2,5-dioxopyrrolidin-1-yl)azetidine-1-carboxamide |
| F3325-0151 | 3-(4-phenylpiperazin-1-yl)-1-propylpyrrolidine-2,5-dione |
| F6477-5317 | 1-({2-[(3-fluoro-4-methoxyphenyl)amino]-1,3-thiazol-4-yl}methyl)pyrrolidine-2,5-dione hydrochloride |
| F6477-5325 | 1-({2-[(4-fluoro-3-methoxyphenyl)amino]-1,3-thiazol-4-yl}methyl)pyrrolidine-2,5-dione hydrochloride |
| F5103-0371 | 2-(2,5-dioxopyrrolidin-1-yl)-N-[2-(thiophen-3-yl)ethyl]acetamide |
| F9995-0937 | 3-(4-propoxyphenyl)propanoic acid |
| F6477-5318 | 1-({2-[(2,4-difluorophenyl)amino]-1,3-thiazol-4-yl}methyl)pyrrolidine-2,5-dione hydrochloride |
| F6477-5321 | 1-({2-[(2,5-difluorophenyl)amino]-1,3-thiazol-4-yl}methyl)pyrrolidine-2,5-dione hydrochloride |
| F6477-5324 | 1-({2-[(4-methoxy-2-methylphenyl)amino]-1,3-thiazol-4-yl}methyl)pyrrolidine-2,5-dione hydrochloride |
| F6477-5327 | 1-({2-[(2,6-difluorophenyl)amino]-1,3-thiazol-4-yl}methyl)pyrrolidine-2,5-dione hydrochloride |
| F6359-0631 | 1-[2-oxo-2-(4-{[5-(trifluoromethyl)pyridin-2-yl]oxy}piperidin-1-yl)ethyl]pyrrolidine-2,5-dione |
| F9994-5470 | 3-(2-aminopropyl)phenol hydrobromide |
| F6663-4248 | 1-cyclobutyl-4-(3-fluoro-4-methylbenzoyl)piperazine |
| F5831-9803 | 2-(2,5-dioxopyrrolidin-1-yl)-N-{[1-(thiophen-2-yl)cyclopropyl]methyl}acetamide |
| F2147-0872 | 4-(2-methoxyethoxy)phenol |
| F6188-0566 | 2-(2,5-dioxopyrrolidin-1-yl)-N-{[4-(phenylsulfanyl)oxan-4-yl]methyl}acetamide |
| F2477-0098 | 8-(ethylamino)-1,3-dimethyl-7-(2-methylpropyl)-2,3,6,7-tetrahydro-1H-purine-2,6-dione |
| F3260-0381 | 7-ethyl-1,3-dimethyl-8-[(2-methylpropyl)amino]-2,3,6,7-tetrahydro-1H-purine-2,6-dione |
| F6414-1061 | 2-(2,5-dioxopyrrolidin-1-yl)-N-[(2-hydroxy-1,2,3,4-tetrahydronaphthalen-2-yl)methyl]acetamide |
| F6617-2447 | N-methyl-1H-indole-3-carboxamide |
| F6110-1919 | 1-{[1-(2,6-difluorobenzoyl)piperidin-4-yl]methyl}-2,4-dimethylpiperazine; bis(formic acid) |
| F9995-0149 | 2-{[(benzyloxy)carbonyl]amino}-3,3-dimethylbutanoic acid |
| F6473-3542 | 1-[1-(1-benzofuran-2-carbonyl)azetidin-3-yl]pyrrolidine-2,5-dione |
| F6110-1920 | 1-{[1-(2,4-difluorobenzoyl)piperidin-4-yl]methyl}-2,4-dimethylpiperazine |
| F6451-1634 | 2-(2,5-dioxopyrrolidin-1-yl)-N-[2-methoxy-2-(2-methylphenyl)ethyl]acetamide |
| F6110-1885 | 1-{[1-(2-fluorobenzoyl)piperidin-4-yl]methyl}-2,4-dimethylpiperazine; bis(formic acid) |
| F8887-5762 | 5-bromo-2,3-dihydro-1H-indol-2-one |
| F2124-0810 | 5,6,7,8-tetrahydronaphthalene-2-carboxylic acid |
| F2145-0647 | 3-bromo-5-(3-methyl-1,2,4-oxadiazol-5-yl)pyridine |
| F6477-5319 | 1-({2-[(2,4-dimethoxyphenyl)amino]-1,3-thiazol-4-yl}methyl)pyrrolidine-2,5-dione hydrochloride |
| F6471-5845 | N-(3,4-dichlorophenyl)-4-(1H-1,2,3-triazol-1-yl)piperidine-1-carboxamide |
| F6473-3901 | 1-[1-(3,4-dimethylbenzenesulfonyl)azetidin-3-yl]pyrrolidine-2,5-dione |
| F3260-0380 | 8-(butylamino)-7-ethyl-1,3-dimethyl-2,3,6,7-tetrahydro-1H-purine-2,6-dione |
| F6473-3909 | 1-[1-(4-fluoro-2-methylbenzenesulfonyl)azetidin-3-yl]pyrrolidine-2,5-dione |
| F6473-3910 | 1-[1-(4-fluoro-3-methylbenzenesulfonyl)azetidin-3-yl]pyrrolidine-2,5-dione |
| F2189-0836 | 2-amino-1-(4-methylphenyl)ethan-1-ol |
| F6473-3858 | N-(4-butoxyphenyl)-3-(2,5-dioxopyrrolidin-1-yl)azetidine-1-carboxamide |
| F6414-2055 | 2-(2,5-dioxopyrrolidin-1-yl)-N-{2-hydroxy-2-[4-(methylsulfanyl)phenyl]ethyl}acetamide |
| F6439-5046 | 2-(2,5-dioxopyrrolidin-1-yl)-N-{2-[3-(thiophen-2-yl)-1H-pyrazol-1-yl]ethyl}acetamide |
| F6440-2354 | 2-methoxy-N-[(1-methyl-4,5,6,7-tetrahydro-1H-indazol-3-yl)methyl]benzamide |
| F6451-2156 | 2-(2,5-dioxopyrrolidin-1-yl)-N-[2-methoxy-2-(3-methoxyphenyl)ethyl]acetamide |
| F6473-3623 | 1-{1-[4-(thiophen-3-yl)benzoyl]azetidin-3-yl}pyrrolidine-2,5-dione |
| F6170-0105 | 7-hydroxy-4H-chromen-4-one |
| F6473-3880 | N-[(3,4-dimethoxyphenyl)methyl]-3-(2,5-dioxopyrrolidin-1-yl)azetidine-1-carboxamide |
| F1918-0023 | 2-[(4-hydroxyphenyl)formamido]acetic acid |
| F6440-2328 | 2-methyl-N-[(1-methyl-4,5,6,7-tetrahydro-1H-indazol-3-yl)methyl]benzamide |
| F3234-0943 | 3-ethyl-1,7-dimethyl-1H,2H,3H,4H,8H-imidazo[1,2-g]purine-2,4-dione |
| F6440-2355 | 3-methoxy-N-[(1-methyl-4,5,6,7-tetrahydro-1H-indazol-3-yl)methyl]benzamide |
| F6469-0994 | 2-(2,5-dioxopyrrolidin-1-yl)-N-[2-(2-methyl-1,3-thiazol-4-yl)phenyl]acetamide |
| F5027-0654 | 1-(2-oxo-2-{2-[4-(trifluoromethyl)phenyl]morpholin-4-yl}ethyl)pyrrolidine-2,5-dione |
| F6287-0440 | 2-(2,5-dioxopyrrolidin-1-yl)-N-{2-[1-methyl-4-(thiophen-2-yl)-1H-imidazol-2-yl]ethyl}acetamide |
| F2185-0115 | 3-[3-(4-chlorophenyl)-1,2,4-oxadiazol-5-yl]propan-1-amine hydrochloride |
| F2185-0117 | 3-[3-(2-chlorophenyl)-1,2,4-oxadiazol-5-yl]propan-1-amine hydrochloride |
| F6395-3665 | 1-[2-(2-benzylmorpholin-4-yl)-2-oxoethyl]pyrrolidine-2,5-dione |
| F3260-0895 | 1-ethyl-3,7-dimethyl-8-(pentylamino)-2,3,6,7-tetrahydro-1H-purine-2,6-dione |
| F6110-1934 | 1-{4-[(2,4-dimethylpiperazin-1-yl)methyl]piperidin-1-yl}-2-(4-methylphenoxy)ethan-1-one |
| F6403-1733 | N-{2-[2-(3,5-dimethyl-1H-pyrazol-1-yl)-1,3-thiazol-4-yl]ethyl}-2-(2,5-dioxopyrrolidin-1-yl)acetamide |
| F2185-0121 | 3-[3-(4-methoxyphenyl)-1,2,4-oxadiazol-5-yl]propan-1-amine hydrochloride |
| F6401-0065 | 2-[2,6-dioxo-4-(thiophen-2-yl)piperidin-1-yl]-N-(5-methyl-1,2-oxazol-3-yl)acetamide |
| F3205-0055 | 1-methyl-3-(4-phenylpiperazin-1-yl)pyrrolidine-2,5-dione |
| F6419-0068 | 1-{2-[2-(3,5-dichlorophenyl)morpholin-4-yl]-2-oxoethyl}pyrrolidine-2,5-dione |
| F6473-3923 | 1-[1-(2-ethoxybenzenesulfonyl)azetidin-3-yl]pyrrolidine-2,5-dione |
| F6439-2365 | N-{2-[5-cyclopropyl-3-(pyridin-4-yl)-1H-pyrazol-1-yl]ethyl}-2-(2,5-dioxopyrrolidin-1-yl)acetamide |
| F6110-1933 | 1-{4-[(2,4-dimethylpiperazin-1-yl)methyl]piperidin-1-yl}-2-(3-methylphenoxy)ethan-1-one |
| F6442-2664 | 1-{2-[3-(4-chlorobenzenesulfonyl)azetidin-1-yl]-2-oxoethyl}pyrrolidine-2,5-dione |
| F1902-0099 | 7-bromo-4H-chromen-4-one |
| F0902-3042 | 3-(4-benzylpiperazin-1-yl)-1-methylpyrrolidine-2,5-dione |
| F3164-0800 | 3-methyl-7-nonyl-2,3,6,7-tetrahydro-1H-purine-2,6-dione |
| F5610-0162 | 3-[3-(azepan-1-yl)-3-oxopropyl]-2,3,4,5-tetrahydro-1H-1,4-benzodiazepine-2,5-dione |
| F5610-0202 | 3-[3-(3-methylpiperidin-1-yl)-3-oxopropyl]-2,3,4,5-tetrahydro-1H-1,4-benzodiazepine-2,5-dione |
| F6442-6647 | N-(3,4-dichlorophenyl)-4-methoxy-[1,4'-bipiperidine]-1'-carboxamide |
| F5033-0010 | N-[(1-methylpiperidin-4-yl)methyl]-2-phenoxyacetamide |
| F6439-0450 | 2-(2,5-dioxopyrrolidin-1-yl)-N-{2-[3-(pyridin-4-yl)-4,5,6,7-tetrahydro-1H-indazol-1-yl]ethyl}acetamide |
| F6473-3924 | 1-[1-(4-methoxy-3-methylbenzenesulfonyl)azetidin-3-yl]pyrrolidine-2,5-dione |
| F6440-2434 | N-[(1-methyl-4,5,6,7-tetrahydro-1H-indazol-3-yl)methyl]-3-(trifluoromethyl)benzamide |
| F5658-0167 | 1-{2-[(3-oxo-2,3-dihydro-1H-inden-5-yl)oxy]acetyl}piperidine-4-carboxamide |
| F0908-3954 | 2-(phenylsulfanyl)acetic acid |
| F6395-4299 | N-(3,4-dichlorophenyl)-1,9-dioxa-4-azaspiro[5.5]undecane-4-carboxamide |
| F6473-3906 | 1-[1-(3-methoxybenzenesulfonyl)azetidin-3-yl]pyrrolidine-2,5-dione |
| F3115-0062 | 1-(4-ethylphenyl)-3-[2-(morpholin-4-yl)cyclohex-2-en-1-yl]pyrrolidine-2,5-dione |
| F5967-0071 | 2-(2,5-dioxopyrrolidin-1-yl)-N-{2-oxo-9-oxa-1-azatricyclo[10.4.0.0^{3,8}]hexadeca-3,5,7-trien-5-yl}acetamide |
| F6473-3944 | 1-[1-(3-chloro-4-methoxybenzenesulfonyl)azetidin-3-yl]pyrrolidine-2,5-dione |
| F3229-0039 | N-(3,4-dimethylphenyl)-2-(2,5-dioxopyrrolidin-1-yl)acetamide |
| F6464-0017 | N-[4-(methylsulfanyl)-1-oxo-1-(3-phenylazepan-1-yl)butan-2-yl]acetamide |
| F6412-2278 | N-[4-(2H-1,3-benzodioxol-5-yloxy)but-2-yn-1-yl]-2-(2,5-dioxopyrrolidin-1-yl)acetamide |
| F5857-3829 | 2-(2,5-dioxopyrrolidin-1-yl)-N-[2-hydroxy-3-(4-methoxyphenyl)-2-methylpropyl]acetamide |
| F6440-2409 | 3-(4-methoxyphenyl)-N-[(1-methyl-4,5,6,7-tetrahydro-1H-indazol-3-yl)methyl]propanamide |
| F6200-3035 | 2-(2,5-dioxopyrrolidin-1-yl)-N-[2-hydroxy-2-(1-methyl-1H-indol-3-yl)ethyl]acetamide |
| F6349-1297 | 2-(2,5-dioxopyrrolidin-1-yl)-N-(4-{5H,6H,7H-pyrrolo[1,2-a]imidazol-3-yl}phenyl)acetamide |
| F3023-0916 | 3-[4-(4-fluorophenyl)piperazin-1-yl]-1-(propan-2-yl)pyrrolidine-2,5-dione |
| F3205-0008 | 1-(4-methylphenyl)-3-[2-(piperidin-1-yl)cyclohex-2-en-1-yl]pyrrolidine-2,5-dione |
| F5882-3200 | N-(3,4-dichlorophenyl)-4-(2-oxopyrrolidin-1-yl)piperidine-1-carboxamide |
| F3228-0187 | 3-(2,4-dihydroxyphenyl)propanoic acid |
| F1130-0019 | 2-[(4-fluorophenyl)formamido]propanoic acid |
| F1799-0234 | 1-(4-bromophenyl)-3-(piperidin-1-yl)pyrrolidine-2,5-dione |
| F6212-0243 | N-(5-chloro-2-hydroxyphenyl)-5H,6H,7H-pyrazolo[3,2-b][1,3]oxazine-3-carboxamide |
| F5860-4145 | methyl 3-[2-(2,5-dioxopyrrolidin-1-yl)acetamido]adamantane-1-carboxylate |
| F6211-0243 | N-(5-chloro-2-hydroxyphenyl)-5H,6H,7H-pyrazolo[3,2-b][1,3]oxazine-2-carboxamide |
| F5857-4208 | 2-(2,5-dioxopyrrolidin-1-yl)-N-(2-hydroxy-2-methyl-4-phenylbutyl)acetamide |
| F0327-0304 | 4-(2,5-dioxopyrrolidin-1-yl)-N-(6-methyl-4,5,6,7-tetrahydro-1,3-benzothiazol-2-yl)benzamide |
| F6374-1736 | 1-[2-(4-{[(4-methoxyphenyl)sulfanyl]methyl}piperidin-1-yl)-2-oxoethyl]pyrrolidine-2,5-dione |
| F3298-0060 | 3-(2,5-dioxopyrrolidin-1-yl)-N-[3-(trifluoromethyl)phenyl]propanamide |
| F3325-0016 | 1-[(4-methylphenyl)methyl]-3-(piperidin-1-yl)pyrrolidine-2,5-dione |
| F3023-0921 | 3-[4-(4-fluorophenyl)piperazin-1-yl]-1-(2-methoxyethyl)pyrrolidine-2,5-dione |
| F6473-3989 | 1-[1-(quinoline-8-sulfonyl)azetidin-3-yl]pyrrolidine-2,5-dione |
| F3393-0524 | 3-(3-phenyl-1,2,4-oxadiazol-5-yl)propanoic acid |
| F3157-0003 | 3-(4-methoxyphenyl)propanoic acid |
| F0347-2366 | 2-[(2-fluorophenyl)formamido]acetic acid |
| F2119-0005 | 3-(3-hydroxyphenyl)propanoic acid |
| F2182-0140 | 2-[(4-methylphenyl)sulfanyl]acetic acid |
| F6245-4960 | 1-{2-[3-(1-methyl-5-oxo-4-phenyl-4,5-dihydro-1H-1,2,4-triazol-3-yl)piperidin-1-yl]-2-oxoethyl}pyrrolidine-2,5-dione |
| F6782-8295 | 2-[(4-methoxyphenyl)methyl]-1H,2H,7H,8H,9H,10H-pyrazino[1,2-b]indazol-1-one |
| F6782-8294 | 2-[(3-methoxyphenyl)methyl]-1H,2H,7H,8H,9H,10H-pyrazino[1,2-b]indazol-1-one |
| F6790-0118 | 4-methyl-N-phenylpyrimidin-2-amine |
| F3325-0115 | 3-(4-benzylpiperazin-1-yl)-1-(propan-2-yl)pyrrolidine-2,5-dione |
| F5658-0165 | N-(3-methylbutyl)-2-[(3-oxo-2,3-dihydro-1H-inden-5-yl)oxy]acetamide |
| F6359-0356 | 1-(2-{[4-(pyridin-2-yloxy)piperidin-1-yl]sulfonyl}ethyl)piperidine-2,6-dione |
| F1929-2096 | 1-benzoylpiperazine hydrochloride |
| F6415-1241 | 2-(2,5-dioxopyrrolidin-1-yl)-N-[(furan-3-yl)methyl]-N-[2-(thiophen-2-yl)ethyl]acetamide |
| F0346-1855 | 2,3-dihydro-1,4-benzodioxine-6-carboxylic acid |
| F2257-0292 | N-(2-oxo-2,3-dihydro-1H-indol-5-yl)acetamide |
| F3191-0224 | 4-(2-methoxyethoxy)benzoic acid |
| F6266-0370 | N-(3,4-dichlorophenyl)-3-oxa-8-azabicyclo[3.2.1]octane-8-carboxamide |
| F6441-3577 | N-(3,4-dichlorophenyl)-4-(2-methylpropanesulfonyl)piperidine-1-carboxamide |
| F5831-7582 | 2-(2,6-dioxopiperidin-1-yl)-N-[(thiophen-3-yl)methyl]ethane-1-sulfonamide |
| F1371-0197 | 1,1-dimethyl-1H,2H,3H,4H,9H-pyrido[3,4-b]indole-3-carboxylic acid |
| F6440-2510 | N-[(1-methyl-4,5,6,7-tetrahydro-1H-indazol-3-yl)methyl]-4-(trifluoromethoxy)benzamide |
| F1243-0099 | 4-bromo-N,N,3,5-tetramethyl-1H-pyrazole-1-sulfonamide |
| F6541-2777 | N-cyclopropyl-2-(1H-indol-3-yl)acetamide |
| F5940-0534 | 1-(2-{4-[(benzyloxy)methyl]piperidin-1-yl}-2-oxoethyl)pyrrolidine-2,5-dione |
| F6276-0549 | 1-{2-[3-(1,3-benzothiazol-2-yloxy)azetidin-1-yl]-2-oxoethyl}pyrrolidine-2,5-dione |
| F0914-3327 | N-methyl-1H-indole-2-carboxamide |
| F5614-0448 | 1-(2-{4-[(1-methyl-1H-1,3-benzodiazol-2-yl)methyl]piperazin-1-yl}-2-oxoethyl)pyrrolidine-2,5-dione |
| F5792-0435 | 1-{2-oxo-2-[4-(5-phenyl-6H-1,3,4-thiadiazin-2-yl)piperazin-1-yl]ethyl}pyrrolidine-2,5-dione hydrochloride |
| F3325-0107 | 3-[4-(4-fluorophenyl)piperazin-1-yl]-1-(3-methoxypropyl)pyrrolidine-2,5-dione |
| F5854-2642 | 2-(2,5-dioxopyrrolidin-1-yl)-N-[(furan-2-yl)methyl]-N-[2-(thiophen-2-yl)ethyl]acetamide |
| F0307-0311 | 2-[(3-methoxyphenyl)formamido]acetic acid |
| F1130-0072 | 2-[(4-methoxyphenyl)formamido]acetic acid |
| F2147-1049 | 3-hydroxybenzene-1,2-dicarboxylic acid |
| F5033-0277 | N-{[1-(2-methoxyethyl)piperidin-4-yl]methyl}-2-(4-methylphenoxy)acetamide |
| F3393-0004 | 1-[3-(4-methylphenyl)-1,2,4-oxadiazol-5-yl]propan-2-one |
| F6265-0370 | N-(3,4-dichlorophenyl)-3-oxa-9-azabicyclo[3.3.1]nonane-9-carboxamide |
| F6525-0205 | 3-(3-methoxyphenyl)-1-methyl-N-({2-methyl-2H,4H,5H,6H-cyclopenta[c]pyrazol-3-yl}methyl)-1H-pyrazole-5-carboxamide |
| F2111-0036 | 2-(phenylformamido)acetic acid |
| F3325-0124 | 3-(4-benzylpiperazin-1-yl)-1-(prop-2-en-1-yl)pyrrolidine-2,5-dione |
| F6374-1528 | 1-{2-[(4-{[(1-methyl-1H-imidazol-2-yl)sulfanyl]methyl}piperidin-1-yl)sulfonyl]ethyl}piperidine-2,6-dione |
| F3259-0267 | 1,3-dimethyl-7-(2-phenylethyl)-2,3,6,7-tetrahydro-1H-purine-2,6-dione |
| F6401-0058 | N-benzyl-2-[2,6-dioxo-4-(thiophen-2-yl)piperidin-1-yl]acetamide |
| F1085-0016 | 2-[(2,4-dichlorophenyl)formamido]acetic acid |
| F3316-0245 | N-[2-(4-chloro-3,5-dimethyl-1H-pyrazol-1-yl)ethyl]thiophene-2-carboxamide |
| F5032-0055 | 1-benzyl-8-hydroxy-3,7-dimethyl-2,3,6,7-tetrahydro-1H-purine-2,6-dione |
| F2135-0535 | [5-(4-methoxyphenyl)-1,2-oxazol-3-yl]methanol |
| F8889-8011 | ethyl 3-oxocyclohexane-1-carboxylate |
| F2130-0143 | 4-bromo-3-(4-fluorophenyl)-1H-pyrazole-5-carboxylic acid |
| F2609-0840 | 1-{2-[4-(6-fluoro-1,3-benzothiazol-2-yl)piperazin-1-yl]-2-oxoethyl}pyrrolidine-2,5-dione |
| F0917-0657 | 2-[(4-fluorophenyl)sulfanyl]acetic acid |
| F5033-0182 | 2-(4-methylphenoxy)-N-{[1-(propan-2-yl)piperidin-4-yl]methyl}acetamide |
| F0327-0305 | 4-(2,5-dioxopyrrolidin-1-yl)-N-(4,5,6,7-tetrahydro-1,3-benzothiazol-2-yl)benzamide |
| F2978-0030 | N-(4,5-dimethyl-1,3-thiazol-2-yl)-4-(2,5-dioxopyrrolidin-1-yl)benzamide |
| F6545-0067 | 1-(1-cyclopentanecarbonylpyrrolidin-3-yl)ethan-1-one |
| F1913-0574 | methyl 2-(oxolan-3-yl)propanoate |
| F1929-1777 | N-[2-(5-methoxy-1H-indol-3-yl)ethyl]acetamide |
| F5854-2345 | 2-(2,5-dioxopyrrolidin-1-yl)-N-[(furan-2-yl)methyl]-N-[(thiophen-3-yl)methyl]acetamide |
| F3259-0257 | 7-[(2-chlorophenyl)methyl]-1,3-dimethyl-2,3,6,7-tetrahydro-1H-purine-2,6-dione |
| F1345-0583 | 1-(piperidin-1-yl)-3-[2-(prop-2-en-1-yl)phenoxy]propan-2-ol; benzoic acid |
| F2135-1140 | 4-chloro-1-phenyl-3-(trifluoromethyl)-1H-pyrazol-5-ol |
| F6401-0070 | 4-(thiophen-2-yl)-1-{[3-(trifluoromethyl)phenyl]methyl}piperidine-2,6-dione |
| F2609-2014 | 1-{2-[4-(4-fluoro-1,3-benzothiazol-2-yl)piperazin-1-yl]-2-oxoethyl}pyrrolidine-2,5-dione |
| F3325-0080 | tert-butyl 4-[1-(4-methylphenyl)-2,5-dioxopyrrolidin-3-yl]piperazine-1-carboxylate |
| F6401-0077 | 1-[2-(4-methoxyphenyl)-2-oxoethyl]-4-(thiophen-2-yl)piperidine-2,6-dione |
| F2148-2605 | 2-(2H-1,3-benzodioxol-5-yl)-2-(morpholin-4-yl)ethan-1-amine |
| F6660-6276 | 4-(3-phenyl-1,2,4-oxadiazol-5-yl)pyridine |
| F5729-1016 | 1-(2-{4-[(2-methyl-1H-1,3-benzodiazol-1-yl)methyl]piperidin-1-yl}-2-oxoethyl)pyrrolidine-2,5-dione; oxalic acid |
| F1926-0026 | 7-bromopyrido[2,3-b]pyrazine-6-carboxylic acid |
| F0396-0210 | N-{2-tert-butyl-2H,4H,6H-thieno[3,4-c]pyrazol-3-yl}-4-(2,5-dioxopyrrolidin-1-yl)benzamide |
| F2468-0237 | 4-(2,5-dioxopyrrolidin-1-yl)-N-{4-[(methylsulfanyl)methyl]-1,3-thiazol-2-yl}benzamide |
| F6400-1510 | N-(3,4-dichlorophenyl)-3-[(morpholin-4-yl)methyl]-1,4-thiazepane-4-carboxamide |
| F6401-0076 | 1-[2-(4-chlorophenyl)-2-oxoethyl]-4-(thiophen-2-yl)piperidine-2,6-dione |
| F3023-0420 | 1-cyclohexyl-3-{[2-(3,4-dimethoxyphenyl)ethyl]amino}pyrrolidine-2,5-dione |
| F2135-1153 | (quinolin-2-yl)methanamine |
| F5834-1577 | N-(1-cyclopropanecarbonyl-2,3-dihydro-1H-indol-6-yl)-2-(2,5-dioxopyrrolidin-1-yl)acetamide |
| F0908-3710 | 3-[(4,6-dimethylpyrimidin-2-yl)amino]phenol |
| F0326-0654 | 4-(2,5-dioxopyrrolidin-1-yl)-N-[5-(propan-2-yl)-1,3,4-thiadiazol-2-yl]benzamide |
| F5033-3623 | phenyl 4-{[2-(2,5-dioxopyrrolidin-1-yl)acetamido]methyl}piperidine-1-carboxylate |
| F6359-3472 | N-(3,4-dichlorophenyl)-4-(pyridazin-3-yloxy)piperidine-1-carboxamide |
| F0211-0006 | 5-chloro-2-hydroxybenzoic acid |
| F6381-4336 | 1-(2-{[3-(4-fluorophenyl)azepan-1-yl]sulfonyl}ethyl)piperidine-2,6-dione |
| F3325-0010 | tert-butyl 4-[1-(2-methoxyphenyl)-2,5-dioxopyrrolidin-3-yl]piperazine-1-carboxylate |
| F0326-0620 | 4-(2,5-dioxopyrrolidin-1-yl)-N-(5-ethyl-1,3,4-thiadiazol-2-yl)benzamide |
| F5939-1818 | 1-(2-{4-[(6-fluoro-1,3-benzothiazol-2-yl)oxy]piperidin-1-yl}-2-oxoethyl)pyrrolidine-2,5-dione |
| F5831-7018 | 2-(2,6-dioxopiperidin-1-yl)-N-({1-[(thiophen-2-yl)methyl]piperidin-4-yl}methyl)ethane-1-sulfonamide |
| F5729-1223 | 1-(2-{4-[(5,6-dimethyl-1H-1,3-benzodiazol-1-yl)methyl]piperidin-1-yl}-2-oxoethyl)pyrrolidine-2,5-dione; oxalic acid |
| F3316-0301 | 2-(4-bromo-3,5-dimethyl-1H-pyrazol-1-yl)-N-(4-methoxyphenyl)acetamide |
| F0901-0811 | 3-[3-(4-methoxyphenyl)-1,2,4-oxadiazol-5-yl]propanoic acid |
| F5225-0161 | N-[6-(ethylsulfanyl)pyridazin-3-yl]-2-fluorobenzamide |
| F3316-0240 | N-[2-(4-chloro-3,5-dimethyl-1H-pyrazol-1-yl)ethyl]-3,4-dimethylbenzene-1-sulfonamide |
| F6064-3285 | 1-[2-oxo-2-(3-{3-[3-(trifluoromethyl)phenyl]-1,2,4-oxadiazol-5-yl}azetidin-1-yl)ethyl]pyrrolidine-2,5-dione |
| F3083-0114 | N-{4-[3-(4-bromo-3,5-dimethyl-1H-pyrazol-1-yl)-2-hydroxypropoxy]phenyl}acetamide |
| F6064-2776 | 1-(2-{3-[3-(3-chlorophenyl)-1,2,4-oxadiazol-5-yl]azetidin-1-yl}-2-oxoethyl)pyrrolidine-2,5-dione |
| F5381-0001 | 1-{2-[(4-phenylpiperazin-1-yl)sulfonyl]ethyl}piperidine-2,6-dione |
| F6374-5610 | 1-(2-{[4-({[(furan-2-yl)methyl]sulfanyl}methyl)piperidin-1-yl]sulfonyl}ethyl)piperidine-2,6-dione |
| F3083-0108 | 1-(4-bromo-3,5-dimethyl-1H-pyrazol-1-yl)-3-(2,3-dihydro-1H-inden-5-yloxy)propan-2-ol |
| F6469-1740 | 2-(2,5-dioxopyrrolidin-1-yl)-N-(2-{imidazo[1,2-a]pyridin-2-yl}phenyl)acetamide |
| F6244-3126 | 2-(2,6-dioxopiperidin-1-yl)-N-[4-(1,2,3,4-tetrahydroisoquinolin-2-yl)but-2-yn-1-yl]ethane-1-sulfonamide |
| F1913-9284 | 4-hydroxybutyl benzoate |
| F5493-0462 | 1-(2-{3-[5-(2-fluorophenyl)-1,3,4-thiadiazol-2-yl]piperidin-1-yl}-2-oxoethyl)pyrrolidine-2,5-dione |
| F5939-1176 | 1-(2-{4-[(4-chloro-1,3-benzothiazol-2-yl)oxy]piperidin-1-yl}-2-oxoethyl)pyrrolidine-2,5-dione |
| F3230-0004 | 3-(4-bromo-3,5-dimethyl-1H-pyrazol-1-yl)-N-(4-methylphenyl)butanamide |
| F5823-0653 | N-(3,4-dichlorophenyl)-4-[(1H-pyrazol-1-yl)methyl]piperidine-1-carboxamide |
| F3230-0069 | 3-(4-bromo-3,5-dimethyl-1H-pyrazol-1-yl)-N-(4-fluorophenyl)propanamide |
| F5860-4825 | 1-[2-({4-[(3,5-dimethyl-1,2-oxazol-4-yl)methyl]piperazin-1-yl}sulfonyl)ethyl]piperidine-2,6-dione |
| F3230-0014 | N-(5-chloro-2-methoxyphenyl)-3-(4-chloro-3,5-dimethyl-1H-pyrazol-1-yl)propanamide |
| F6359-3074 | N-(3,4-dichlorophenyl)-4-(pyrazin-2-yloxy)piperidine-1-carboxamide |
| F3230-0073 | N-(3-acetylphenyl)-3-(4-bromo-3,5-dimethyl-1H-pyrazol-1-yl)propanamide |
| F3230-0020 | N-(5-chloro-2-methylphenyl)-3-(4-chloro-3,5-dimethyl-1H-pyrazol-1-yl)butanamide |
| F6359-0288 | N-(3,4-dichlorophenyl)-4-(pyridin-2-yloxy)piperidine-1-carboxamide |
| F5855-0774 | 1-(2-{4-[2-(4-fluorophenyl)cyclopropanecarbonyl]piperazin-1-yl}-2-oxoethyl)pyrrolidine-2,5-dione |
| F3230-0010 | N-(5-chloro-2-methoxyphenyl)-3-(4-chloro-3,5-dimethyl-1H-pyrazol-1-yl)butanamide |
| F6245-0328 | N-(3,4-dichlorophenyl)-4-[5-(trifluoromethyl)-1,3,4-oxadiazol-2-yl]piperidine-1-carboxamide |
| F5381-0007 | 1-(2-{[4-(2-fluorophenyl)piperazin-1-yl]sulfonyl}ethyl)piperidine-2,6-dione |
| F3316-0246 | 3-bromo-N-[2-(4-chloro-3,5-dimethyl-1H-pyrazol-1-yl)ethyl]benzamide |
| F5831-9365 | 2-(2,6-dioxopiperidin-1-yl)-N-[(4-phenyloxan-4-yl)methyl]ethane-1-sulfonamide |
| F6057-0486 | N-(3,4-dichlorophenyl)-4-[2-(1H-pyrazol-1-yl)ethyl]piperazine-1-carboxamide |
| F6782-3511 | 4-(4-tert-butylphenyl)-4-oxobutanoic acid |
| F2167-9989 | 2-methyl-1H,2H,3H,4H,9H-pyrido[3,4-b]indole-1-carboxylic acid |
| F5831-6629 | N-[(1-benzylpiperidin-4-yl)methyl]-2-(2,6-dioxopiperidin-1-yl)ethane-1-sulfonamide |
| F5381-0021 | 1-(2-{[4-(3-methylphenyl)piperazin-1-yl]sulfonyl}ethyl)piperidine-2,6-dione |
| F5577-0281 | ethyl 6-fluoro-1-methyl-4-oxo-7-(piperidin-1-yl)-1,4-dihydroquinoline-3-carboxylate |
| F5381-0024 | 1-(2-{[4-(2,5-dimethylphenyl)piperazin-1-yl]sulfonyl}ethyl)piperidine-2,6-dione |
| F9994-6554 | ethyl 3-oxocyclopentane-1-carboxylate |
| F2163-0132 | (1S,3R)-3-(methoxycarbonyl)cyclohexane-1-carboxylic acid |
| F2167-1144 | tert-butyl 3-hydroxy-8-azabicyclo[3.2.1]octane-8-carboxylate |
| F2167-9988 | 1H,2H,3H,4H,9H-pyrido[3,4-b]indole-1-carboxylic acid |
| F6359-1880 | 4-[(3-cyanopyridin-2-yl)oxy]-N-(3,4-dichlorophenyl)piperidine-1-carboxamide |
| F1913-8005 | 2-{[(benzyloxy)carbonyl]amino}-4-fluorobutanoic acid |
| F0722-8675 | 7-methoxy-1-methyl-9H-pyrido[3,4-b]indole |
| F2733-0046 | 4-(2,5-dioxopyrrolidin-1-yl)-N-{thieno[2,3-d]pyrimidin-4-yl}benzamide |
| F2158-2187 | (5-hydroxy-4-oxo-4H-pyran-2-yl)methyl acetate |
| F5381-0010 | 1-(2-{[4-(2-methoxyphenyl)piperazin-1-yl]sulfonyl}ethyl)piperidine-2,6-dione |
| F5381-0005 | 1-(2-{[4-(2-chlorophenyl)piperazin-1-yl]sulfonyl}ethyl)piperidine-2,6-dione |
| F5381-0015 | 1-(2-{[4-(4-acetylphenyl)piperazin-1-yl]sulfonyl}ethyl)piperidine-2,6-dione |
| F6356-0566 | 2-(2,5-dioxopyrrolidin-1-yl)-N-[1-(furan-3-yl)propan-2-yl]acetamide |
| F3259-0262 | 1,3-dimethyl-7-pentyl-2,3,6,7-tetrahydro-1H-purine-2,6-dione |
| F6037-0311 | 1-(2-{[3-(1H-1,3-benzodiazol-2-yl)azetidin-1-yl]sulfonyl}ethyl)piperidine-2,6-dione |
| F5461-1211 | 1-(4-{4-[(4-cyclopropyl-1,3-thiazol-2-yl)methyl]piperazine-1-carbonyl}phenyl)pyrrolidine-2,5-dione |
| F6057-0607 | N-(3,4-dichlorophenyl)-4-[2-(3,5-dimethyl-1H-pyrazol-1-yl)ethyl]piperazine-1-carboxamide |
| F5592-0043 | 1-[2-({4-[5-(5-methyl-1,2,4-oxadiazol-3-yl)pyridin-2-yl]piperazin-1-yl}sulfonyl)ethyl]piperidine-2,6-dione |
| F5381-0017 | 1-(2-{[4-(2-phenylethyl)piperazin-1-yl]sulfonyl}ethyl)piperidine-2,6-dione |
| F5825-0161 | 1-(2-{[4-(1-methyl-1H-1,3-benzodiazol-2-yl)piperazin-1-yl]sulfonyl}ethyl)piperidine-2,6-dione |
| F1903-9727 | N1-methyl-N4-phenylbenzene-1,4-diamine dihydrochloride |
| F6439-1216 | 2-(2,5-dioxopyrrolidin-1-yl)-N-{2-[3-(trifluoromethyl)-4,5,6,7-tetrahydro-1H-indazol-1-yl]ethyl}acetamide |
| F1916-0149 | 1,4-diethyl benzene-1,4-dicarboxylate |
| F2158-1561 | 1-acetylazepan-4-one |
| F3350-0613 | 4-(2,5-dioxopyrrolidin-1-yl)-N-(4-methylpyridin-2-yl)benzene-1-sulfonamide |
| F3316-0237 | N-[2-(4-chloro-3,5-dimethyl-1H-pyrazol-1-yl)ethyl]benzenesulfonamide |
| F6439-3514 | N-{2-[5-cyclopropyl-3-(trifluoromethyl)-1H-pyrazol-1-yl]ethyl}-2-(2,5-dioxopyrrolidin-1-yl)acetamide |
| F1468-0051 | 7-[(azepan-1-yl)methyl]-1,3-dimethyl-2,3,6,7-tetrahydro-1H-purine-2,6-dione |
| F1468-0053 | 1,3-dimethyl-7-[(2-methylpiperidin-1-yl)methyl]-2,3,6,7-tetrahydro-1H-purine-2,6-dione |
| F5381-0026 | 1-(2-{[4-(5-chloro-2-methylphenyl)piperazin-1-yl]sulfonyl}ethyl)piperidine-2,6-dione |
| F6415-0386 | 1-(3,4-dichlorophenyl)-3-[(furan-3-yl)methyl]-3-(2-methoxyethyl)urea |
| F0745-1002 | 2-[(thiophen-2-yl)formamido]acetic acid |
| F5832-4178 | N-[2-(dimethylamino)-2-(thiophen-2-yl)ethyl]-2-(2,5-dioxopyrrolidin-1-yl)acetamide |
| F5940-1920 | 1-[2-({4-[(benzyloxy)methyl]piperidin-1-yl}sulfonyl)ethyl]piperidine-2,6-dione |
| F3259-0266 | 1,3-dimethyl-7-[(4-methylphenyl)methyl]-2,3,6,7-tetrahydro-1H-purine-2,6-dione |
| F3316-0238 | N-[2-(4-chloro-3,5-dimethyl-1H-pyrazol-1-yl)ethyl]-4-methylbenzene-1-sulfonamide |
| F1468-0150 | 1,3-dimethyl-7-[(3-methylpiperidin-1-yl)methyl]-2,3,6,7-tetrahydro-1H-purine-2,6-dione |
| F9994-0533 | 1-(2-hydroxy-3-methoxyphenyl)ethan-1-one |
| F6374-1842 | 1-{2-[(4-{[(4-methoxyphenyl)sulfanyl]methyl}piperidin-1-yl)sulfonyl]ethyl}piperidine-2,6-dione |
| F2185-0118 | 3-[3-(thiophen-2-yl)-1,2,4-oxadiazol-5-yl]propan-1-amine hydrochloride |
| F5381-0016 | 1-[2-({4-[(2H-1,3-benzodioxol-5-yl)methyl]piperazin-1-yl}sulfonyl)ethyl]piperidine-2,6-dione |
| F2145-0662 | 6-(3-methyl-1,2,4-oxadiazol-5-yl)quinoxaline |
| F3259-0258 | 7-benzyl-1,3-dimethyl-2,3,6,7-tetrahydro-1H-purine-2,6-dione |
| F5833-7898 | 2-(2,5-dioxopyrrolidin-1-yl)-N-[1-(propane-1-sulfonyl)-1,2,3,4-tetrahydroquinolin-6-yl]acetamide |
| F2185-0119 | 3-[3-(furan-2-yl)-1,2,4-oxadiazol-5-yl]propan-1-amine hydrochloride |
| F5381-0025 | 1-(2-{[4-(2,3-dichlorophenyl)piperazin-1-yl]sulfonyl}ethyl)piperidine-2,6-dione |
| F6452-1112 | N-[2-(3-chlorophenyl)-2-methoxypropyl]-2-(2,5-dioxopyrrolidin-1-yl)acetamide |
| F2590-0085 | N-(3-cyano-4,5-dimethylthiophen-2-yl)-2-(2,5-dioxopyrrolidin-1-yl)acetamide |
| F0921-3451 | 7-[(4-chlorophenyl)methyl]-1,3-dimethyl-2,3,6,7-tetrahydro-1H-purine-2,6-dione |
| F1064-0183 | 8-(ethylamino)-1,3-dimethyl-7-propyl-2,3,6,7-tetrahydro-1H-purine-2,6-dione |
| F3259-0268 | 1,3-dimethyl-7-(3-phenylpropyl)-2,3,6,7-tetrahydro-1H-purine-2,6-dione |
| F6451-2678 | N-[2-(2-chlorophenyl)-2-methoxyethyl]-2-(2,5-dioxopyrrolidin-1-yl)acetamide |
| F0440-0324 | 2-(2,5-dioxopyrrolidin-1-yl)-N-(6-methyl-4,5,6,7-tetrahydro-1,3-benzothiazol-2-yl)acetamide |
| F1913-1938 | 5-methylnaphthalene-1-carboxylic acid |
| F5882-0705 | 1-(2-oxo-2-{4-[(thiophen-3-yl)methyl]piperazin-1-yl}ethyl)pyrrolidine-2,5-dione hydrochloride |
| F5577-0013 | 6-fluoro-1-methyl-7-(4-methylpiperidin-1-yl)-4-oxo-1,4-dihydroquinoline-3-carboxylic acid |
| F5831-1860 | 2-(2,5-dioxopyrrolidin-1-yl)-N-[1-(furan-2-yl)propan-2-yl]acetamide |
| F0440-0382 | N-(3-cyano-4,5,6,7-tetrahydro-1-benzothiophen-2-yl)-2-(2,5-dioxopyrrolidin-1-yl)acetamide |
| F5831-2307 | 2-(2,5-dioxopyrrolidin-1-yl)-N-[1-(thiophen-2-yl)propan-2-yl]acetamide |
| F3146-0160 | tert-butyl 4-hydroxypiperidine-1-carboxylate |
| F1967-6724 | 4-methylbenzene-1-sulfonic acid; benzyl 2-aminoacetate |
| F6374-0794 | 1-[2-(4-{[(4-methyl-1,3-thiazol-2-yl)sulfanyl]methyl}piperidin-1-yl)-2-oxoethyl]pyrrolidine-2,5-dione |
| F0020-1632 | 2-[(adamantan-1-yl)formamido]acetic acid |
| F0440-0383 | 2-[2-(2,5-dioxopyrrolidin-1-yl)acetamido]-6-methyl-4,5,6,7-tetrahydro-1-benzothiophene-3-carboxamide |
| F6617-3967 | 3-[3-(2H-1,3-benzodioxol-5-yl)-1,2,4-oxadiazol-5-yl]propanoic acid |
| F8890-6347 | 4-oxo-4-(5,6,7,8-tetrahydronaphthalen-2-yl)butanoic acid |
| F5796-1601 | N-(4-chloro-2-cyanophenyl)-4-(dimethylsulfamoyl)-2,5-dimethylfuran-3-carboxamide |
| F6374-5504 | 1-{2-[4-({[(furan-2-yl)methyl]sulfanyl}methyl)piperidin-1-yl]-2-oxoethyl}pyrrolidine-2,5-dione |
| F6277-0404 | 1-[2-({4-[4-(trifluoromethyl)-1,3-benzothiazol-2-yl]piperazin-1-yl}sulfonyl)ethyl]piperidine-2,6-dione |
| F2184-0204 | tert-butyl 2-(2-hydroxyethyl)piperidine-1-carboxylate |
| F1907-0661 | 1H,2H,3H,4H,5H,10H-benzo[b]1,6-naphthyridin-10-one |
| F6477-5329 | 1-({2-[(2H-1,3-benzodioxol-5-yl)amino]-1,3-thiazol-4-yl}methyl)pyrrolidine-2,5-dione hydrochloride |
| F0806-0416 | 2-[2-(2,5-dioxopyrrolidin-1-yl)acetamido]-4H,5H,6H,7H,8H-cyclohepta[b]thiophene-3-carboxamide |
| F6477-5323 | 1-[(2-{[4-(dimethylamino)phenyl]amino}-1,3-thiazol-4-yl)methyl]pyrrolidine-2,5-dione hydrochloride |
| F0700-0135 | N-(5,5-dimethyl-7-oxo-4,5,6,7-tetrahydro-1,3-benzothiazol-2-yl)-2-(2,5-dioxopyrrolidin-1-yl)acetamide |
| F6401-0078 | 1-[(2-phenyl-1,3-thiazol-4-yl)methyl]-4-(thiophen-2-yl)piperidine-2,6-dione |
| F5896-0231 | 2-(2,5-dioxopyrrolidin-1-yl)-N-[2-(1H-pyrrol-1-yl)quinolin-8-yl]acetamide |
| F0526-1128 | N-[3-cyano-6-(propan-2-yl)-4H,5H,6H,7H-thieno[2,3-c]pyridin-2-yl]-2-(2,5-dioxopyrrolidin-1-yl)acetamide hydrochloride |
| F0001-0330 | 1,4-dimethyl benzene-1,4-dicarboxylate |
| F9994-6549 | 4-oxo-4-[4-(propan-2-yl)phenyl]butanoic acid |
| F9994-0695 | 1-{1H,2H,3H,4H,9H-pyrido[3,4-b]indol-1-yl}propan-2-one hydrochloride |
| F5795-1955 | N-(4-chloro-2-cyanophenyl)-4-[(2,6-dimethylmorpholin-4-yl)sulfonyl]-5-methylfuran-2-carboxamide |
| F6782-6659 | 1-(cyclopropylmethyl)-6-fluoro-4-oxo-1,4-dihydroquinoline-3-carboxylic acid |
| F5854-3791 | N-cyclopropyl-2-(2,5-dioxopyrrolidin-1-yl)-N-[(1-methyl-1H-pyrrol-2-yl)methyl]acetamide |
| F3376-1857 | 8-(5-chloro-2-hydroxyphenyl)-1,6,7-trimethyl-1H,2H,3H,4H,8H-imidazo[1,2-g]purine-2,4-dione |
| F6036-2026 | 1-(2-{[3-(3-benzyl-1,2,4-oxadiazol-5-yl)azetidin-1-yl]sulfonyl}ethyl)piperidine-2,6-dione |
| F2158-0411 | 2-(hydroxymethyl)-5-methoxy-4H-pyran-4-one |
| F1957-0079 | 4-{4-[(tert-butoxy)carbonyl]piperazin-1-yl}pyridine-2-carboxylic acid |
| F9995-0424 | 3,5-di-tert-butylbenzoic acid |
| F2156-0063 | 1-ethyl-6-fluoro-4-oxo-1,4-dihydroquinoline-3-carboxylic acid |
| F6451-0068 | 2-(2,5-dioxopyrrolidin-1-yl)-N-[2-methoxy-2-(2-methoxyphenyl)ethyl]acetamide |
| F5494-0170 | 1-[2-({3-[5-(2-fluorophenyl)-1,3,4-thiadiazol-2-yl]piperidin-1-yl}sulfonyl)ethyl]piperidine-2,6-dione |
| F6414-0594 | 2-(2-chlorophenyl)-N-[2-(2,3-dihydro-1,4-benzodioxin-6-yl)-2-hydroxyethyl]acetamide |
| F6443-0044 | 2-(2,5-dioxopyrrolidin-1-yl)-N-[4-(3-methoxypiperidin-1-yl)phenyl]acetamide |
| F5460-0040 | N-[1-(1,3-benzothiazol-2-yl)-3-methyl-1H-pyrazol-5-yl]-2-(2,5-dioxopyrrolidin-1-yl)acetamide |
| F2477-0100 | 1,3-dimethyl-7-(2-methylpropyl)-8-(propylamino)-2,3,6,7-tetrahydro-1H-purine-2,6-dione |
| F6439-0833 | 2-(2,5-dioxopyrrolidin-1-yl)-N-{2-[3-(pyridin-2-yl)-4,5,6,7-tetrahydro-1H-indazol-1-yl]ethyl}acetamide |
| F3260-0811 | 1,3-dimethyl-8-[(2-methylpropyl)amino]-7-propyl-2,3,6,7-tetrahydro-1H-purine-2,6-dione |
| F1800-0015 | N-(2,3-dimethylphenyl)-2-(3,5-dioxothiomorpholin-4-yl)acetamide |
| F6372-1997 | 2-(2,5-dioxopyrrolidin-1-yl)-N-(2-{[3-(furan-2-yl)-1,2,4-oxadiazol-5-yl]methyl}phenyl)acetamide |
| F6439-1982 | N-{2-[5-cyclopropyl-3-(thiophen-2-yl)-1H-pyrazol-1-yl]ethyl}-2-(2,5-dioxopyrrolidin-1-yl)acetamide |
| F0381-3870 | 3-methyl-7-pentyl-2,3,6,7-tetrahydro-1H-purine-2,6-dione |
| F6473-3916 | 1-[1-(2,5-difluorobenzenesulfonyl)azetidin-3-yl]pyrrolidine-2,5-dione |
| F6473-3934 | 1-[1-(3-chloro-4-fluorobenzenesulfonyl)azetidin-3-yl]pyrrolidine-2,5-dione |
| F6064-0987 | N-(3,4-dichlorophenyl)-3-[3-(pyrimidin-2-yl)-1,2,4-oxadiazol-5-yl]azetidine-1-carboxamide |
| F6413-0563 | 2-(2,5-dioxopyrrolidin-1-yl)-N-{[5-(furan-2-carbonyl)thiophen-2-yl]methyl}acetamide |
| F6415-0068 | 2-(2,5-dioxopyrrolidin-1-yl)-N-[(furan-3-yl)methyl]-N-(2-methoxyethyl)acetamide |
| F3023-0925 | 3-{[4-(dimethylamino)phenyl]amino}-1-ethylpyrrolidine-2,5-dione |
| F6473-3604 | 1-[1-(9H-xanthene-9-carbonyl)azetidin-3-yl]pyrrolidine-2,5-dione |
| F1800-0168 | 2-(2-methyl-3,5-dioxothiomorpholin-4-yl)-N-(2,4,6-trimethylphenyl)acetamide |
| F5831-9284 | 2-(2,5-dioxopyrrolidin-1-yl)-N-[(4-phenyloxan-4-yl)methyl]acetamide |
| F5753-0272 | 1-[2-({4-[(5,6-dimethyl-1H-1,3-benzodiazol-1-yl)methyl]piperidin-1-yl}sulfonyl)ethyl]piperidine-2,6-dione |
| F5460-0500 | 2-(2,5-dioxopyrrolidin-1-yl)-N-[1-(6-fluoro-1,3-benzothiazol-2-yl)-3-methyl-1H-pyrazol-5-yl]acetamide |
| F3023-0980 | 1-methyl-3-{[4-(piperidin-1-yl)phenyl]amino}pyrrolidine-2,5-dione |
| F5831-9107 | 2-(2,5-dioxopyrrolidin-1-yl)-N-{[1-(4-fluorophenyl)cyclopropyl]methyl}acetamide |
| F1800-0053 | 2-(3,5-dioxothiomorpholin-4-yl)-N-(2,4,6-trimethylphenyl)acetamide |
| F6473-3933 | 1-[1-(naphthalene-1-sulfonyl)azetidin-3-yl]pyrrolidine-2,5-dione |
| F3395-2209 | 2-phenylethyl butanoate |
| F1167-0035 | 7-hexyl-1,3-dimethyl-8-(methylamino)-2,3,6,7-tetrahydro-1H-purine-2,6-dione |
| F3260-0382 | 7-ethyl-1,3-dimethyl-8-(pentylamino)-2,3,6,7-tetrahydro-1H-purine-2,6-dione |
| F6412-1460 | 2-(2,5-dioxopyrrolidin-1-yl)-N-[4-(2-methoxyphenoxy)but-2-yn-1-yl]acetamide |
| F1800-0173 | N-(4-fluorophenyl)-2-(2-methyl-3,5-dioxothiomorpholin-4-yl)acetamide |
| F6274-0155 | 1-(4-methoxy-1,3-benzothiazol-2-yl)azetidin-3-yl 2-(2,5-dioxopyrrolidin-1-yl)acetate |
| F1907-0022 | 2-[3-(2H-1,3-benzodioxol-5-yl)-1,2,4-oxadiazol-5-yl]acetonitrile |
| F6448-2277 | N-{1-[(2,3-dihydro-1,4-benzodioxin-2-yl)methyl]-1H-pyrazol-4-yl}-2-(2,5-dioxopyrrolidin-1-yl)acetamide |
| F0526-0357 | ethyl 2-[2-(2,5-dioxopyrrolidin-1-yl)acetamido]-6-ethyl-4H,5H,6H,7H-thieno[2,3-c]pyridine-3-carboxylate hydrochloride |
| F1800-0016 | N-(2,5-dimethylphenyl)-2-(3,5-dioxothiomorpholin-4-yl)acetamide |
| F1800-0017 | N-(2,4-dimethylphenyl)-2-(3,5-dioxothiomorpholin-4-yl)acetamide |
| F1800-0171 | N-(2-fluorophenyl)-2-(2-methyl-3,5-dioxothiomorpholin-4-yl)acetamide |
| F2189-0266 | 5-amino-1-methyl-2,3-dihydro-1H-indol-2-one |
| F0737-0267 | 4-(2,5-dioxopyrrolidin-1-yl)-N-[4-(5-methylfuran-2-yl)-1,3-thiazol-2-yl]benzamide |
| F0737-0314 | 3-(2,5-dioxopyrrolidin-1-yl)-N-[4-(5-methylfuran-2-yl)-1,3-thiazol-2-yl]benzamide |
| F2257-0230 | N-(2-oxo-2,3-dihydro-1H-indol-5-yl)butanamide |
| F1800-0165 | N-(2,5-dimethylphenyl)-2-(2-methyl-3,5-dioxothiomorpholin-4-yl)acetamide |
| F1130-0080 | 2-[2-(adamantan-1-yl)acetamido]acetic acid |
| F1593-0047 | 3-{[4-(morpholin-4-yl)phenyl]amino}-1-(propan-2-yl)pyrrolidine-2,5-dione |
| F1800-0177 | N-(2,4-difluorophenyl)-2-(2-methyl-3,5-dioxothiomorpholin-4-yl)acetamide |
| F2191-0004 | 1-(4-hydroxy-3-methoxyphenyl)ethan-1-one |
| F6439-2748 | N-{2-[5-cyclopropyl-3-(pyridin-2-yl)-1H-pyrazol-1-yl]ethyl}-2-(2,5-dioxopyrrolidin-1-yl)acetamide |
| F6473-3950 | 1-[1-(2,4-dichloro-5-methylbenzenesulfonyl)azetidin-3-yl]pyrrolidine-2,5-dione |
| F0789-0022 | 3-(2,5-dioxopyrrolidin-1-yl)-N-[4-(pyridin-3-yl)-1,3-thiazol-2-yl]benzamide |
| F3023-0954 | 1-ethyl-3-{[4-(morpholin-4-yl)phenyl]amino}pyrrolidine-2,5-dione |
| F0896-0475 | 1-methyl-1H,2H,3H,4H,9H-pyrido[3,4-b]indole-3-carboxylic acid |
| F5857-2313 | 2-(2,5-dioxopyrrolidin-1-yl)-N-[2-hydroxy-2-(naphthalen-1-yl)ethyl]acetamide |
| F0700-0021 | 4-(2,5-dioxopyrrolidin-1-yl)-N-[4-(pyridin-2-yl)-1,3-thiazol-2-yl]benzamide |
| F9995-0575 | 3'-methoxy-[1,1'-biphenyl]-4-carboxylic acid |
| F0715-0484 | 4-(2,5-dioxopyrrolidin-1-yl)-N-[4-(pyridin-4-yl)-1,3-thiazol-2-yl]benzamide |
| F2590-0359 | 2-[2-(2,5-dioxopyrrolidin-1-yl)acetamido]-N,4,5-trimethylthiophene-3-carboxamide |
| F1800-0018 | N-(3,5-dimethylphenyl)-2-(3,5-dioxothiomorpholin-4-yl)acetamide |
| F1800-0049 | 2-(3,5-dioxothiomorpholin-4-yl)-N-(3-methoxyphenyl)acetamide |
| F1800-0182 | N-(3-methoxyphenyl)-2-(2-methyl-3,5-dioxothiomorpholin-4-yl)acetamide |
| F1175-0181 | 2-chloro-4-nitro-N-[(oxolan-2-yl)methyl]benzamide |
| F6412-1869 | 2-(2,5-dioxopyrrolidin-1-yl)-N-{4-[3-(trifluoromethyl)phenoxy]but-2-yn-1-yl}acetamide |
| F3023-0956 | 3-{[4-(morpholin-4-yl)phenyl]amino}-1-propylpyrrolidine-2,5-dione |
| F0440-0406 | N-(4-bromophenyl)-2-(2,5-dioxopyrrolidin-1-yl)acetamide |
| F1800-0181 | N-(2-methoxyphenyl)-2-(2-methyl-3,5-dioxothiomorpholin-4-yl)acetamide |
| F2156-0041 | 1-ethyl-8-fluoro-4-oxo-1,4-dihydroquinoline-3-carboxylic acid |
| F6414-3116 | N-[2-(3,4-dimethoxyphenyl)-2-hydroxyethyl]-2-phenoxypropanamide |
| F1800-0169 | N-(4-ethylphenyl)-2-(2-methyl-3,5-dioxothiomorpholin-4-yl)acetamide |
| F3023-0406 | 3-[(4-methoxyphenyl)amino]-1-(propan-2-yl)pyrrolidine-2,5-dione |
| F1800-0167 | N-(3,5-dimethylphenyl)-2-(2-methyl-3,5-dioxothiomorpholin-4-yl)acetamide |
| F1800-0180 | N-(5-chloro-2-methoxyphenyl)-2-(2-methyl-3,5-dioxothiomorpholin-4-yl)acetamide |
| F1800-0041 | N-(5-chloro-2-methoxyphenyl)-2-(3,5-dioxothiomorpholin-4-yl)acetamide |
| F6274-0681 | 1-(1,3-benzothiazol-2-yl)azetidin-3-yl 2-(2,5-dioxopyrrolidin-1-yl)acetate |
| F1800-0008 | 2-(3,5-dioxothiomorpholin-4-yl)-N-(4-methoxyphenyl)acetamide |
| F3205-0032 | 3-{[4-(morpholin-4-yl)phenyl]amino}-1-(prop-2-en-1-yl)pyrrolidine-2,5-dione |
| F6413-0068 | 2-(2,5-dioxopyrrolidin-1-yl)-N-{[5-(thiophene-2-carbonyl)thiophen-2-yl]methyl}acetamide |
| F9995-2665 | 4-[(1E)-2-(4-hydroxy-3-methoxyphenyl)ethenyl]-2-methoxyphenol |
| F3115-0095 | ethyl 4-[1-(2-methylphenyl)-2,5-dioxopyrrolidin-3-yl]piperazine-1-carboxylate |
| F1800-0014 | 2-(3,5-dioxothiomorpholin-4-yl)-N-(4-ethoxyphenyl)acetamide |
| F1800-0022 | N-(2H-1,3-benzodioxol-5-yl)-2-(3,5-dioxothiomorpholin-4-yl)acetamide |
| F3023-0835 | 3-[(4-ethoxyphenyl)amino]-1-(2-methoxyethyl)pyrrolidine-2,5-dione |
| F3023-0907 | 3-(4-benzylpiperazin-1-yl)-1-propylpyrrolidine-2,5-dione |
| F0440-0367 | N-(4-acetylphenyl)-2-(2,5-dioxopyrrolidin-1-yl)acetamide |
| F0440-0381 | ethyl 2-[2-(2,5-dioxopyrrolidin-1-yl)acetamido]-4,5,6,7-tetrahydro-1-benzothiophene-3-carboxylate |
| F1800-0184 | N-(2-ethoxyphenyl)-2-(2-methyl-3,5-dioxothiomorpholin-4-yl)acetamide |
| F5476-0546 | N-[2-(dimethylamino)-2-(1-methyl-1H-indol-3-yl)ethyl]-2-(2,5-dioxopyrrolidin-1-yl)acetamide |
| F1800-0176 | N-(4-bromophenyl)-2-(2-methyl-3,5-dioxothiomorpholin-4-yl)acetamide |
| F3222-1846 | 11-acetyl-4-(4-methoxyphenyl)-5-(methylsulfanyl)-8-thia-4,6,11-triazatricyclo[7.4.0.0^{2,7}]trideca-1(9),2(7),5-trien-3-one |
| F5065-0008 | N-(3,4-dichlorophenyl)-4-{3-methyl-3H-[1,2,3]triazolo[4,5-d]pyrimidin-7-yl}piperazine-1-carboxamide |
| F5854-1753 | 2-(2,5-dioxopyrrolidin-1-yl)-N-[(pyridin-3-yl)methyl]-N-[2-(thiophen-2-yl)ethyl]acetamide |
| F0440-0368 | N-(3-acetylphenyl)-2-(2,5-dioxopyrrolidin-1-yl)acetamide |
| F3023-0919 | 3-[4-(4-fluorophenyl)piperazin-1-yl]-1-propylpyrrolidine-2,5-dione |
| F1829-0108 | 2-[2-(2,5-dioxopyrrolidin-1-yl)acetamido]-N,6-dimethyl-4,5,6,7-tetrahydro-1-benzothiophene-3-carboxamide |
| F5033-5422 | 2-(2,5-dioxopyrrolidin-1-yl)-N-[(1-{[2-(methylsulfanyl)phenyl]methyl}piperidin-4-yl)methyl]acetamide |
| F6451-2738 | N-[2-(2-chlorophenyl)-2-methoxyethyl]-2-(2-methoxyphenoxy)acetamide |
| F1800-0175 | N-(4-chlorophenyl)-2-(2-methyl-3,5-dioxothiomorpholin-4-yl)acetamide |
| F1374-1104 | N-[5-(5-chlorothiophen-2-yl)-1,3,4-oxadiazol-2-yl]-3-(2,5-dioxopyrrolidin-1-yl)benzamide |
| F1757-0096 | 4-cyanophenyl morpholine-4-carboxylate |
| F1800-0174 | N-(3-chlorophenyl)-2-(2-methyl-3,5-dioxothiomorpholin-4-yl)acetamide |
| F6245-7288 | N-[2-(3-cyclopropyl-5-oxo-4-phenyl-4,5-dihydro-1H-1,2,4-triazol-1-yl)ethyl]-2-(2,5-dioxopyrrolidin-1-yl)acetamide |
| F1800-0186 | N-(2,4-dimethoxyphenyl)-2-(2-methyl-3,5-dioxothiomorpholin-4-yl)acetamide |
| F3115-0091 | ethyl 4-[1-(4-hydroxyphenyl)-2,5-dioxopyrrolidin-3-yl]piperazine-1-carboxylate |
| F6401-0050 | 2-[2,6-dioxo-4-(thiophen-2-yl)piperidin-1-yl]-N-(2-methoxyphenyl)acetamide |
| F1593-0022 | 1-cyclohexyl-3-{[4-(morpholin-4-yl)phenyl]amino}pyrrolidine-2,5-dione |
| F0348-2897 | ethyl N-(4-methoxyphenyl)carbamate |
| F0440-0357 | 2-(2,5-dioxopyrrolidin-1-yl)-N-[4-(thiophen-2-yl)-1,3-thiazol-2-yl]acetamide |
| F1335-0047 | methyl 2-[(4-methoxyphenyl)formamido]acetate |
| F1800-0185 | N-(4-ethoxyphenyl)-2-(2-methyl-3,5-dioxothiomorpholin-4-yl)acetamide |
| F3188-0012 | ethyl 4-[1-(3-methylphenyl)-2,5-dioxopyrrolidin-3-yl]piperazine-1-carboxylate |
| F2130-0132 | methyl 4-bromo-3-(4-fluorophenyl)-1H-pyrazole-5-carboxylate |
| F1800-0019 | N-(2,4-dimethoxyphenyl)-2-(3,5-dioxothiomorpholin-4-yl)acetamide |
| F1800-0187 | N-(2,5-dimethoxyphenyl)-2-(2-methyl-3,5-dioxothiomorpholin-4-yl)acetamide |
| F5870-0404 | 2-(2,5-dioxopyrrolidin-1-yl)-N-{[4-(2-methoxyphenyl)oxan-4-yl]methyl}acetamide |
| F6473-8345 | N-(3,4-dichlorophenyl)-4-[(6-methyl-2-oxo-2H-pyran-4-yl)oxy]piperidine-1-carboxamide |
| F2590-0037 | N-(3-cyano-4,5-dimethylthiophen-2-yl)-4-(2,5-dioxopyrrolidin-1-yl)benzamide |
| F3023-0932 | 3-{[4-(dimethylamino)phenyl]amino}-1-(2-methoxyethyl)pyrrolidine-2,5-dione |
| F3325-0155 | 1-(2-methylphenyl)-3-(4-phenylpiperazin-1-yl)pyrrolidine-2,5-dione |
| F1374-2755 | N-[5-(2,5-dichlorothiophen-3-yl)-1,3,4-oxadiazol-2-yl]-3-(2,5-dioxopyrrolidin-1-yl)benzamide |
| F3284-8018 | 2-(3-oxo-1,3-dihydro-2-benzofuran-1-yl)acetic acid |
| F3115-0097 | ethyl 4-[1-(2,3-dimethylphenyl)-2,5-dioxopyrrolidin-3-yl]piperazine-1-carboxylate |
| F3205-0042 | 3-[(4-fluorophenyl)amino]-1-(3-methoxypropyl)pyrrolidine-2,5-dione |
| F0722-1840 | 5-oxo-5-phenylpentanoic acid |
| F3115-0100 | ethyl 4-[1-(2,4-dimethylphenyl)-2,5-dioxopyrrolidin-3-yl]piperazine-1-carboxylate |
| F6416-5530 | N-[3-(1-benzofuran-2-yl)-3-hydroxypropyl]-2-(2,5-dioxopyrrolidin-1-yl)acetamide |
| F3023-0829 | 3-[(4-ethoxyphenyl)amino]-1-(propan-2-yl)pyrrolidine-2,5-dione |
| F5079-0263 | N-{5-[(5-chlorothiophen-2-yl)methyl]-1,3,4-oxadiazol-2-yl}-3-(2,5-dioxopyrrolidin-1-yl)benzamide |
| F1799-0236 | 3-(azepan-1-yl)-1-(4-bromophenyl)pyrrolidine-2,5-dione |
| F6064-2523 | N-(3,4-dichlorophenyl)-3-[3-(pyrazin-2-yl)-1,2,4-oxadiazol-5-yl]azetidine-1-carboxamide |
| F0412-0123 | 1-[4-(1,2,3,4-tetrahydroisoquinoline-2-carbonyl)phenyl]pyrrolidine-2,5-dione |
| F3284-7941 | 2,2-dimethyl-3,4-dihydro-2H-1-benzopyran-4-one |
| F3188-0121 | 3-[(2H-1,3-benzodioxol-5-yl)amino]-1-cyclohexylpyrrolidine-2,5-dione |
| F3325-0134 | 3-{[4-(dimethylamino)phenyl]amino}-1-(prop-2-en-1-yl)pyrrolidine-2,5-dione |
| F3188-0112 | 1-cyclopentyl-3-{[4-(piperidin-1-yl)phenyl]amino}pyrrolidine-2,5-dione |
| F5831-9881 | 2-(2,6-dioxopiperidin-1-yl)-N-{[1-(thiophen-2-yl)cyclopropyl]methyl}ethane-1-sulfonamide |
| F3023-0927 | 3-{[4-(dimethylamino)phenyl]amino}-1-(2-methylpropyl)pyrrolidine-2,5-dione |
| F1800-0179 | N-(3-chloro-4-methylphenyl)-2-(2-methyl-3,5-dioxothiomorpholin-4-yl)acetamide |
| F3205-0085 | 1-cyclohexyl-3-{[4-(dimethylamino)phenyl]amino}pyrrolidine-2,5-dione |
| F3023-0955 | 1-butyl-3-{[4-(morpholin-4-yl)phenyl]amino}pyrrolidine-2,5-dione |
| F5323-0338 | 2-(2,5-dioxopyrrolidin-1-yl)-N-{2-phenyl-2H,4H,5H,6H-cyclopenta[c]pyrazol-3-yl}acetamide |
| F2257-0235 | 2-(4-chlorophenyl)-N-(2-oxo-2,3-dihydro-1H-indol-5-yl)acetamide |
| F3325-0009 | tert-butyl 4-[1-(4-hydroxyphenyl)-2,5-dioxopyrrolidin-3-yl]piperazine-1-carboxylate |
| F2257-0257 | 2-(4-fluorophenyl)-N-(2-oxo-2,3-dihydro-1H-indol-5-yl)acetamide |
| F1800-0194 | 2-(2-methyl-3,5-dioxothiomorpholin-4-yl)-N-(4-nitrophenyl)acetamide |
| F3023-0958 | 1-(2-methoxyethyl)-3-{[4-(morpholin-4-yl)phenyl]amino}pyrrolidine-2,5-dione |
| F3188-0116 | 3-[(2H-1,3-benzodioxol-5-yl)amino]-1-(2-methylpropyl)pyrrolidine-2,5-dione |
| F1800-0035 | 2-(3,5-dioxothiomorpholin-4-yl)-N-(4-nitrophenyl)acetamide |
| F1918-0045 | 1-methyl-1H,2H,3H,4H,9H-pyrido[3,4-b]indole-3-carboxylic acid |
| F3323-0590 | 2-(3,5-dioxo-2,3,4,5-tetrahydro-1,4-benzoxazepin-4-yl)-N-(2-methylphenyl)acetamide |
| F2191-0017 | 6-methoxynaphthalene-2-carboxylic acid |
| F2668-0038 | 4-chloro-N-{2-oxo-1-azatricyclo[7.3.1.0^{5,13}]trideca-5,7,9(13)-trien-7-yl}benzamide |
| F2106-0149 | 6-amino-4H-chromen-4-one |
| F5791-0108 | 2-(2,5-dioxopyrrolidin-1-yl)-N-(2-methylquinolin-5-yl)acetamide |
| F6414-3168 | 2-(4-chlorophenoxy)-N-[2-(3,4-dimethoxyphenyl)-2-hydroxyethyl]acetamide |
| F0412-0104 | 1-[4-(2,3-dihydro-1H-indole-1-carbonyl)phenyl]pyrrolidine-2,5-dione |
| F1800-0178 | N-(3,4-dichlorophenyl)-2-(2-methyl-3,5-dioxothiomorpholin-4-yl)acetamide |
| F6401-0082 | 1-[(2,5-difluorophenyl)methyl]-4-(thiophen-2-yl)piperidine-2,6-dione |
| F5577-0009 | 6-fluoro-1-methyl-4-oxo-7-(pyrrolidin-1-yl)-1,4-dihydroquinoline-3-carboxylic acid |
| F1800-0052 | N-(3,4-dichlorophenyl)-2-(3,5-dioxothiomorpholin-4-yl)acetamide |
| F2749-0202 | N-(5-chloro-2-hydroxyphenyl)-3-(1,1,3-trioxo-2,3-dihydro-1lambda6,2-benzothiazol-2-yl)propanamide |
| F3205-0045 | 3-{[4-(piperidin-1-yl)phenyl]amino}-1-(prop-2-en-1-yl)pyrrolidine-2,5-dione |
| F3115-0088 | ethyl 4-[1-(3,4-dimethylphenyl)-2,5-dioxopyrrolidin-3-yl]piperazine-1-carboxylate |
| F3188-0079 | tert-butyl 4-[1-(2-methylphenyl)-2,5-dioxopyrrolidin-3-yl]piperazine-1-carboxylate |
| F0440-0407 | ethyl 4-[2-(2,5-dioxopyrrolidin-1-yl)acetamido]benzoate |
| F1243-0065 | 4-nitrophenyl piperidine-1-carboxylate |
| F2257-0267 | N-(2-oxo-2,3-dihydro-1H-indol-5-yl)-3-phenylpropanamide |
| F0440-0352 | N-[4-(5-chlorothiophen-2-yl)-1,3-thiazol-2-yl]-2-(2,5-dioxopyrrolidin-1-yl)acetamide |
| F5854-2938 | 2-(2,5-dioxopyrrolidin-1-yl)-N-[(1-methyl-1H-pyrrol-2-yl)methyl]-N-[(thiophen-2-yl)methyl]acetamide |
| F1374-1139 | N-[5-(5-chlorothiophen-2-yl)-1,3,4-oxadiazol-2-yl]-2-(2,5-dioxopyrrolidin-1-yl)acetamide |
| F6241-0243 | 4-benzyl-N-(5-chloro-2-hydroxyphenyl)-5-oxomorpholine-3-carboxamide |
| F6401-0051 | N-(2H-1,3-benzodioxol-5-yl)-2-[2,6-dioxo-4-(thiophen-2-yl)piperidin-1-yl]acetamide |
| F3115-0101 | ethyl 4-[1-(2-chlorophenyl)-2,5-dioxopyrrolidin-3-yl]piperazine-1-carboxylate |
| F0700-0138 | 2-(2,5-dioxopyrrolidin-1-yl)-N-[4-(pyridin-3-yl)-1,3-thiazol-2-yl]acetamide |
| F2767-0093 | N-[5-(3,4-dimethylphenyl)-1,3,4-oxadiazol-2-yl]-2-(2,5-dioxopyrrolidin-1-yl)acetamide |
| F0327-0367 | N-(2,4-difluorophenyl)-4-(2,5-dioxopyrrolidin-1-yl)benzamide |
| F0715-0490 | 2-(2,5-dioxopyrrolidin-1-yl)-N-[4-(pyridin-4-yl)-1,3-thiazol-2-yl]acetamide |
| F1300-0285 | N-(4,5-dimethyl-1,3-benzothiazol-2-yl)-2-(2,5-dioxopyrrolidin-1-yl)acetamide |
| F0327-0395 | N-(3-cyano-5-methyl-4,5,6,7-tetrahydro-1-benzothiophen-2-yl)-4-(2,5-dioxopyrrolidin-1-yl)benzamide |
| F3205-0102 | 3-[4-(4-fluorophenyl)piperazin-1-yl]-1-(2-methylpropyl)pyrrolidine-2,5-dione |
| F1374-2793 | N-[5-(2,5-dichlorothiophen-3-yl)-1,3,4-oxadiazol-2-yl]-2-(2,5-dioxopyrrolidin-1-yl)acetamide |
| F3083-0083 | 1-[4-(benzenesulfonyl)piperazin-1-yl]-3-(4-bromo-3,5-dimethyl-1H-pyrazol-1-yl)propan-1-one |
| F3205-0092 | 3-{[4-(dimethylamino)phenyl]amino}-1-(3-methoxypropyl)pyrrolidine-2,5-dione |
| F3205-0039 | 3-[(4-methoxyphenyl)amino]-1-(3-methoxypropyl)pyrrolidine-2,5-dione |
| F3325-0153 | 1-(2,4-dimethylphenyl)-3-(4-phenylpiperazin-1-yl)pyrrolidine-2,5-dione |
| F6401-0048 | 1-[2-(2,3-dihydro-1H-indol-1-yl)-2-oxoethyl]-4-(thiophen-2-yl)piperidine-2,6-dione |
| F3325-0108 | 3-[4-(4-fluorophenyl)piperazin-1-yl]-1-(2-methylphenyl)pyrrolidine-2,5-dione |
| F5065-0059 | N-(3,4-dichlorophenyl)-4-{3-ethyl-3H-[1,2,3]triazolo[4,5-d]pyrimidin-7-yl}piperazine-1-carboxamide |
| F1318-0212 | 3-nitrophenyl piperidine-1-carboxylate |
| F3385-3831 | 7-[2-(morpholin-4-yl)ethoxy]-3-phenyl-4H-chromen-4-one |
| F0266-0124 | butyl 4-hydroxybenzoate |
| F0440-0329 | 2-(2,5-dioxopyrrolidin-1-yl)-N-(6-fluoro-1,3-benzothiazol-2-yl)acetamide |
| F3205-0034 | 1-(3-methoxypropyl)-3-{[4-(morpholin-4-yl)phenyl]amino}pyrrolidine-2,5-dione |
| F5577-0089 | 1-ethyl-6-fluoro-7-(morpholin-4-yl)-4-oxo-1,4-dihydroquinoline-3-carboxylic acid |
| F1318-0211 | 3-nitrophenyl morpholine-4-carboxylate |
| F2191-0037 | 4-phenoxybenzoic acid |
| F3230-0012 | 3-(4-chloro-3,5-dimethyl-1H-pyrazol-1-yl)-1-[4-(4-methylbenzenesulfonyl)piperazin-1-yl]butan-1-one |
| F0440-0330 | 2-(2,5-dioxopyrrolidin-1-yl)-N-{4H,5H-naphtho[1,2-d][1,3]thiazol-2-yl}acetamide |
| F0440-0328 | N-(4,6-dimethyl-1,3-benzothiazol-2-yl)-2-(2,5-dioxopyrrolidin-1-yl)acetamide |
| F2190-0411 | N-phenylaniline |
| F3325-0130 | 1-butyl-3-{[4-(dimethylamino)phenyl]amino}pyrrolidine-2,5-dione |
| F1813-0844 | N-(4,7-dimethyl-1,3-benzothiazol-2-yl)-2-(2,5-dioxopyrrolidin-1-yl)acetamide |
| F3115-0098 | ethyl 4-[1-(2-ethylphenyl)-2,5-dioxopyrrolidin-3-yl]piperazine-1-carboxylate |
| F1142-6543 | 1-(3,4,5-trimethoxybenzoyl)azepan-2-one |
| F2183-0479 | 6-fluoro-4-oxo-1-propyl-1,4-dihydroquinoline-3-carboxylic acid |
| F0412-0066 | N-[2-chloro-5-(trifluoromethyl)phenyl]-4-(2,5-dioxopyrrolidin-1-yl)benzamide |
| F1800-0007 | ethyl 4-[2-(3,5-dioxothiomorpholin-4-yl)acetamido]benzoate |
| F0907-0036 | ethyl 1-ethyl-6-fluoro-4-oxo-7-(pyrrolidin-1-yl)-1,4-dihydroquinoline-3-carboxylate |
| F0466-0057 | N-(3-cyano-4,5,6,7-tetrahydro-1-benzothiophen-2-yl)-3-(2,5-dioxopyrrolidin-1-yl)benzamide |
| F0550-0115 | N-(2-chloro-4-nitrophenyl)-5-oxo-2H,3H,5H-[1,3]thiazolo[3,2-a]pyrimidine-6-carboxamide |
| F0550-0116 | N-(4-chloro-2-nitrophenyl)-5-oxo-2H,3H,5H-[1,3]thiazolo[3,2-a]pyrimidine-6-carboxamide |
| F0440-0372 | 2-(2,5-dioxopyrrolidin-1-yl)-N-(4-methoxy-2-nitrophenyl)acetamide |
| F0440-0322 | N-(1,3-benzothiazol-2-yl)-2-(2,5-dioxopyrrolidin-1-yl)acetamide |
| F1813-0847 | N-(5,7-dimethyl-1,3-benzothiazol-2-yl)-2-(2,5-dioxopyrrolidin-1-yl)acetamide |
| F2880-0243 | 2-chloro-N-[2-(dimethylamino)-2-(thiophen-2-yl)ethyl]-4-nitrobenzamide |
| F1318-0213 | 3-nitrophenyl N,N-dimethylcarbamate |
| F3023-0908 | 3-(4-benzylpiperazin-1-yl)-1-(2-methylpropyl)pyrrolidine-2,5-dione |
| F5893-0231 | 2-(2,5-dioxopyrrolidin-1-yl)-N-(2-methoxyquinolin-8-yl)acetamide |
| F3325-0099 | 3-[4-(4-fluorophenyl)piperazin-1-yl]-1-(3-methylphenyl)pyrrolidine-2,5-dione |
| F1813-1253 | N-(4,6-difluoro-1,3-benzothiazol-2-yl)-2-(2,5-dioxopyrrolidin-1-yl)acetamide |
| F3205-0043 | 3-[(4-ethylphenyl)amino]-1-(prop-2-en-1-yl)pyrrolidine-2,5-dione |
| F3395-0160 | (naphthalen-2-yl)methanol |
| F1813-0840 | N-(6-chloro-4-methyl-1,3-benzothiazol-2-yl)-2-(2,5-dioxopyrrolidin-1-yl)acetamide |
| F1813-0839 | N-(5-chloro-4-methyl-1,3-benzothiazol-2-yl)-2-(2,5-dioxopyrrolidin-1-yl)acetamide |
| F0327-0394 | N-{3-cyano-4H,5H,6H-cyclopenta[b]thiophen-2-yl}-4-(2,5-dioxopyrrolidin-1-yl)benzamide |
| F0466-0060 | N-{3-cyano-4H,5H,6H,7H,8H-cyclohepta[b]thiophen-2-yl}-3-(2,5-dioxopyrrolidin-1-yl)benzamide |
| F0017-0155 | 1-(1H-1,3-benzodiazol-2-ylsulfanyl)-3,3-dimethylbutan-2-one; 2,4,6-trinitrophenol |
| F2266-0069 | 3-chloro-N-(1-ethyl-2-oxo-1,2,3,4-tetrahydroquinolin-6-yl)benzamide |
| F0327-0365 | N-(3,5-dichlorophenyl)-4-(2,5-dioxopyrrolidin-1-yl)benzamide |
| F0440-0318 | N-(6-chloro-1,3-benzothiazol-2-yl)-2-(2,5-dioxopyrrolidin-1-yl)acetamide |
| F2158-0861 | 4-[(diethylcarbamoyl)oxy]benzoic acid |
| F3083-0086 | 1-[4-(benzenesulfonyl)piperazin-1-yl]-3-(4-bromo-3,5-dimethyl-1H-pyrazol-1-yl)butan-1-one |
| F2995-0094 | N-(2-cyanophenyl)-2-(4-methylphenyl)acetamide |
| F0327-0355 | N-[4-(5-bromothiophen-2-yl)-1,3-thiazol-2-yl]-4-(2,5-dioxopyrrolidin-1-yl)benzamide |
| F0440-0374 | 2-(2,5-dioxopyrrolidin-1-yl)-N-(2-methoxy-4-nitrophenyl)acetamide |
| F3023-0961 | 3-{[4-(morpholin-4-yl)phenyl]amino}-1-[3-(propan-2-yloxy)propyl]pyrrolidine-2,5-dione |
| F6617-3965 | 2-[(2E)-3-(4-methoxyphenyl)prop-2-enamido]acetic acid |
| F3205-0035 | 1-(3-methoxypropyl)-3-{[4-(piperidin-1-yl)phenyl]amino}pyrrolidine-2,5-dione |
| F2590-0186 | 2-[3-(2,5-dioxopyrrolidin-1-yl)benzamido]-4,5-dimethylthiophene-3-carboxamide |
| F2266-0010 | 4-chloro-N-(1-ethyl-2-oxo-1,2,3,4-tetrahydroquinolin-6-yl)benzamide |
| F3115-0084 | ethyl 4-[1-(4-ethylphenyl)-2,5-dioxopyrrolidin-3-yl]piperazine-1-carboxylate |
| F3325-0127 | 3-(4-benzylpiperazin-1-yl)-1-(4-methylphenyl)pyrrolidine-2,5-dione |
| F0700-0157 | N-[4-(5-bromothiophen-2-yl)-1,3-thiazol-2-yl]-3-(2,5-dioxopyrrolidin-1-yl)benzamide |
| F0849-3787 | 3-[4-(4-fluorophenyl)piperazin-1-yl]-1-(2-methoxyphenyl)pyrrolidine-2,5-dione |
| F1813-0843 | N-(7-chloro-4-methyl-1,3-benzothiazol-2-yl)-2-(2,5-dioxopyrrolidin-1-yl)acetamide |
| F2769-0049 | N-(1-acetyl-1,2,3,4-tetrahydroquinolin-6-yl)-4-chlorobenzamide |
| F0327-0357 | N-(5-acetyl-4-methyl-1,3-thiazol-2-yl)-4-(2,5-dioxopyrrolidin-1-yl)benzamide |
| F2769-0037 | N-(1-acetyl-1,2,3,4-tetrahydroquinolin-6-yl)-3-chlorobenzamide |
| F1105-0086 | 3-(2,5-dioxopyrrolidin-1-yl)-N-(2-methyl-1,3-dioxo-2,3-dihydro-1H-isoindol-4-yl)benzamide |
| F3083-0082 | 3-(4-bromo-3,5-dimethyl-1H-pyrazol-1-yl)-1-[4-(4-methylbenzenesulfonyl)piperazin-1-yl]propan-1-one |
| F1800-0036 | N-(4-butylphenyl)-2-(3,5-dioxothiomorpholin-4-yl)acetamide |
| F3325-0112 | 3-[4-(4-fluorophenyl)piperazin-1-yl]-1-(3-methoxyphenyl)pyrrolidine-2,5-dione |
| F1822-0643 | 2-(2,5-dioxopyrrolidin-1-yl)-N-(4-ethyl-1,3-benzothiazol-2-yl)acetamide |
| F1822-0644 | N-{4,6-dioxa-10-thia-12-azatricyclo[7.3.0.0^{3,7}]dodeca-1(9),2,7,11-tetraen-11-yl}-2-(2,5-dioxopyrrolidin-1-yl)acetamide |
| F2670-0050 | N-(1-acetyl-1,2,3,4-tetrahydroquinolin-7-yl)-3-methylbenzamide |
| F5882-5135 | 2-(2,5-dioxopyrrolidin-1-yl)-N-[4-(3-methyl-1,2,4-oxadiazol-5-yl)phenyl]acetamide |
| F0327-0381 | 4-(2,5-dioxopyrrolidin-1-yl)-N-(2-methyl-1,3-dioxo-2,3-dihydro-1H-isoindol-5-yl)benzamide |
| F5577-0081 | 1-ethyl-6-fluoro-7-(4-methylpiperidin-1-yl)-4-oxo-1,4-dihydroquinoline-3-carboxylic acid |
| F3385-0896 | 2-{[3-(4-methoxyphenyl)-4-oxo-4H-chromen-7-yl]oxy}acetonitrile |
| F1800-0195 | ethyl 4-[2-(2-methyl-3,5-dioxothiomorpholin-4-yl)acetamido]benzoate |
| F3385-4482 | 5-ethoxy-7-hydroxy-2-phenyl-4H-chromen-4-one |
| F3182-0011 | 11-(2,5-dioxopyrrolidin-1-yl)undecanoic acid |
| F5123-0411 | N-(3,4-dichlorophenyl)-4-{3-ethyl-[1,2,4]triazolo[4,3-b]pyridazin-6-yl}piperazine-1-carboxamide |
| F0440-0332 | 2-(2,5-dioxopyrrolidin-1-yl)-N-[4-(4-methylphenyl)-1,3-thiazol-2-yl]acetamide |
| F0873-0006 | 2-[(4-nitrophenyl)formamido]acetic acid |
| F2158-0864 | 1-[3-(4-chlorophenyl)-1,2,4-oxadiazol-5-yl]propan-2-one |
| F2313-0092 | 2-(2,5-dioxopyrrolidin-1-yl)-N-{5-[3-(propan-2-ylsulfanyl)phenyl]-1,3,4-oxadiazol-2-yl}acetamide |
| F0440-0315 | 2-(2,5-dioxopyrrolidin-1-yl)-N-(6-methoxy-1,3-benzothiazol-2-yl)acetamide |
| F0440-0331 | 2-(2,5-dioxopyrrolidin-1-yl)-N-(4-phenyl-1,3-thiazol-2-yl)acetamide |
| F1916-0010 | 3-{4-[4-(2-carboxyethyl)phenoxy]phenyl}propanoic acid |
| F2073-0219 | 2-chloro-N-{2,6-dimethyl-4-oxo-4H-pyrido[1,2-a]pyrimidin-3-yl}-4-nitrobenzamide |
| F1813-0845 | 2-(2,5-dioxopyrrolidin-1-yl)-N-(4-methoxy-7-methyl-1,3-benzothiazol-2-yl)acetamide |
| F0349-4092 | 4-oxo-4-(2,4,5-trimethoxyphenyl)butanoic acid |
| F0440-0319 | N-(6-bromo-1,3-benzothiazol-2-yl)-2-(2,5-dioxopyrrolidin-1-yl)acetamide |
| F0440-0338 | N-[4-(2,5-dimethylphenyl)-1,3-thiazol-2-yl]-2-(2,5-dioxopyrrolidin-1-yl)acetamide |
| F0440-0347 | 2-(2,5-dioxopyrrolidin-1-yl)-N-[4-(4-fluorophenyl)-1,3-thiazol-2-yl]acetamide |
| F0440-0337 | N-[4-(2,4-dimethylphenyl)-1,3-thiazol-2-yl]-2-(2,5-dioxopyrrolidin-1-yl)acetamide |
| F6443-5308 | N-(3,4-dichlorophenyl)-3-oxo-3H-spiro[2-benzofuran-1,3'-piperidine]-1'-carboxamide |
| F0608-0631 | 2-(2,5-dioxopyrrolidin-1-yl)-N-[5-(5,6,7,8-tetrahydronaphthalen-2-yl)-1,3,4-oxadiazol-2-yl]acetamide |
| F5889-0027 | N-[2-(morpholin-4-yl)-2-oxoethyl]-1H-indole-3-carboxamide |
| F6369-0505 | 1-[4-(trifluoromethyl)-1,3-benzothiazol-2-yl]azetidin-3-yl 2-(2,5-dioxopyrrolidin-1-yl)acetate |
| F0440-0339 | N-[4-(3,4-dimethylphenyl)-1,3-thiazol-2-yl]-2-(2,5-dioxopyrrolidin-1-yl)acetamide |
| F2518-0396 | N-[5-(2,5-dimethylphenyl)-1,3,4-oxadiazol-2-yl]-2-(2,5-dioxopyrrolidin-1-yl)acetamide |
| F3385-0894 | 3-(3,4-dimethoxyphenyl)-7-methoxy-4H-chromen-4-one |
| F0466-0033 | N-(5-acetyl-4-methyl-1,3-thiazol-2-yl)-3-(2,5-dioxopyrrolidin-1-yl)benzamide |
| F2073-0017 | 2-chloro-N-{2-methyl-4-oxo-4H-pyrido[1,2-a]pyrimidin-3-yl}-4-nitrobenzamide |
| F3228-0188 | 5,7-dimethoxy-3-(2-methoxyphenyl)-4H-chromen-4-one |
| F0789-0023 | N-(5,5-dimethyl-7-oxo-4,5,6,7-tetrahydro-1,3-benzothiazol-2-yl)-3-(2,5-dioxopyrrolidin-1-yl)benzamide |
| F6401-0075 | benzyl 2-[2,6-dioxo-4-(thiophen-2-yl)piperidin-1-yl]acetate |
| F0700-0132 | 2-(2,5-dioxopyrrolidin-1-yl)-N-[2-(methylsulfanyl)-1,3-benzothiazol-6-yl]acetamide |
| F2191-0001 | 1-hydroxynaphthalene-2-carboxylic acid |
| F3205-0012 | 1-[4-(morpholin-4-yl)phenyl]-3-[2-(piperidin-1-yl)cyclohex-2-en-1-yl]pyrrolidine-2,5-dione |
| F3083-0091 | 3-(4-bromo-3,5-dimethyl-1H-pyrazol-1-yl)-1-[4-(4-methylbenzenesulfonyl)piperazin-1-yl]butan-1-one |
| F3325-0150 | 1-(2-ethoxyphenyl)-3-(4-phenylpiperazin-1-yl)pyrrolidine-2,5-dione |
| F3083-0081 | 3-(4-bromo-3,5-dimethyl-1H-pyrazol-1-yl)-1-[4-(4-chlorobenzenesulfonyl)piperazin-1-yl]propan-1-one |
| F0327-0396 | 2-[4-(2,5-dioxopyrrolidin-1-yl)benzamido]-5-methyl-4,5,6,7-tetrahydro-1-benzothiophene-3-carboxamide |
| F1374-2249 | 2-(2,5-dioxopyrrolidin-1-yl)-N-{5-[4-(trifluoromethyl)phenyl]-1,3,4-oxadiazol-2-yl}acetamide |
| F0327-0306 | N-(5,5-dimethyl-7-oxo-4,5,6,7-tetrahydro-1,3-benzothiazol-2-yl)-4-(2,5-dioxopyrrolidin-1-yl)benzamide |
| F1130-0015 | 2-{[3-(4-methylphenyl)adamantan-1-yl]formamido}acetic acid |
| F2518-0252 | N-[5-(2,4-dimethylphenyl)-1,3,4-oxadiazol-2-yl]-2-(2,5-dioxopyrrolidin-1-yl)acetamide |
| F3115-0085 | ethyl 4-{1-[4-(morpholin-4-yl)phenyl]-2,5-dioxopyrrolidin-3-yl}piperazine-1-carboxylate |
| F0440-0334 | 2-(2,5-dioxopyrrolidin-1-yl)-N-[4-(4-methoxyphenyl)-1,3-thiazol-2-yl]acetamide |
| F0440-0346 | 2-(2,5-dioxopyrrolidin-1-yl)-N-[4-(2-methoxyphenyl)-1,3-thiazol-2-yl]acetamide |
| F1813-0838 | N-(4,5-dichloro-1,3-benzothiazol-2-yl)-2-(2,5-dioxopyrrolidin-1-yl)acetamide |
| F3385-0975 | 2-{[3-(3,4-dimethoxyphenyl)-4-oxo-4H-chromen-7-yl]oxy}acetamide |
| F2670-0026 | N-(1-acetyl-1,2,3,4-tetrahydroquinolin-7-yl)-3-chlorobenzamide |
| F0327-0391 | 2-[4-(2,5-dioxopyrrolidin-1-yl)benzamido]-4,5,6,7-tetrahydro-1-benzothiophene-3-carboxamide |
| F0440-0317 | 2-(2,5-dioxopyrrolidin-1-yl)-N-(6-ethoxy-1,3-benzothiazol-2-yl)acetamide |
| F2670-0038 | N-(1-acetyl-1,2,3,4-tetrahydroquinolin-7-yl)-4-chlorobenzamide |
| F3228-0184 | 5,7-dimethoxy-2-phenyl-4H-chromen-4-one |
| F1105-0061 | N-(2-methyl-1,3-dioxo-2,3-dihydro-1H-isoindol-4-yl)-3-(trifluoromethyl)benzamide |
| F3325-0128 | 3-(4-benzylpiperazin-1-yl)-1-(2-methoxyphenyl)pyrrolidine-2,5-dione |
| F3323-0631 | 4-[2-(3-fluoro-4-methoxyphenyl)-2-oxoethyl]-2,3,4,5-tetrahydro-1,4-benzoxazepine-3,5-dione |
| F1813-0842 | N-(4,7-dichloro-1,3-benzothiazol-2-yl)-2-(2,5-dioxopyrrolidin-1-yl)acetamide |
| F3323-0583 | 4-[2-(2-methoxyphenyl)-2-oxoethyl]-2,3,4,5-tetrahydro-1,4-benzoxazepine-3,5-dione |
| F0349-0111 | ethyl N-{2-[(ethoxycarbonyl)amino]phenyl}carbamate |
| F3188-0006 | ethyl 4-[1-(2,5-dimethoxyphenyl)-2,5-dioxopyrrolidin-3-yl]piperazine-1-carboxylate |
| F6250-0491 | 3-[2-(2,5-dioxopyrrolidin-1-yl)acetamido]cyclohexyl N-(4-fluorophenyl)carbamate |
| F0806-0077 | methyl 3-[2-(2,5-dioxopyrrolidin-1-yl)acetamido]-1-benzothiophene-2-carboxylate |
| F0806-0417 | 2-[3-(2,5-dioxopyrrolidin-1-yl)benzamido]-4H,5H,6H,7H,8H-cyclohepta[b]thiophene-3-carboxamide |
| F0777-0033 | 2-(dibenzylamino)acetic acid |
| F2590-1466 | methyl 2-[4-(2,5-dioxopyrrolidin-1-yl)benzamido]-4,5-dimethylthiophene-3-carboxylate |
| F3188-0014 | ethyl 4-{1-[4-(difluoromethoxy)phenyl]-2,5-dioxopyrrolidin-3-yl}piperazine-1-carboxylate |
| F3385-2703 | 2-{[3-(3,4-dimethoxyphenyl)-4-oxo-4H-chromen-7-yl]oxy}acetonitrile |
| F0415-0017 | 2-(pyridin-4-yl)-4H-chromen-4-one |
| F0196-0437 | 3-ethyl-4-(7-hydroxy-2-methyl-4-oxo-4H-chromen-3-yl)-2-methyl-1,3-thiazol-3-ium 4-methylbenzene-1-sulfonate |
| F3095-1901 | 2-{[1,1'-biphenyl]-4-yloxy}-N-[(oxolan-2-yl)methyl]acetamide |
| F0440-0333 | 2-(2,5-dioxopyrrolidin-1-yl)-N-[4-(4-ethylphenyl)-1,3-thiazol-2-yl]acetamide |
| F6441-6721 | N-(3,4-dichlorophenyl)-4-(4-methoxybenzenesulfonyl)piperidine-1-carboxamide |
| F0286-0220 | 2-chloro-4-nitro-N-(1,3-thiazol-2-yl)benzamide |
| F2018-0987 | N-[2-(dimethylamino)ethyl]-2-(2,5-dioxopyrrolidin-1-yl)-N-(4-methyl-1,3-benzothiazol-2-yl)acetamide hydrochloride |
| F1300-0076 | methyl 2-[2-(2,5-dioxopyrrolidin-1-yl)acetamido]-1,3-benzothiazole-6-carboxylate |
| F1358-0042 | methyl 2-[4-(2,5-dioxopyrrolidin-1-yl)benzamido]thiophene-3-carboxylate |
| F1166-0209 | 2-(2,5-dioxopyrrolidin-1-yl)-N-[4-(5,6,7,8-tetrahydronaphthalen-2-yl)-1,3-thiazol-2-yl]acetamide |
| F1813-0846 | N-(4,7-dimethoxy-1,3-benzothiazol-2-yl)-2-(2,5-dioxopyrrolidin-1-yl)acetamide |
| F2898-5747 | 2-(2,5-dioxopyrrolidin-1-yl)-N-[(oxolan-2-yl)methyl]-N-[4-(trifluoromethyl)-1,3-benzothiazol-2-yl]acetamide |
| F2049-0468 | N-(1-benzoyl-1,2,3,4-tetrahydroquinolin-7-yl)acetamide |
| F3188-0005 | ethyl 4-[1-(2,4-dimethoxyphenyl)-2,5-dioxopyrrolidin-3-yl]piperazine-1-carboxylate |
| F2073-0118 | 2-chloro-N-{7-chloro-2-methyl-4-oxo-4H-pyrido[1,2-a]pyrimidin-3-yl}-4-nitrobenzamide |
| F2517-1310 | 2-(2,5-dioxopyrrolidin-1-yl)-N-{5-[4-(trifluoromethoxy)phenyl]-1,3,4-oxadiazol-2-yl}acetamide |
| F1358-0173 | ethyl 2-[4-(2,5-dioxopyrrolidin-1-yl)benzamido]thiophene-3-carboxylate |
| F3316-0247 | N-[2-(4-chloro-3,5-dimethyl-1H-pyrazol-1-yl)ethyl]-3-nitrobenzamide |
| F3095-3637 | 2-({4'-methyl-[1,1'-biphenyl]-4-yl}oxy)-1-(morpholin-4-yl)ethan-1-one |
| F5228-0080 | N-(5-chloro-2-hydroxyphenyl)-2-(6-oxo-4-phenyl-1,6-dihydropyrimidin-1-yl)acetamide |
| F3385-4187 | 3-(4-methoxyphenyl)-8-methyl-7-[2-(morpholin-4-yl)ethoxy]-4H-chromen-4-one |
| F5689-0059 | 4-(2,5-dioxopyrrolidin-1-yl)-N-[2-(4-methyl-5-oxo-3-phenyl-4,5-dihydro-1H-1,2,4-triazol-1-yl)ethyl]benzamide |
| F1757-0012 | 1-{4-[3-(4-bromo-3,5-dimethyl-1H-pyrazol-1-yl)-2-hydroxypropoxy]-3-methoxyphenyl}ethan-1-one |
| F2518-0108 | N-[5-(2,5-dichlorophenyl)-1,3,4-oxadiazol-2-yl]-2-(2,5-dioxopyrrolidin-1-yl)acetamide |
| F3385-0895 | 3-(3,4-dimethoxyphenyl)-7-ethoxy-4H-chromen-4-one |
| F6435-1028 | 3-(benzenesulfonyl)-N-(3,4-dichlorophenyl)pyrrolidine-1-carboxamide |
| F1757-0120 | 1-{4-[3-(4-bromo-3,5-dimethyl-1H-pyrazol-1-yl)-2-hydroxypropoxy]phenyl}propan-1-one |
| F1591-0157 | 4-amino-N-methylbenzene-1-sulfonamide |
| F2493-3641 | N-{[5-(2,3-dihydro-1,4-benzodioxin-6-yl)-1,2-oxazol-3-yl]methyl}-2-(2,5-dioxopyrrolidin-1-yl)acetamide |
| F3019-0029 | 3-[(adamantan-1-yl)amino]-1-[4-(dimethylamino)phenyl]pyrrolidine-2,5-dione |
| F0440-0345 | 2-(2,5-dioxopyrrolidin-1-yl)-N-{4-[4-(propan-2-yl)phenyl]-1,3-thiazol-2-yl}acetamide |
| F1589-0514 | 4-[5-(2-chloro-4-nitrophenyl)furan-2-carbonyl]morpholine |
| F3188-0015 | ethyl 4-(1-{4-[(difluoromethyl)sulfanyl]phenyl}-2,5-dioxopyrrolidin-3-yl)piperazine-1-carboxylate |
| F3228-0172 | 2-(4-chlorophenyl)-7-hydroxy-4H-chromen-4-one |
| F3229-0031 | 2-(3-ethyl-2,6-dioxo-3-phenylpiperidin-1-yl)-N-(2,4,6-trimethylphenyl)acetamide |
| F6195-2940 | N-{5-[(3,4-dichlorophenyl)carbamoyl]-4H,5H,6H,7H-[1,3]thiazolo[5,4-c]pyridin-2-yl}pyrazine-2-carboxamide |
| F1589-0509 | 5-(2-chloro-4-nitrophenyl)-N-(propan-2-yl)furan-2-carboxamide |
| F6439-8110 | 2-(2,5-dioxopyrrolidin-1-yl)-N-{2-[6-(furan-2-yl)-1H-pyrazolo[1,5-a]imidazol-1-yl]ethyl}acetamide |
| F1751-0007 | 3-[(adamantan-1-yl)amino]-1-(4-methoxyphenyl)pyrrolidine-2,5-dione |
| F6473-3975 | 1-[1-({3',4'-dichloro-[1,1'-biphenyl]-4-yl}sulfonyl)azetidin-3-yl]pyrrolidine-2,5-dione |
| F1084-0888 | 2-chloro-4-nitro-N-(pyridin-2-yl)benzamide |
| F0526-0592 | methyl 2-[4-(2,5-dioxopyrrolidin-1-yl)benzamido]-6-(propan-2-yl)-4H,5H,6H,7H-thieno[2,3-c]pyridine-3-carboxylate hydrochloride |
| F5940-1789 | N-(3,4-dichlorophenyl)-4-[(prop-2-yn-1-yloxy)methyl]piperidine-1-carboxamide |
| F3323-0578 | 4-[2-(5-chloro-2-methoxyphenyl)-2-oxoethyl]-2,3,4,5-tetrahydro-1,4-benzoxazepine-3,5-dione |
| F6195-1924 | N-(3,4-dichlorophenyl)-2-(furan-2-amido)-4H,5H,6H,7H-[1,3]thiazolo[5,4-c]pyridine-5-carboxamide |
| F1300-0180 | ethyl 2-[2-(2,5-dioxopyrrolidin-1-yl)acetamido]-1,3-benzothiazole-6-carboxylate |
| F6663-4473 | (2E)-3-(2H-1,3-benzodioxol-5-yl)-1-(4,4-difluoropiperidin-1-yl)prop-2-en-1-one |
| F3110-2246 | naphthalene-1-carboxylic acid |
| F3323-0632 | 4-[2-(2-ethoxy-5-fluorophenyl)-2-oxoethyl]-2,3,4,5-tetrahydro-1,4-benzoxazepine-3,5-dione |
| F2019-0479 | N-[3-(dimethylamino)propyl]-2-(2,5-dioxopyrrolidin-1-yl)-N-(6-fluoro-1,3-benzothiazol-2-yl)acetamide hydrochloride |
| F2019-1368 | N-[3-(dimethylamino)propyl]-2-(2,5-dioxopyrrolidin-1-yl)-N-(4-fluoro-1,3-benzothiazol-2-yl)acetamide hydrochloride |
| F2496-3074 | [5-(2-fluorophenyl)-1,2-oxazol-3-yl]methyl 2-(2,5-dioxopyrrolidin-1-yl)acetate |
| F3375-0129 | 5-(2-chloro-4-nitrophenyl)-N-(1,1-dioxo-1lambda6-thiolan-3-yl)furan-2-carboxamide |
| F0526-0593 | methyl 2-[3-(2,5-dioxopyrrolidin-1-yl)benzamido]-6-ethyl-4H,5H,6H,7H-thieno[2,3-c]pyridine-3-carboxylate hydrochloride |
| F6170-0024 | 3-ethyl 5-methyl 2-[(2-aminoethoxy)methyl]-4-(2-chlorophenyl)-6-methyl-1,4-dihydropyridine-3,5-dicarboxylate; benzenesulfonic acid |
| F0440-0349 | N-[4-(3,4-dichlorophenyl)-1,3-thiazol-2-yl]-2-(2,5-dioxopyrrolidin-1-yl)acetamide |
| F5831-4222 | N-({1,4-dioxaspiro[4.5]decan-2-yl}methyl)-2-(2,5-dioxopyrrolidin-1-yl)acetamide |
| F1383-0018 | 2-[(1-ethyl-2,5-dioxopyrrolidin-3-yl)sulfanyl]pyridine-3-carboxylic acid |
| F0440-0335 | N-[4-(4-bromophenyl)-1,3-thiazol-2-yl]-2-(2,5-dioxopyrrolidin-1-yl)acetamide |
| F3385-4481 | ethyl 2-[(7-hydroxy-4-oxo-2-phenyl-4H-chromen-5-yl)oxy]acetate |
| F0440-0343 | N-[4-(2,5-dichlorophenyl)-1,3-thiazol-2-yl]-2-(2,5-dioxopyrrolidin-1-yl)acetamide |
| F3349-0203 | 4-(2,5-dioxopyrrolidin-1-yl)-N-(2-methylphenyl)benzene-1-sulfonamide |
| F3310-1337 | 3-(benzyloxy)benzoic acid |
| F0882-0814 | ethyl 5-[4-(2,5-dioxopyrrolidin-1-yl)benzamido]-3-methylthiophene-2-carboxylate |
| F0440-0344 | N-[4-(3,4-dimethoxyphenyl)-1,3-thiazol-2-yl]-2-(2,5-dioxopyrrolidin-1-yl)acetamide |
| F0715-0438 | N-[4-(2,4-dimethoxyphenyl)-1,3-thiazol-2-yl]-2-(2,5-dioxopyrrolidin-1-yl)acetamide |
| F5526-0468 | N-[2-(4-ethoxyphenoxy)ethyl]-4-methoxybenzamide |
| F6195-0976 | N-{5-[2-(2,6-dioxopiperidin-1-yl)ethanesulfonyl]-4H,5H,6H,7H-[1,3]thiazolo[5,4-c]pyridin-2-yl}cyclopropanecarboxamide |
| F3095-0745 | 1-(4-bromobenzoyl)-1,2,3,4-tetrahydroquinoline |
| F2040-0434 | 4-chloro-N-(1-propanoyl-1,2,3,4-tetrahydroquinolin-6-yl)benzamide |
| F0882-0957 | N-[4-(2,4-dichlorophenyl)-1,3-thiazol-2-yl]-2-(2,5-dioxopyrrolidin-1-yl)acetamide |
| F3350-0562 | 4-(2,5-dioxopyrrolidin-1-yl)-N-(4-methylphenyl)benzene-1-sulfonamide |
| F2019-2765 | N-(4,6-difluoro-1,3-benzothiazol-2-yl)-N-[3-(dimethylamino)propyl]-2-(2,5-dioxopyrrolidin-1-yl)acetamide hydrochloride |
| F2496-3406 | [5-(2,3-dihydro-1,4-benzodioxin-6-yl)-1,2-oxazol-3-yl]methyl 2-(2,5-dioxopyrrolidin-1-yl)acetate |
| F3318-0187 | 1-(4-chlorobenzoyl)-2-methyl-2,3-dihydro-1H-indole |
| F0739-0015 | 2-({5,6-dimethyl-4-oxo-3-phenyl-3H,4H-thieno[2,3-d]pyrimidin-2-yl}sulfanyl)-N-(2-hydroxyethyl)acetamide |
| F0866-0212 | ethyl 2-[4-(2,5-dioxopyrrolidin-1-yl)benzamido]-4,5-dimethylthiophene-3-carboxylate |
| F2416-0104 | N-(2-acetyl-1,2,3,4-tetrahydroisoquinolin-7-yl)-2-(2,4-dichlorophenoxy)acetamide |
| F3230-0005 | 4-chlorophenyl 2-(4-chloro-3,5-dimethyl-1H-pyrazol-1-yl)acetate |
| F3205-0038 | 2-[(1-cyclopentyl-2,5-dioxopyrrolidin-3-yl)sulfanyl]pyridine-3-carboxylic acid |
| F0920-6579 | 6-methoxy-9-methyl-8-oxa-10,12-diazatricyclo[7.3.1.0^{2,7}]trideca-2,4,6-trien-11-one |
| F3367-0020 | 4-[(2,5-dioxopyrrolidin-1-yl)methyl]-N-phenylbenzene-1-sulfonamide |
| F2590-0321 | 2-[3-(2,5-dioxopyrrolidin-1-yl)benzamido]-N,4,5-trimethylthiophene-3-carboxamide |
| F3019-0032 | 3-[(adamantan-1-yl)amino]-1-(2,4-dimethoxyphenyl)pyrrolidine-2,5-dione |
| F3350-0581 | 1-[4-(2,3-dihydro-1H-indole-1-sulfonyl)phenyl]pyrrolidine-2,5-dione |
| F2019-0606 | N-(6-chloro-1,3-benzothiazol-2-yl)-N-[3-(dimethylamino)propyl]-2-(2,5-dioxopyrrolidin-1-yl)acetamide hydrochloride |
| F2019-1495 | N-(4-chloro-1,3-benzothiazol-2-yl)-N-[3-(dimethylamino)propyl]-2-(2,5-dioxopyrrolidin-1-yl)acetamide hydrochloride |
| F2604-0411 | 3-[4-(2-chloro-4-nitrobenzoyl)piperazin-1-yl]-6-(piperidin-1-yl)pyridazine |
| F0807-0474 | 2-{[4,6-bis(ethylamino)-1,3,5-triazin-2-yl]sulfanyl}-1-phenylethan-1-one |
| F6660-2186 | 7-[4-(2-cyanoacetyl)piperazin-1-yl]-1-ethyl-6-fluoro-4-oxo-1,4-dihydroquinoline-3-carboxylic acid |
| F3350-0589 | 4-(2,5-dioxopyrrolidin-1-yl)-N-(2-fluorophenyl)benzene-1-sulfonamide |
| F6660-2932 | 7-[4-(2-cyanoacetyl)piperazin-1-yl]-1-ethyl-6-fluoro-4-oxo-1,4-dihydroquinoline-3-carboxylic acid |
| F0327-0351 | ethyl 2-[4-(2,5-dioxopyrrolidin-1-yl)benzamido]-4-methyl-1,3-thiazole-5-carboxylate |
| F1383-0047 | 2-{[2,5-dioxo-1-(propan-2-yl)pyrrolidin-3-yl]sulfanyl}pyridine-3-carboxylic acid |
| F2604-0480 | 1-{6-[4-(2-chloro-4-nitrobenzoyl)piperazin-1-yl]pyridazin-3-yl}azepane |
| F5689-1366 | 4-(2,5-dioxopyrrolidin-1-yl)-N-{2-[4-methyl-5-oxo-3-(thiophen-2-yl)-4,5-dihydro-1H-1,2,4-triazol-1-yl]ethyl}benzamide |
| F1018-1721 | 1-(4-chloro-2-nitrobenzoyl)-3,5-dimethyl-1H-pyrazole |
| F3349-0202 | 4-(2,5-dioxopyrrolidin-1-yl)-N-phenylbenzene-1-sulfonamide |
| F3275-0038 | 1-(4-bromo-3,5-dimethyl-1H-pyrazol-1-yl)-3-[5-methyl-2-(propan-2-yl)phenoxy]propan-2-ol |
| F1383-0046 | 2-[(1-methyl-2,5-dioxopyrrolidin-3-yl)sulfanyl]pyridine-3-carboxylic acid |
| F2693-0113 | 4-chloro-9-methyl-8-oxa-10,12-diazatricyclo[7.3.1.0^{2,7}]trideca-2,4,6-trien-11-one |
| F6660-2871 | 7-[4-(2-cyanoacetyl)piperazin-1-yl]-1-cyclopropyl-6-fluoro-4-oxo-1,4-dihydroquinoline-3-carboxylic acid |
| F0327-0393 | ethyl 2-[4-(2,5-dioxopyrrolidin-1-yl)benzamido]-4H,5H,6H-cyclopenta[b]thiophene-3-carboxylate |
| F0838-0491 | 1,4-bis(2-fluorobenzoyl)-2-methylpiperazine |
| F3385-4499 | 3-(3,4-dimethoxyphenyl)-7-[2-(4-ethylpiperazin-1-yl)ethoxy]-4H-chromen-4-one dihydrochloride |
| F1829-0066 | 2-[3-(2,5-dioxopyrrolidin-1-yl)benzamido]-N,6-dimethyl-4,5,6,7-tetrahydro-1-benzothiophene-3-carboxamide |
| F1412-0027 | 2-[(1-cyclohexyl-2,5-dioxopyrrolidin-3-yl)sulfanyl]pyridine-3-carboxylic acid |
| F3342-0215 | N-(3-{[(furan-2-yl)methyl]amino}-2-hydroxypropyl)-N-(2,4,6-trimethylphenyl)methanesulfonamide; benzoic acid |
| F0808-0453 | 4-tert-butyl-N-(1,3-thiazol-2-yl)benzene-1-sulfonamide |
| F0440-0351 | 2-(2,5-dioxopyrrolidin-1-yl)-N-[4-(4-propoxyphenyl)-1,3-thiazol-2-yl]acetamide |
| F1830-0065 | 2-[3-(2,5-dioxopyrrolidin-1-yl)benzamido]-N-methyl-4H,5H,6H-cyclopenta[b]thiophene-3-carboxamide |
| F2049-0559 | 3-chloro-N-(1-propanoyl-1,2,3,4-tetrahydroquinolin-7-yl)benzamide |
| F2040-0492 | 3-chloro-N-(1-propanoyl-1,2,3,4-tetrahydroquinolin-6-yl)benzamide |
| F2049-0501 | 4-chloro-N-(1-propanoyl-1,2,3,4-tetrahydroquinolin-7-yl)benzamide |
| F1828-0066 | 2-[3-(2,5-dioxopyrrolidin-1-yl)benzamido]-N-methyl-4,5,6,7-tetrahydro-1-benzothiophene-3-carboxamide |
| F6064-1564 | 1-[2-({3-[3-(thiophen-2-yl)-1,2,4-oxadiazol-5-yl]azetidin-1-yl}sulfonyl)ethyl]piperidine-2,6-dione |
| F3284-8534 | (2E)-3-(3,4,5-trimethoxyphenyl)prop-2-enoic acid |
| F5882-4966 | 1-{1-[(2E)-3-(2H-1,3-benzodioxol-5-yl)prop-2-enoyl]piperidin-4-yl}pyrrolidin-2-one |
| F3325-0095 | 2-{[1-(2-methylpropyl)-2,5-dioxopyrrolidin-3-yl]sulfanyl}pyridine-3-carboxylic acid |
| F3023-0293 | 2-[(2,5-dioxo-1-propylpyrrolidin-3-yl)sulfanyl]pyridine-3-carboxylic acid |
| F2764-0087 | 2-chloro-N-({1,4-dioxaspiro[4.4]nonan-2-yl}methyl)-4-nitrobenzamide |
| F3292-0438 | 14-hydroxy-2,14,15-trimethyltetracyclo[8.7.0.0^{2,7}.0^{11,15}]heptadec-6-en-5-one |
| F0020-1803 | 4-(4-carboxyphenoxy)benzoic acid |
| F6782-0558 | (2E)-3-(3-methoxyphenyl)prop-2-enoic acid |
| F5834-2996 | 2-(2,5-dioxopyrrolidin-1-yl)-N-[2-(thiophene-2-carbonyl)-1,2,3,4-tetrahydroisoquinolin-7-yl]acetamide |
| F6660-4137 | 3-phenyl-5-(1H-pyrrol-2-yl)-1,2,4-oxadiazole |
| F1864-0070 | 6-chloro-4-({1,4-dioxa-8-azaspiro[4.5]decan-8-yl}methyl)-7-hydroxy-2H-chromen-2-one |
| F3350-0608 | 4-(2,5-dioxopyrrolidin-1-yl)-N-[(4-methoxyphenyl)methyl]benzene-1-sulfonamide |
| F1831-0048 | 2-[4-(2,5-dioxopyrrolidin-1-yl)benzamido]-N-methyl-4H,5H,6H,7H,8H-cyclohepta[b]thiophene-3-carboxamide |
| F3350-0593 | 4-(2,5-dioxopyrrolidin-1-yl)-N-(3-methoxyphenyl)benzene-1-sulfonamide |
| F3375-0138 | 5-(4-chloro-2-nitrophenyl)-N-(1,1-dioxo-1lambda6-thiolan-3-yl)-N-methylfuran-2-carboxamide |
| F3023-0022 | 2-{[1-(2-methoxyethyl)-2,5-dioxopyrrolidin-3-yl]sulfanyl}pyridine-3-carboxylic acid |
| F0909-0423 | 2-(methylsulfanyl)-1,3-benzothiazole; methoxysulfonic acid |
| F3230-0009 | methyl 4-{[3-(4-chloro-3,5-dimethyl-1H-pyrazol-1-yl)propanoyl]oxy}benzoate |
| F6448-2483 | N-{1-[(2,3-dihydro-1,4-benzodioxin-2-yl)methyl]-1H-pyrazol-4-yl}-2-(2,6-dioxopiperidin-1-yl)ethane-1-sulfonamide |
| F3350-0566 | 4-(2,5-dioxopyrrolidin-1-yl)-N-(2-methoxyphenyl)benzene-1-sulfonamide |
| F0526-0209 | ethyl 2-[3-(2,5-dioxopyrrolidin-1-yl)benzamido]-6-ethyl-4H,5H,6H,7H-thieno[2,3-c]pyridine-3-carboxylate hydrochloride |
| F3244-0178 | 5-(2-chloro-4-nitrophenyl)-2-methylfuran-3-carboxylic acid |
| F0327-0402 | 1-[4-(1,3-benzothiazol-2-yl)phenyl]pyrrolidine-2,5-dione |
| F1345-0315 | 1-(9H-carbazol-9-yl)-3-(morpholin-4-yl)propan-2-ol; benzoic acid |
| F1829-0048 | 2-[4-(2,5-dioxopyrrolidin-1-yl)benzamido]-N,6-dimethyl-4,5,6,7-tetrahydro-1-benzothiophene-3-carboxamide |
| F1831-0066 | 2-[3-(2,5-dioxopyrrolidin-1-yl)benzamido]-N-methyl-4H,5H,6H,7H,8H-cyclohepta[b]thiophene-3-carboxamide |
| F0327-0392 | ethyl 2-[4-(2,5-dioxopyrrolidin-1-yl)benzamido]-4,5,6,7-tetrahydro-1-benzothiophene-3-carboxylate |
| F2019-0733 | N-(6-bromo-1,3-benzothiazol-2-yl)-N-[3-(dimethylamino)propyl]-2-(2,5-dioxopyrrolidin-1-yl)acetamide hydrochloride |
| F2167-9997 | 2-{4-[4-(2-aminoethyl)phenoxy]phenyl}ethan-1-amine dihydrochloride |
| F0327-0320 | 4-(2,5-dioxopyrrolidin-1-yl)-N-(2-methyl-1,3-benzothiazol-5-yl)benzamide |
| F0327-0319 | 4-(2,5-dioxopyrrolidin-1-yl)-N-(2-methyl-1,3-benzothiazol-6-yl)benzamide |
| F3350-0625 | N-(3-acetylphenyl)-4-(2,5-dioxopyrrolidin-1-yl)benzene-1-sulfonamide |
| F1864-0084 | 6-chloro-4-[(2,6-dimethylmorpholin-4-yl)methyl]-7-hydroxy-2H-chromen-2-one |
| F6233-0526 | 1-(3-chlorophenyl)-3-{4-[4-(2-methoxyethyl)-5-oxo-4,5-dihydro-1H-1,2,3,4-tetrazol-1-yl]phenyl}urea |
| F6233-0549 | 1-[(4-chlorophenyl)methyl]-3-{4-[4-(2-methoxyethyl)-5-oxo-4,5-dihydro-1H-1,2,3,4-tetrazol-1-yl]phenyl}urea |
| F0466-0009 | N-(1,3-benzothiazol-2-yl)-3-(2,5-dioxopyrrolidin-1-yl)benzamide |
| F5834-1816 | N-(1-acetyl-2,3-dihydro-1H-indol-6-yl)-2-(2,6-dioxopiperidin-1-yl)ethane-1-sulfonamide |
| F3350-0621 | 4-(2,5-dioxopyrrolidin-1-yl)-N-[2-(thiophen-2-yl)ethyl]benzene-1-sulfonamide |
| F3210-0020 | 5-(4-chloro-2-nitrophenyl)-2-methylfuran-3-carboxylic acid |
| F3350-0564 | N-(4-acetylphenyl)-4-(2,5-dioxopyrrolidin-1-yl)benzene-1-sulfonamide |
| F3350-0696 | N-(3-acetylphenyl)-4-(2,5-dioxopyrrolidin-1-yl)-3-methylbenzene-1-sulfonamide |
| F3350-0586 | 4-(2,5-dioxopyrrolidin-1-yl)-N-(4-ethoxyphenyl)benzene-1-sulfonamide |
| F2416-0239 | 2-(2,4-dichlorophenoxy)-N-(2-propanoyl-1,2,3,4-tetrahydroisoquinolin-7-yl)acetamide |
| F1903-9745 | 6-methyl-2-sulfanylpyridine-3-carboxylic acid |
| F2992-0095 | N-[3-(1,3-benzothiazol-2-yl)thiophen-2-yl]-2-(2,5-dioxopyrrolidin-1-yl)acetamide |
| F0817-0075 | N-(1-benzothiophen-5-yl)-3-(2,5-dioxopyrrolidin-1-yl)benzamide |
| F0817-0057 | N-(1-benzothiophen-5-yl)-4-(2,5-dioxopyrrolidin-1-yl)benzamide |
| F3386-1380 | N-(5-chloro-2-hydroxyphenyl)-2-ethoxy-5-methylbenzene-1-sulfonamide |
| F1864-0146 | 6-chloro-7-hydroxy-4-[(piperidin-1-yl)methyl]-2H-chromen-2-one |
| F9995-0036 | 4-(6-ethoxy-1,3-benzothiazol-2-yl)aniline |
| F2763-0086 | 2-chloro-N-({1,4-dioxaspiro[4.5]decan-2-yl}methyl)-4-nitrobenzamide |
| F0526-0208 | ethyl 2-[4-(2,5-dioxopyrrolidin-1-yl)benzamido]-6-(propan-2-yl)-4H,5H,6H,7H-thieno[2,3-c]pyridine-3-carboxylate hydrochloride |
| F3205-0033 | 2-{[1-(3-methoxypropyl)-2,5-dioxopyrrolidin-3-yl]sulfanyl}pyridine-3-carboxylic acid |
| F1813-1188 | 3-(2,5-dioxopyrrolidin-1-yl)-N-(4-fluoro-1,3-benzothiazol-2-yl)benzamide |
| F9994-5243 | 2-[(6-chloropyridin-3-yl)formamido]acetic acid |
| F1300-0241 | N-(4,5-dimethyl-1,3-benzothiazol-2-yl)-4-(2,5-dioxopyrrolidin-1-yl)benzamide |
| F1300-0252 | N-(4,5-dimethyl-1,3-benzothiazol-2-yl)-3-(2,5-dioxopyrrolidin-1-yl)benzamide |
| F1813-0349 | N-(4,7-dimethyl-1,3-benzothiazol-2-yl)-4-(2,5-dioxopyrrolidin-1-yl)benzamide |
| F1813-0352 | N-(5,7-dimethyl-1,3-benzothiazol-2-yl)-4-(2,5-dioxopyrrolidin-1-yl)benzamide |
| F1015-0025 | ethyl 2-[4-(2,5-dioxopyrrolidin-1-yl)benzamido]-6-methyl-4,5,6,7-tetrahydro-1-benzothiophene-3-carboxylate |
| F1813-1164 | 4-(2,5-dioxopyrrolidin-1-yl)-N-(4-fluoro-1,3-benzothiazol-2-yl)benzamide |
| F3386-1386 | N-(5-chloro-2-hydroxyphenyl)-2,5-dimethoxy-4-methylbenzene-1-sulfonamide |
| F5971-0150 | (2E)-3-(2H-1,3-benzodioxol-5-yl)-N-(2-hydroxy-3-methoxy-2-methylpropyl)prop-2-enamide |
| F3386-1379 | N-(5-chloro-2-hydroxyphenyl)-4-ethoxybenzene-1-sulfonamide |
| F3386-1376 | 5-chloro-N-(5-chloro-2-hydroxyphenyl)-2-methoxybenzene-1-sulfonamide |
| F1864-0335 | 6-chloro-7-hydroxy-4-[(4-methylpiperidin-1-yl)methyl]-2H-chromen-2-one |
| F6244-1336 | (2E)-3-(2H-1,3-benzodioxol-5-yl)-N-[4-(pyrrolidin-1-yl)but-2-yn-1-yl]prop-2-enamide |
| F1813-0481 | N-(4,7-dimethyl-1,3-benzothiazol-2-yl)-3-(2,5-dioxopyrrolidin-1-yl)benzamide |
| F1813-0484 | N-(5,7-dimethyl-1,3-benzothiazol-2-yl)-3-(2,5-dioxopyrrolidin-1-yl)benzamide |
| F0327-0303 | N-(4,6-dimethyl-1,3-benzothiazol-2-yl)-4-(2,5-dioxopyrrolidin-1-yl)benzamide |
| F1813-1187 | N-(4,6-difluoro-1,3-benzothiazol-2-yl)-3-(2,5-dioxopyrrolidin-1-yl)benzamide |
| F0009-0381 | 2-methyl-N-{4-[(1,3-thiazol-2-yl)sulfamoyl]phenyl}propanamide |
| F0440-0388 | 4-(2,5-dioxopyrrolidin-1-yl)-2-[(2E)-1-methyl-2,3-dihydro-1H-1,3-benzodiazol-2-ylidene]-3-oxobutanenitrile |
| F6617-4185 | 3-[3-(thiophen-2-yl)-1,2,4-oxadiazol-5-yl]propanehydrazide |
| F6237-0243 | N-(5-chloro-2-hydroxyphenyl)-2-phenyl-2H-1,2,3,4-tetrazole-5-carboxamide |
| F1199-0067 | 2-{[2,5-dioxo-1-(propan-2-yl)pyrrolidin-3-yl]sulfanyl}benzoic acid |
| F0326-0268 | 4-(2,5-dioxopyrrolidin-1-yl)-N-[5-(3-methylphenyl)-1,3,4-thiadiazol-2-yl]benzamide |
| F3269-0037 | 3-[2-(2,5-dioxopyrrolidin-1-yl)ethyl]-5-[(4-methylphenyl)amino]-1,3-thiazolidine-2,4-dione |
| F0327-0299 | N-(4-chloro-1,3-benzothiazol-2-yl)-4-(2,5-dioxopyrrolidin-1-yl)benzamide |
| F0327-0293 | N-(6-chloro-1,3-benzothiazol-2-yl)-4-(2,5-dioxopyrrolidin-1-yl)benzamide |
| F1822-0283 | 4-(2,5-dioxopyrrolidin-1-yl)-N-(4-ethyl-1,3-benzothiazol-2-yl)benzamide |
| F6250-0278 | 3-[2-(2,6-dioxopiperidin-1-yl)ethanesulfonamido]cyclohexyl N-phenylcarbamate |
| F3350-0579 | N-(3,4-dimethoxyphenyl)-4-(2,5-dioxopyrrolidin-1-yl)benzene-1-sulfonamide |
| F0920-5439 | 6-methyl 3-propan-2-yl 4-(3-chloro-5-ethoxy-4-hydroxyphenyl)-2,7-dimethyl-5-oxo-1,4,5,6,7,8-hexahydroquinoline-3,6-dicarboxylate |
| F1199-0041 | 2-[(1-ethyl-2,5-dioxopyrrolidin-3-yl)sulfanyl]benzoic acid |
| F2708-0061 | N-(7-chloro-4-methoxy-1,3-benzothiazol-2-yl)-3-(2,5-dioxopyrrolidin-1-yl)benzamide |
| F0327-0291 | 4-(2,5-dioxopyrrolidin-1-yl)-N-(4-methoxy-1,3-benzothiazol-2-yl)benzamide |
| F0466-0001 | 3-(2,5-dioxopyrrolidin-1-yl)-N-(6-methoxy-1,3-benzothiazol-2-yl)benzamide |
| F3269-0033 | 3-[2-(2,5-dioxopyrrolidin-1-yl)ethyl]-5-(phenylamino)-1,3-thiazolidine-2,4-dione |
| F1757-0285 | 1-(4-chloro-3,5-dimethyl-1H-pyrazol-1-yl)-3-(naphthalen-2-yloxy)propan-2-ol |
| F3023-0448 | 2-[(2,5-dioxo-1-propylpyrrolidin-3-yl)sulfanyl]benzoic acid |
| F3350-0559 | methyl 4-[4-(2,5-dioxopyrrolidin-1-yl)benzenesulfonamido]benzoate |
| F3350-0575 | methyl 2-[4-(2,5-dioxopyrrolidin-1-yl)benzenesulfonamido]benzoate |
| F1902-0079 | 3-(4-aminophenyl)-6,7-dimethyl-4H-chromen-4-one |
| F3374-0425 | N-(6-chloroquinolin-4-yl)-4-(2,5-dioxopyrrolidin-1-yl)benzamide |
| F1822-0391 | 3-(2,5-dioxopyrrolidin-1-yl)-N-(4-ethyl-1,3-benzothiazol-2-yl)benzamide |
| F0806-0249 | 3-(2,5-dioxopyrrolidin-1-yl)-N-(5-phenyl-1,3-thiazol-2-yl)benzamide |
| F1721-0034 | 2-[(4-methylphenoxy)methyl]-5-phenyl-1,3,4-oxadiazole |
| F2738-1944 | N-(4,6-dimethyl-1,3-benzothiazol-2-yl)-2-(2,5-dioxopyrrolidin-1-yl)-N-[(pyridin-2-yl)methyl]acetamide |
| F1822-0288 | 4-(2,5-dioxopyrrolidin-1-yl)-N-[6-(propan-2-yl)-1,3-benzothiazol-2-yl]benzamide |
| F1813-0344 | N-(5-chloro-4-methyl-1,3-benzothiazol-2-yl)-4-(2,5-dioxopyrrolidin-1-yl)benzamide |
| F1813-0345 | N-(6-chloro-4-methyl-1,3-benzothiazol-2-yl)-4-(2,5-dioxopyrrolidin-1-yl)benzamide |
| F1813-0348 | N-(7-chloro-4-methyl-1,3-benzothiazol-2-yl)-4-(2,5-dioxopyrrolidin-1-yl)benzamide |
| F1813-0480 | N-(7-chloro-4-methyl-1,3-benzothiazol-2-yl)-3-(2,5-dioxopyrrolidin-1-yl)benzamide |
| F0327-0290 | 4-(2,5-dioxopyrrolidin-1-yl)-N-(6-methoxy-1,3-benzothiazol-2-yl)benzamide |
| F1382-0013 | 3-(2,5-dioxopyrrolidin-1-yl)-N-[4-(piperidine-1-sulfonyl)phenyl]benzamide |
| F0327-0322 | 4-(2,5-dioxopyrrolidin-1-yl)-N-(4-phenyl-1,3-thiazol-2-yl)benzamide |
| F0012-0791 | N-{4-[(1,3-thiazol-2-yl)sulfamoyl]phenyl}acetamide |
| F1374-1927 | N-[5-(2,4-difluorophenyl)-1,3,4-oxadiazol-2-yl]-4-(2,5-dioxopyrrolidin-1-yl)benzamide |
| F5497-0110 | 1-[2-({4-[5-(furan-2-yl)-1,3,4-thiadiazol-2-yl]piperidin-1-yl}sulfonyl)ethyl]piperidine-2,6-dione |
| F6237-2966 | N-(5-chloro-2-hydroxyphenyl)-2-[4-(trifluoromethyl)phenyl]-2H-1,2,3,4-tetrazole-5-carboxamide |
| F2895-0419 | N-(6-chloro-4-methyl-1,3-benzothiazol-2-yl)-2-(2,5-dioxopyrrolidin-1-yl)-N-[(pyridin-3-yl)methyl]acetamide |
| F2895-0253 | N-(6-chloro-4-methyl-1,3-benzothiazol-2-yl)-2-(2,5-dioxopyrrolidin-1-yl)-N-[(pyridin-2-yl)methyl]acetamide |
| F1757-0030 | 1-(4-bromo-3,5-dimethyl-1H-pyrazol-1-yl)-3-(naphthalen-2-yloxy)propan-2-ol |
| F1813-0350 | 4-(2,5-dioxopyrrolidin-1-yl)-N-(4-methoxy-7-methyl-1,3-benzothiazol-2-yl)benzamide |
| F0777-2101 | 2-(phenoxymethyl)-5-phenyl-1,3,4-oxadiazole |
| F0852-0174 | 2-(4-methoxyphenyl)-1H-1,3-benzodiazole |
| F2590-0796 | ethyl 4-cyano-5-[2-(2,5-dioxopyrrolidin-1-yl)acetamido]-3-methylthiophene-2-carboxylate |
| F1822-0396 | 3-(2,5-dioxopyrrolidin-1-yl)-N-[6-(propan-2-yl)-1,3-benzothiazol-2-yl]benzamide |
| F2368-0950 | 3-(2,5-dioxopyrrolidin-1-yl)-N-{5-[(4-methylphenyl)methyl]-1,3,4-oxadiazol-2-yl}benzamide |
| F1374-0894 | 3-(2,5-dioxopyrrolidin-1-yl)-N-(5-phenyl-1,3,4-oxadiazol-2-yl)benzamide |
| F1813-0476 | N-(5-chloro-4-methyl-1,3-benzothiazol-2-yl)-3-(2,5-dioxopyrrolidin-1-yl)benzamide |
| F1786-0014 | ethyl 4-(3,5-dichloro-2-hydroxybenzenesulfonyl)piperazine-1-carboxylate |
| F0739-0014 | 2-({5,6-dimethyl-4-oxo-3-phenyl-3H,4H-thieno[2,3-d]pyrimidin-2-yl}sulfanyl)-N-[3-(morpholin-4-yl)propyl]acetamide |
| F0703-0001 | N-(2,3,5-trichloro-4-hydroxyphenyl)acetamide |
| F0778-0280 | N-[2-(2,4-dimethylphenyl)-2H,4H,6H-thieno[3,4-c]pyrazol-3-yl]-4-(2,5-dioxopyrrolidin-1-yl)benzamide |
| F1620-0030 | 3-[4-(2,5-dioxopyrrolidin-1-yl)benzamido]-1-benzofuran-2-carboxamide |
| F3350-0558 | ethyl 4-[4-(2,5-dioxopyrrolidin-1-yl)benzenesulfonamido]benzoate |
| F1813-0477 | N-(6-chloro-4-methyl-1,3-benzothiazol-2-yl)-3-(2,5-dioxopyrrolidin-1-yl)benzamide |
| F1108-0022 | N-benzyl-2-(1H-indol-3-yl)acetamide |
| F0326-0686 | 4-(2,5-dioxopyrrolidin-1-yl)-N-[5-(2-methylphenyl)-1,3,4-thiadiazol-2-yl]benzamide |
| F0326-0655 | 4-(2,5-dioxopyrrolidin-1-yl)-N-(5-phenyl-1,3,4-thiadiazol-2-yl)benzamide |
| F2738-2549 | N-(4,5-dimethyl-1,3-benzothiazol-2-yl)-2-(2,5-dioxopyrrolidin-1-yl)-N-[(pyridin-2-yl)methyl]acetamide |
| F2738-0218 | 2-(2,5-dioxopyrrolidin-1-yl)-N-(4-ethyl-1,3-benzothiazol-2-yl)-N-[(pyridin-2-yl)methyl]acetamide |
| F0834-1072 | 3-[3-(2,5-dioxopyrrolidin-1-yl)benzamido]-1-benzofuran-2-carboxamide |
| F1174-3261 | 3-phenylquinoline-2,4-dicarboxylic acid |
| F1813-0482 | 3-(2,5-dioxopyrrolidin-1-yl)-N-(4-methoxy-7-methyl-1,3-benzothiazol-2-yl)benzamide |
| F3019-0042 | 2-{[1-(2-methylpropyl)-2,5-dioxopyrrolidin-3-yl]sulfanyl}benzoic acid |
| F5460-0730 | 2-(2,5-dioxopyrrolidin-1-yl)-N-[1-(4-fluoro-1,3-benzothiazol-2-yl)-3-methyl-1H-pyrazol-5-yl]acetamide |
| F6213-0291 | N-(3,4-dichlorophenyl)-7,7-dimethyl-6,8-dioxa-2-azaspiro[3.5]nonane-2-carboxamide |
| F2756-0037 | N-(1,3-benzothiazol-2-yl)-4-(2,5-dioxopyrrolidin-1-yl)-N-[2-(morpholin-4-yl)ethyl]benzamide hydrochloride |
| F1328-0052 | 1-acetyl-3-(2,6-dimethyl-4H-pyran-4-ylidene)-2,3-dihydro-1H-indol-2-one |
| F0327-0327 | 4-(2,5-dioxopyrrolidin-1-yl)-N-[4-(4-methylphenyl)-1,3-thiazol-2-yl]benzamide |
| F5860-4860 | 2-(2,6-dioxopiperidin-1-yl)-N-{[3-(4-fluorophenoxy)phenyl]methyl}ethane-1-sulfonamide |
| F0327-0339 | 4-(2,5-dioxopyrrolidin-1-yl)-N-[4-(4-fluorophenyl)-1,3-thiazol-2-yl]benzamide |
| F0526-2106 | methyl 5-[(2H-1,3-benzodioxol-5-yl)methyl]-2-[2-(2,5-dioxopyrrolidin-1-yl)acetamido]-4-methylthiophene-3-carboxylate |
| F2635-0575 | N-(1,3-benzothiazol-2-yl)-2-(2,5-dioxopyrrolidin-1-yl)-N-[(pyridin-2-yl)methyl]acetamide |
| F2738-1339 | N-(6-chloro-1,3-benzothiazol-2-yl)-2-(2,5-dioxopyrrolidin-1-yl)-N-[(pyridin-2-yl)methyl]acetamide |
| F0466-0002 | 3-(2,5-dioxopyrrolidin-1-yl)-N-(6-ethoxy-1,3-benzothiazol-2-yl)benzamide |
| F3095-3044 | (2E)-3-(4-ethoxy-3-methoxyphenyl)-1-(4-methylpiperidin-1-yl)prop-2-en-1-one |
| F1822-0285 | N-(4-bromo-1,3-benzothiazol-2-yl)-4-(2,5-dioxopyrrolidin-1-yl)benzamide |
| F1799-0246 | 1-(4-bromophenyl)-3-(2-methyl-1H-1,3-benzodiazol-1-yl)pyrrolidine-2,5-dione |
| F0918-6267 | 9,13-dimethyl-8-oxa-10,12-diazatricyclo[7.3.1.0^{2,7}]trideca-2,4,6-triene-11-thione |
| F2510-0331 | 1-methyl 2-{4-oxo-6-[(pyrimidin-2-ylsulfanyl)methyl]-4H-pyran-3-yl} benzene-1,2-dicarboxylate |
| F1721-0084 | 2-(2-chlorophenyl)-5-[(2-methoxyphenoxy)methyl]-1,3,4-oxadiazole |
| F2738-2307 | 2-(2,5-dioxopyrrolidin-1-yl)-N-(4-methoxy-7-methyl-1,3-benzothiazol-2-yl)-N-[(pyridin-2-yl)methyl]acetamide |
| F2510-0332 | 1-methyl 2-(6-{[(4-methylpyrimidin-2-yl)sulfanyl]methyl}-4-oxo-4H-pyran-3-yl) benzene-1,2-dicarboxylate |
| F0853-0315 | 2-phenyl-1H-1,3-benzodiazole |
| F2645-0211 | 3-(2,5-dioxopyrrolidin-1-yl)-N-{5-[3-(methylsulfanyl)phenyl]-1,3,4-oxadiazol-2-yl}benzamide |
| F2738-0976 | 2-(2,5-dioxopyrrolidin-1-yl)-N-(6-ethyl-1,3-benzothiazol-2-yl)-N-[(pyridin-2-yl)methyl]acetamide |
| F2738-2670 | N-(5-chloro-4-methyl-1,3-benzothiazol-2-yl)-2-(2,5-dioxopyrrolidin-1-yl)-N-[(pyridin-2-yl)methyl]acetamide |
| F3019-0047 | 2-{[1-(2-methoxyethyl)-2,5-dioxopyrrolidin-3-yl]sulfanyl}benzoic acid |
| F1822-0393 | N-(4-bromo-1,3-benzothiazol-2-yl)-3-(2,5-dioxopyrrolidin-1-yl)benzamide |
| F0326-0401 | N-[5-(4-chlorophenyl)-1,3,4-thiadiazol-2-yl]-4-(2,5-dioxopyrrolidin-1-yl)benzamide |
| F0327-0294 | N-(6-bromo-1,3-benzothiazol-2-yl)-4-(2,5-dioxopyrrolidin-1-yl)benzamide |
| F1813-0343 | N-(4,5-dichloro-1,3-benzothiazol-2-yl)-4-(2,5-dioxopyrrolidin-1-yl)benzamide |
| F3260-0607 | 8-bromo-1,7-diethyl-3-methyl-2,3,6,7-tetrahydro-1H-purine-2,6-dione |
| F3374-0059 | methyl 4-[4-(2,5-dioxopyrrolidin-1-yl)benzamido]-2-methylquinoline-6-carboxylate |
| F9995-2667 | 6-benzoyl-2,3-dihydro-1H-indol-2-one |
| F1374-0789 | N-[5-(4-chlorophenyl)-1,3,4-oxadiazol-2-yl]-3-(2,5-dioxopyrrolidin-1-yl)benzamide |
| F1374-0774 | N-[5-(4-chlorophenyl)-1,3,4-oxadiazol-2-yl]-4-(2,5-dioxopyrrolidin-1-yl)benzamide |
| F1591-0629 | N-[4-(diethylsulfamoyl)phenyl]-4-(2,5-dioxopyrrolidin-1-yl)benzamide |
| F1601-0132 | 4-(4-chlorophenyl)-1-phenyl-1H-1,2,3-triazol-5-amine |
| F0327-0335 | 4-(2,5-dioxopyrrolidin-1-yl)-N-[4-(2,4,6-trimethylphenyl)-1,3-thiazol-2-yl]benzamide |
| F0327-0340 | N-[4-(4-cyanophenyl)-1,3-thiazol-2-yl]-4-(2,5-dioxopyrrolidin-1-yl)benzamide |
| F0327-0329 | N-[4-(3,4-dimethylphenyl)-1,3-thiazol-2-yl]-4-(2,5-dioxopyrrolidin-1-yl)benzamide |
| F1813-0346 | N-(4,6-dichloro-1,3-benzothiazol-2-yl)-4-(2,5-dioxopyrrolidin-1-yl)benzamide |
| F1813-0347 | N-(4,7-dichloro-1,3-benzothiazol-2-yl)-4-(2,5-dioxopyrrolidin-1-yl)benzamide |
| F1199-0046 | 2-[(1-butyl-2,5-dioxopyrrolidin-3-yl)sulfanyl]benzoic acid |
| F2738-1702 | N-(4,6-difluoro-1,3-benzothiazol-2-yl)-2-(2,5-dioxopyrrolidin-1-yl)-N-[(pyridin-2-yl)methyl]acetamide |
| F3269-0032 | 5-[(4-bromophenyl)amino]-3-[2-(2,5-dioxopyrrolidin-1-yl)ethyl]-1,3-thiazolidine-2,4-dione |
| F3260-0610 | 8-bromo-1-ethyl-3-methyl-7-(propan-2-yl)-2,3,6,7-tetrahydro-1H-purine-2,6-dione |
| F3205-0048 | 2-{[1-(3-methoxypropyl)-2,5-dioxopyrrolidin-3-yl]sulfanyl}benzoic acid |
| F3205-0041 | 3-(4-methylpiperazin-1-yl)-1-(naphthalen-1-yl)pyrrolidine-2,5-dione |
| F0327-0338 | 4-(2,5-dioxopyrrolidin-1-yl)-N-[4-(2-methoxyphenyl)-1,3-thiazol-2-yl]benzamide |
| F1813-0475 | N-(4,5-dichloro-1,3-benzothiazol-2-yl)-3-(2,5-dioxopyrrolidin-1-yl)benzamide |
| F0327-0353 | 4-(2,5-dioxopyrrolidin-1-yl)-N-[4-(thiophen-2-yl)-1,3-thiazol-2-yl]benzamide |
| F0327-0317 | 4-(2,5-dioxopyrrolidin-1-yl)-N-{4H,5H-naphtho[1,2-d][1,3]thiazol-2-yl}benzamide |
| F1813-0478 | N-(4,6-dichloro-1,3-benzothiazol-2-yl)-3-(2,5-dioxopyrrolidin-1-yl)benzamide |
| F2173-1249 | methyl 1-hydroxynaphthalene-2-carboxylate |
| F6276-0577 | 1-{3-[3-(1,3-benzothiazol-2-yloxy)azetidine-1-carbonyl]phenyl}pyrrolidine-2,5-dione |
| F2708-0385 | 1-{3-[4-(7-chloro-4-methoxy-1,3-benzothiazol-2-yl)piperazine-1-carbonyl]phenyl}pyrrolidine-2,5-dione |
| F2738-2428 | N-(7-chloro-4-methyl-1,3-benzothiazol-2-yl)-2-(2,5-dioxopyrrolidin-1-yl)-N-[(pyridin-2-yl)methyl]acetamide |
| F3260-0609 | 8-bromo-1-ethyl-3-methyl-7-propyl-2,3,6,7-tetrahydro-1H-purine-2,6-dione |
| F6159-0248 | 1-(3-{4-[(1,3-benzothiazol-2-yloxy)methyl]piperidine-1-carbonyl}phenyl)pyrrolidine-2,5-dione |
| F0466-0018 | N-[4-(4-chlorophenyl)-1,3-thiazol-2-yl]-3-(2,5-dioxopyrrolidin-1-yl)benzamide |
| F1813-0479 | N-(4,7-dichloro-1,3-benzothiazol-2-yl)-3-(2,5-dioxopyrrolidin-1-yl)benzamide |
| F3367-0007 | N-(6-bromo-2-methylquinolin-4-yl)-4-(2,5-dioxopyrrolidin-1-yl)benzamide |
| F0396-0402 | 4-(2,5-dioxopyrrolidin-1-yl)-N-[2-(3-methylphenyl)-2H,4H,6H-thieno[3,4-c]pyrazol-3-yl]benzamide |
| F0526-2010 | ethyl 5-[(2H-1,3-benzodioxol-5-yl)methyl]-2-[2-(2,5-dioxopyrrolidin-1-yl)acetamido]-4-methylthiophene-3-carboxylate |
| F2898-4411 | N-(6-chloro-4-methyl-1,3-benzothiazol-2-yl)-4-(2,5-dioxopyrrolidin-1-yl)-N-[(oxolan-2-yl)methyl]benzamide |
| F2515-0860 | 2-(2,5-dioxopyrrolidin-1-yl)-N-(6-ethyl-1,3-benzothiazol-2-yl)-N-[(pyridin-3-yl)methyl]acetamide |
| F0536-0555 | N-{4H-chromeno[4,3-d][1,3]thiazol-2-yl}-4-(2,5-dioxopyrrolidin-1-yl)benzamide |
| F0327-0323 | N-[4-(4-chlorophenyl)-1,3-thiazol-2-yl]-4-(2,5-dioxopyrrolidin-1-yl)benzamide |
| F3385-2580 | ethyl 4-[(6-chloro-7-hydroxy-2-oxo-2H-chromen-4-yl)methyl]piperazine-1-carboxylate |
| F1374-0999 | 3-(2,5-dioxopyrrolidin-1-yl)-N-[5-(thiophen-2-yl)-1,3,4-oxadiazol-2-yl]benzamide |
| F5450-0502 | 4-(2,5-dioxopyrrolidin-1-yl)-N-{4-[(1,2,3,4-tetrahydroisoquinolin-2-yl)methyl]-1,3-thiazol-2-yl}benzamide hydrochloride |
| F0466-0029 | 3-(2,5-dioxopyrrolidin-1-yl)-N-[4-(4-methoxy-3-methylphenyl)-1,3-thiazol-2-yl]benzamide |
| F1813-0483 | N-(4,7-dimethoxy-1,3-benzothiazol-2-yl)-3-(2,5-dioxopyrrolidin-1-yl)benzamide |
| F0608-0592 | 3-(2,5-dioxopyrrolidin-1-yl)-N-[5-(5,6,7,8-tetrahydronaphthalen-2-yl)-1,3,4-oxadiazol-2-yl]benzamide |
| F6274-0182 | 1-(4-methoxy-1,3-benzothiazol-2-yl)azetidin-3-yl 3-(2,5-dioxopyrrolidin-1-yl)benzoate |
| F0778-0277 | 4-(2,5-dioxopyrrolidin-1-yl)-N-[2-(2-methylphenyl)-2H,4H,6H-thieno[3,4-c]pyrazol-3-yl]benzamide |
| F3023-0295 | 2-{[1-(2-methylphenyl)-2,5-dioxopyrrolidin-3-yl]sulfanyl}pyridine-3-carboxylic acid |
| F6237-3744 | N-(5-chloro-2-hydroxyphenyl)-2-[4-(trifluoromethoxy)phenyl]-2H-1,2,3,4-tetrazole-5-carboxamide |
| F2738-0702 | 2-(2,5-dioxopyrrolidin-1-yl)-N-(5-methoxy-1,3-benzothiazol-2-yl)-N-[(pyridin-2-yl)methyl]acetamide |
| F0536-0731 | N-{4H-chromeno[4,3-d][1,3]thiazol-2-yl}-3-(2,5-dioxopyrrolidin-1-yl)benzamide |
| F3023-0024 | 2-{[1-(2,3-dimethylphenyl)-2,5-dioxopyrrolidin-3-yl]sulfanyl}pyridine-3-carboxylic acid |
| F0396-0211 | 4-(2,5-dioxopyrrolidin-1-yl)-N-{2-phenyl-2H,4H,6H-thieno[3,4-c]pyrazol-3-yl}benzamide |
| F0778-0278 | 4-(2,5-dioxopyrrolidin-1-yl)-N-[2-(4-methylphenyl)-2H,4H,6H-thieno[3,4-c]pyrazol-3-yl]benzamide |
| F1957-0113 | 4-bromo-5,6,7,8-tetrahydro-1,5-naphthyridine-2-carboxylic acid |
| F3275-0015 | (2E)-3-(4-propoxyphenyl)prop-2-enoic acid |
| F3023-0025 | 2-{[1-(2,4-dimethylphenyl)-2,5-dioxopyrrolidin-3-yl]sulfanyl}pyridine-3-carboxylic acid |
| F5323-0610 | N-[2-(1,3-benzothiazol-2-yl)-2H,4H,5H,6H-cyclopenta[c]pyrazol-3-yl]-2-(2,5-dioxopyrrolidin-1-yl)acetamide |
| F2018-0572 | N-(6-chloro-1,3-benzothiazol-2-yl)-N-[2-(dimethylamino)ethyl]-3-(2,5-dioxopyrrolidin-1-yl)benzamide hydrochloride |
| F2019-0936 | N-[3-(dimethylamino)propyl]-4-(2,5-dioxopyrrolidin-1-yl)-N-(4-methyl-1,3-benzothiazol-2-yl)benzamide hydrochloride |
| F2019-0953 | N-[3-(dimethylamino)propyl]-3-(2,5-dioxopyrrolidin-1-yl)-N-(4-methyl-1,3-benzothiazol-2-yl)benzamide hydrochloride |
| F3023-0278 | 2-({2,5-dioxo-1-[3-(propan-2-yloxy)propyl]pyrrolidin-3-yl}sulfanyl)benzoic acid |
| F1822-0286 | N-{10,13-dioxa-4-thia-6-azatricyclo[7.4.0.0^{3,7}]trideca-1,3(7),5,8-tetraen-5-yl}-4-(2,5-dioxopyrrolidin-1-yl)benzamide |
| F2189-0261 | 1-benzoyl-1,2,3,4-tetrahydroquinolin-6-amine |
| F3023-0617 | 2-{[1-(4-methylphenyl)-2,5-dioxopyrrolidin-3-yl]sulfanyl}pyridine-3-carboxylic acid |
| F2518-0480 | N-[5-(4-bromophenyl)-1,3,4-oxadiazol-2-yl]-4-(2,5-dioxopyrrolidin-1-yl)benzamide |
| F2518-0498 | N-[5-(4-bromophenyl)-1,3,4-oxadiazol-2-yl]-3-(2,5-dioxopyrrolidin-1-yl)benzamide |
| F2748-0183 | N-(5-chloro-2-hydroxyphenyl)-2-(1,1,3-trioxo-2,3-dihydro-1lambda6,2-benzothiazol-2-yl)acetamide |
| F2257-0298 | 2-(naphthalen-1-yl)-N-(2-oxo-2,3-dihydro-1H-indol-5-yl)acetamide |
| F1918-0038 | 7-hydroxy-4-methyl-2H-chromen-2-one |
| F5460-0270 | 2-(2,5-dioxopyrrolidin-1-yl)-N-[1-(6-methoxy-1,3-benzothiazol-2-yl)-3-methyl-1H-pyrazol-5-yl]acetamide |
| F2019-0428 | N-[3-(dimethylamino)propyl]-4-(2,5-dioxopyrrolidin-1-yl)-N-(6-fluoro-1,3-benzothiazol-2-yl)benzamide hydrochloride |
| F2019-1317 | N-[3-(dimethylamino)propyl]-4-(2,5-dioxopyrrolidin-1-yl)-N-(4-fluoro-1,3-benzothiazol-2-yl)benzamide hydrochloride |
| F1822-0394 | N-{10,13-dioxa-4-thia-6-azatricyclo[7.4.0.0^{3,7}]trideca-1,3(7),5,8-tetraen-5-yl}-3-(2,5-dioxopyrrolidin-1-yl)benzamide |
| F2515-0606 | N-(6-chloro-1,3-benzothiazol-2-yl)-2-(2,5-dioxopyrrolidin-1-yl)-N-[(pyridin-3-yl)methyl]acetamide |
| F2189-0257 | 1-benzoyl-1,2,3,4-tetrahydroquinolin-7-amine |
| F0778-0281 | N-[2-(3,5-dimethylphenyl)-2H,4H,6H-thieno[3,4-c]pyrazol-3-yl]-4-(2,5-dioxopyrrolidin-1-yl)benzamide |
| F0373-0285 | 8-bromo-1,3-dimethyl-7-propyl-2,3,6,7-tetrahydro-1H-purine-2,6-dione |
| F0373-0540 | 8-bromo-1,3-dimethyl-7-(propan-2-yl)-2,3,6,7-tetrahydro-1H-purine-2,6-dione |
| F2756-2235 | N-(5,6-dimethyl-1,3-benzothiazol-2-yl)-4-(2,5-dioxopyrrolidin-1-yl)-N-[2-(morpholin-4-yl)ethyl]benzamide hydrochloride |
| F2211-0008 | 6-amino-2-(2-methoxyphenyl)-4H-chromen-4-one |
| F2515-1114 | 2-(2,5-dioxopyrrolidin-1-yl)-N-(4-methoxy-1,3-benzothiazol-2-yl)-N-[(pyridin-3-yl)methyl]acetamide |
| F2738-0339 | 2-(2,5-dioxopyrrolidin-1-yl)-N-(4-ethoxy-1,3-benzothiazol-2-yl)-N-[(pyridin-2-yl)methyl]acetamide |
| F2756-1735 | N-(4,7-dimethyl-1,3-benzothiazol-2-yl)-3-(2,5-dioxopyrrolidin-1-yl)-N-[2-(morpholin-4-yl)ethyl]benzamide hydrochloride |
| F5857-2692 | N-(2-{[1,1'-biphenyl]-4-yl}-2-hydroxypropyl)-2-(2,5-dioxopyrrolidin-1-yl)acetamide |
| F0799-0087 | 8-bromo-1,3-dimethyl-7-pentyl-2,3,6,7-tetrahydro-1H-purine-2,6-dione |
| F1211-0295 | 4-(2,5-dioxopyrrolidin-1-yl)-N-[4-(4-ethoxyphenyl)-5-methyl-1,3-thiazol-2-yl]benzamide |
| F1065-0545 | 2-{[1-(2-chlorophenyl)-2,5-dioxopyrrolidin-3-yl]sulfanyl}pyridine-3-carboxylic acid |
| F0373-0284 | 8-bromo-7-ethyl-1,3-dimethyl-2,3,6,7-tetrahydro-1H-purine-2,6-dione |
| F2783-0556 | 2-chloro-N-[4-methyl-3-(2-oxopyrrolidin-1-yl)phenyl]-4-nitrobenzamide |
| F2784-0437 | 2-chloro-4-nitro-N-[3-(2-oxopiperidin-1-yl)phenyl]benzamide |
| F0021-0037 | 3-{[4-(morpholin-4-yl)phenyl]amino}-1-phenylpyrrolidine-2,5-dione |
| F3023-0624 | 2-{[1-(4-ethylphenyl)-2,5-dioxopyrrolidin-3-yl]sulfanyl}pyridine-3-carboxylic acid |
| F0001-0277 | (2E)-3-(2,5-difluorophenyl)prop-2-enoic acid |
| F2093-0698 | N-{5-benzyl-4H,5H,6H,7H-[1,3]thiazolo[5,4-c]pyridin-2-yl}-3-(2,5-dioxopyrrolidin-1-yl)benzamide hydrochloride |
| F0496-0188 | 3-(2,5-dioxopyrrolidin-1-yl)-N-[4-(4-ethoxyphenyl)-1,3-thiazol-2-yl]benzamide |
| F1065-0447 | 2-[(1-benzyl-2,5-dioxopyrrolidin-3-yl)sulfanyl]pyridine-3-carboxylic acid |
| F0496-0161 | 4-(2,5-dioxopyrrolidin-1-yl)-N-[4-(4-ethoxyphenyl)-1,3-thiazol-2-yl]benzamide |
| F0326-0135 | N-[5-(adamantan-1-yl)-1,3,4-thiadiazol-2-yl]-4-(2,5-dioxopyrrolidin-1-yl)benzamide |
| F1905-6545 | triethyl[2-(3,4,5-trimethoxybenzoyloxy)ethyl]azanium 4-methylbenzene-1-sulfonate |
| F0675-0061 | 2-(1H-indol-3-yl)-N-(3-methylbutyl)-2-oxoacetamide |
| F6414-3147 | N-[2-(3,4-dimethoxyphenyl)-2-hydroxyethyl]-2-(4-ethoxyphenyl)acetamide |
| F2666-0051 | 2-chloro-4-nitro-N-{2-oxo-1-azatricyclo[6.3.1.0^{4,12}]dodeca-4,6,8(12)-trien-6-yl}benzamide |
| F3098-4784 | (2E)-3-(3,4-diethoxyphenyl)-N-(2-methylpropyl)prop-2-enamide |
| F2756-0460 | N-(4-chloro-1,3-benzothiazol-2-yl)-3-(2,5-dioxopyrrolidin-1-yl)-N-[2-(morpholin-4-yl)ethyl]benzamide hydrochloride |
| F3230-0044 | 7-[3-(4-chloro-3,5-dimethyl-1H-pyrazol-1-yl)-2-hydroxypropoxy]-4-methyl-2H-chromen-2-one |
| F0373-0004 | 8-bromo-3-methyl-7-(propan-2-yl)-2,3,6,7-tetrahydro-1H-purine-2,6-dione |
| F0396-0212 | N-[2-(4-chlorophenyl)-2H,4H,6H-thieno[3,4-c]pyrazol-3-yl]-4-(2,5-dioxopyrrolidin-1-yl)benzamide |
| F3023-0833 | 1-cyclohexyl-3-[(4-ethoxyphenyl)amino]pyrrolidine-2,5-dione |
| F0327-0346 | N-[4-(3,4-dimethoxyphenyl)-1,3-thiazol-2-yl]-4-(2,5-dioxopyrrolidin-1-yl)benzamide |
| F2664-0051 | 2-chloro-4-nitro-N-{11-oxo-1-azatricyclo[6.3.1.0^{4,12}]dodeca-4(12),5,7-trien-6-yl}benzamide |
| F2736-1116 | 4-(2,5-dioxopyrrolidin-1-yl)-N-(6-methyl-1,3-benzothiazol-2-yl)-N-[3-(morpholin-4-yl)propyl]benzamide hydrochloride |
| F0850-6838 | (2E)-3-(4-bromophenyl)prop-2-enoic acid |
| F9994-5179 | 2-methoxy-3-(methoxymethoxy)benzaldehyde |
| F3023-0379 | 3-[(4-ethylphenyl)amino]-1-phenylpyrrolidine-2,5-dione |
| F3064-0111 | 8-bromo-1,3-dimethyl-7-(2-methylpropyl)-2,3,6,7-tetrahydro-1H-purine-2,6-dione |
| F2018-2477 | N-[2-(dimethylamino)ethyl]-3-(2,5-dioxopyrrolidin-1-yl)-N-(4-methoxy-7-methyl-1,3-benzothiazol-2-yl)benzamide hydrochloride |
| F0327-0324 | N-[4-(4-bromophenyl)-1,3-thiazol-2-yl]-4-(2,5-dioxopyrrolidin-1-yl)benzamide |
| F2750-0071 | N-(5-chloro-2-hydroxyphenyl)-2-(1,1,3-trioxo-2,3-dihydro-1lambda6,2-benzothiazol-2-yl)propanamide |
| F3023-0302 | 2-{[1-(4-hydroxyphenyl)-2,5-dioxopyrrolidin-3-yl]sulfanyl}pyridine-3-carboxylic acid |
| F5460-1190 | 2-(2,5-dioxopyrrolidin-1-yl)-N-[1-(4-ethoxy-1,3-benzothiazol-2-yl)-3-methyl-1H-pyrazol-5-yl]acetamide |
| F3023-0613 | 2-{[1-(4-chlorophenyl)-2,5-dioxopyrrolidin-3-yl]sulfanyl}pyridine-3-carboxylic acid |
| F2710-0582 | N-(7-chloro-4-methoxy-1,3-benzothiazol-2-yl)-2-(2,5-dioxopyrrolidin-1-yl)-N-[(pyridin-3-yl)methyl]acetamide |
| F2756-0879 | 4-(2,5-dioxopyrrolidin-1-yl)-N-(6-ethyl-1,3-benzothiazol-2-yl)-N-[2-(morpholin-4-yl)ethyl]benzamide hydrochloride |
| F3385-2010 | 8-[(dimethylamino)methyl]-7-hydroxy-3-(4-methoxyphenyl)-4H-chromen-4-one |
| F2515-2511 | 2-(2,5-dioxopyrrolidin-1-yl)-N-(4-methoxy-7-methyl-1,3-benzothiazol-2-yl)-N-[(pyridin-3-yl)methyl]acetamide |
| F3385-4424 | 3-(4-hydroxyphenyl)-5,7-dimethoxy-4H-chromen-4-one |
| F2736-1881 | 4-(2,5-dioxopyrrolidin-1-yl)-N-(6-fluoro-1,3-benzothiazol-2-yl)-N-[3-(morpholin-4-yl)propyl]benzamide hydrochloride |
| F5614-0649 | 1-(2-{4-[(1-benzyl-1H-1,3-benzodiazol-2-yl)methyl]piperazin-1-yl}-2-oxoethyl)pyrrolidine-2,5-dione |
| F2880-0923 | 2-chloro-N-[2-(dimethylamino)-2-(4-fluorophenyl)ethyl]-4-nitrobenzamide |
| F3386-3208 | 5-(butylamino)-4-chloro-2-phenyl-2,3-dihydropyridazin-3-one |
| F6064-4996 | N-(3,4-dichlorophenyl)-3-[3-(thiophen-3-yl)-1,2,4-oxadiazol-5-yl]azetidine-1-carboxamide |
| F2707-0450 | N-(3,4-dichlorophenyl)-1-(pyridin-3-yl)-1H,2H,3H,4H-pyrrolo[1,2-a]pyrazine-2-carboxamide |
| F2707-0484 | N-(3,4-dichlorophenyl)-1-(pyridin-4-yl)-1H,2H,3H,4H-pyrrolo[1,2-a]pyrazine-2-carboxamide |
| F2191-0179 | 2-chloropyridine-3-carboxylic acid |
| F3115-0092 | 2-{[1-(3-chloro-2-methylphenyl)-2,5-dioxopyrrolidin-3-yl]sulfanyl}pyridine-3-carboxylic acid |
| F1614-0007 | 8-bromo-7-(2-ethoxyethyl)-1,3-dimethyl-2,3,6,7-tetrahydro-1H-purine-2,6-dione |
| F0526-1893 | N-[3-(1,3-benzothiazol-2-yl)-6-ethyl-4H,5H,6H,7H-thieno[2,3-c]pyridin-2-yl]-2-(2,5-dioxopyrrolidin-1-yl)acetamide hydrochloride |
| F3325-0017 | 2-({1-[(4-methylphenyl)methyl]-2,5-dioxopyrrolidin-3-yl}sulfanyl)pyridine-3-carboxylic acid |
| F2783-0439 | 2-chloro-4-nitro-N-[3-(2-oxopyrrolidin-1-yl)phenyl]benzamide |
| F2783-0088 | 2-chloro-4-nitro-N-[4-(2-oxopyrrolidin-1-yl)phenyl]benzamide |
| F2784-0552 | 2-chloro-N-[4-methyl-3-(2-oxopiperidin-1-yl)phenyl]-4-nitrobenzamide |
| F3023-0928 | 3-{[4-(dimethylamino)phenyl]amino}-1-phenylpyrrolidine-2,5-dione |
| F0466-0056 | N-(2H-1,3-benzodioxol-5-yl)-3-(2,5-dioxopyrrolidin-1-yl)benzamide |
| F2167-4302 | 4-tert-butyl-3-hydroxybenzaldehyde |
| F5939-1203 | 1-(3-{4-[(4-chloro-1,3-benzothiazol-2-yl)oxy]piperidine-1-carbonyl}phenyl)pyrrolidine-2,5-dione |
| F5461-0265 | 1-[4-(4-{[4-(4-fluorophenyl)-1,3-thiazol-2-yl]methyl}piperazine-1-carbonyl)phenyl]pyrrolidine-2,5-dione hydrochloride |
| F3023-0933 | 3-{[4-(dimethylamino)phenyl]amino}-1-(2-methylphenyl)pyrrolidine-2,5-dione |
| F0675-0110 | 2-(1H-indol-3-yl)-2-oxo-N-pentylacetamide |
| F2880-1008 | 2-chloro-N-[2-(4-fluorophenyl)-2-(morpholin-4-yl)ethyl]-4-nitrobenzamide |
| F3139-2868 | 3-(2,3-dihydro-1,4-benzodioxin-6-yl)-7-hydroxy-8-methyl-4H-chromen-4-one |
| F3205-0001 | 3-[(2H-1,3-benzodioxol-5-yl)amino]-1-(2-methylphenyl)pyrrolidine-2,5-dione |
| F2666-0121 | 2-chloro-N-{3-methyl-2-oxo-1-azatricyclo[6.3.1.0^{4,12}]dodeca-4,6,8(12)-trien-6-yl}-4-nitrobenzamide |
| F2738-2186 | N-(4,7-dimethoxy-1,3-benzothiazol-2-yl)-2-(2,5-dioxopyrrolidin-1-yl)-N-[(pyridin-2-yl)methyl]acetamide |
| F3205-0025 | 2-{[1-(3-chloro-4-methylphenyl)-2,5-dioxopyrrolidin-3-yl]sulfanyl}pyridine-3-carboxylic acid |
| F6403-2768 | N-(3,4-dichlorophenyl)-4-({imidazo[1,2-a]pyridin-3-yl}methyl)piperazine-1-carboxamide |
| F0035-0009 | 1-(adamantan-1-yl)propan-1-ol |
| F0396-0213 | 4-(2,5-dioxopyrrolidin-1-yl)-N-[2-(4-methoxyphenyl)-2H,4H,6H-thieno[3,4-c]pyrazol-3-yl]benzamide |
| F2211-0002 | 6-amino-2-(2-methylphenyl)-4H-chromen-4-one |
| F0327-0301 | 4-(2,5-dioxopyrrolidin-1-yl)-N-(6-acetamido-1,3-benzothiazol-2-yl)benzamide |
| F3385-1184 | 7-hydroxy-3-phenyl-8-[(pyrrolidin-1-yl)methyl]-4H-chromen-4-one |
| F0921-1014 | 3-[2-(4-methylphenyl)-2-oxoethyl]-1,3-dihydro-2-benzofuran-1-one |
| F1065-0330 | 3-[(4-methoxyphenyl)amino]-1-phenylpyrrolidine-2,5-dione |
| F1065-0594 | 3-[(4-fluorophenyl)amino]-1-[4-(morpholin-4-yl)phenyl]pyrrolidine-2,5-dione |
| F1190-0476 | 3-(3,4-dimethoxyphenyl)-7-hydroxy-2-methyl-4H-chromen-4-one |
| F1065-0444 | 1-(2-fluorophenyl)-3-{[4-(morpholin-4-yl)phenyl]amino}pyrrolidine-2,5-dione |
| F3023-0977 | 1-(3,5-dimethylphenyl)-3-{[4-(morpholin-4-yl)phenyl]amino}pyrrolidine-2,5-dione |
| F0327-0384 | N-(3-acetylphenyl)-4-(2,5-dioxopyrrolidin-1-yl)benzamide |
| F1383-0022 | 2-{[1-(2H-1,3-benzodioxol-5-yl)-2,5-dioxopyrrolidin-3-yl]sulfanyl}pyridine-3-carboxylic acid |
| F2668-0051 | 2-chloro-4-nitro-N-{2-oxo-1-azatricyclo[7.3.1.0^{5,13}]trideca-5,7,9(13)-trien-7-yl}benzamide |
| F3023-0990 | 1-(2-fluorophenyl)-3-{[4-(piperidin-1-yl)phenyl]amino}pyrrolidine-2,5-dione |
| F3188-0115 | 3-[(2H-1,3-benzodioxol-5-yl)amino]-1-phenylpyrrolidine-2,5-dione |
| F0919-4623 | 6-methoxy-2H-1,3-benzodioxole-5-carbaldehyde |
| F1383-0013 | 2-{[1-(4-ethoxyphenyl)-2,5-dioxopyrrolidin-3-yl]sulfanyl}pyridine-3-carboxylic acid |
| F2756-0977 | 4-(2,5-dioxopyrrolidin-1-yl)-N-(6-methoxy-1,3-benzothiazol-2-yl)-N-[2-(morpholin-4-yl)ethyl]benzamide hydrochloride |
| F0799-0088 | 8-bromo-7-hexyl-1,3-dimethyl-2,3,6,7-tetrahydro-1H-purine-2,6-dione |
| F9994-5178 | 3-hydroxy-2-methoxybenzaldehyde |
| F3139-1207 | 7-hydroxy-3-(4-methoxyphenyl)-4H-chromen-4-one |
| F3144-0137 | 8-bromo-3,7-dimethyl-1-(prop-2-en-1-yl)-2,3,6,7-tetrahydro-1H-purine-2,6-dione |
| F6064-5505 | N-(3,4-dichlorophenyl)-3-{3-[(2-methoxyphenyl)methyl]-1,2,4-oxadiazol-5-yl}azetidine-1-carboxamide |
| F2191-0011 | 6-aminonaphthalene-2-carboxylic acid |
| F0327-0385 | N-(4-acetylphenyl)-4-(2,5-dioxopyrrolidin-1-yl)benzamide |
| F3385-0097 | 7-hydroxy-3-(4-methoxyphenyl)-8-[(pyrrolidin-1-yl)methyl]-4H-chromen-4-one |
| F2121-0053 | (2E)-3-(2,5-dichlorophenyl)prop-2-enoic acid |
| F3129-0236 | 2-{4-methoxy-6-methyl-2H,5H,6H,7H,8H-[1,3]dioxolo[4,5-g]isoquinolin-5-yl}-1-phenylethan-1-one |
| F0526-1896 | N-[3-(1,3-benzothiazol-2-yl)-6-(propan-2-yl)-4H,5H,6H,7H-thieno[2,3-c]pyridin-2-yl]-2-(2,5-dioxopyrrolidin-1-yl)acetamide hydrochloride |
| F1065-0020 | 1-(2,3-dimethylphenyl)-3-{[4-(morpholin-4-yl)phenyl]amino}pyrrolidine-2,5-dione |
| F2113-0600 | 6-hydroxy-2H-1,3-benzodioxole-5-carbaldehyde |
| F3023-0294 | 2-{[2,5-dioxo-1-(2-phenylethyl)pyrrolidin-3-yl]sulfanyl}pyridine-3-carboxylic acid |
| F3023-0411 | 3-[(4-methoxyphenyl)amino]-1-(3-methylphenyl)pyrrolidine-2,5-dione |
| F1166-0172 | 3-(2,5-dioxopyrrolidin-1-yl)-N-[4-(5,6,7,8-tetrahydronaphthalen-2-yl)-1,3-thiazol-2-yl]benzamide |
| F0758-0046 | 7-hydroxy-3-(4-methoxyphenyl)-2-methyl-4H-chromen-4-one |
| F1268-0176 | 1-(4-methylphenyl)-3-(phenylamino)pyrrolidine-2,5-dione |
| F3023-0917 | 1-butyl-3-[4-(4-fluorophenyl)piperazin-1-yl]pyrrolidine-2,5-dione |
| F3023-0298 | 2-{[1-(2-ethoxyphenyl)-2,5-dioxopyrrolidin-3-yl]sulfanyl}pyridine-3-carboxylic acid |
| F2211-0001 | 6-amino-2-phenyl-4H-chromen-4-one |
| F2783-0322 | 2-chloro-N-[3-methoxy-4-(2-oxopyrrolidin-1-yl)phenyl]-4-nitrobenzamide |
| F2783-0673 | 2-chloro-N-[4-methoxy-3-(2-oxopyrrolidin-1-yl)phenyl]-4-nitrobenzamide |
| F0222-0029 | 1-(4-fluorophenyl)-3-{[4-(morpholin-4-yl)phenyl]amino}pyrrolidine-2,5-dione |
| F3095-4413 | 7-methoxy-3-(2-methoxyphenyl)-4H-chromen-4-one |
| F3205-0103 | 3-[(2H-1,3-benzodioxol-5-yl)amino]-1-(4-hydroxyphenyl)pyrrolidine-2,5-dione |
| F2266-0311 | 2-chloro-4-nitro-N-(2-oxo-1,2,3,4-tetrahydroquinolin-6-yl)benzamide |
| F2146-0754 | 4,7-dimethoxy-3-(2-methoxyethyl)-2,3-dihydro-1,3-benzothiazol-2-imine; 4-methylbenzene-1-sulfonic acid |
| F2784-0088 | 2-chloro-4-nitro-N-[4-(2-oxopiperidin-1-yl)phenyl]benzamide |
| F3228-0143 | 7-hydroxy-3-(4-methoxyphenyl)-2,8-dimethyl-4H-chromen-4-one |
| F3023-0946 | 3-{[4-(dimethylamino)phenyl]amino}-1-(4-methylphenyl)pyrrolidine-2,5-dione |
| F2146-0748 | 4-methylbenzene-1-sulfonic acid; 6-fluoro-3-(2-methoxyethyl)-2,3-dihydro-1,3-benzothiazol-2-imine |
| F2146-0751 | 4-fluoro-3-(2-methoxyethyl)-2,3-dihydro-1,3-benzothiazol-2-imine; 4-methylbenzene-1-sulfonic acid |
| F2784-0669 | 2-chloro-N-[4-methoxy-3-(2-oxopiperidin-1-yl)phenyl]-4-nitrobenzamide |
| F1065-0258 | 3-[(4-fluorophenyl)amino]-1-(2-methoxyphenyl)pyrrolidine-2,5-dione |
| F1065-0329 | 1-(2-methoxyphenyl)-3-[(4-methylphenyl)amino]pyrrolidine-2,5-dione |
| F2211-0005 | 6-amino-2-(2-fluorophenyl)-4H-chromen-4-one |
| F3023-0618 | 2-({2,5-dioxo-1-[4-(piperidin-1-yl)phenyl]pyrrolidin-3-yl}sulfanyl)pyridine-3-carboxylic acid |
| F3023-0942 | 3-{[4-(dimethylamino)phenyl]amino}-1-(4-fluorophenyl)pyrrolidine-2,5-dione |
| F3385-4420 | 8-[(dimethylamino)methyl]-7-hydroxy-5-methoxy-2-phenyl-4H-chromen-4-one |
| F0349-4255 | 3,6-dihydroxy-9H-xanthen-9-one |
| F3188-0124 | 3-[(2H-1,3-benzodioxol-5-yl)amino]-1-(3-methylphenyl)pyrrolidine-2,5-dione |
| F0030-0090 | 1-benzyl-3-{[4-(morpholin-4-yl)phenyl]amino}pyrrolidine-2,5-dione |
| F2880-0498 | 2-chloro-N-[2-(4-methylphenyl)-2-(morpholin-4-yl)ethyl]-4-nitrobenzamide |
| F3385-2564 | 3-(3,4-dimethoxyphenyl)-8-[(dimethylamino)methyl]-7-hydroxy-4H-chromen-4-one |
| F6442-5567 | 2-(2,4-dichlorophenoxy)-N-[4-(4-methoxypiperidin-1-yl)phenyl]acetamide |
| F0440-0371 | 2-(2,5-dioxopyrrolidin-1-yl)-N-(2-phenoxyphenyl)acetamide |
| F3023-0619 | 2-{[1-(3-chloro-4-fluorophenyl)-2,5-dioxopyrrolidin-3-yl]sulfanyl}pyridine-3-carboxylic acid |
| F3228-0134 | 7-hydroxy-3-(4-methoxyphenyl)-8-methyl-4H-chromen-4-one |
| F5834-4901 | 2-{4-[2-(2,6-dioxopiperidin-1-yl)ethanesulfonamido]phenyl}-N-[(thiophen-2-yl)methyl]acetamide |
| F3228-0133 | 7-hydroxy-3-(4-methoxyphenyl)-5-methyl-4H-chromen-4-one |
| F0196-0125 | 3-(2,3-dihydro-1,4-benzodioxin-6-yl)-7-hydroxy-4H-chromen-4-one |
| F3385-1000 | 2-{[3-(2,3-dihydro-1,4-benzodioxin-6-yl)-4-oxo-4H-chromen-7-yl]oxy}acetonitrile |
| F0466-0040 | 3-(2,5-dioxopyrrolidin-1-yl)-N-(2-phenoxyphenyl)benzamide |
| F2146-0728 | 3-(2-ethoxyethyl)-6-methoxy-2,3-dihydro-1,3-benzothiazol-2-imine; 4-methylbenzene-1-sulfonic acid |
| F1065-0606 | 1-benzyl-3-(phenylamino)pyrrolidine-2,5-dione |
| F3023-0978 | 1-(3-chloro-2-methylphenyl)-3-{[4-(morpholin-4-yl)phenyl]amino}pyrrolidine-2,5-dione |
| F3023-0378 | 1-butyl-3-[(4-ethylphenyl)amino]pyrrolidine-2,5-dione |
| F0902-0374 | 1-(2-chlorophenyl)-3-{[4-(morpholin-4-yl)phenyl]amino}pyrrolidine-2,5-dione |
| F1424-0124 | 2-[(2,4-dichlorophenoxy)methyl]-5-(3,4,5-trimethoxyphenyl)-1,3,4-oxadiazole |
| F3325-0136 | 1-(2-chlorophenyl)-3-{[4-(dimethylamino)phenyl]amino}pyrrolidine-2,5-dione |
| F2146-0749 | 3-(2-methoxyethyl)-2,3-dihydro-1,3-benzothiazol-2-imine; 4-methylbenzene-1-sulfonic acid |
| F3139-0815 | 2-[(4-oxo-3-phenyl-4H-chromen-7-yl)oxy]acetonitrile |
| F2211-0003 | 6-amino-2-(3-methylphenyl)-4H-chromen-4-one |
| F3023-1012 | 1-(2,3-dimethylphenyl)-3-{[4-(piperidin-1-yl)phenyl]amino}pyrrolidine-2,5-dione |
| F6064-3978 | N-(3,4-dichlorophenyl)-3-{3-[3-(methylcarbamoyl)phenyl]-1,2,4-oxadiazol-5-yl}azetidine-1-carboxamide |
| F3023-0404 | 3-[(4-methylphenyl)amino]-1-[4-(piperidin-1-yl)phenyl]pyrrolidine-2,5-dione |
| F2146-0750 | 4-ethoxy-3-(2-methoxyethyl)-2,3-dihydro-1,3-benzothiazol-2-imine; 4-methylbenzene-1-sulfonic acid |
| F9995-4161 | 3-(4-methylphenoxy)benzoic acid |
| F1967-2640 | 5-(bromomethyl)-2-(2-fluorophenyl)-1,3-oxazole |
| F2146-0727 | 4-methylbenzene-1-sulfonic acid; methyl 3-(2-ethoxyethyl)-2-imino-2,3-dihydro-1,3-benzothiazole-6-carboxylate |
| F0758-0033 | 7-hydroxy-2-methyl-3-phenyl-4H-chromen-4-one |
| F3023-1003 | 1-(4-methylphenyl)-3-{[4-(piperidin-1-yl)phenyl]amino}pyrrolidine-2,5-dione |
| F1799-0313 | 1-(3-chlorophenyl)-3-(4-phenylpiperazin-1-yl)pyrrolidine-2,5-dione |
| F3205-0099 | 3-{[4-(dimethylamino)phenyl]amino}-1-(4-ethylphenyl)pyrrolidine-2,5-dione |
| F1799-0316 | 1-(4-chlorophenyl)-3-(4-phenylpiperazin-1-yl)pyrrolidine-2,5-dione |
| F3115-0020 | 2-{[1-(2-bromo-4-methylphenyl)-2,5-dioxopyrrolidin-3-yl]sulfanyl}pyridine-3-carboxylic acid |
| F3385-0965 | 7-methoxy-3-(4-methoxyphenyl)-4H-chromen-4-one |
| F1065-0552 | 1-(3-chlorophenyl)-3-{[4-(morpholin-4-yl)phenyl]amino}pyrrolidine-2,5-dione |
| F3385-0122 | 7-hydroxy-3-(4-methoxyphenyl)-8-[(piperidin-1-yl)methyl]-4H-chromen-4-one |
| F3139-0461 | 2-{[3-(2-methoxyphenyl)-4-oxo-4H-chromen-7-yl]oxy}acetonitrile |
| F3023-0029 | 2-{[1-(2,3-dichlorophenyl)-2,5-dioxopyrrolidin-3-yl]sulfanyl}pyridine-3-carboxylic acid |
| F3139-1694 | 3-(3,4-dimethoxyphenyl)-5,7-dihydroxy-2-methyl-4H-chromen-4-one |
| F2211-0006 | 6-amino-2-(4-fluorophenyl)-4H-chromen-4-one |
| F0722-2692 | 2-chloro-N-[4-(morpholin-4-yl)phenyl]-4-nitrobenzamide |
| F3023-0934 | 3-{[4-(dimethylamino)phenyl]amino}-1-(2-methoxyphenyl)pyrrolidine-2,5-dione |
| F0270-0027 | 1-(2-chlorophenyl)-3-[(4-methoxyphenyl)amino]pyrrolidine-2,5-dione |
| F3023-0970 | 1-(4-acetylphenyl)-3-{[4-(morpholin-4-yl)phenyl]amino}pyrrolidine-2,5-dione |
| F3023-0973 | 1-(4-ethylphenyl)-3-{[4-(morpholin-4-yl)phenyl]amino}pyrrolidine-2,5-dione |
| F2146-0716 | 4-methylbenzene-1-sulfonic acid; methyl 3-ethyl-2-imino-2,3-dihydro-1,3-benzothiazole-6-carboxylate |
| F3023-0626 | 2-{[1-(3-bromophenyl)-2,5-dioxopyrrolidin-3-yl]sulfanyl}pyridine-3-carboxylic acid |
| F1199-0110 | 1-(3-methoxyphenyl)-3-{[4-(morpholin-4-yl)phenyl]amino}pyrrolidine-2,5-dione |
| F2794-0088 | 2-chloro-N-[4-(1,1-dioxo-1lambda6,2-thiazinan-2-yl)phenyl]-4-nitrobenzamide |
| F0849-4319 | 1-(4-methoxyphenyl)-3-[(4-methoxyphenyl)amino]pyrrolidine-2,5-dione |
| F0158-0017 | 1-(4-methoxyphenyl)-3-{[4-(morpholin-4-yl)phenyl]amino}pyrrolidine-2,5-dione |
| F0220-0040 | 3-[(4-methoxyphenyl)amino]-1-[4-(morpholin-4-yl)phenyl]pyrrolidine-2,5-dione |
| F1199-0027 | 3-[(4-fluorophenyl)amino]-1-[4-(piperidin-1-yl)phenyl]pyrrolidine-2,5-dione |
| F2146-0730 | 3-(2-ethoxyethyl)-4,7-dimethoxy-2,3-dihydro-1,3-benzothiazol-2-imine; 4-methylbenzene-1-sulfonic acid |
| F3023-0610 | 2-{[1-(2,4-dichlorophenyl)-2,5-dioxopyrrolidin-3-yl]sulfanyl}pyridine-3-carboxylic acid |
| F0196-0113 | 3-(3,4-dihydro-2H-1,5-benzodioxepin-7-yl)-5,7-dihydroxy-2-methyl-4H-chromen-4-one |
| F0440-0366 | N-(2-benzoyl-4-methylphenyl)-2-(2,5-dioxopyrrolidin-1-yl)acetamide |
| F1383-0014 | 2-({1-[4-(acetyloxy)phenyl]-2,5-dioxopyrrolidin-3-yl}sulfanyl)pyridine-3-carboxylic acid |
| F2384-0011 | 4-chloro-N-(2-oxo-1-propyl-1,2,3,4-tetrahydroquinolin-6-yl)benzamide |
| F3205-0101 | 3-[(2H-1,3-benzodioxol-5-yl)amino]-1-[4-(dimethylamino)phenyl]pyrrolidine-2,5-dione |
| F3325-0135 | 1-benzyl-3-{[4-(dimethylamino)phenyl]amino}pyrrolidine-2,5-dione |
| F3023-1011 | 1-(4-acetylphenyl)-3-{[4-(piperidin-1-yl)phenyl]amino}pyrrolidine-2,5-dione |
| F1190-0490 | 5,7-dihydroxy-3-(4-methoxyphenyl)-2-methyl-4H-chromen-4-one |
| F5831-0290 | 2-(2,6-dioxopiperidin-1-yl)-N-{2-[1-(4-methylbenzenesulfonyl)piperidin-2-yl]ethyl}ethane-1-sulfonamide |
| F2146-0724 | 3-(2-ethoxyethyl)-6-methyl-2,3-dihydro-1,3-benzothiazol-2-imine; 4-methylbenzene-1-sulfonic acid |
| F3030-0002 | 3-[(4-ethylphenyl)amino]-1-(2-fluorophenyl)pyrrolidine-2,5-dione |
| F0030-0094 | 1-(2-chlorophenyl)-3-[(4-fluorophenyl)amino]pyrrolidine-2,5-dione |
| F3023-0938 | 1-(3-chlorophenyl)-3-{[4-(dimethylamino)phenyl]amino}pyrrolidine-2,5-dione |
| F3023-0953 | 1-(3-chloro-2-methylphenyl)-3-{[4-(dimethylamino)phenyl]amino}pyrrolidine-2,5-dione |
| F0915-6107 | 1-(2,4-dihydroxyphenyl)-2-(3,4-dimethoxyphenyl)ethan-1-one |
| F0196-0870 | 7-hydroxy-3-phenyl-4H-chromen-4-one |
| F2211-0018 | N-[2-(4-fluorophenyl)-4-oxo-4H-chromen-6-yl]acetamide |
| F2106-0046 | 7-hydroxy-5-methoxy-2-phenyl-4H-chromen-4-one |
| F2880-0668 | 2-chloro-N-[2-(3-methoxyphenyl)-2-(morpholin-4-yl)ethyl]-4-nitrobenzamide |
| F3019-0019 | 3-[(4-ethylphenyl)amino]-1-(2-methoxyphenyl)pyrrolidine-2,5-dione |
| F3023-0957 | 3-{[4-(morpholin-4-yl)phenyl]amino}-1-(1-phenylethyl)pyrrolidine-2,5-dione |
| F3139-2717 | 3-(4-methoxyphenyl)-4-oxo-4H-chromen-7-yl acetate |
| F2146-0753 | 3-(2-methoxyethyl)-5,6-dimethyl-2,3-dihydro-1,3-benzothiazol-2-imine; 4-methylbenzene-1-sulfonic acid |
| F0220-0047 | 1-(4-chlorophenyl)-3-{[4-(morpholin-4-yl)phenyl]amino}pyrrolidine-2,5-dione |
| F2146-0752 | 3-(2-methoxyethyl)-5,7-dimethyl-2,3-dihydro-1,3-benzothiazol-2-imine; 4-methylbenzene-1-sulfonic acid |
| F1190-0497 | 2-(2-fluorophenyl)-6-methoxy-3,4-dihydro-2H-1-benzopyran-4-one |
| F3023-0398 | 1-benzyl-3-[(4-methylphenyl)amino]pyrrolidine-2,5-dione |
| F0425-0017 | 3-[(4-methylphenyl)amino]-1-(3-nitrophenyl)pyrrolidine-2,5-dione |
| F0758-0105 | 8-methyl-4-oxo-3-phenyl-4H-chromen-7-yl acetate |
| F2146-0723 | 3-(2-ethoxyethyl)-2,3-dihydro-1,3-benzothiazol-2-imine; 4-methylbenzene-1-sulfonic acid |
| F1800-0170 | N-(4-butylphenyl)-2-(2-methyl-3,5-dioxothiomorpholin-4-yl)acetamide |
| F2384-0077 | 3-chloro-N-(2-oxo-1-propyl-1,2,3,4-tetrahydroquinolin-6-yl)benzamide |
| F3023-0612 | 2-{[1-(3,4-dichlorophenyl)-2,5-dioxopyrrolidin-3-yl]sulfanyl}pyridine-3-carboxylic acid |
| F3023-0918 | 1-benzyl-3-[4-(4-fluorophenyl)piperazin-1-yl]pyrrolidine-2,5-dione |
| F3139-0996 | 2-{[3-(2,3-dihydro-1,4-benzodioxin-6-yl)-6-ethyl-4-oxo-4H-chromen-7-yl]oxy}acetamide |
| F3385-4418 | 6-[(dimethylamino)methyl]-5-hydroxy-7-methoxy-2-phenyl-4H-chromen-4-one |
| F2212-0015 | N-[2-(4-methylphenyl)-4-oxo-4H-chromen-7-yl]acetamide |
| F2992-0058 | N-[3-(1,3-benzothiazol-2-yl)thiophen-2-yl]-3-(2,5-dioxopyrrolidin-1-yl)benzamide |
| F3139-0714 | 5-hydroxy-3-(4-hydroxyphenyl)-7-(propan-2-yloxy)-4H-chromen-4-one |
| F0196-0676 | 4-oxo-3-phenyl-4H-chromen-7-yl acetate |
| F1065-0413 | 1-(4-methoxyphenyl)-3-{[4-(piperidin-1-yl)phenyl]amino}pyrrolidine-2,5-dione |
| F3385-3754 | 8-[(diethylamino)methyl]-7-hydroxy-3-(4-methoxyphenyl)-4H-chromen-4-one |
| F3139-0913 | 6-methoxy-2-(4-methylphenyl)-3,4-dihydro-2H-1-benzopyran-4-one |
| F2018-1698 | N-[2-(dimethylamino)ethyl]-N-{10,13-dioxa-4-thia-6-azatricyclo[7.4.0.0^{3,7}]trideca-1,3(7),5,8-tetraen-5-yl}-4-(2,5-dioxopyrrolidin-1-yl)benzamide hydrochloride |
| F2146-0725 | 3-(2-ethoxyethyl)-6-fluoro-2,3-dihydro-1,3-benzothiazol-2-imine; 4-methylbenzene-1-sulfonic acid |
| F1199-0125 | 2-({1-[4-(methoxycarbonyl)phenyl]-2,5-dioxopyrrolidin-3-yl}sulfanyl)pyridine-3-carboxylic acid |
| F0758-0031 | 2,8-dimethyl-4-oxo-3-phenyl-4H-chromen-7-yl acetate |
| F2515-1622 | N-{4,6-dioxa-10-thia-12-azatricyclo[7.3.0.0^{3,7}]dodeca-1(9),2,7,11-tetraen-11-yl}-2-(2,5-dioxopyrrolidin-1-yl)-N-[(pyridin-3-yl)methyl]acetamide |
| F0001-2524 | 4-cyclobutylbenzaldehyde |
| F0200-0002 | 1-benzyl-3-[(4-fluorophenyl)amino]pyrrolidine-2,5-dione |
| F0327-0386 | 4-(2,5-dioxopyrrolidin-1-yl)-N-(2-methoxy-4-nitrophenyl)benzamide |
| F3139-0700 | 2-{[3-(4-chlorophenyl)-4-oxo-4H-chromen-7-yl]oxy}acetonitrile |
| F3292-0445 | 2-{4-methoxy-6-methyl-2H,5H,6H,7H,8H-[1,3]dioxolo[4,5-g]isoquinolin-5-yl}-1-(3-methoxyphenyl)ethan-1-one |
| F3139-0614 | 2-(3,4-dimethoxyphenyl)-6-fluoro-4H-chromen-4-one |
| F3139-0622 | 2-(2-methoxyphenyl)-6-methyl-4H-chromen-4-one |
| F0758-0028 | 7-methoxy-2-methyl-3-phenyl-4H-chromen-4-one |
| F3205-0036 | 1-[(4-methylphenyl)methyl]-3-{[4-(morpholin-4-yl)phenyl]amino}pyrrolidine-2,5-dione |
| F3385-0893 | 7-ethoxy-3-(4-methoxyphenyl)-4H-chromen-4-one |
| F1800-0200 | ethyl 2-[2-(2-methyl-3,5-dioxothiomorpholin-4-yl)acetamido]-4,5,6,7-tetrahydro-1-benzothiophene-3-carboxylate |
| F3205-0015 | 3-[(4-methoxyphenyl)amino]-1-[(4-methylphenyl)methyl]pyrrolidine-2,5-dione |
| F3097-5834 | 2-chloro-N-(2,6-diethylphenyl)-4-nitrobenzamide |
| F0196-1328 | 3-(2,3-dihydro-1,4-benzodioxin-6-yl)-4-oxo-4H-chromen-7-yl acetate |
| F1175-0183 | 2-chloro-N-(1,5-dimethyl-3-oxo-2-phenyl-2,3-dihydro-1H-pyrazol-4-yl)-4-nitrobenzamide |
| F1799-0488 | 1-benzyl-3-(4-benzylpiperazin-1-yl)pyrrolidine-2,5-dione |
| F0327-0368 | ethyl 4-[4-(2,5-dioxopyrrolidin-1-yl)benzamido]benzoate |
| F1533-0348 | N-(3-methoxyphenyl)piperidine-1-carbothioamide |
| F1736-0003 | N-(2-chloro-4-nitrophenyl)-2,3-dihydro-1,4-benzodioxine-2-carboxamide |
| F3023-0301 | 2-({1-[4-(ethoxycarbonyl)phenyl]-2,5-dioxopyrrolidin-3-yl}sulfanyl)pyridine-3-carboxylic acid |
| F0758-0027 | 7-methoxy-2,8-dimethyl-3-phenyl-4H-chromen-4-one |
| F2784-0320 | 2-chloro-N-[3-methoxy-4-(2-oxopiperidin-1-yl)phenyl]-4-nitrobenzamide |
| F3205-0063 | 2-({2,5-dioxo-1-[4-(trifluoromethoxy)phenyl]pyrrolidin-3-yl}sulfanyl)pyridine-3-carboxylic acid |
| F3385-1001 | 2-{[3-(4-bromophenyl)-4-oxo-4H-chromen-7-yl]oxy}acetonitrile |
| F0196-0909 | 3-phenyl-7-(propan-2-yloxy)-4H-chromen-4-one |
| F1799-0249 | 1-(4-bromophenyl)-3-[(4-methoxyphenyl)amino]pyrrolidine-2,5-dione |
| F6195-7343 | N-(3,4-dichlorophenyl)-2-(furan-3-amido)-4H,5H,6H,7H-[1,3]thiazolo[5,4-c]pyridine-5-carboxamide |
| F1638-0034 | 6-(4-chloro-3-nitrophenyl)-5-methyl-2,3,4,5-tetrahydropyridazin-3-one |
| F2106-0103 | 2-[(4-oxo-3-phenyl-4H-chromen-7-yl)oxy]acetic acid |
| F3139-1115 | 4-oxo-3-phenyl-4H-chromen-7-yl 2,2-dimethylpropanoate |
| F3215-0209 | N-(4-chloro-2-nitrophenyl)-2,3-dihydro-1,4-benzodioxine-2-carboxamide |
| F3385-4073 | 3-(4-methoxyphenyl)-7-[2-(morpholin-4-yl)ethoxy]-4H-chromen-4-one |
| F0850-4773 | 3-(2-methoxyphenyl)-4-oxo-4H-chromen-7-yl propanoate |
| F2874-0131 | 2-chloro-N-[4-(1-methyl-6-oxo-1,6-dihydropyridazin-3-yl)phenyl]-4-nitrobenzamide |
| F0466-0262 | 2-(2,5-dioxopyrrolidin-1-yl)-N-[4-(4-nitrothiophen-2-yl)-1,3-thiazol-2-yl]acetamide |
| F0911-3695 | 3-(2-methoxyphenyl)-4-oxo-4H-chromen-7-yl 2,2-dimethylpropanoate |
| F2384-0386 | 4-chloro-N-[1-(2-methylpropyl)-2-oxo-1,2,3,4-tetrahydroquinolin-6-yl]benzamide |
| F3323-0579 | 4-[2-(5-fluoro-2-methoxyphenyl)-2-oxoethyl]-2,3,4,5-tetrahydro-1,4-benzoxazepine-3,5-dione |
| F3325-0126 | 3-(4-benzylpiperazin-1-yl)-1-(2-ethylphenyl)pyrrolidine-2,5-dione |
| F3139-1062 | 4-oxo-3-phenyl-4H-chromen-7-yl propanoate |
| F3385-1569 | 4-oxo-3-phenyl-4H-chromen-7-yl N,N-dimethylcarbamate |
| F3115-0070 | 3-[(4-methoxyphenyl)amino]-1-(2-methyl-5-nitrophenyl)pyrrolidine-2,5-dione |
| F3139-0691 | 7-ethoxy-3-(4-methoxyphenyl)-2-methyl-4H-chromen-4-one |
| F8885-7819 | 2-[(chloromethyl)sulfanyl]-1,3-benzothiazole |
| F3139-0931 | 2-(6-bromo-2,4-dihydro-1,3-benzodioxin-8-yl)-4H-chromen-4-one |
| F0196-0180 | 3-(4-chlorophenyl)-7-hydroxy-4H-chromen-4-one |
| F1105-0218 | N-(1,3-dioxo-2,3-dihydro-1H-isoindol-5-yl)-2-nitrobenzamide |
| F2764-0083 | 4-chloro-N-({1,4-dioxaspiro[4.4]nonan-2-yl}methyl)-3-nitrobenzamide |
| F0196-0030 | 7-methoxy-3-phenyl-4H-chromen-4-one |
| F1065-0364 | 3-{[4-(morpholin-4-yl)phenyl]amino}-1-[3-(trifluoromethyl)phenyl]pyrrolidine-2,5-dione |
| F1065-0411 | 1-(4-ethoxyphenyl)-3-{[4-(morpholin-4-yl)phenyl]amino}pyrrolidine-2,5-dione |
| F1065-0769 | 3-{[4-(morpholin-4-yl)phenyl]amino}-1-[4-(trifluoromethyl)phenyl]pyrrolidine-2,5-dione |
| F3323-0624 | 4-[2-(2-methoxy-5-methylphenyl)-2-oxoethyl]-2,3,4,5-tetrahydro-1,4-benzoxazepine-3,5-dione |
| F5252-0296 | N-(3,4-dichlorophenyl)-4-{[1-(4-methoxyphenyl)-1H-1,2,3,4-tetrazol-5-yl]methyl}piperazine-1-carboxamide |
| F3023-0959 | 3-{[4-(morpholin-4-yl)phenyl]amino}-1-(2-phenylethyl)pyrrolidine-2,5-dione |
| F3325-0111 | 1-(3-chlorophenyl)-3-[4-(4-fluorophenyl)piperazin-1-yl]pyrrolidine-2,5-dione |
| F0486-1748 | 2-{4-[cyano(4-methoxyphenyl)methyl]piperazin-1-yl}-2-(4-methoxyphenyl)acetonitrile |
| F0758-0077 | 7-ethoxy-3-phenyl-4H-chromen-4-one |
| F3139-1444 | 3-(3,4-dimethoxyphenyl)-7-ethoxy-2-methyl-4H-chromen-4-one |
| F3385-1337 | 3-(4-methoxyphenyl)-4-oxo-4H-chromen-7-yl 2,2-dimethylpropanoate |
| F3325-0133 | 3-{[4-(dimethylamino)phenyl]amino}-1-(2-phenylethyl)pyrrolidine-2,5-dione |
| F3385-0858 | 3-(4-chlorophenyl)-4-oxo-4H-chromen-7-yl acetate |
| F3115-0096 | ethyl 4-[1-(naphthalen-1-yl)-2,5-dioxopyrrolidin-3-yl]piperazine-1-carboxylate |
| F2271-0010 | 4-chloro-N-[2-(1-methyl-1,2,3,4-tetrahydroquinolin-6-yl)ethyl]benzamide |
| F3188-0123 | 3-[(2H-1,3-benzodioxol-5-yl)amino]-1-(2-phenylethyl)pyrrolidine-2,5-dione |
| F3385-6145 | 7-methoxy-2-phenyl-4H-chromen-4-one |
| F3139-0612 | 6-chloro-2-(3,4-dimethoxyphenyl)-4H-chromen-4-one |
| F3023-0884 | 3-[(4-ethoxyphenyl)amino]-1-[4-(morpholin-4-yl)phenyl]pyrrolidine-2,5-dione |
| F0425-0019 | 3-[(4-methoxyphenyl)amino]-1-(3-nitrophenyl)pyrrolidine-2,5-dione |
| F5460-0026 | N-[1-(1,3-benzothiazol-2-yl)-3-methyl-1H-pyrazol-5-yl]-3-(2,5-dioxopyrrolidin-1-yl)benzamide |
| F0196-0669 | 3-(4-bromophenyl)-7-hydroxy-4H-chromen-4-one |
| F0715-0381 | ethyl (2Z)-2-{[2-(2,5-dioxopyrrolidin-1-yl)acetyl]imino}-3,4-dimethyl-2,3-dihydro-1,3-thiazole-5-carboxylate |
| F3023-0394 | 1-(2,4-dimethoxyphenyl)-3-[(4-ethylphenyl)amino]pyrrolidine-2,5-dione |
| F3139-0847 | methyl 2-[(4-oxo-3-phenyl-4H-chromen-7-yl)oxy]acetate |
| F3023-0974 | 1-(2,3-dichlorophenyl)-3-{[4-(morpholin-4-yl)phenyl]amino}pyrrolidine-2,5-dione |
| F3325-0121 | 1-[(2H-1,3-benzodioxol-5-yl)methyl]-3-(4-benzylpiperazin-1-yl)pyrrolidine-2,5-dione |
| F0440-0365 | N-(2-benzoyl-4-chlorophenyl)-2-(2,5-dioxopyrrolidin-1-yl)acetamide |
| F0191-5659 | N-(2-chloro-4-nitrophenyl)-2-methoxybenzamide |
| F0327-0387 | 4-(2,5-dioxopyrrolidin-1-yl)-N-(2-methoxy-5-nitrophenyl)benzamide |
| F0196-0181 | 3-(4-chlorophenyl)-5,7-dihydroxy-4H-chromen-4-one |
| F3325-0119 | 3-(4-benzylpiperazin-1-yl)-1-(4-ethylphenyl)pyrrolidine-2,5-dione |
| F1199-0131 | 1-(2-ethoxyphenyl)-3-{[4-(morpholin-4-yl)phenyl]amino}pyrrolidine-2,5-dione |
| F1190-0477 | 3-(3,4-dimethoxyphenyl)-2-methyl-4-oxo-4H-chromen-7-yl acetate |
| F1457-0030 | 2-{[1-(2-butoxyphenyl)-2,5-dioxopyrrolidin-3-yl]sulfanyl}pyridine-3-carboxylic acid |
| F2545-0219 | 4-chloro-N-(1,1-dioxo-1lambda6-thiolan-3-yl)-3-nitrobenzamide |
| F3139-0449 | 3-(2-methoxyphenyl)-7-[(2-methylprop-2-en-1-yl)oxy]-4H-chromen-4-one |
| F3139-0860 | 3-(4-chlorophenyl)-7-methoxy-4H-chromen-4-one |
| F1716-0347 | 2-sulfanylpyridine-3-carboxylic acid |
| F6374-0900 | 1-{2-[(4-{[(4-methyl-1,3-thiazol-2-yl)sulfanyl]methyl}piperidin-1-yl)sulfonyl]ethyl}piperidine-2,6-dione |
| F0196-1335 | 3-(2,3-dihydro-1,4-benzodioxin-6-yl)-6-ethyl-4-oxo-4H-chromen-7-yl acetate |
| F3099-7561 | 4-chloro-2-nitro-N-(2-phenylethyl)benzamide |
| F3139-0620 | 6-chloro-2-(2-methoxyphenyl)-4H-chromen-4-one |
| F0902-0905 | 4-(3-{[4-(morpholin-4-yl)phenyl]amino}-2,5-dioxopyrrolidin-1-yl)phenyl acetate |
| F0327-0389 | 4-(2,5-dioxopyrrolidin-1-yl)-N-(4-methoxy-2-nitrophenyl)benzamide |
| F1199-0083 | 3-{[4-(morpholin-4-yl)phenyl]amino}-1-(3-nitrophenyl)pyrrolidine-2,5-dione |
| F3323-0587 | 4-[2-(4-ethoxyphenyl)-2-oxoethyl]-2,3,4,5-tetrahydro-1,4-benzoxazepine-3,5-dione |
| F3023-0948 | 1-{4-[(difluoromethyl)sulfanyl]phenyl}-3-{[4-(dimethylamino)phenyl]amino}pyrrolidine-2,5-dione |
| F3139-0636 | 2-(2-fluorophenyl)-7-methoxy-4H-chromen-4-one |
| F3230-0028 | 3-(4-bromo-3,5-dimethyl-1H-pyrazol-1-yl)-1-[4-(2,4-dimethylbenzenesulfonyl)piperazin-1-yl]butan-1-one |
| F1537-0041 | 1-(2,4-dimethoxyphenyl)-3-{[4-(morpholin-4-yl)phenyl]amino}pyrrolidine-2,5-dione |
| F3385-4501 | 3-(3,4-dimethoxyphenyl)-7-[2-(4-methylpiperazin-1-yl)ethoxy]-4H-chromen-4-one dihydrochloride |
| F1593-0038 | 3-(3-{[4-(morpholin-4-yl)phenyl]amino}-2,5-dioxopyrrolidin-1-yl)phenyl acetate |
| F3323-0630 | 4-[2-(4-ethoxy-3-fluorophenyl)-2-oxoethyl]-2,3,4,5-tetrahydro-1,4-benzoxazepine-3,5-dione |
| F1199-0084 | 1-(2-bromo-4-methylphenyl)-3-{[4-(morpholin-4-yl)phenyl]amino}pyrrolidine-2,5-dione |
| F3323-0591 | 4-[2-(3-nitrophenyl)-2-oxoethyl]-2,3,4,5-tetrahydro-1,4-benzoxazepine-3,5-dione |
| F1383-0023 | 2-({1-[2-(3,4-dimethoxyphenyl)ethyl]-2,5-dioxopyrrolidin-3-yl}sulfanyl)pyridine-3-carboxylic acid |
| F0182-0152 | 4-(2-nitrophenyl)-6-oxo-2-sulfanyl-1,4,5,6-tetrahydropyridine-3-carbonitrile; 4-methylmorpholine |
| F1199-0099 | 1-(2-methyl-5-nitrophenyl)-3-{[4-(morpholin-4-yl)phenyl]amino}pyrrolidine-2,5-dione |
| F3139-1630 | 3-(4-bromophenyl)-4-oxo-4H-chromen-7-yl acetate |
| F3139-0633 | 6-bromo-2-(2-methoxyphenyl)-4H-chromen-4-one |
| F0196-0664 | 3-(4-bromophenyl)-7-methoxy-4H-chromen-4-one |
| F3205-0003 | tert-butyl 4-[2,5-dioxo-1-(2-phenylethyl)pyrrolidin-3-yl]piperazine-1-carboxylate |
| F1065-0214 | 1-(4-ethoxyphenyl)-3-{[4-(piperidin-1-yl)phenyl]amino}pyrrolidine-2,5-dione |
| F3023-1007 | 1-[4-(difluoromethoxy)phenyl]-3-{[4-(piperidin-1-yl)phenyl]amino}pyrrolidine-2,5-dione |
| F0196-0860 | 2-(6-bromo-2,4-dihydro-1,3-benzodioxin-8-yl)-7-methoxy-4H-chromen-4-one |
| F3385-2700 | 3-(4-methoxyphenyl)-4-oxo-4H-chromen-7-yl 2-methylpropanoate |
| F1065-0021 | 1-{4-[(difluoromethyl)sulfanyl]phenyl}-3-{[4-(morpholin-4-yl)phenyl]amino}pyrrolidine-2,5-dione |
| F2823-0077 | N-{1-tert-butyl-4-oxo-1H,4H,5H-pyrazolo[3,4-d]pyrimidin-5-yl}-4-chloro-3-nitrobenzamide |
| F0902-0375 | 3-[(4-methoxyphenyl)amino]-1-(2-propoxyphenyl)pyrrolidine-2,5-dione |
| F1219-1682 | 5-(3,4-dimethoxyphenyl)-2,3-dihydro-1,3,4-oxadiazole-2-thione |
| F1593-0044 | 1-[4-(difluoromethoxy)phenyl]-3-{[4-(morpholin-4-yl)phenyl]amino}pyrrolidine-2,5-dione |
| F3139-0846 | ethyl 2-[(4-oxo-3-phenyl-4H-chromen-7-yl)oxy]acetate |
| F6660-2140 | 3-(3-cyclopropyl-1,2,4-oxadiazol-5-yl)piperidine |
| F3385-3642 | 8-[(diethylamino)methyl]-3-(3,4-dimethoxyphenyl)-7-hydroxy-4H-chromen-4-one |
| F2763-0082 | 4-chloro-N-({1,4-dioxaspiro[4.5]decan-2-yl}methyl)-3-nitrobenzamide |
| F3325-0131 | methyl 4-(3-{[4-(dimethylamino)phenyl]amino}-2,5-dioxopyrrolidin-1-yl)benzoate |
| F3139-0832 | propan-2-yl 2-[(4-oxo-3-phenyl-4H-chromen-7-yl)oxy]acetate |
| F3385-0024 | 3-(4-bromophenyl)-7-ethoxy-4H-chromen-4-one |
| F2261-0203 | 2-chloro-N-(5-ethyl-3,3-dimethyl-4-oxo-2,3,4,5-tetrahydro-1,5-benzoxazepin-8-yl)-4-nitrobenzamide |
| F3385-5409 | 2-{[3-(3,4-dimethoxyphenyl)-4-oxo-4H-chromen-7-yl]oxy}acetic acid |
| F1175-0173 | 4-(2-chloro-4-nitrobenzamido)benzoic acid |
| F2769-0191 | 4-chloro-N-[1-(2-methylpropanoyl)-1,2,3,4-tetrahydroquinolin-6-yl]benzamide |
| F2271-0197 | 4-chloro-N-[2-(1-ethyl-1,2,3,4-tetrahydroquinolin-6-yl)ethyl]benzamide |
| F3205-0077 | 1-{4-[(difluoromethyl)sulfanyl]phenyl}-3-(4-phenylpiperazin-1-yl)pyrrolidine-2,5-dione |
| F5526-0149 | 2-chloro-N-[2-(4-fluorophenoxy)ethyl]-4-nitrobenzamide |
| F1199-0132 | 1-(2H-1,3-benzodioxol-5-yl)-3-{[4-(morpholin-4-yl)phenyl]amino}pyrrolidine-2,5-dione |
| F3139-0615 | 6-fluoro-2-(2-fluorophenyl)-4H-chromen-4-one |
| F3228-0186 | 5-hydroxy-4-oxo-2-phenyl-4H-chromen-7-yl N,N-dimethylcarbamate |
| F0440-0362 | 2-(2,5-dioxopyrrolidin-1-yl)-N-[(2E)-3-methyl-2,3-dihydro-1,3-benzothiazol-2-ylidene]acetamide |
| F3385-4502 | 3-(3,4-dimethoxyphenyl)-7-[2-(morpholin-4-yl)ethoxy]-4H-chromen-4-one |
| F1199-0088 | methyl 4-(3-{[4-(morpholin-4-yl)phenyl]amino}-2,5-dioxopyrrolidin-1-yl)benzoate |
| F2545-0216 | 4-chloro-N-(1,1-dioxo-1lambda6-thiolan-3-yl)-N-ethyl-3-nitrobenzamide |
| F2545-0439 | 4-chloro-N-methyl-N-(3-methyl-1,1-dioxo-1lambda6-thiolan-3-yl)-3-nitrobenzamide |
| F0196-1334 | 3-(2H-1,3-benzodioxol-5-yl)-6-ethyl-4-oxo-4H-chromen-7-yl acetate |
| F1298-0076 | ethyl 3-carbamoyl-2-[2-(2,5-dioxopyrrolidin-1-yl)acetamido]-4H,5H,6H,7H-thieno[2,3-c]pyridine-6-carboxylate |
| F0777-2196 | 2-[(E)-[(3,5-dichloro-2-hydroxyphenyl)methylidene]amino]cyclopent-1-ene-1-carbonitrile |
| F3139-0638 | 6-fluoro-2-(4-fluorophenyl)-4H-chromen-4-one |
| F0834-1059 | 2-(2,5-dioxopyrrolidin-1-yl)-N-[(2E)-6-fluoro-3-methyl-2,3-dihydro-1,3-benzothiazol-2-ylidene]acetamide |
| F1567-0169 | 2,4-dichloro-N-(4-oxo-2-sulfanylidene-1,3-thiazolidin-3-yl)benzamide |
| F1814-1807 | 2-(2,5-dioxopyrrolidin-1-yl)-N-[(2Z)-4-fluoro-3-methyl-2,3-dihydro-1,3-benzothiazol-2-ylidene]acetamide |
| F2384-0515 | 4-chloro-N-[1-(3-methylbutyl)-2-oxo-1,2,3,4-tetrahydroquinolin-6-yl]benzamide |
| F3385-3769 | 5-methoxy-4-oxo-2-phenyl-4H-chromen-7-yl N,N-dimethylcarbamate |
| F0834-1057 | 2-(2,5-dioxopyrrolidin-1-yl)-N-[(2E)-4-methoxy-3-methyl-2,3-dihydro-1,3-benzothiazol-2-ylidene]acetamide |
| F1691-2965 | 1-acetyl-3-[(2Z,5Z)-3-ethyl-2-(ethylimino)-4-oxo-1,3-thiazolidin-5-ylidene]-2,3-dihydro-1H-indol-2-one |
| F3095-1899 | 2-{[1,1'-biphenyl]-4-yloxy}-1-(4-methylpiperidin-1-yl)ethan-1-one |
| F3139-0414 | 7-(acetyloxy)-3-(4-methoxyphenyl)-4-oxo-4H-chromen-5-yl acetate |
| F3139-1025 | 5-(acetyloxy)-3-(2-methoxyphenyl)-4-oxo-4H-chromen-7-yl acetate |
| F1799-0504 | 3-[(adamantan-1-yl)amino]-1-benzylpyrrolidine-2,5-dione |
| F1814-1805 | N-[(2Z)-4,6-difluoro-3-methyl-2,3-dihydro-1,3-benzothiazol-2-ylidene]-2-(2,5-dioxopyrrolidin-1-yl)acetamide |
| F1175-0231 | 1-(4-chloro-3-nitrobenzoyl)-4-methylpiperidine |
| F0440-0364 | N-[(2E)-3,6-dimethyl-2,3-dihydro-1,3-benzothiazol-2-ylidene]-2-(2,5-dioxopyrrolidin-1-yl)acetamide |
| F5460-0486 | 3-(2,5-dioxopyrrolidin-1-yl)-N-[1-(6-fluoro-1,3-benzothiazol-2-yl)-3-methyl-1H-pyrazol-5-yl]benzamide |
| F3023-0930 | 1-[2-(3,4-dimethoxyphenyl)ethyl]-3-{[4-(dimethylamino)phenyl]amino}pyrrolidine-2,5-dione |
| F3023-0969 | ethyl 4-(3-{[4-(morpholin-4-yl)phenyl]amino}-2,5-dioxopyrrolidin-1-yl)benzoate |
| F3350-0591 | 4-(2,5-dioxopyrrolidin-1-yl)-N-(4-fluorophenyl)benzene-1-sulfonamide |
| F3385-0042 | ethyl 3-(4-methoxyphenyl)-4-oxo-4H-chromen-7-yl carbonate |
| F2049-0479 | N-(1-benzoyl-1,2,3,4-tetrahydroquinolin-7-yl)benzamide |
| F3385-4229 | ethyl 2-{[3-(4-methoxyphenyl)-4-oxo-4H-chromen-7-yl]oxy}acetate |
| F0847-0015 | phenyl N-(2-hydroxy-5-nitrophenyl)carbamate |
| F3323-0626 | 4-[2-(4-methoxy-3-nitrophenyl)-2-oxoethyl]-2,3,4,5-tetrahydro-1,4-benzoxazepine-3,5-dione |
| F3367-0019 | 4-[(2,5-dioxopyrrolidin-1-yl)methyl]-N-(3-methylphenyl)benzene-1-sulfonamide |
| F3023-1010 | methyl 4-(2,5-dioxo-3-{[4-(piperidin-1-yl)phenyl]amino}pyrrolidin-1-yl)benzoate |
| F3385-2702 | 3-(3,4-dimethoxyphenyl)-4-oxo-4H-chromen-7-yl N,N-dimethylcarbamate |
| F1318-0203 | 5-[(dimethylcarbamoyl)oxy]naphthalen-1-yl N,N-dimethylcarbamate |
| F3385-2709 | methyl 2-{[3-(3,4-dimethoxyphenyl)-4-oxo-4H-chromen-7-yl]oxy}acetate |
| F0196-0692 | methyl 2-{[3-(2,3-dihydro-1,4-benzodioxin-6-yl)-4-oxo-4H-chromen-7-yl]oxy}propanoate |
| F2738-2743 | N-(5,6-dimethyl-1,3-benzothiazol-2-yl)-4-(2,5-dioxopyrrolidin-1-yl)-N-[(pyridin-2-yl)methyl]benzamide |
| F3023-0949 | ethyl 4-(3-{[4-(dimethylamino)phenyl]amino}-2,5-dioxopyrrolidin-1-yl)benzoate |
| F1318-0190 | 4-(4-chlorobenzoyl)phenyl N,N-dimethylcarbamate |
| F2738-1654 | N-(4,6-difluoro-1,3-benzothiazol-2-yl)-4-(2,5-dioxopyrrolidin-1-yl)-N-[(pyridin-2-yl)methyl]benzamide |
| F2040-0025 | N-(1-acetyl-1,2,3,4-tetrahydroquinolin-6-yl)-2-chloro-4-nitrobenzamide |
| F0827-0102 | 2-(4-oxo-2-sulfanylidene-1,3-thiazolidin-3-yl)-2,3-dihydro-1H-isoindole-1,3-dione |
| F0745-0119 | 3,4,5-trimethoxy-N-(4-oxo-2-sulfanylidene-1,3-thiazolidin-3-yl)benzamide |
| F2590-0745 | ethyl 4-cyano-5-[4-(2,5-dioxopyrrolidin-1-yl)benzamido]-3-methylthiophene-2-carboxylate |
| F2590-0758 | ethyl 4-cyano-5-[3-(2,5-dioxopyrrolidin-1-yl)benzamido]-3-methylthiophene-2-carboxylate |
| F0700-0133 | 2-(2,5-dioxopyrrolidin-1-yl)-N-(4-methoxy-6-nitro-1,3-benzothiazol-2-yl)acetamide |
| F3385-2698 | 3-(3,4-dimethoxyphenyl)-4-oxo-4H-chromen-7-yl 2,2-dimethylpropanoate |
| F6442-0883 | N-(3,4-dichlorophenyl)-3-(4-methoxybenzenesulfonyl)azetidine-1-carboxamide |
| F3385-2913 | 3-(4-methoxyphenyl)-7-(2-oxo-2-phenylethoxy)-4H-chromen-4-one |
| F0703-0193 | N-(3-chloro-4-hydroxy-2,5-dimethylphenyl)benzenesulfonamide |
| F2721-0443 | 3-[(4-chloro-3-nitrophenyl)carbamoyl]-2-(ethylamino)propanoic acid |
| F1243-0042 | 7-[(dimethylcarbamoyl)oxy]naphthalen-1-yl N,N-dimethylcarbamate |
| F1243-0047 | 7-[(dimethylcarbamoyl)oxy]naphthalen-2-yl N,N-dimethylcarbamate |
| F0440-0361 | 2-(2,5-dioxopyrrolidin-1-yl)-N-[(2E)-3-methyl-6-nitro-2,3-dihydro-1,3-benzothiazol-2-ylidene]acetamide |
| F1814-1146 | 2-(2,5-dioxopyrrolidin-1-yl)-N-[(2Z)-4-methoxy-3,7-dimethyl-2,3-dihydro-1,3-benzothiazol-2-ylidene]acetamide |
| F6469-1367 | 2-(2,5-dioxopyrrolidin-1-yl)-N-{2-[2-(2-fluorophenyl)-1,3-thiazol-4-yl]phenyl}acetamide |
| F2273-0249 | 3-(2,5-dioxopyrrolidin-1-yl)-N-[5-(7-ethoxy-1-benzofuran-2-yl)-1,3,4-oxadiazol-2-yl]benzamide |
| F2261-0410 | 2-chloro-N-(3,3-dimethyl-4-oxo-5-propyl-2,3,4,5-tetrahydro-1,5-benzoxazepin-8-yl)-4-nitrobenzamide |
| F3007-0202 | 2-{[4-(4-chlorophenyl)-3-oxo-8-thia-4,6-diazatricyclo[7.4.0.0^{2,7}]trideca-1(9),2(7),5-trien-5-yl]sulfanyl}acetamide |
| F3385-3983 | 3-(3,4-dimethoxyphenyl)-5-hydroxy-4-oxo-4H-chromen-7-yl N,N-dimethylcarbamate |
| F2635-0515 | N-(1,3-benzothiazol-2-yl)-4-(2,5-dioxopyrrolidin-1-yl)-N-[(pyridin-2-yl)methyl]benzamide |
| F2545-0218 | 4-chloro-N-(1,1-dioxo-1lambda6-thiolan-3-yl)-N-(2-methylpropyl)-3-nitrobenzamide |
| F1814-1144 | 2-(2,5-dioxopyrrolidin-1-yl)-N-[(2Z)-3,5,7-trimethyl-2,3-dihydro-1,3-benzothiazol-2-ylidene]acetamide |
| F1814-1145 | 2-(2,5-dioxopyrrolidin-1-yl)-N-[(2Z)-3,4,7-trimethyl-2,3-dihydro-1,3-benzothiazol-2-ylidene]acetamide |
| F2738-0188 | 3-(2,5-dioxopyrrolidin-1-yl)-N-(4-ethyl-1,3-benzothiazol-2-yl)-N-[(pyridin-2-yl)methyl]benzamide |
| F1816-1183 | 2-(2,5-dioxopyrrolidin-1-yl)-N-[(2Z)-4-methoxy-3-(prop-2-yn-1-yl)-2,3-dihydro-1,3-benzothiazol-2-ylidene]acetamide |
| F3385-4356 | 5-ethoxy-4-oxo-2-phenyl-4H-chromen-7-yl N,N-dimethylcarbamate |
| F2610-0316 | 2-(2,5-dioxopyrrolidin-1-yl)-N-[(2Z)-3-propyl-2,3-dihydro-1,3-benzothiazol-2-ylidene]acetamide |
| F3139-1697 | 3-(3,4-dimethoxyphenyl)-4-oxo-4H-chromen-7-yl ethyl carbonate |
| F3385-2699 | methyl 2-{[3-(3,4-dimethoxyphenyl)-4-oxo-4H-chromen-7-yl]oxy}propanoate |
| F0918-6187 | 4-(4-chloro-5-methyl-3-nitro-1H-pyrazol-1-yl)-N-(5-methyl-1,2-oxazol-3-yl)butanamide |
| F3260-0633 | 3,7-dimethyl-1-[(4-methylphenyl)methyl]-8-sulfanyl-2,3,6,7-tetrahydro-1H-purine-2,6-dione |
| F0208-0057 | 4-chloro-2-nitrophenyl 2-phenoxyacetate |
| F0346-1555 | N-(2-chloro-4-nitrophenyl)-2-(3-nitro-1H-1,2,4-triazol-1-yl)acetamide |
| F0774-0090 | 1-(2,4-dichlorobenzoyl)-2-methyl-1,2,3,4-tetrahydroquinoline |
| F1366-3540 | N-[(2Z)-4,6-difluoro-3-(2-methoxyethyl)-2,3-dihydro-1,3-benzothiazol-2-ylidene]-2-(2,5-dioxopyrrolidin-1-yl)acetamide |
| F2515-2096 | N-(4,5-dimethyl-1,3-benzothiazol-2-yl)-3-(2,5-dioxopyrrolidin-1-yl)-N-[(pyridin-3-yl)methyl]benzamide |
| F1008-0057 | 4-(2-methylphenyl)-5-(methylsulfanyl)-8-thia-4,6-diazatricyclo[7.4.0.0^{2,7}]trideca-1(9),2(7),5-trien-3-one |
| F3386-1372 | N-(5-chloro-2-hydroxyphenyl)-2-methoxy-5-methylbenzene-1-sulfonamide |
| F1814-1808 | 2-(2,5-dioxopyrrolidin-1-yl)-N-[(2Z)-3-ethyl-4-fluoro-2,3-dihydro-1,3-benzothiazol-2-ylidene]acetamide |
| F2610-3868 | 2-(2,5-dioxopyrrolidin-1-yl)-N-[(2Z)-6-fluoro-3-propyl-2,3-dihydro-1,3-benzothiazol-2-ylidene]acetamide |
| F3188-0013 | ethyl 4-{1-[2-(3,4-dimethoxyphenyl)ethyl]-2,5-dioxopyrrolidin-3-yl}piperazine-1-carboxylate |
| F2615-1251 | 2-(2,5-dioxopyrrolidin-1-yl)-N-[(2Z)-4-fluoro-3-propyl-2,3-dihydro-1,3-benzothiazol-2-ylidene]acetamide |
| F5526-0475 | 2-chloro-N-[2-(4-ethoxyphenoxy)ethyl]-4-nitrobenzamide |
| F3260-0165 | 1-benzyl-3,7-dimethyl-8-sulfanyl-2,3,6,7-tetrahydro-1H-purine-2,6-dione |
| F0808-0581 | N-(3-chloro-4-hydroxyphenyl)-2,4-dimethylbenzene-1-sulfonamide |
| F3367-0021 | N-(3-chlorophenyl)-4-[(2,5-dioxopyrrolidin-1-yl)methyl]benzene-1-sulfonamide |
| F0373-0620 | 1-benzyl-8-bromo-3,7-dimethyl-2,3,6,7-tetrahydro-1H-purine-2,6-dione |
| F1359-0108 | 2-(2,5-dioxopyrrolidin-1-yl)-N-[(2Z)-3-(2-methoxyethyl)-6-methyl-2,3-dihydro-1,3-benzothiazol-2-ylidene]acetamide |
| F1814-1148 | N-[(2Z)-7-chloro-3,4-dimethyl-2,3-dihydro-1,3-benzothiazol-2-ylidene]-2-(2,5-dioxopyrrolidin-1-yl)acetamide |
| F1365-2616 | 2-(2,5-dioxopyrrolidin-1-yl)-N-[(2Z)-6-fluoro-3-(prop-2-yn-1-yl)-2,3-dihydro-1,3-benzothiazol-2-ylidene]acetamide |
| F1366-4728 | 2-(2,5-dioxopyrrolidin-1-yl)-N-[(2Z)-4-fluoro-3-(prop-2-yn-1-yl)-2,3-dihydro-1,3-benzothiazol-2-ylidene]acetamide |
| F0808-0637 | N-(3-chloro-4-hydroxyphenyl)-2,5-dimethylbenzene-1-sulfonamide |
| F2049-0369 | 4-chloro-N-[1-(2-methylpropanoyl)-1,2,3,4-tetrahydroquinolin-7-yl]benzamide |
| F0149-0042 | 4-nitrophenyl 2-(3-methoxyphenoxy)acetate |
| F3161-0035 | 1-{5-hydroxy-2,15-dimethyltetracyclo[8.7.0.0^{2,7}.0^{11,15}]heptadec-7-en-14-yl}ethan-1-one |
| F3386-1371 | N-(5-chloro-2-hydroxyphenyl)-2,4,5-trimethylbenzene-1-sulfonamide |
| F1814-1806 | 2-(2,5-dioxopyrrolidin-1-yl)-N-[(2Z)-3-ethyl-4,6-difluoro-2,3-dihydro-1,3-benzothiazol-2-ylidene]acetamide |
| F3115-0094 | ethyl 4-[1-(2-butoxyphenyl)-2,5-dioxopyrrolidin-3-yl]piperazine-1-carboxylate |
| F3385-2739 | ethyl 2-{[3-(3,4-dimethoxyphenyl)-4-oxo-4H-chromen-7-yl]oxy}acetate |
| F1814-1147 | N-[(2Z)-4,7-dimethoxy-3-methyl-2,3-dihydro-1,3-benzothiazol-2-ylidene]-2-(2,5-dioxopyrrolidin-1-yl)acetamide |
| F1816-1185 | 2-(2,5-dioxopyrrolidin-1-yl)-N-[(2Z)-4-methyl-3-(prop-2-yn-1-yl)-2,3-dihydro-1,3-benzothiazol-2-ylidene]acetamide |
| F3064-0198 | 8-bromo-3,7-dimethyl-1-[(4-methylphenyl)methyl]-2,3,6,7-tetrahydro-1H-purine-2,6-dione |
| F2515-2223 | N-(4,6-dimethyl-1,3-benzothiazol-2-yl)-3-(2,5-dioxopyrrolidin-1-yl)-N-[(pyridin-3-yl)methyl]benzamide |
| F2524-0976 | 2-(2,5-dioxopyrrolidin-1-yl)-N-[(5Z)-6-(prop-2-yn-1-yl)-10,13-dioxa-4-thia-6-azatricyclo[7.4.0.0^{3,7}]trideca-1,3(7),8-trien-5-ylidene]acetamide |
| F3385-4094 | 3-(3,4-dimethoxyphenyl)-5-methoxy-4-oxo-4H-chromen-7-yl N,N-dimethylcarbamate |
| F3386-1370 | N-(5-chloro-2-hydroxyphenyl)-2,4,6-trimethylbenzene-1-sulfonamide |
| F6401-0072 | 1-[(naphthalen-1-yl)methyl]-4-(thiophen-2-yl)piperidine-2,6-dione |
| F2515-2731 | N-(4,6-difluoro-1,3-benzothiazol-2-yl)-3-(2,5-dioxopyrrolidin-1-yl)-N-[(pyridin-3-yl)methyl]benzamide |
| F2040-0081 | 2-chloro-N-(1-cyclopropanecarbonyl-1,2,3,4-tetrahydroquinolin-6-yl)-4-nitrobenzamide |
| F1029-0049 | 6-(methylsulfanyl)-2-oxo-3,8-diphenyl-1H,2H-5lambda5-[1,3]thiazolo[3,4-b][1,2,4]triazin-5-ylium benzenesulfonate |
| F2073-0018 | 4-chloro-N-{2-methyl-4-oxo-4H-pyrido[1,2-a]pyrimidin-3-yl}-3-nitrobenzamide |
| F2515-0826 | 3-(2,5-dioxopyrrolidin-1-yl)-N-(6-ethyl-1,3-benzothiazol-2-yl)-N-[(pyridin-3-yl)methyl]benzamide |
| F1366-0504 | methyl 2-[(2Z)-2-{[2-(2,5-dioxopyrrolidin-1-yl)acetyl]imino}-2,3-dihydro-1,3-benzothiazol-3-yl]acetate |
| F0293-0022 | 1-chloro-2-nitro-4-trifluoromethanesulfonylbenzene |
| F0433-0012 | 4-chloro-N-(3-chloro-4-hydroxyphenyl)benzene-1-sulfonamide |
| F2545-0217 | N-butyl-4-chloro-N-(1,1-dioxo-1lambda6-thiolan-3-yl)-3-nitrobenzamide |
| F3325-0110 | 1-[2-(3,4-dimethoxyphenyl)ethyl]-3-[4-(4-fluorophenyl)piperazin-1-yl]pyrrolidine-2,5-dione |
| F1814-1154 | 2-(2,5-dioxopyrrolidin-1-yl)-N-[(2Z)-3-ethyl-4-methoxy-7-methyl-2,3-dihydro-1,3-benzothiazol-2-ylidene]acetamide |
| F3386-1385 | N-(5-chloro-2-hydroxyphenyl)-5-ethyl-2-methoxybenzene-1-sulfonamide |
| F5581-1075 | N-(4,6-dimethyl-1,3-benzothiazol-2-yl)-3-(2,5-dioxopyrrolidin-1-yl)-N-[2-(1H-pyrazol-1-yl)ethyl]benzamide |
| F0440-0325 | N-[(2E)-6-bromo-3-methyl-2,3-dihydro-1,3-benzothiazol-2-ylidene]-2-(2,5-dioxopyrrolidin-1-yl)acetamide |
| F2779-0189 | ethyl 2-[4-(2,5-dioxopyrrolidin-1-yl)benzamido]-1-benzothiophene-3-carboxylate |
| F2073-0321 | 4-chloro-N-{2,8-dimethyl-4-oxo-4H-pyrido[1,2-a]pyrimidin-3-yl}-3-nitrobenzamide |
| F1816-1181 | N-[(2Z)-5,7-dimethyl-3-(prop-2-yn-1-yl)-2,3-dihydro-1,3-benzothiazol-2-ylidene]-2-(2,5-dioxopyrrolidin-1-yl)acetamide |
| F1298-0284 | 3,6-diethyl 2-[2-(2,5-dioxopyrrolidin-1-yl)acetamido]-4H,5H,6H,7H-thieno[2,3-c]pyridine-3,6-dicarboxylate |
| F2073-0422 | 4-chloro-N-{2,9-dimethyl-4-oxo-4H-pyrido[1,2-a]pyrimidin-3-yl}-3-nitrobenzamide |
| F1086-0007 | 5,6-dimethyl-3-phenyl-2-(prop-2-yn-1-ylsulfanyl)-3H,4H-thieno[2,3-d]pyrimidin-4-one |
| F5045-0060 | 3-(2,5-dioxopyrrolidin-1-yl)-N-[4-(6-methoxy-1-benzofuran-2-yl)-1,3-thiazol-2-yl]benzamide |
| F0541-1187 | N-{2-tert-butyl-2H,4H,6H-thieno[3,4-c]pyrazol-3-yl}-4-chloro-3-nitrobenzamide |
| F1359-1560 | 2-(2,5-dioxopyrrolidin-1-yl)-N-[(2Z)-6-methoxy-3-(2-methoxyethyl)-2,3-dihydro-1,3-benzothiazol-2-ylidene]acetamide |
| F1086-0008 | 11-phenyl-10-(prop-2-yn-1-ylsulfanyl)-7-thia-9,11-diazatricyclo[6.4.0.0^{2,6}]dodeca-1(8),2(6),9-trien-12-one |
| F5581-0655 | 3-(2,5-dioxopyrrolidin-1-yl)-N-(6-fluoro-1,3-benzothiazol-2-yl)-N-[2-(1H-pyrazol-1-yl)ethyl]benzamide |
| F2527-0077 | [6-methyl-3-(methylsulfanyl)-5-oxo-4,5-dihydro-1,2,4-triazin-4-yl]methyl 4-chloro-3-nitrobenzoate |
| F2290-0045 | 4-(2,5-dioxopyrrolidin-1-yl)-N-[4-(5-methoxy-1-benzofuran-2-yl)-1,3-thiazol-2-yl]benzamide |
| F2252-0059 | 3-(2,5-dioxopyrrolidin-1-yl)-N-[4-(7-methoxy-1-benzofuran-2-yl)-1,3-thiazol-2-yl]benzamide |
| F2738-1170 | 4-(2,5-dioxopyrrolidin-1-yl)-N-(6-ethoxy-1,3-benzothiazol-2-yl)-N-[(pyridin-2-yl)methyl]benzamide |
| F2018-3400 | N-(6-chloro-1,3-benzothiazol-2-yl)-N-[2-(diethylamino)ethyl]-2-(2,5-dioxopyrrolidin-1-yl)acetamide hydrochloride |
| F2770-0240 | 2-chloro-N-(1-methanesulfonyl-1,2,3,4-tetrahydroquinolin-6-yl)-4-nitrobenzamide |
| F2018-4289 | N-(4-chloro-1,3-benzothiazol-2-yl)-N-[2-(diethylamino)ethyl]-2-(2,5-dioxopyrrolidin-1-yl)acetamide hydrochloride |
| F0808-0002 | N-(3,5-dichloro-4-hydroxyphenyl)-4-methoxybenzene-1-sulfonamide |
| F1359-2616 | 2-(2,5-dioxopyrrolidin-1-yl)-N-[(2Z)-3-(2-ethoxyethyl)-6-fluoro-2,3-dihydro-1,3-benzothiazol-2-ylidene]acetamide |
| F1366-0108 | methyl 2-[(2Z)-2-{[2-(2,5-dioxopyrrolidin-1-yl)acetyl]imino}-6-methyl-2,3-dihydro-1,3-benzothiazol-3-yl]acetate |
| F1345-0286 | 1-(9H-carbazol-9-yl)-3-(2,6-dimethylmorpholin-4-yl)propan-2-ol; benzoic acid |
| F2019-1241 | N-[3-(dimethylamino)propyl]-2-(2,5-dioxopyrrolidin-1-yl)-N-(4-ethoxy-1,3-benzothiazol-2-yl)acetamide hydrochloride |
| F1365-2352 | N-[(2Z)-6-chloro-3-(prop-2-yn-1-yl)-2,3-dihydro-1,3-benzothiazol-2-ylidene]-2-(2,5-dioxopyrrolidin-1-yl)acetamide |
| F0808-1789 | 4-chloro-3-nitro-N-(4-oxocyclohexa-2,5-dien-1-ylidene)benzene-1-sulfonamide |
| F1365-1164 | 2-(2,5-dioxopyrrolidin-1-yl)-N-[(2Z)-6-methanesulfonyl-3-(prop-2-en-1-yl)-2,3-dihydro-1,3-benzothiazol-2-ylidene]acetamide |
| F3385-2696 | propan-2-yl 2-{[3-(3,4-dimethoxyphenyl)-4-oxo-4H-chromen-7-yl]oxy}acetate |
| F1297-0181 | 2-(2,5-dioxopyrrolidin-1-yl)-N-[(2E)-4-ethoxy-3-ethyl-2,3-dihydro-1,3-benzothiazol-2-ylidene]acetamide |
| F0466-0008 | 3-(2,5-dioxopyrrolidin-1-yl)-N-{naphtho[2,1-d][1,3]thiazol-2-yl}benzamide |
| F0846-0687 | N-[2-(1,3-benzoxazol-2-yl)phenyl]-2-(2,5-dioxopyrrolidin-1-yl)acetamide |
| F0327-0308 | 4-(2,5-dioxopyrrolidin-1-yl)-N-{naphtho[1,2-d][1,3]thiazol-2-yl}benzamide |
| F0913-0305 | 3-(1,3-benzoxazol-2-yl)aniline |
| F1443-0926 | 2-{5-hydroxy-2,15-dimethyltetracyclo[8.7.0.0^{2,7}.0^{11,15}]heptadec-7-en-14-yl}-2-oxoethyl acetate |
| F1359-1824 | 2-(2,5-dioxopyrrolidin-1-yl)-N-[(2Z)-3-(2-ethoxyethyl)-6-methyl-2,3-dihydro-1,3-benzothiazol-2-ylidene]acetamide |
| F3386-1378 | N-(5-chloro-2-hydroxyphenyl)-2-methoxy-5-(propan-2-yl)benzene-1-sulfonamide |
| F2966-0198 | 3-(2,5-dioxopyrrolidin-1-yl)-N-[4-(methylsulfanyl)-1,3-benzothiazol-2-yl]-N-[(pyridin-3-yl)methyl]benzamide |
| F1365-2748 | 2-(2,5-dioxopyrrolidin-1-yl)-N-[(2Z)-6-nitro-3-(prop-2-yn-1-yl)-2,3-dihydro-1,3-benzothiazol-2-ylidene]acetamide |
| F2630-0142 | N-(4-chloro-3-nitrophenyl)-4-methyl-1,2,3-thiadiazole-5-carboxamide |
| F3386-1383 | 4-chloro-N-(5-chloro-2-hydroxyphenyl)-3-methoxybenzene-1-sulfonamide |
| F1814-1155 | 2-(2,5-dioxopyrrolidin-1-yl)-N-[(2Z)-3-ethyl-4,7-dimethoxy-2,3-dihydro-1,3-benzothiazol-2-ylidene]acetamide |
| F1815-1183 | 2-(2,5-dioxopyrrolidin-1-yl)-N-[(2Z)-4-methoxy-3-(prop-2-en-1-yl)-2,3-dihydro-1,3-benzothiazol-2-ylidene]acetamide |
| F5581-0445 | 3-(2,5-dioxopyrrolidin-1-yl)-N-(6-methoxy-1,3-benzothiazol-2-yl)-N-[2-(1H-pyrazol-1-yl)ethyl]benzamide |
| F3350-0588 | N-(2,4-dimethoxyphenyl)-4-(2,5-dioxopyrrolidin-1-yl)benzene-1-sulfonamide |
| F2167-9481 | 1,6-dimethylphenanthrene-9-carboxylic acid |
| F3385-0999 | 3-(2,3-dihydro-1,4-benzodioxin-6-yl)-7-(2-oxo-2-phenylethoxy)-4H-chromen-4-one |
| F1086-0006 | 4-phenyl-5-(prop-2-yn-1-ylsulfanyl)-8-thia-4,6-diazatricyclo[7.4.0.0^{2,7}]trideca-1(9),2(7),5-trien-3-one |
| F1816-1187 | N-[(2Z)-4-chloro-3-(prop-2-yn-1-yl)-2,3-dihydro-1,3-benzothiazol-2-ylidene]-2-(2,5-dioxopyrrolidin-1-yl)acetamide |
| F2843-0109 | N-(2,6-difluorobenzoyl)-N-phenylpiperidine-1-carboxamide |
| F1381-1222 | ethyl (2E)-2-{[2-(2,5-dioxopyrrolidin-1-yl)acetyl]imino}-3-methyl-2,3-dihydro-1,3-benzothiazole-6-carboxylate |
| F0715-0371 | ethyl (2Z)-2-{[4-(2,5-dioxopyrrolidin-1-yl)benzoyl]imino}-3,4-dimethyl-2,3-dihydro-1,3-thiazole-5-carboxylate |
| F0037-5306 | 4-(1,3-benzoxazol-2-yl)aniline |
| F0440-0369 | N-(1,2-dihydroacenaphthylen-5-yl)-2-(2,5-dioxopyrrolidin-1-yl)acetamide |
| F3244-0167 | 2-[5-(2-chloro-4-nitrophenyl)furan-2-yl]-5-(furan-2-yl)-1,3,4-oxadiazole |
| F0541-0058 | N-{2-tert-butyl-5,5-dioxo-2H,4H,6H-5lambda6-thieno[3,4-c]pyrazol-3-yl}-4-chloro-3-nitrobenzamide |
| F0326-1085 | 4-chloro-N-(5-methyl-1,3,4-thiadiazol-2-yl)-3-nitrobenzamide |
| F3386-1381 | 5-chloro-N-(5-chloro-2-hydroxyphenyl)-2-methoxy-4-methylbenzene-1-sulfonamide |
| F2515-0318 | 3-(2,5-dioxopyrrolidin-1-yl)-N-(6-ethoxy-1,3-benzothiazol-2-yl)-N-[(pyridin-3-yl)methyl]benzamide |
| F3041-0409 | N,N-dimethyl-2-(3-methylbutanoyl)-3-oxo-2-azatricyclo[6.3.1.0^{4,12}]dodeca-1(11),4(12),5,7,9-pentaene-9-sulfonamide |
| F2791-0178 | 3-[4-(4-chloro-3-nitrobenzoyl)piperazin-1-yl]-6-(4-methylpiperidin-1-yl)pyridazine |
| F1816-1184 | 2-(2,5-dioxopyrrolidin-1-yl)-N-[(2Z)-4-ethoxy-3-(prop-2-yn-1-yl)-2,3-dihydro-1,3-benzothiazol-2-ylidene]acetamide |
| F6169-0499 | N-(4-chloro-3-nitrophenyl)-2-[6-oxo-3-(1H-pyrazol-1-yl)-1,6-dihydropyridazin-1-yl]propanamide |
| F0348-1184 | 2-(4-chloro-5-methyl-3-nitro-1H-pyrazol-1-yl)-N-[(4-chlorophenyl)methyl]acetamide |
| F0327-0318 | 4-(2,5-dioxopyrrolidin-1-yl)-N-[2-(methylsulfanyl)-1,3-benzothiazol-6-yl]benzamide |
| F0440-0379 | 2-(2,5-dioxopyrrolidin-1-yl)-N-[5-(4-nitrobenzenesulfonyl)-1,3-thiazol-2-yl]acetamide |
| F2701-0046 | 3,6-dimethyl 2-[4-(2,5-dioxopyrrolidin-1-yl)benzamido]-4H,5H,6H,7H-thieno[2,3-c]pyridine-3,6-dicarboxylate |
| F2701-0060 | 3,6-dimethyl 2-[3-(2,5-dioxopyrrolidin-1-yl)benzamido]-4H,5H,6H,7H-thieno[2,3-c]pyridine-3,6-dicarboxylate |
| F0700-0137 | 2-(2,5-dioxopyrrolidin-1-yl)-N-{11-methyl-3,12-dithia-5,10-diazatricyclo[7.3.0.0^{2,6}]dodeca-1(9),2(6),4,7,10-pentaen-4-yl}acetamide |
| F0447-0399 | 2-(2,5-dioxopyrrolidin-1-yl)-N-{4'-[2-(2,5-dioxopyrrolidin-1-yl)acetamido]-[1,1'-biphenyl]-4-yl}acetamide |
| F3386-1387 | 4-chloro-N-(5-chloro-2-hydroxyphenyl)-2,5-dimethoxybenzene-1-sulfonamide |
| F5834-4449 | 2-(2,6-dioxopiperidin-1-yl)-N-{6-methyl-10-oxo-2-oxa-9-azatricyclo[9.4.0.0^{3,8}]pentadeca-1(11),3(8),4,6,12,14-hexaen-13-yl}ethane-1-sulfonamide |
| F0345-4219 | 2-(4-chloro-5-methyl-3-nitro-1H-pyrazol-1-yl)-N-(3,4-dimethoxyphenyl)acetamide |
| F1366-1560 | methyl 2-[(2Z)-2-{[2-(2,5-dioxopyrrolidin-1-yl)acetyl]imino}-6-methoxy-2,3-dihydro-1,3-benzothiazol-3-yl]acetate |
| F2252-0237 | 3-(2,5-dioxopyrrolidin-1-yl)-N-[4-(7-ethoxy-1-benzofuran-2-yl)-1,3-thiazol-2-yl]benzamide |
| F0371-0083 | 6-[(2,4-dinitrophenyl)sulfanyl]-N2,N4-diethyl-1,3,5-triazine-2,4-diamine |
| F0326-1109 | 4-chloro-3-nitro-N-[5-(propan-2-yl)-1,3,4-thiadiazol-2-yl]benzamide |
| F0440-0360 | N-[4-(1,3-benzothiazol-2-yl)-3-hydroxyphenyl]-2-(2,5-dioxopyrrolidin-1-yl)acetamide |
| F2018-4416 | N-[2-(diethylamino)ethyl]-N-{4,6-dioxa-10-thia-12-azatricyclo[7.3.0.0^{3,7}]dodeca-1(9),2,7,11-tetraen-11-yl}-2-(2,5-dioxopyrrolidin-1-yl)acetamide hydrochloride |
| F6617-5677 | 2-chloro-6-(3-methyl-1,2,4-oxadiazol-5-yl)pyridine |
| F0700-0136 | N-[3-(1,3-benzothiazol-2-yl)-4-hydroxyphenyl]-2-(2,5-dioxopyrrolidin-1-yl)acetamide |
| F2614-0760 | N-[(2Z)-4,7-dimethoxy-3-propyl-2,3-dihydro-1,3-benzothiazol-2-ylidene]-2-(2,5-dioxopyrrolidin-1-yl)acetamide |
| F1359-1692 | 2-(2,5-dioxopyrrolidin-1-yl)-N-[(2Z)-6-ethoxy-3-(2-methoxyethyl)-2,3-dihydro-1,3-benzothiazol-2-ylidene]acetamide |
| F1366-0636 | methyl 2-[(2Z)-6-chloro-2-{[2-(2,5-dioxopyrrolidin-1-yl)acetyl]imino}-2,3-dihydro-1,3-benzothiazol-3-yl]acetate |
| F5882-3004 | N-(3,4-dichlorophenyl)-4-[(thiophen-3-yl)methyl]piperazine-1-carboxamide |
| F2018-3527 | N-(6-bromo-1,3-benzothiazol-2-yl)-N-[2-(diethylamino)ethyl]-2-(2,5-dioxopyrrolidin-1-yl)acetamide hydrochloride |
| F3386-1377 | 5-bromo-N-(5-chloro-2-hydroxyphenyl)-2-methoxybenzene-1-sulfonamide |
| F0440-0348 | 2-(2,5-dioxopyrrolidin-1-yl)-N-[4-(naphthalen-2-yl)-1,3-thiazol-2-yl]acetamide |
| F0758-0104 | 4-oxo-3-phenyl-4H-chromen-7-yl acetate |
| F1198-0001 | 2-({5,6-dimethyl-4-oxo-3-phenyl-3H,4H-thieno[2,3-d]pyrimidin-2-yl}sulfanyl)-N,N-bis(2-hydroxyethyl)acetamide |
| F5017-2080 | phenyl 4-{[2-(2,6-dioxopiperidin-1-yl)ethanesulfonamido]methyl}piperidine-1-carboxylate |
| F1558-0186 | 2-(4-methylphenyl)-1H-1,3-benzodiazole |
| F2018-4543 | N-[2-(diethylamino)ethyl]-N-{10,13-dioxa-4-thia-6-azatricyclo[7.4.0.0^{3,7}]trideca-1,3(7),5,8-tetraen-5-yl}-2-(2,5-dioxopyrrolidin-1-yl)acetamide hydrochloride |
| F0049-0055 | 4-chloro-3-nitro-N-(2,2,6,6-tetramethylpiperidin-4-yl)benzene-1-sulfonamide |
| F1815-1184 | 2-(2,5-dioxopyrrolidin-1-yl)-N-[(2Z)-4-ethoxy-3-(prop-2-en-1-yl)-2,3-dihydro-1,3-benzothiazol-2-ylidene]acetamide |
| F1365-1692 | 2-(2,5-dioxopyrrolidin-1-yl)-N-[(2Z)-6-ethoxy-3-(prop-2-en-1-yl)-2,3-dihydro-1,3-benzothiazol-2-ylidene]acetamide |
| F0817-0238 | N-[4-(1,3-benzoxazol-2-yl)phenyl]-3-(2,5-dioxopyrrolidin-1-yl)benzamide |
| F3115-0077 | N'-[1-(4-methoxyphenyl)-2,5-dioxopyrrolidin-3-yl]benzohydrazide |
| F3375-2581 | 4-[5-(3-chloro-4-nitrophenyl)-2-methylfuran-3-carbonyl]morpholine |
| F6274-0445 | 1-(6-fluoro-1,3-benzothiazol-2-yl)azetidin-3-yl 3-(2,5-dioxopyrrolidin-1-yl)benzoate |
| F0472-0083 | 4-hydroxy-3-[(1E)-[(pyrimidin-2-yl)imino]methyl]-2H-chromen-2-one |
| F1359-0768 | N-[(2Z)-6-bromo-3-(2-methoxyethyl)-2,3-dihydro-1,3-benzothiazol-2-ylidene]-2-(2,5-dioxopyrrolidin-1-yl)acetamide |
| F0349-0376 | 2-(4-bromo-5-methyl-3-nitro-1H-pyrazol-1-yl)-N-(4-chloro-2-methoxyphenyl)acetamide |
| F3271-0041 | 1-{4-[5-(2-chloro-4-nitrophenyl)furan-2-carbothioyl]piperazin-1-yl}ethan-1-one |
| F0700-0168 | (1,3-benzothiazol-2-yl)methyl 3-(2,5-dioxopyrrolidin-1-yl)benzoate |
| F3342-0153 | N-(4-fluorophenyl)-N-[2-hydroxy-3-(piperidin-1-yl)propyl]-4-methylbenzene-1-sulfonamide; benzoic acid |
| F1911-8405 | methyl 4-phenoxybenzoate |
| F0200-0014 | 2-{[1-(2-methoxyphenyl)-2,5-dioxopyrrolidin-3-yl]sulfanyl}benzoic acid |
| F1359-0372 | methyl (2Z)-2-{[2-(2,5-dioxopyrrolidin-1-yl)acetyl]imino}-3-(2-methoxyethyl)-2,3-dihydro-1,3-benzothiazole-6-carboxylate |
| F0017-0031 | 2,4,6-trinitrophenol; 2-(1H-1,3-benzodiazol-2-ylsulfanyl)-1-phenylethan-1-one |
| F0700-0154 | 3-(2,5-dioxopyrrolidin-1-yl)-N-[2-(methylsulfanyl)-1,3-benzothiazol-6-yl]benzamide |
| F1558-0154 | 2-(3-methylphenyl)-1H-1,3-benzodiazole |
| F1011-0198 | 1-[3-(3,6-dichloro-9H-carbazol-9-yl)-2-hydroxypropyl]pyrrolidine-2,5-dione |
| F3023-0825 | ethyl 4-[1-(4-iodo-2-methylphenyl)-2,5-dioxopyrrolidin-3-yl]piperazine-1-carboxylate |
| F0327-0315 | N-[3-(1,3-benzothiazol-2-yl)-4-hydroxyphenyl]-4-(2,5-dioxopyrrolidin-1-yl)benzamide |
| F1365-0372 | methyl (2Z)-2-{[2-(2,5-dioxopyrrolidin-1-yl)acetyl]imino}-3-(prop-2-en-1-yl)-2,3-dihydro-1,3-benzothiazole-6-carboxylate |
| F1799-0413 | N'-[1-(4-chlorophenyl)-2,5-dioxopyrrolidin-3-yl]-2-methylbenzohydrazide |
| F0700-0156 | N-[3-(1,3-benzothiazol-2-yl)-4-hydroxyphenyl]-3-(2,5-dioxopyrrolidin-1-yl)benzamide |
| F1791-1068 | 2-{[1-(4-hydroxyphenyl)-2,5-dioxopyrrolidin-3-yl]sulfanyl}benzoic acid |
| F0725-0495 | N-[3-(1,3-benzothiazol-2-yl)phenyl]-4-(2,5-dioxopyrrolidin-1-yl)benzamide |
| F0816-0636 | N-[4-(1,3-benzothiazol-2-yl)phenyl]-3-(2,5-dioxopyrrolidin-1-yl)benzamide |
| F0838-0508 | N-(1,3-benzothiazol-2-yl)-4-chloro-2-nitrobenzamide |
| F1065-0275 | 2-{[1-(2-methylphenyl)-2,5-dioxopyrrolidin-3-yl]sulfanyl}benzoic acid |
| F1819-1181 | ethyl 2-[(2Z)-2-{[2-(2,5-dioxopyrrolidin-1-yl)acetyl]imino}-5,7-dimethyl-2,3-dihydro-1,3-benzothiazol-3-yl]acetate |
| F0466-0059 | 3-[3-(2,5-dioxopyrrolidin-1-yl)phenyl]-2-[(2E)-1-methyl-2,3-dihydro-1H-1,3-benzodiazol-2-ylidene]-3-oxopropanenitrile |
| F1298-0356 | ethyl 3-cyano-2-[3-(2,5-dioxopyrrolidin-1-yl)benzamido]-4H,5H,6H,7H-thieno[2,3-c]pyridine-6-carboxylate |
| F0440-0389 | 2-(2,5-dioxopyrrolidin-1-yl)-N-[4-({4-[2-(2,5-dioxopyrrolidin-1-yl)acetamido]phenyl}methyl)phenyl]acetamide |
| F0761-0032 | 1-{4-[5-(4-chloro-2-nitrophenyl)furan-2-carbothioyl]piperazin-1-yl}ethan-1-one |
| F3162-0015 | 6-methyl-2-phenyl-1H-1,3-benzodiazole |
| F3275-0017 | 1-(4-chloro-3,5-dimethyl-1H-pyrazol-1-yl)-3-(2,3-dimethyl-1H-indol-1-yl)propan-2-ol hydrochloride |
| F6411-4564 | 4-[3-(3-fluorophenyl)-1H-pyrazole-5-carbonyl]-1-(2-phenylethyl)piperazine-2,6-dione |
| F0327-0348 | 4-(2,5-dioxopyrrolidin-1-yl)-N-[4-(naphthalen-2-yl)-1,3-thiazol-2-yl]benzamide |
| F0327-0347 | 4-(2,5-dioxopyrrolidin-1-yl)-N-[4-(naphthalen-1-yl)-1,3-thiazol-2-yl]benzamide |
| F0806-0078 | methyl 3-[3-(2,5-dioxopyrrolidin-1-yl)benzamido]-1-benzothiophene-2-carboxylate |
| F3374-0020 | N-(2-chloro-4-nitrophenyl)-3-(morpholine-4-sulfonyl)benzamide |
| F0806-0059 | methyl 3-[4-(2,5-dioxopyrrolidin-1-yl)benzamido]-1-benzothiophene-2-carboxylate |
| F1118-0368 | N-(4-chloro-3-nitrophenyl)-3-(5-methylfuran-2-yl)propanamide |
| F1799-0414 | N'-[1-(4-chlorophenyl)-2,5-dioxopyrrolidin-3-yl]-3-methylbenzohydrazide |
| F2724-0268 | 2-chloro-N-{3-[6-(morpholin-4-yl)pyridazin-3-yl]phenyl}-4-nitrobenzamide |
| F3271-0040 | 3-{4-[5-(2-chloro-4-nitrophenyl)furan-2-carbothioyl]piperazin-1-yl}propanenitrile |
| F2724-0678 | 2-chloro-N-{4-[6-(morpholin-4-yl)pyridazin-3-yl]phenyl}-4-nitrobenzamide |
| F0588-0148 | ethyl 3-[4-(2,5-dioxopyrrolidin-1-yl)benzamido]-1H-indole-2-carboxylate |
| F1175-0184 | N-(1,3-benzothiazol-2-yl)-2-chloro-4-nitrobenzamide |
| F2547-0087 | 4-chloro-N-(1,1-dioxo-1lambda6-thiolan-3-yl)-N-ethyl-3-nitrobenzene-1-sulfonamide |
| F1300-0044 | methyl 2-[3-(2,5-dioxopyrrolidin-1-yl)benzamido]-1,3-benzothiazole-6-carboxylate |
| F1065-0793 | 2-({1-[4-(dimethylamino)phenyl]-2,5-dioxopyrrolidin-3-yl}sulfanyl)benzoic acid |
| F3395-0158 | (2E)-3-(naphthalen-2-yl)prop-2-enoic acid |
| F1359-2484 | N-[(2Z)-6-bromo-3-(2-ethoxyethyl)-2,3-dihydro-1,3-benzothiazol-2-ylidene]-2-(2,5-dioxopyrrolidin-1-yl)acetamide |
| F0614-0106 | 4-(2,5-dioxopyrrolidin-1-yl)-N-{4-methyl-5-oxo-5H-chromeno[4,3-b]pyridin-2-yl}benzamide |
| F2167-9582 | 3,4-bis(benzyloxy)benzoic acid |
| F0359-0020 | 2,4-diethyl 5-[4-(2,5-dioxopyrrolidin-1-yl)benzamido]-3-methylthiophene-2,4-dicarboxylate |
| F0762-0395 | (2E)-N-(2-chloro-4-nitrophenyl)-2-cyano-3-(furan-2-yl)prop-2-enamide |
| F1065-0404 | 2-{[1-(2-ethylphenyl)-2,5-dioxopyrrolidin-3-yl]sulfanyl}benzoic acid |
| F2106-0084 | 4,7-dihydroxy-3-(2-methoxyphenyl)-2H-chromen-2-one |
| F1065-0002 | 2-{[1-(4-fluorophenyl)-2,5-dioxopyrrolidin-3-yl]sulfanyl}benzoic acid |
| F0440-0340 | 2-(2,5-dioxopyrrolidin-1-yl)-N-(4,5-diphenyl-1,3-thiazol-2-yl)acetamide |
| F3098-1918 | (2E)-3-(4-tert-butylphenyl)prop-2-enoic acid |
| F0762-0316 | (2Z)-N-(4-chloro-2-nitrophenyl)-2-cyano-3-(5-methylfuran-2-yl)prop-2-enamide |
| F2701-0561 | methyl 2-[3-(2,5-dioxopyrrolidin-1-yl)benzamido]-3-(methylcarbamoyl)-4H,5H,6H,7H-thieno[2,3-c]pyridine-6-carboxylate |
| F1822-0284 | N-{4,6-dioxa-10-thia-12-azatricyclo[7.3.0.0^{3,7}]dodeca-1(9),2,7,11-tetraen-11-yl}-4-(2,5-dioxopyrrolidin-1-yl)benzamide |
| F2106-0072 | 3-(3,4-dimethoxyphenyl)-4,7-dihydroxy-2H-chromen-2-one |
| F1441-0664 | 10-{[2-(azepan-1-yl)-2-oxoethyl]sulfanyl}-11-phenyl-7-thia-9,11-diazatricyclo[6.4.0.0^{2,6}]dodeca-1(8),2(6),9-trien-12-one |
| F1757-0335 | 1-(4-benzoylphenoxy)-3-(4-chloro-3,5-dimethyl-1H-pyrazol-1-yl)propan-2-ol |
| F1298-0044 | ethyl 3-carbamoyl-2-[3-(2,5-dioxopyrrolidin-1-yl)benzamido]-4H,5H,6H,7H-thieno[2,3-c]pyridine-6-carboxylate |
| F0013-0083 | 2-(4-ethoxyphenyl)-1,3-benzothiazole |
| F0799-0005 | 8-bromo-1,3-dimethyl-7-(prop-2-en-1-yl)-2,3,6,7-tetrahydro-1H-purine-2,6-dione |
| F3048-0123 | 2-{[1-(2-methoxyphenyl)-2,5-dioxopyrrolidin-3-yl]sulfanyl}-N-phenylacetamide |
| F3023-0915 | 3-[4-(4-fluorophenyl)piperazin-1-yl]-1-methylpyrrolidine-2,5-dione |
| F0346-1856 | 2-(4-bromo-5-methyl-3-nitro-1H-pyrazol-1-yl)-N-(4-chloro-2,5-dimethoxyphenyl)acetamide |
| F1300-0148 | ethyl 2-[3-(2,5-dioxopyrrolidin-1-yl)benzamido]-1,3-benzothiazole-6-carboxylate |
| F1408-0414 | 11-methyl-5-{[2-oxo-2-(pyrrolidin-1-yl)ethyl]sulfanyl}-4-phenyl-8-thia-4,6-diazatricyclo[7.4.0.0^{2,7}]trideca-1(9),2(7),5-trien-3-one |
| F1822-0392 | N-{4,6-dioxa-10-thia-12-azatricyclo[7.3.0.0^{3,7}]dodeca-1(9),2,7,11-tetraen-11-yl}-3-(2,5-dioxopyrrolidin-1-yl)benzamide |
| F2547-0089 | 4-chloro-N-(1,1-dioxo-1lambda6-thiolan-3-yl)-N-(2-methylpropyl)-3-nitrobenzene-1-sulfonamide |
| F0846-0695 | ethyl 3-[3-(2,5-dioxopyrrolidin-1-yl)benzamido]-1-benzofuran-2-carboxylate |
| F2106-0076 | 4,7-dihydroxy-3-(4-methoxyphenyl)-2H-chromen-2-one |
| F1065-0537 | 2-({1-[2-(methylsulfanyl)phenyl]-2,5-dioxopyrrolidin-3-yl}sulfanyl)benzoic acid |
| F0817-0154 | 2-(2,5-dioxopyrrolidin-1-yl)-N-[11-(methylsulfanyl)-3,12-dithia-5,10-diazatricyclo[7.3.0.0^{2,6}]dodeca-1(9),2(6),4,7,10-pentaen-4-yl]acetamide |
| F1457-0009 | 2-{[1-(2H-1,3-benzodioxol-5-yl)-2,5-dioxopyrrolidin-3-yl]sulfanyl}benzoic acid |
| F1552-0073 | 1-(4-methylphenyl)-3-{[2-(morpholine-4-carbonyl)phenyl]sulfanyl}pyrrolidine-2,5-dione |
| F1199-0108 | 2-{[1-(3-methoxyphenyl)-2,5-dioxopyrrolidin-3-yl]sulfanyl}benzoic acid |
| F1412-0047 | 2-{[1-(4-acetylphenyl)-2,5-dioxopyrrolidin-3-yl]sulfanyl}benzoic acid |
| F1799-0447 | N'-[1-(4-bromophenyl)-2,5-dioxopyrrolidin-3-yl]-2-methylbenzohydrazide |
| F1065-0405 | 2-({2,5-dioxo-1-[4-(piperidin-1-yl)phenyl]pyrrolidin-3-yl}sulfanyl)benzoic acid |
| F1593-0033 | N-(4-chloro-2-nitrophenyl)-2-(3-oxo-3,4-dihydro-2H-1,4-benzothiazin-2-yl)acetamide |
| F2106-0049 | 4,7-dihydroxy-3-phenyl-2H-chromen-2-one |
| F0336-0491 | ethyl 2-[2-(4-chloro-3-nitrobenzamido)-1,3-thiazol-4-yl]acetate |
| F0013-0791 | 6-methyl-5-oxo-5H,6H,11H-12lambda5-isoindolo[2,1-a]quinazolin-12-ylium 4-methylbenzene-1-sulfonate |
| F0466-0005 | 3-(2,5-dioxopyrrolidin-1-yl)-N-(6-sulfamoyl-1,3-benzothiazol-2-yl)benzamide |
| F2724-1006 | 2-chloro-N-[3-(6-methoxypyridazin-3-yl)phenyl]-4-nitrobenzamide |
| F1799-0448 | N'-[1-(4-bromophenyl)-2,5-dioxopyrrolidin-3-yl]-3-methylbenzohydrazide |
| F1174-3093 | 5-{[2-(2-methylpiperidin-1-yl)-2-oxoethyl]sulfanyl}-4-phenyl-8-thia-4,6-diazatricyclo[7.4.0.0^{2,7}]trideca-1(9),2(7),5-trien-3-one |
| F6660-6033 | 1-cyclopropyl-6-fluoro-4-oxo-7-(4-phenylmethanesulfonylpiperazin-1-yl)-1,4-dihydroquinoline-3-carboxylic acid |
| F1065-0279 | 2-{[1-(4-chlorophenyl)-2,5-dioxopyrrolidin-3-yl]sulfanyl}benzoic acid |
| F1065-0354 | 2-{[1-(3-chlorophenyl)-2,5-dioxopyrrolidin-3-yl]sulfanyl}benzoic acid |
| F3019-0041 | 2-{[1-(4-acetamidophenyl)-2,5-dioxopyrrolidin-3-yl]sulfanyl}benzoic acid |
| F6660-2069 | 2-[3-(4-methoxyphenyl)-1,2,4-oxadiazol-5-yl]acetonitrile |
| F1058-0140 | 4-chloro-3-nitro-N,N-dipropylbenzene-1-sulfonamide |
| F1199-0124 | 2-{[1-(4-iodo-2-methylphenyl)-2,5-dioxopyrrolidin-3-yl]sulfanyl}pyridine-3-carboxylic acid |
| F3019-0039 | 2-{[1-(3-chloro-2-methylphenyl)-2,5-dioxopyrrolidin-3-yl]sulfanyl}benzoic acid |
| F3016-0044 | 2-(2,5-dioxopyrrolidin-1-yl)-N-{4-[(5-ethyl-1,3,4-thiadiazol-2-yl)sulfamoyl]phenyl}acetamide |
| F0779-0531 | methyl 3-[4-(2,5-dioxopyrrolidin-1-yl)benzamido]-5-phenylthiophene-2-carboxylate |
| F1799-0449 | N'-[1-(4-bromophenyl)-2,5-dioxopyrrolidin-3-yl]-4-methylbenzohydrazide |
| F0239-0433 | N-(3-methoxyphenyl)-2-methyl-4-(4-nitrophenyl)-1,3-thiazole-5-carboxamide |
| F2547-0088 | N-butyl-4-chloro-N-(1,1-dioxo-1lambda6-thiolan-3-yl)-3-nitrobenzene-1-sulfonamide |
| F0838-0514 | 4-chloro-N-(6-ethoxy-1,3-benzothiazol-2-yl)-2-nitrobenzamide |
| F3205-0093 | methyl 2-({1-[4-(dimethylamino)phenyl]-2,5-dioxopyrrolidin-3-yl}sulfanyl)benzoate |
| F3228-0173 | 3-(4-chlorophenyl)-4,7-dihydroxy-2H-chromen-2-one |
| F3023-0449 | 2-{[2,5-dioxo-1-(2-phenylethyl)pyrrolidin-3-yl]sulfanyl}benzoic acid |
| F2567-0077 | 6-{[(1-methyl-1H-imidazol-2-yl)sulfanyl]methyl}-4-oxo-4H-pyran-3-yl 4-chloro-3-nitrobenzoate |
| F0866-0364 | N-[4-(2H-1,3-benzodioxol-5-yl)-1,3-thiazol-2-yl]-4-(2,5-dioxopyrrolidin-1-yl)benzamide |
| F1065-0277 | 2-({2,5-dioxo-1-[3-(trifluoromethyl)phenyl]pyrrolidin-3-yl}sulfanyl)benzoic acid |
| F0327-0369 | 4-(2,5-dioxopyrrolidin-1-yl)-N-(4-methoxynaphthalen-1-yl)benzamide |
| F1199-0152 | 2-((1-methyl-2,5-dioxopyrrolidin-3-yl)thio)benzoic acid |
| F3385-4492 | 3-(4-bromophenyl)-4,7-dihydroxy-2H-chromen-2-one |
| F0466-0024 | 3-(2,5-dioxopyrrolidin-1-yl)-N-[4-(4-propoxyphenyl)-1,3-thiazol-2-yl]benzamide |
| F1065-0145 | 2-{[1-(4-ethoxyphenyl)-2,5-dioxopyrrolidin-3-yl]sulfanyl}benzoic acid |
| F2055-0269 | 2-chloro-N-{2-[2-(3-fluorophenyl)-1,3-thiazol-4-yl]ethyl}-4-nitrobenzamide |
| F9995-0228 | N-benzyl-4-chloro-2-nitroaniline |
| F3222-1848 | 11-acetyl-5-{[(3-fluorophenyl)methyl]sulfanyl}-4-(4-methoxyphenyl)-8-thia-4,6,11-triazatricyclo[7.4.0.0^{2,7}]trideca-1(9),2(7),5-trien-3-one |
| F1577-0004 | N'-[(3Z)-1-acetyl-5-bromo-2-oxo-2,3-dihydro-1H-indol-3-ylidene]benzohydrazide |
| F3023-0282 | 2-{[1-(2,5-dimethoxyphenyl)-2,5-dioxopyrrolidin-3-yl]sulfanyl}benzoic acid |
| F3048-0226 | 2-{[1-(3,4-dimethylphenyl)-2,5-dioxopyrrolidin-3-yl]sulfanyl}-N-(4-methoxyphenyl)acetamide |
| F3019-0040 | 2-{[1-(3-chloro-4-fluorophenyl)-2,5-dioxopyrrolidin-3-yl]sulfanyl}benzoic acid |
| F1298-0137 | 6-ethyl 3-methyl 2-[4-(2,5-dioxopyrrolidin-1-yl)benzamido]-4H,5H,6H,7H-thieno[2,3-c]pyridine-3,6-dicarboxylate |
| F8888-0334 | 2-chloro-4-(3-methyl-1,2,4-oxadiazol-5-yl)pyridine |
| F1298-0148 | 6-ethyl 3-methyl 2-[3-(2,5-dioxopyrrolidin-1-yl)benzamido]-4H,5H,6H,7H-thieno[2,3-c]pyridine-3,6-dicarboxylate |
| F3048-0060 | 2-{[1-(3,5-dimethylphenyl)-2,5-dioxopyrrolidin-3-yl]sulfanyl}-N-(3-methoxyphenyl)acetamide |
| F8881-4530 | 2-chloro-3-(3-methyl-1,2,4-oxadiazol-5-yl)pyridine |
| F0915-0501 | 3-{3-[(2-carboxyphenyl)sulfanyl]-2,5-dioxopyrrolidin-1-yl}benzoic acid |
| F3222-0033 | 7-methoxy-1,2,3,4,9,10-hexahydroacridin-9-one |
| F0700-0166 | N-[4-(2H-1,3-benzodioxol-5-yl)-1,3-thiazol-2-yl]-3-(2,5-dioxopyrrolidin-1-yl)benzamide |
| F1065-0525 | 2-({1-[4-(acetyloxy)phenyl]-2,5-dioxopyrrolidin-3-yl}sulfanyl)benzoic acid |
| F3205-0078 | 2-({1-[3-(methoxycarbonyl)thiophen-2-yl]-2,5-dioxopyrrolidin-3-yl}sulfanyl)pyridine-3-carboxylic acid |
| F1029-0066 | 3-amino-2,7-diphenyl-4lambda5-[1,3]thiazolo[3,2-a]pyrimidin-4-ylium benzenesulfonate |
| F1199-0032 | 2-({2,5-dioxo-1-[4-(trifluoromethyl)phenyl]pyrrolidin-3-yl}sulfanyl)benzoic acid |
| F1065-0321 | 2-{[1-(4-bromophenyl)-2,5-dioxopyrrolidin-3-yl]sulfanyl}benzoic acid |
| F3023-0929 | 3-{[4-(dimethylamino)phenyl]amino}-1-(naphthalen-1-yl)pyrrolidine-2,5-dione |
| F2224-0099 | 2-chloro-N-(2-methyl-4-{2-methyl-4-oxo-3H,4H-pyrido[2,3-d]pyrimidin-3-yl}phenyl)-4-nitrobenzamide |
| F3205-0061 | 2-{[1-(3-chloro-4-methoxyphenyl)-2,5-dioxopyrrolidin-3-yl]sulfanyl}benzoic acid |
| F1199-0003 | 2-{[1-(2-bromo-4-methylphenyl)-2,5-dioxopyrrolidin-3-yl]sulfanyl}benzoic acid |
| F2224-0261 | 2-chloro-N-(2-fluoro-5-{2-methyl-4-oxo-3H,4H-pyrido[2,3-d]pyrimidin-3-yl}phenyl)-4-nitrobenzamide |
| F3019-0021 | 2-{[1-(2,5-dichlorophenyl)-2,5-dioxopyrrolidin-3-yl]sulfanyl}benzoic acid |
| F3023-0014 | 2-{[1-(2,4-dichlorophenyl)-2,5-dioxopyrrolidin-3-yl]sulfanyl}benzoic acid |
| F3023-0409 | 3-[(4-methoxyphenyl)amino]-1-(naphthalen-1-yl)pyrrolidine-2,5-dione |
| F2224-0018 | 2-chloro-N-(4-{2-methyl-4-oxo-3H,4H-pyrido[2,3-d]pyrimidin-3-yl}phenyl)-4-nitrobenzamide |
| F0466-0032 | ethyl 2-[3-(2,5-dioxopyrrolidin-1-yl)benzamido]-4-phenyl-1,3-thiazole-5-carboxylate |
| F1199-0004 | 2-({1-[2-(difluoromethoxy)phenyl]-2,5-dioxopyrrolidin-3-yl}sulfanyl)benzoic acid |
| F2018-5525 | N-[2-(diethylamino)ethyl]-N-(4,6-difluoro-1,3-benzothiazol-2-yl)-3-(2,5-dioxopyrrolidin-1-yl)benzamide hydrochloride |
| F1936-0008 | 6-hydroxy-2-phenyl-7-(prop-2-en-1-yl)-4H-chromen-4-one |
| F3385-5916 | 2-{[(6-chloro-7-hydroxy-2-oxo-2H-chromen-4-yl)methyl]sulfanyl}-4,6-dimethylpyridine-3-carbonitrile |
| F1298-0252 | 3,6-diethyl 2-[3-(2,5-dioxopyrrolidin-1-yl)benzamido]-4H,5H,6H,7H-thieno[2,3-c]pyridine-3,6-dicarboxylate |
| F3382-3365 | ethyl 6-fluoro-1-methyl-4-oxo-7-(pyrrolidin-1-yl)-1,4-dihydroquinoline-3-carboxylate |
| F3023-0834 | 3-[(4-ethoxyphenyl)amino]-1-(1-phenylethyl)pyrrolidine-2,5-dione |
| F0466-0061 | ethyl 3-[3-(2,5-dioxopyrrolidin-1-yl)benzamido]-5-phenylthiophene-2-carboxylate |
| F3292-0442 | 2-{4-methoxy-6-methyl-2H,5H,6H,7H,8H-[1,3]dioxolo[4,5-g]isoquinolin-5-yl}-1-(4-methylphenyl)ethan-1-one |
| F0327-0350 | ethyl 2-[4-(2,5-dioxopyrrolidin-1-yl)benzamido]-4-phenyl-1,3-thiazole-5-carboxylate |
| F2607-0311 | 2-(4-ethylpiperazin-1-yl)-1,3-benzothiazol-6-yl 3-(2,5-dioxopyrrolidin-1-yl)benzoate |
| F9995-0534 | 4'-methoxy-[1,1'-biphenyl]-4-carbaldehyde |
| F9995-0540 | 3'-methoxy-[1,1'-biphenyl]-4-carbaldehyde |
| F1192-0005 | 1-(2-chlorophenyl)-3-(1,2,3,4-tetrahydroquinolin-1-yl)pyrrolidine-2,5-dione |
| F3023-0450 | 2-({1-[2-(methoxycarbonyl)phenyl]-2,5-dioxopyrrolidin-3-yl}sulfanyl)benzoic acid |
| F1298-0241 | 3,6-diethyl 2-[4-(2,5-dioxopyrrolidin-1-yl)benzamido]-4H,5H,6H,7H-thieno[2,3-c]pyridine-3,6-dicarboxylate |
| F2018-1842 | N-(5,6-dimethoxy-1,3-benzothiazol-2-yl)-N-[2-(dimethylamino)ethyl]-3-(2,5-dioxopyrrolidin-1-yl)benzamide hydrochloride |
| F1457-0041 | 2-{[1-(4-methyl-2-nitrophenyl)-2,5-dioxopyrrolidin-3-yl]sulfanyl}pyridine-3-carboxylic acid |
| F2211-0016 | 6-amino-2-(4-tert-butylphenyl)-4H-chromen-4-one |
| F2018-1571 | N-[2-(dimethylamino)ethyl]-N-{4,6-dioxa-10-thia-12-azatricyclo[7.3.0.0^{3,7}]dodeca-1(9),2,7,11-tetraen-11-yl}-4-(2,5-dioxopyrrolidin-1-yl)benzamide hydrochloride |
| F0902-0900 | 2-[(2,5-dioxo-1-{2-[(trifluoromethyl)sulfanyl]phenyl}pyrrolidin-3-yl)sulfanyl]benzoic acid |
| F1065-0219 | 2-{[1-(4-nitrophenyl)-2,5-dioxopyrrolidin-3-yl]sulfanyl}pyridine-3-carboxylic acid |
| F3205-0105 | 2-({2,5-dioxo-1-[4-(trifluoromethoxy)phenyl]pyrrolidin-3-yl}sulfanyl)benzoic acid |
| F2018-2985 | N-[2-(diethylamino)ethyl]-3-(2,5-dioxopyrrolidin-1-yl)-N-(6-methoxy-1,3-benzothiazol-2-yl)benzamide hydrochloride |
| F2018-3874 | N-[2-(diethylamino)ethyl]-3-(2,5-dioxopyrrolidin-1-yl)-N-(4-methoxy-1,3-benzothiazol-2-yl)benzamide hydrochloride |
| F0287-0039 | 3-[(1E)-[(2-chloro-4-nitrophenyl)imino]methyl]-2-{[(furan-2-yl)methyl]amino}-9-methyl-4H-pyrido[1,2-a]pyrimidin-4-one |
| F2862-0070 | 1-(4-chloro-3-nitrobenzoyl)-2,3-dihydro-1H-indole-2-carboxamide |
| F1065-0366 | 1-benzyl-3-{[4-(piperidin-1-yl)phenyl]amino}pyrrolidine-2,5-dione |
| F1799-0241 | 1-(4-bromophenyl)-3-[(3-methylphenyl)amino]pyrrolidine-2,5-dione |
| F1383-0025 | 2-({1-[3-(methoxycarbonyl)-4,5,6,7-tetrahydro-1-benzothiophen-2-yl]-2,5-dioxopyrrolidin-3-yl}sulfanyl)pyridine-3-carboxylic acid |
| F2146-0721 | 4-ethoxy-3-ethyl-2,3-dihydro-1,3-benzothiazol-2-imine; 4-methylbenzene-1-sulfonic acid |
| F3139-0181 | (2E)-1-(5-chloro-2-hydroxyphenyl)-3-(thiophen-2-yl)prop-2-en-1-one |
| F1065-0747 | 2-({1-[2-(ethoxycarbonyl)phenyl]-2,5-dioxopyrrolidin-3-yl}sulfanyl)benzoic acid |
| F3096-1216 | 4-chloro-N-[4-(piperidin-1-yl)phenyl]benzamide |
| F3048-0087 | methyl 4-[3-({[(2-ethylphenyl)carbamoyl]methyl}sulfanyl)-2,5-dioxopyrrolidin-1-yl]benzoate |
| F3048-0066 | N-(3-bromophenyl)-2-{[1-(4-methoxyphenyl)-2,5-dioxopyrrolidin-3-yl]sulfanyl}acetamide |
| F5252-0200 | N-(3,4-dichlorophenyl)-4-{[1-(4-methylphenyl)-1H-1,2,3,4-tetrazol-5-yl]methyl}piperazine-1-carboxamide |
| F1199-0057 | 2-{[1-(3-cyano-4,5,6,7-tetrahydro-1-benzothiophen-2-yl)-2,5-dioxopyrrolidin-3-yl]sulfanyl}benzoic acid |
| F1383-0007 | 2-({1-[2-(3,4-dimethoxyphenyl)ethyl]-2,5-dioxopyrrolidin-3-yl}sulfanyl)benzoic acid |
| F3210-0017 | 5-(4-chloro-2-nitrophenyl)-2-methyl-N-[3-(2-oxopropyl)-1,2,4-thiadiazol-5-yl]furan-3-carboxamide |
| F2019-1588 | N-[3-(dimethylamino)propyl]-N-{4,6-dioxa-10-thia-12-azatricyclo[7.3.0.0^{3,7}]dodeca-1(9),2,7,11-tetraen-11-yl}-3-(2,5-dioxopyrrolidin-1-yl)benzamide hydrochloride |
| F0466-0043 | N-(2-benzoyl-4-methylphenyl)-3-(2,5-dioxopyrrolidin-1-yl)benzamide |
| F3023-0304 | 2-{[1-(4-ethoxy-2-nitrophenyl)-2,5-dioxopyrrolidin-3-yl]sulfanyl}pyridine-3-carboxylic acid |
| F3385-0117 | 8-[(azepan-1-yl)methyl]-7-hydroxy-3-(4-methoxyphenyl)-4H-chromen-4-one |
| F0343-0002 | 2-(1,3-benzothiazol-2-yl)quinoline |
| F3023-0383 | 1-(2-chlorophenyl)-3-[(4-ethylphenyl)amino]pyrrolidine-2,5-dione |
| F3386-5305 | N,N-dibenzyl-4-methylbenzamide |
| F2899-2239 | 3-(2,5-dioxopyrrolidin-1-yl)-N-(6-fluoro-1,3-benzothiazol-2-yl)-N-[(furan-2-yl)methyl]benzamide |
| F3023-0308 | 2-{[1-(4-chloro-2-nitrophenyl)-2,5-dioxopyrrolidin-3-yl]sulfanyl}pyridine-3-carboxylic acid |
| F3115-0039 | 1-(2-bromo-4-methylphenyl)-3-[(4-fluorophenyl)amino]pyrrolidine-2,5-dione |
| F0758-0117 | 2-methyl-3-phenyl-7-(propan-2-yloxy)-4H-chromen-4-one |
| F1199-0101 | 2-({1-[4-(ethoxycarbonyl)phenyl]-2,5-dioxopyrrolidin-3-yl}sulfanyl)benzoic acid |
| F2146-0718 | 3-ethyl-4-methoxy-7-methyl-2,3-dihydro-1,3-benzothiazol-2-imine; 4-methylbenzene-1-sulfonic acid |
| F3023-0287 | 2-({1-[3-(methoxycarbonyl)-6-methyl-4,5,6,7-tetrahydro-1-benzothiophen-2-yl]-2,5-dioxopyrrolidin-3-yl}sulfanyl)pyridine-3-carboxylic acid |
| F2146-0709 | 4-methylbenzene-1-sulfonic acid; 6-ethoxy-3-ethyl-2,3-dihydro-1,3-benzothiazol-2-imine |
| F1190-0498 | 2-(2-chlorophenyl)-6-methoxy-3,4-dihydro-2H-1-benzopyran-4-one |
| F2040-0165 | 2-chloro-N-[1-(furan-2-carbonyl)-1,2,3,4-tetrahydroquinolin-6-yl]-4-nitrobenzamide |
| F3139-0687 | 3-(3,4-dihydro-2H-1,5-benzodioxepin-7-yl)-6-ethyl-7-methoxy-4H-chromen-4-one |
| F3325-0132 | 3-{[4-(dimethylamino)phenyl]amino}-1-[3-(trifluoromethyl)phenyl]pyrrolidine-2,5-dione |
| F3023-0397 | 3-[(4-methylphenyl)amino]-1-(2-phenylethyl)pyrrolidine-2,5-dione |
| F0440-0391 | N-[4-bromo-2-(2-chlorobenzoyl)phenyl]-3-(2,5-dioxopyrrolidin-1-yl)-2-oxopropanamide |
| F2146-0732 | 3-(2-ethoxyethyl)-5,6-dimethyl-2,3-dihydro-1,3-benzothiazol-2-imine; 4-methylbenzene-1-sulfonic acid |
| F0327-0377 | N-(2-benzoyl-4-methylphenyl)-4-(2,5-dioxopyrrolidin-1-yl)benzamide |
| F0758-0026 | 7-ethoxy-2,8-dimethyl-3-phenyl-4H-chromen-4-one |
| F0196-0379 | 3-(2,3-dihydro-1,4-benzodioxin-6-yl)-6-ethyl-7-methoxy-4H-chromen-4-one |
| F2146-0731 | 3-(2-ethoxyethyl)-5,7-dimethyl-2,3-dihydro-1,3-benzothiazol-2-imine; 4-methylbenzene-1-sulfonic acid |
| F1593-0029 | 3-{[4-(morpholin-4-yl)phenyl]amino}-1-(naphthalen-1-yl)pyrrolidine-2,5-dione |
| F2384-0320 | N-(1-butyl-2-oxo-1,2,3,4-tetrahydroquinolin-6-yl)-3-chlorobenzamide |
| F2384-0258 | N-(1-butyl-2-oxo-1,2,3,4-tetrahydroquinolin-6-yl)-4-chlorobenzamide |
| F3139-0624 | 7-methoxy-2-(4-methylphenyl)-4H-chromen-4-one |
| F3095-3041 | (2E)-3-(4-ethoxy-3-methoxyphenyl)-N,N-bis(2-methylpropyl)prop-2-enamide |
| F0758-0076 | 7-ethoxy-8-methyl-3-phenyl-4H-chromen-4-one |
| F3205-0064 | 2-({1-[3-(methoxycarbonyl)thiophen-2-yl]-2,5-dioxopyrrolidin-3-yl}sulfanyl)benzoic acid |
| F1751-0037 | 1-(4-nitrophenyl)-3-{[(2E)-1,3,3-trimethyl-2,3-dihydro-1H-indol-2-ylidene]methyl}pyrrolidine-2,5-dione |
| F3097-0487 | 5-hydroxy-7-methoxy-2-phenyl-4H-chromen-4-one |
| F0433-0006 | N-(2,3,5-trichloro-4-hydroxyphenyl)benzenesulfonamide |
| F3023-0384 | 1-(2-ethoxyphenyl)-3-[(4-ethylphenyl)amino]pyrrolidine-2,5-dione |
| F1589-0512 | 5-(2-chloro-4-nitrophenyl)-N-(4-acetamidophenyl)furan-2-carboxamide |
| F2146-0729 | 4-methylbenzene-1-sulfonic acid; 6-bromo-3-(2-ethoxyethyl)-2,3-dihydro-1,3-benzothiazol-2-imine |
| F1913-0659 | ethyl 3-propylhexanoate |
| F2018-4001 | N-[2-(diethylamino)ethyl]-3-(2,5-dioxopyrrolidin-1-yl)-N-(4-ethoxy-1,3-benzothiazol-2-yl)benzamide hydrochloride |
| F3139-0640 | 6-methoxy-2-(4-methylphenyl)-4H-chromen-4-one |
| F0737-0246 | 4-(2,5-dioxopyrrolidin-1-yl)-N-{11-methyl-3,10-dithia-5,12-diazatricyclo[7.3.0.0^{2,6}]dodeca-1(9),2(6),4,7,11-pentaen-4-yl}benzamide |
| F1190-0499 | 2-(2-chlorophenyl)-6-methyl-3,4-dihydro-2H-1-benzopyran-4-one |
| F6660-4201 | 4-[5-(1H-pyrrol-2-yl)-1,2,4-oxadiazol-3-yl]pyridine |
| F3139-0623 | 6-bromo-2-(3,4-dimethoxyphenyl)-4H-chromen-4-one |
| F3139-1043 | 3-(2,3-dihydro-1,4-benzodioxin-6-yl)-7-ethoxy-6-ethyl-4H-chromen-4-one |
| F3023-0286 | 2-({1-[3-(methoxycarbonyl)-5-methylthiophen-2-yl]-2,5-dioxopyrrolidin-3-yl}sulfanyl)benzoic acid |
| F3386-1384 | N-(5-chloro-2-hydroxyphenyl)-2-oxo-2H-chromene-6-sulfonamide |
| F2147-0933 | 1-(4-iodophenyl)-4-propyl-2,6,7-trioxabicyclo[2.2.2]octane |
| F3023-0880 | 3-[(4-ethoxyphenyl)amino]-1-[3-(trifluoromethyl)phenyl]pyrrolidine-2,5-dione |
| F3385-2858 | 3-(2-methoxyphenyl)-7-(2-oxo-2-phenylethoxy)-4H-chromen-4-one |
| F1243-0007 | 1-chloronaphthalen-2-yl N,N-diethylcarbamate |
| F1457-0027 | 2-({1-[3-(methoxycarbonyl)-4,5-dimethylthiophen-2-yl]-2,5-dioxopyrrolidin-3-yl}sulfanyl)benzoic acid |
| F3385-0004 | 6-methyl-2-(4-methylphenyl)-4H-chromen-4-one |
| F3139-0613 | 2-(2-fluorophenyl)-6-methyl-4H-chromen-4-one |
| F0779-0619 | N-[4-(1,3-benzothiazol-2-yl)-1,3-thiazol-2-yl]-3-(2,5-dioxopyrrolidin-1-yl)benzamide |
| F3139-0641 | 2-(2-chlorophenyl)-6-methoxy-4H-chromen-4-one |
| F0196-0383 | 3-(2H-1,3-benzodioxol-5-yl)-7-methoxy-2-methyl-6-propyl-4H-chromen-4-one |
| F3139-0629 | 6-fluoro-2-(4-methylphenyl)-4H-chromen-4-one |
| F0327-0373 | N-(2-benzoyl-4-chlorophenyl)-4-(2,5-dioxopyrrolidin-1-yl)benzamide |
| F3385-0990 | 7-[(2-methylprop-2-en-1-yl)oxy]-3-phenyl-4H-chromen-4-one |
| F0196-0377 | 3-(3,4-dihydro-2H-1,5-benzodioxepin-7-yl)-7-methoxy-6-propyl-4H-chromen-4-one |
| F1420-0613 | N-[6-(dimethylsulfamoyl)-1,3-benzothiazol-2-yl]-4-(2,5-dioxopyrrolidin-1-yl)benzamide |
| F3139-0642 | 2-(4-chlorophenyl)-6-methoxy-4H-chromen-4-one |
| F1916-0009 | 4-(4-formylphenoxy)benzaldehyde |
| F1065-0148 | 1-(2,3-dimethylphenyl)-3-(N-phenylhydroxyamino)pyrrolidine-2,5-dione |
| F2206-0129 | 2-chloro-N-(4-{7-methylimidazo[1,2-a]pyrimidin-2-yl}phenyl)-4-nitrobenzamide |
| F0400-0026 | 7-chloro-2-phenyl-4H-chromen-4-one |
| F0472-0061 | 4-hydroxy-3-[1-(1H-indol-3-yl)-2-nitroethyl]-2H-chromen-2-one |
| F2638-0066 | 2-chloro-N-[4-(2-methyl-4-oxo-3,4-dihydroquinazolin-3-yl)phenyl]-4-nitrobenzamide |
| F0921-2538 | 4-chloronaphthalen-1-yl N,N-diethylcarbamate |
| F2666-0047 | 4-chloro-3-nitro-N-{2-oxo-1-azatricyclo[6.3.1.0^{4,12}]dodeca-4,6,8(12)-trien-6-yl}benzamide |
| F2638-0065 | 2-chloro-N-[3-(2-methyl-4-oxo-3,4-dihydroquinazolin-3-yl)phenyl]-4-nitrobenzamide |
| F3394-0325 | N-(3-acetylphenyl)-2-({12-oxo-11-phenyl-7-thia-9,11-diazatricyclo[6.4.0.0^{2,6}]dodeca-1(8),2(6),9-trien-10-yl}sulfanyl)acetamide |
| F0825-0278 | N-(4-oxo-2-sulfanylidene-1,3-thiazolidin-3-yl)naphthalene-1-carboxamide |
| F3139-0635 | 2-(2-chlorophenyl)-6-fluoro-4H-chromen-4-one |
| F0327-0376 | N-(2-benzoyl-4-bromophenyl)-4-(2,5-dioxopyrrolidin-1-yl)benzamide |
| F3139-0964 | 7-[(3-methylbut-2-en-1-yl)oxy]-3-phenyl-4H-chromen-4-one |
| F2231-0026 | N-[6-(dimethylsulfamoyl)-1,3-benzothiazol-2-yl]-3-(2,5-dioxopyrrolidin-1-yl)benzamide |
| F1920-0045 | 2-(4-chlorophenyl)-6-methyl-4H-chromen-4-one |
| F3023-0381 | 1-[2-(3,4-dimethoxyphenyl)ethyl]-3-[(4-ethylphenyl)amino]pyrrolidine-2,5-dione |
| F3096-1151 | 4-bromo-N-(4-chloro-2-nitrophenyl)benzamide |
| F3139-0634 | 2-(2,4-dichlorophenyl)-7-methoxy-4H-chromen-4-one |
| F3139-0626 | 6-chloro-2-(4-methylphenyl)-4H-chromen-4-one |
| F3023-0886 | 1-{4-[(difluoromethyl)sulfanyl]phenyl}-3-[(4-ethoxyphenyl)amino]pyrrolidine-2,5-dione |
| F3205-0104 | methyl 2-(3-{[2-(methoxycarbonyl)phenyl]sulfanyl}-2,5-dioxopyrrolidin-1-yl)thiophene-3-carboxylate |
| F3139-0625 | 2-(4-chlorophenyl)-6-fluoro-4H-chromen-4-one |
| F3385-3067 | 8-{[benzyl(methyl)amino]methyl}-3-(3,4-dimethoxyphenyl)-7-hydroxy-4H-chromen-4-one |
| F0779-0470 | 2-(2,5-dioxopyrrolidin-1-yl)-N-{5-thia-3-azatetracyclo[6.6.1.0^{2,6}.0^{11,15}]pentadeca-1(15),2(6),3,7,11,13-hexaen-4-yl}acetamide |
| F0918-0101 | 4-chlorophenyl benzenesulfonate |
| F3139-0616 | 6-bromo-2-(4-methylphenyl)-4H-chromen-4-one |
| F0196-1271 | 3-(3,4-dihydro-2H-1,5-benzodioxepin-7-yl)-4-oxo-6-propyl-4H-chromen-7-yl acetate |
| F3386-4724 | phenyl 4-bromobenzene-1-sulfonate |
| F3139-0619 | 6-chloro-2-(2-fluorophenyl)-4H-chromen-4-one |
| F3139-0637 | 6-chloro-2-(4-fluorophenyl)-4H-chromen-4-one |
| F1751-0008 | 2-{[1-(naphthalen-1-yl)-2,5-dioxopyrrolidin-3-yl]sulfanyl}pyridine-3-carboxylic acid |
| F2664-0047 | 4-chloro-3-nitro-N-{11-oxo-1-azatricyclo[6.3.1.0^{4,12}]dodeca-4(12),5,7-trien-6-yl}benzamide |
| F3139-0617 | 6-bromo-2-(2-fluorophenyl)-4H-chromen-4-one |
| F3228-0111 | 6,8-dichloro-2-(3,4,5-trimethoxyphenyl)-4H-chromen-4-one |
| F2666-0117 | 4-chloro-N-{3-methyl-2-oxo-1-azatricyclo[6.3.1.0^{4,12}]dodeca-4,6,8(12)-trien-6-yl}-3-nitrobenzamide |
| F1065-0535 | 2-({1-[3-(ethoxycarbonyl)-4,5-dimethylthiophen-2-yl]-2,5-dioxopyrrolidin-3-yl}sulfanyl)benzoic acid |
| F0466-0012 | 3-(2,5-dioxopyrrolidin-1-yl)-N'-[(2E)-3-methyl-2,3-dihydro-1,3-benzothiazol-2-ylidene]benzohydrazide |
| F0882-0657 | 4-(2,5-dioxopyrrolidin-1-yl)-N-[(2E)-4-methoxy-3-methyl-2,3-dihydro-1,3-benzothiazol-2-ylidene]benzamide |
| F3374-0266 | N-(2-chloro-4-nitrophenyl)-4-(1,3-dioxo-2,3-dihydro-1H-isoindol-2-yl)benzamide |
| F2271-0114 | 4-chloro-N-[2-(1-propyl-1,2,3,4-tetrahydroquinolin-6-yl)ethyl]benzamide |
| F3385-2912 | 7-[2-(4-methoxyphenyl)-2-oxoethoxy]-3-phenyl-4H-chromen-4-one |
| F2147-0935 | trimethyl[2-(4-{4-propyl-2,6,7-trioxabicyclo[2.2.2]octan-1-yl}phenyl)ethynyl]silane |
| F2668-0047 | 4-chloro-3-nitro-N-{2-oxo-1-azatricyclo[7.3.1.0^{5,13}]trideca-5,7,9(13)-trien-7-yl}benzamide |
| F3023-0922 | 1-[(2H-1,3-benzodioxol-5-yl)methyl]-3-[4-(4-fluorophenyl)piperazin-1-yl]pyrrolidine-2,5-dione |
| F2049-0482 | N-(1-benzoyl-1,2,3,4-tetrahydroquinolin-7-yl)-4-methylbenzamide |
| F3386-3206 | 4-chloro-5-{[(4-methylphenyl)methyl]amino}-2-phenyl-2,3-dihydropyridazin-3-one |
| F2049-0480 | N-(1-benzoyl-1,2,3,4-tetrahydroquinolin-7-yl)-3-methylbenzamide |
| F3139-0610 | 6-bromo-2-(2-chlorophenyl)-4H-chromen-4-one |
| F1420-1251 | N-[6-(diethylsulfamoyl)-1,3-benzothiazol-2-yl]-4-(2,5-dioxopyrrolidin-1-yl)benzamide |
| F2874-0132 | 4-chloro-N-[4-(1-methyl-6-oxo-1,6-dihydropyridazin-3-yl)phenyl]-3-nitrobenzamide |
| F2748-0227 | N-(4-chloro-3-nitrophenyl)-2-(1,1,3-trioxo-2,3-dihydro-1lambda6,2-benzothiazol-2-yl)acetamide |
| F3139-0992 | 3-(2-methoxyphenyl)-7-[2-(4-methoxyphenyl)-2-oxoethoxy]-4H-chromen-4-one |
| F0349-0205 | 1-benzoyl-3-(2-ethylphenyl)thiourea |
| F3139-0618 | 6-chloro-2-(4-chlorophenyl)-4H-chromen-4-one |
| F0078-0024 | 1-benzyl-3-(N-phenylhydroxyamino)pyrrolidine-2,5-dione |
| F3139-0611 | 6-bromo-2-(4-chlorophenyl)-4H-chromen-4-one |
| F0808-0261 | N-(2,3-dichloro-4-hydroxynaphthalen-1-yl)benzenesulfonamide |
| F2548-0070 | 1'-(4-chloro-3-nitrobenzoyl)-3,4-dihydrospiro[1-benzopyran-2,4'-piperidine]-4-one |
| F3139-0991 | 3-(2,3-dihydro-1,4-benzodioxin-6-yl)-6-ethyl-7-[(2-methylprop-2-en-1-yl)oxy]-4H-chromen-4-one |
| F1620-0061 | 4-(2,5-dioxopyrrolidin-1-yl)-N-[(2E)-3-methyl-2,3-dihydro-1,3-benzothiazol-2-ylidene]benzamide |
| F3228-0128 | 7-hydroxy-4-oxo-3-phenyl-4H-chromene-8-carbaldehyde |
| F1365-1296 | 2-(2,5-dioxopyrrolidin-1-yl)-N-[(2Z)-3-(prop-2-en-1-yl)-6-sulfamoyl-2,3-dihydro-1,3-benzothiazol-2-ylidene]acetamide |
| F1814-1152 | 2-(2,5-dioxopyrrolidin-1-yl)-N-[(2Z)-3-ethyl-5,7-dimethyl-2,3-dihydro-1,3-benzothiazol-2-ylidene]acetamide |
| F2749-0247 | N-(4-chloro-3-nitrophenyl)-3-(1,1,3-trioxo-2,3-dihydro-1lambda6,2-benzothiazol-2-yl)propanamide |
| F3023-0021 | 2-({1-[3-(methoxycarbonyl)-5-propylthiophen-2-yl]-2,5-dioxopyrrolidin-3-yl}sulfanyl)benzoic acid |
| F0808-0278 | N-(3-chloro-4-hydroxyphenyl)-4-(propan-2-yl)benzene-1-sulfonamide |
| F1814-1627 | 4-(2,5-dioxopyrrolidin-1-yl)-N-[(2Z)-4-fluoro-3-methyl-2,3-dihydro-1,3-benzothiazol-2-ylidene]benzamide |
| F2750-0113 | N-(4-chloro-3-nitrophenyl)-2-(1,1,3-trioxo-2,3-dihydro-1lambda6,2-benzothiazol-2-yl)propanamide |
| F2794-0084 | 4-chloro-N-[4-(1,1-dioxo-1lambda6,2-thiazinan-2-yl)phenyl]-3-nitrobenzamide |
| F0328-0304 | 5-chloro-N-(2-methyl-1,3-dioxo-2,3-dihydro-1H-isoindol-5-yl)-2-nitrobenzamide |
| F1814-1143 | N-[(2Z)-4,6-dichloro-3-methyl-2,3-dihydro-1,3-benzothiazol-2-ylidene]-2-(2,5-dioxopyrrolidin-1-yl)acetamide |
| F0834-1075 | 3-(2,5-dioxopyrrolidin-1-yl)-N-[(2E)-6-fluoro-3-methyl-2,3-dihydro-1,3-benzothiazol-2-ylidene]benzamide |
| F1814-1675 | 3-(2,5-dioxopyrrolidin-1-yl)-N-[(2Z)-4-fluoro-3-methyl-2,3-dihydro-1,3-benzothiazol-2-ylidene]benzamide |
| F0466-0014 | N-[(2E)-3,6-dimethyl-2,3-dihydro-1,3-benzothiazol-2-ylidene]-3-(2,5-dioxopyrrolidin-1-yl)benzamide |
| F0328-0311 | 5-chloro-N-(1,3-dioxo-2,3-dihydro-1H-isoindol-4-yl)-2-nitrobenzamide |
| F3386-1389 | N-(5-chloro-2-hydroxyphenyl)-4-methoxynaphthalene-1-sulfonamide |
| F1365-0108 | 2-(2,5-dioxopyrrolidin-1-yl)-N-[(2Z)-6-methyl-3-(prop-2-en-1-yl)-2,3-dihydro-1,3-benzothiazol-2-ylidene]acetamide |
| F1815-1185 | 2-(2,5-dioxopyrrolidin-1-yl)-N-[(2Z)-4-methyl-3-(prop-2-en-1-yl)-2,3-dihydro-1,3-benzothiazol-2-ylidene]acetamide |
| F1009-0410 | 4-(2,5-dioxopyrrolidin-1-yl)-N-[(2E)-6-methoxy-3-methyl-2,3-dihydro-1,3-benzothiazol-2-ylidene]benzamide |
| F3139-1067 | 3-(2,3-dihydro-1,4-benzodioxin-6-yl)-4-oxo-6-propyl-4H-chromen-7-yl propanoate |
| F1575-0079 | 2-(benzyloxy)-5-chloro-4-nitroaniline |
| F3386-1375 | N-(5-chloro-2-hydroxyphenyl)-4-ethoxy-3-methylbenzene-1-sulfonamide |
| F0834-1073 | 3-(2,5-dioxopyrrolidin-1-yl)-N-[(2E)-4-methoxy-3-methyl-2,3-dihydro-1,3-benzothiazol-2-ylidene]benzamide |
| F0536-0736 | 3-(2,5-dioxopyrrolidin-1-yl)-N-[(2E)-3,4,6-trimethyl-2,3-dihydro-1,3-benzothiazol-2-ylidene]benzamide |
| F1814-0649 | 3-(2,5-dioxopyrrolidin-1-yl)-N-[(2Z)-3,5,7-trimethyl-2,3-dihydro-1,3-benzothiazol-2-ylidene]benzamide |
| F2743-0102 | 1-benzyl-N-(4-chloro-3-nitrophenyl)-2-oxo-1,2-dihydropyridine-3-carboxamide |
| F2744-0102 | 1-benzyl-N-(4-chloro-3-nitrophenyl)-6-oxo-1,6-dihydropyridine-3-carboxamide |
| F1814-1673 | N-[(2Z)-4,6-difluoro-3-methyl-2,3-dihydro-1,3-benzothiazol-2-ylidene]-3-(2,5-dioxopyrrolidin-1-yl)benzamide |
| F2874-0017 | 4-chloro-N-[3-(1-methyl-6-oxo-1,6-dihydropyridazin-3-yl)phenyl]-3-nitrobenzamide |
| F1814-0650 | 3-(2,5-dioxopyrrolidin-1-yl)-N-[(2Z)-3,4,7-trimethyl-2,3-dihydro-1,3-benzothiazol-2-ylidene]benzamide |
| F3139-1013 | 3-(2-methoxyphenyl)-4-oxo-4H-chromen-7-yl 3,4-dimethoxybenzoate |
| F0222-0027 | 3-(N-phenylhydroxyamino)-1-(2-propoxyphenyl)pyrrolidine-2,5-dione |
| F0536-0560 | 4-(2,5-dioxopyrrolidin-1-yl)-N-[(2E)-3,4,6-trimethyl-2,3-dihydro-1,3-benzothiazol-2-ylidene]benzamide |
| F3385-3844 | 7-(2-bromoethoxy)-3-(4-methoxyphenyl)-4H-chromen-4-one |
| F1826-0075 | 6-methyl-3-nitro-2-oxo-1,2-dihydropyridin-4-yl 4-chloro-3-nitrobenzoate |
| F1381-0066 | N-[(2E)-4-chloro-3-methyl-2,3-dihydro-1,3-benzothiazol-2-ylidene]-3-(2,5-dioxopyrrolidin-1-yl)benzamide |
| F3325-0165 | N-(4-chloro-3-nitrophenyl)-2-(3-oxo-3,4-dihydro-2H-1,4-benzothiazin-2-yl)acetamide |
| F1814-0651 | 3-(2,5-dioxopyrrolidin-1-yl)-N-[(2Z)-4-methoxy-3,7-dimethyl-2,3-dihydro-1,3-benzothiazol-2-ylidene]benzamide |
| F0494-0156 | 1-[5-(4-chloro-3-nitrophenyl)furan-2-carbothioyl]piperidine |
| F2670-0047 | N-(1-acetyl-1,2,3,4-tetrahydroquinolin-7-yl)-4-chloro-3-nitrobenzamide |
| F0611-0515 | 4-chloro-N-[2-(7-methyl-2-oxo-1,2-dihydroquinolin-3-yl)ethyl]-3-nitrobenzamide |
| F1381-1021 | 4-(2,5-dioxopyrrolidin-1-yl)-N-[(2E)-3-ethyl-6-fluoro-2,3-dihydro-1,3-benzothiazol-2-ylidene]benzamide |
| F1814-1676 | 3-(2,5-dioxopyrrolidin-1-yl)-N-[(2Z)-3-ethyl-4-fluoro-2,3-dihydro-1,3-benzothiazol-2-ylidene]benzamide |
| F2744-0456 | N-(4-chloro-3-nitrophenyl)-1-[(2-methylphenyl)methyl]-6-oxo-1,6-dihydropyridine-3-carboxamide |
| F0347-0397 | 4-chloro-3-nitro-N-(1-phenylethyl)benzamide |
| F2743-0456 | N-(4-chloro-3-nitrophenyl)-1-[(2-methylphenyl)methyl]-2-oxo-1,2-dihydropyridine-3-carboxamide |
| F1359-0444 | 4-(2,5-dioxopyrrolidin-1-yl)-N-[(2Z)-3-(2-methoxyethyl)-2,3-dihydro-1,3-benzothiazol-2-ylidene]benzamide |
| F1168-0056 | 4-(4-chlorophenyl)-6-phenyl-1,2,3,4-tetrahydropyrimidine-2-thione |
| F2106-0042 | 7-(2-bromoethoxy)-3-phenyl-4H-chromen-4-one |
| F0611-0842 | 4-chloro-N-[2-(7,8-dimethyl-2-oxo-1,2-dihydroquinolin-3-yl)ethyl]-3-nitrobenzamide |
| F1529-0125 | (2E)-3-[5-(2-chloro-4-nitrophenyl)furan-2-yl]-2-cyano-N-[(furan-2-yl)methyl]prop-2-enamide |
| F2743-0810 | N-(4-chloro-3-nitrophenyl)-1-[(3-methylphenyl)methyl]-2-oxo-1,2-dihydropyridine-3-carboxamide |
| F2743-1164 | N-(4-chloro-3-nitrophenyl)-1-[(4-methylphenyl)methyl]-2-oxo-1,2-dihydropyridine-3-carboxamide |
| F2744-0810 | N-(4-chloro-3-nitrophenyl)-1-[(3-methylphenyl)methyl]-6-oxo-1,6-dihydropyridine-3-carboxamide |
| F3139-0439 | propan-2-yl 2-{[3-(2,3-dihydro-1,4-benzodioxin-6-yl)-6-ethyl-4-oxo-4H-chromen-7-yl]oxy}acetate |
| F5581-0865 | 3-(2,5-dioxopyrrolidin-1-yl)-N-(4-ethoxy-1,3-benzothiazol-2-yl)-N-[2-(1H-pyrazol-1-yl)ethyl]benzamide |
| F2663-0278 | 8-(2-chloro-4-nitrobenzoyl)-4-(thiophene-2-sulfonyl)-1-oxa-4,8-diazaspiro[4.5]decane |
| F2743-0220 | N-(4-chloro-3-nitrophenyl)-1-[(3-fluorophenyl)methyl]-2-oxo-1,2-dihydropyridine-3-carboxamide |
| F2744-1400 | N-(4-chloro-3-nitrophenyl)-1-[(2-fluorophenyl)methyl]-6-oxo-1,6-dihydropyridine-3-carboxamide |
| F2743-0338 | N-(4-chloro-3-nitrophenyl)-1-[(4-fluorophenyl)methyl]-2-oxo-1,2-dihydropyridine-3-carboxamide |
| F2743-1400 | N-(4-chloro-3-nitrophenyl)-1-[(2-fluorophenyl)methyl]-2-oxo-1,2-dihydropyridine-3-carboxamide |
| F0466-0010 | 3-(2,5-dioxopyrrolidin-1-yl)-N-[(2Z)-6-ethoxy-3-methyl-2,3-dihydro-1,3-benzothiazol-2-ylidene]benzamide |
| F2744-0338 | N-(4-chloro-3-nitrophenyl)-1-[(4-fluorophenyl)methyl]-6-oxo-1,6-dihydropyridine-3-carboxamide |
| F2524-0973 | methyl 2-[(5Z)-5-{[2-(2,5-dioxopyrrolidin-1-yl)acetyl]imino}-10,13-dioxa-4-thia-6-azatricyclo[7.4.0.0^{3,7}]trideca-1,3(7),8-trien-6-yl]acetate |
| F0611-0733 | 4-chloro-N-[2-(5,8-dimethyl-2-oxo-1,2-dihydroquinolin-3-yl)ethyl]-3-nitrobenzamide |
| F3386-1374 | 2,5-dichloro-N-(5-chloro-2-hydroxyphenyl)benzene-1-sulfonamide |
| F0762-0364 | (2Z)-N-(2-chloro-4-nitrophenyl)-2-cyano-3-(4-methylphenyl)prop-2-enamide |
| F0327-0383 | N-(1,2-dihydroacenaphthylen-5-yl)-4-(2,5-dioxopyrrolidin-1-yl)benzamide |
| F0611-0079 | 4-chloro-N-[2-(6,7-dimethyl-2-oxo-1,2-dihydroquinolin-3-yl)ethyl]-3-nitrobenzamide |
| F2843-0251 | N-(4-chlorophenyl)-N-(2-fluorobenzoyl)piperidine-1-carboxamide |
| F1814-1674 | 3-(2,5-dioxopyrrolidin-1-yl)-N-[(2Z)-3-ethyl-4,6-difluoro-2,3-dihydro-1,3-benzothiazol-2-ylidene]benzamide |
| F2843-0327 | N-(3-chlorophenyl)-N-(2-fluorobenzoyl)piperidine-1-carboxamide |
| F1814-0652 | N-[(2Z)-4,7-dimethoxy-3-methyl-2,3-dihydro-1,3-benzothiazol-2-ylidene]-3-(2,5-dioxopyrrolidin-1-yl)benzamide |
| F1365-1782 | 3-(2,5-dioxopyrrolidin-1-yl)-N-[(2Z)-6-methyl-3-(prop-2-yn-1-yl)-2,3-dihydro-1,3-benzothiazol-2-ylidene]benzamide |
| F5526-0036 | 4-chloro-3-nitro-N-(2-phenoxyethyl)benzamide |
| F3386-1382 | 5-bromo-N-(5-chloro-2-hydroxyphenyl)-2-methoxy-4-methylbenzene-1-sulfonamide |
| F0273-0019 | 4-chloro-N-(1,1-dioxo-2,3-dihydro-1lambda6-thiophen-3-yl)-N-(4-methylphenyl)-3-nitrobenzamide |
| F1359-2178 | 3-(2,5-dioxopyrrolidin-1-yl)-N-[(2Z)-3-(2-ethoxyethyl)-2,3-dihydro-1,3-benzothiazol-2-ylidene]benzamide |
| F1814-1626 | 4-(2,5-dioxopyrrolidin-1-yl)-N-[(2Z)-3-ethyl-4,6-difluoro-2,3-dihydro-1,3-benzothiazol-2-ylidene]benzamide |
| F0624-0081 | (4-oxo-3,4-dihydro-1,2,3-benzotriazin-3-yl)methyl 4-chloro-3-nitrobenzoate |
| F1175-0217 | 4-(4-chloro-3-nitrobenzamido)benzoic acid |
| F1816-0723 | 3-(2,5-dioxopyrrolidin-1-yl)-N-[(2Z)-4-methyl-3-(prop-2-yn-1-yl)-2,3-dihydro-1,3-benzothiazol-2-ylidene]benzamide |
| F5526-0150 | 4-chloro-N-[2-(4-fluorophenoxy)ethyl]-3-nitrobenzamide |
| F0611-0624 | 4-chloro-N-[2-(7-methoxy-2-oxo-1,2-dihydroquinolin-3-yl)ethyl]-3-nitrobenzamide |
| F0700-0159 | 3-(2,5-dioxopyrrolidin-1-yl)-N-[4-(2-oxo-2H-chromen-3-yl)-1,3-thiazol-2-yl]benzamide |
| F3139-0763 | 3-(2-methoxyphenyl)-4-oxo-4H-chromen-7-yl 3,4,5-trimethoxybenzoate |
| F2515-1588 | N-{4,6-dioxa-10-thia-12-azatricyclo[7.3.0.0^{3,7}]dodeca-1(9),2,7,11-tetraen-11-yl}-3-(2,5-dioxopyrrolidin-1-yl)-N-[(pyridin-3-yl)methyl]benzamide |
| F1816-0721 | 3-(2,5-dioxopyrrolidin-1-yl)-N-[(2Z)-4-methoxy-3-(prop-2-yn-1-yl)-2,3-dihydro-1,3-benzothiazol-2-ylidene]benzamide |
| F2843-0261 | N-(4-chlorophenyl)-N-(2,6-difluorobenzoyl)piperidine-1-carboxamide |
| F2843-0337 | N-(3-chlorophenyl)-N-(2,6-difluorobenzoyl)piperidine-1-carboxamide |
| F1814-0659 | 3-(2,5-dioxopyrrolidin-1-yl)-N-[(2Z)-3-ethyl-4-methoxy-7-methyl-2,3-dihydro-1,3-benzothiazol-2-ylidene]benzamide |
| F1199-0085 | 1-(4-iodo-2-methylphenyl)-3-{[4-(morpholin-4-yl)phenyl]amino}pyrrolidine-2,5-dione |
| F2515-1571 | N-{4,6-dioxa-10-thia-12-azatricyclo[7.3.0.0^{3,7}]dodeca-1(9),2,7,11-tetraen-11-yl}-4-(2,5-dioxopyrrolidin-1-yl)-N-[(pyridin-3-yl)methyl]benzamide |
| F1359-1518 | 3-(2,5-dioxopyrrolidin-1-yl)-N-[(2Z)-6-methoxy-3-(2-methoxyethyl)-2,3-dihydro-1,3-benzothiazol-2-ylidene]benzamide |
| F1365-0768 | N-[(2Z)-6-bromo-3-(prop-2-en-1-yl)-2,3-dihydro-1,3-benzothiazol-2-ylidene]-2-(2,5-dioxopyrrolidin-1-yl)acetamide |
| F3342-0440 | N-[2-hydroxy-3-(piperidin-1-yl)propyl]-N-(4-methoxyphenyl)-4-methylbenzene-1-sulfonamide; benzoic acid |
| F1823-0787 | 3-(2,5-dioxopyrrolidin-1-yl)-N-[(5E)-6-methyl-10,13-dioxa-4-thia-6-azatricyclo[7.4.0.0^{3,7}]trideca-1,3(7),8-trien-5-ylidene]benzamide |
| F3385-4474 | 7-(2-bromoethoxy)-3-(3,4-dimethoxyphenyl)-4H-chromen-4-one |
| F1365-0858 | 3-(2,5-dioxopyrrolidin-1-yl)-N-[(2Z)-6-fluoro-3-(prop-2-en-1-yl)-2,3-dihydro-1,3-benzothiazol-2-ylidene]benzamide |
| F0762-0368 | (2Z)-N-(2-chloro-4-nitrophenyl)-3-(4-chlorophenyl)-2-cyanoprop-2-enamide |
| F1366-0066 | methyl 2-[(2Z)-2-{[3-(2,5-dioxopyrrolidin-1-yl)benzoyl]imino}-6-methyl-2,3-dihydro-1,3-benzothiazol-3-yl]acetate |
| F1365-1104 | 4-(2,5-dioxopyrrolidin-1-yl)-N-[(2Z)-6-methanesulfonyl-3-(prop-2-en-1-yl)-2,3-dihydro-1,3-benzothiazol-2-ylidene]benzamide |
| F1825-0063 | N-(2-chloro-4-nitrophenyl)-1-[(4-fluorophenyl)methoxy]-2-oxo-1,2-dihydropyridine-3-carboxamide |
| F3385-1741 | 3-(2,3-dihydro-1,4-benzodioxin-6-yl)-7-[2-(4-methoxyphenyl)-2-oxoethoxy]-4H-chromen-4-one |
| F0752-0089 | diphenyl(sulfanyl)-lambda5-phosphanethione; morpholine |
| F1815-0523 | 4-(2,5-dioxopyrrolidin-1-yl)-N-[(2Z)-4-methoxy-3-(prop-2-en-1-yl)-2,3-dihydro-1,3-benzothiazol-2-ylidene]benzamide |
| F1815-0721 | 3-(2,5-dioxopyrrolidin-1-yl)-N-[(2Z)-4-methoxy-3-(prop-2-en-1-yl)-2,3-dihydro-1,3-benzothiazol-2-ylidene]benzamide |
| F1297-0137 | 4-(2,5-dioxopyrrolidin-1-yl)-N-[(2E)-4-ethoxy-3-ethyl-2,3-dihydro-1,3-benzothiazol-2-ylidene]benzamide |
| F1365-2310 | N-[(2Z)-6-chloro-3-(prop-2-yn-1-yl)-2,3-dihydro-1,3-benzothiazol-2-ylidene]-3-(2,5-dioxopyrrolidin-1-yl)benzamide |
| F1359-2574 | 3-(2,5-dioxopyrrolidin-1-yl)-N-[(2Z)-3-(2-ethoxyethyl)-6-fluoro-2,3-dihydro-1,3-benzothiazol-2-ylidene]benzamide |
| F1673-4093 | 4-chloro-N-{[4-(dimethylamino)phenyl]methyl}-N-(1,1-dioxo-1lambda6-thiolan-3-yl)-3-nitrobenzamide |
| F5526-0591 | N-[2-(2H-1,3-benzodioxol-5-yloxy)ethyl]-4-chloro-3-nitrobenzamide |
| F1366-0858 | methyl 2-[(2Z)-2-{[3-(2,5-dioxopyrrolidin-1-yl)benzoyl]imino}-6-fluoro-2,3-dihydro-1,3-benzothiazol-3-yl]acetate |
| F3115-0023 | 2-{[1-(4-iodophenyl)-2,5-dioxopyrrolidin-3-yl]sulfanyl}benzoic acid |
| F0327-0296 | 4-(2,5-dioxopyrrolidin-1-yl)-N-(6-nitro-1,3-benzothiazol-2-yl)benzamide |
| F1359-1782 | 3-(2,5-dioxopyrrolidin-1-yl)-N-[(2Z)-3-(2-ethoxyethyl)-6-methyl-2,3-dihydro-1,3-benzothiazol-2-ylidene]benzamide |
| F1412-0032 | 2-{[1-(naphthalen-1-yl)-2,5-dioxopyrrolidin-3-yl]sulfanyl}benzoic acid |
| F1816-0725 | N-[(2Z)-4-chloro-3-(prop-2-yn-1-yl)-2,3-dihydro-1,3-benzothiazol-2-ylidene]-3-(2,5-dioxopyrrolidin-1-yl)benzamide |
| F1365-3366 | 3-(2,5-dioxopyrrolidin-1-yl)-N-[(2Z)-6-ethoxy-3-(prop-2-yn-1-yl)-2,3-dihydro-1,3-benzothiazol-2-ylidene]benzamide |
| F1825-0064 | N-(4-chloro-2-nitrophenyl)-1-[(4-fluorophenyl)methoxy]-2-oxo-1,2-dihydropyridine-3-carboxamide |
| F2743-1636 | N-(4-chloro-3-nitrophenyl)-1-[(3-nitrophenyl)methyl]-2-oxo-1,2-dihydropyridine-3-carboxamide |
| F2744-1636 | N-(4-chloro-3-nitrophenyl)-1-[(3-nitrophenyl)methyl]-6-oxo-1,6-dihydropyridine-3-carboxamide |
| F1366-0372 | methyl (2Z)-2-{[2-(2,5-dioxopyrrolidin-1-yl)acetyl]imino}-3-(2-methoxy-2-oxoethyl)-2,3-dihydro-1,3-benzothiazole-6-carboxylate |
| F0239-0350 | 2-methyl-N-(3-methylphenyl)-4-(4-nitrophenyl)-1,3-thiazole-5-carboxamide |
| F1199-0098 | 2-{[1-(4-iodo-2-methylphenyl)-2,5-dioxopyrrolidin-3-yl]sulfanyl}benzoic acid |
| F1814-0480 | 4-(2,5-dioxopyrrolidin-1-yl)-N-[(2Z)-3-ethyl-4,7-dimethoxy-2,3-dihydro-1,3-benzothiazol-2-ylidene]benzamide |
| F1814-0660 | 3-(2,5-dioxopyrrolidin-1-yl)-N-[(2Z)-3-ethyl-4,7-dimethoxy-2,3-dihydro-1,3-benzothiazol-2-ylidene]benzamide |
| F1673-4952 | 4-chloro-N-(1,1-dioxo-1lambda6-thiolan-3-yl)-N-[(4-methoxyphenyl)methyl]-3-nitrobenzamide |
| F6739-8494 | [(3,4-dimethoxyphenyl)methyl](methyl)(1-phenylethyl)amine |
| F1816-0722 | 3-(2,5-dioxopyrrolidin-1-yl)-N-[(2Z)-4-ethoxy-3-(prop-2-yn-1-yl)-2,3-dihydro-1,3-benzothiazol-2-ylidene]benzamide |
| F1820-0521 | methyl 2-[(2Z)-2-{[4-(2,5-dioxopyrrolidin-1-yl)benzoyl]imino}-5,7-dimethyl-2,3-dihydro-1,3-benzothiazol-3-yl]acetate |
| F1820-0719 | methyl 2-[(2Z)-2-{[3-(2,5-dioxopyrrolidin-1-yl)benzoyl]imino}-5,7-dimethyl-2,3-dihydro-1,3-benzothiazol-3-yl]acetate |
| F1365-0240 | ethyl (2Z)-2-{[2-(2,5-dioxopyrrolidin-1-yl)acetyl]imino}-3-(prop-2-en-1-yl)-2,3-dihydro-1,3-benzothiazole-6-carboxylate |
| F1673-3710 | 4-chloro-N-[(4-chlorophenyl)methyl]-N-(1,1-dioxo-1lambda6-thiolan-3-yl)-3-nitrobenzamide |
| F0709-0274 | (2Z)-N-(4-chloro-2-nitrophenyl)-2-cyano-3-(3,4-dimethoxyphenyl)prop-2-enamide |
| F1366-2178 | ethyl 2-[(2Z)-2-{[3-(2,5-dioxopyrrolidin-1-yl)benzoyl]imino}-2,3-dihydro-1,3-benzothiazol-3-yl]acetate |
| F1757-0140 | 1-(4-benzoylphenoxy)-3-(4-bromo-3,5-dimethyl-1H-pyrazol-1-yl)propan-2-ol |
| F1366-1518 | methyl 2-[(2Z)-2-{[3-(2,5-dioxopyrrolidin-1-yl)benzoyl]imino}-6-methoxy-2,3-dihydro-1,3-benzothiazol-3-yl]acetate |
| F1751-0027 | 1-(naphthalen-1-yl)-3-[2-(4-nitrophenyl)hydrazin-1-yl]pyrrolidine-2,5-dione |
| F0611-0297 | 4-chloro-N-[2-(5,8-dimethoxy-2-oxo-1,2-dihydroquinolin-3-yl)ethyl]-3-nitrobenzamide |
| F1366-4950 | ethyl 2-[(2Z)-2-{[3-(2,5-dioxopyrrolidin-1-yl)benzoyl]imino}-4-fluoro-2,3-dihydro-1,3-benzothiazol-3-yl]acetate |
| F1673-4175 | 4-chloro-N-(1,1-dioxo-1lambda6-thiolan-3-yl)-N-[(4-ethylphenyl)methyl]-3-nitrobenzamide |
| F1365-2046 | methyl (2Z)-2-{[3-(2,5-dioxopyrrolidin-1-yl)benzoyl]imino}-3-(prop-2-yn-1-yl)-2,3-dihydro-1,3-benzothiazole-6-carboxylate |
| F0709-0029 | (2Z)-N-(4-chloro-2-nitrophenyl)-2-cyano-3-(3-nitrophenyl)prop-2-enamide |
| F1366-1782 | ethyl 2-[(2Z)-2-{[3-(2,5-dioxopyrrolidin-1-yl)benzoyl]imino}-6-methyl-2,3-dihydro-1,3-benzothiazol-3-yl]acetate |
| F1359-0708 | N-[(2Z)-6-bromo-3-(2-methoxyethyl)-2,3-dihydro-1,3-benzothiazol-2-ylidene]-4-(2,5-dioxopyrrolidin-1-yl)benzamide |
| F1365-2442 | N-[(2Z)-6-bromo-3-(prop-2-yn-1-yl)-2,3-dihydro-1,3-benzothiazol-2-ylidene]-3-(2,5-dioxopyrrolidin-1-yl)benzamide |
| F5520-0095 | 2-({5-[4-(4-chloro-3-nitrobenzoyl)piperazin-1-yl]-1,3,4-thiadiazol-2-yl}sulfanyl)acetamide |
| F1365-2424 | N-[(2Z)-6-bromo-3-(prop-2-yn-1-yl)-2,3-dihydro-1,3-benzothiazol-2-ylidene]-4-(2,5-dioxopyrrolidin-1-yl)benzamide |
| F2660-0054 | 2-chloro-N-[2-(furan-2-yl)-2-(1,2,3,4-tetrahydroisoquinolin-2-yl)ethyl]-4-nitrobenzamide |
| F2823-0236 | 4-chloro-3-nitro-N-{4-oxo-1-phenyl-1H,4H,5H-pyrazolo[3,4-d]pyrimidin-5-yl}benzamide |
| F0327-0298 | 4-(2,5-dioxopyrrolidin-1-yl)-N-(4-methoxy-6-nitro-1,3-benzothiazol-2-yl)benzamide |
| F0327-0341 | 4-(2,5-dioxopyrrolidin-1-yl)-N-[4-(4-nitrophenyl)-1,3-thiazol-2-yl]benzamide |
| F0398-0056 | 4-methyl-7-[(3-methylphenyl)methoxy]-2H-chromen-2-one |
| F2147-0938 | (2E)-3-{[1,1'-biphenyl]-4-yl}prop-2-enoic acid |
| F0700-0155 | 3-(2,5-dioxopyrrolidin-1-yl)-N-(4-methoxy-6-nitro-1,3-benzothiazol-2-yl)benzamide |
| F0433-0272 | 4-chloro-N-(1,5-dimethyl-3-oxo-2-phenyl-2,3-dihydro-1H-pyrazol-4-yl)-3-nitrobenzene-1-sulfonamide |
| F1359-0312 | methyl (2Z)-2-{[4-(2,5-dioxopyrrolidin-1-yl)benzoyl]imino}-3-(2-methoxyethyl)-2,3-dihydro-1,3-benzothiazole-6-carboxylate |
| F2823-0872 | 4-chloro-N-[1-(3,4-dimethylphenyl)-4-oxo-1H,4H,5H-pyrazolo[3,4-d]pyrimidin-5-yl]-3-nitrobenzamide |
| F3139-0671 | (2E)-3-(2,4-dimethoxyphenyl)-1-(2-hydroxy-5-methylphenyl)prop-2-en-1-one |
| F1536-0042 | (2E)-3-[5-(2-chloro-4-nitrophenyl)furan-2-yl]-2-[(E)-piperidine-1-carbonyl]prop-2-enenitrile |
| F1365-0330 | methyl (2Z)-2-{[3-(2,5-dioxopyrrolidin-1-yl)benzoyl]imino}-3-(prop-2-en-1-yl)-2,3-dihydro-1,3-benzothiazole-6-carboxylate |
| F1190-0643 | (2E)-1-(5-chloro-2-hydroxyphenyl)-3-(3,4-dimethoxyphenyl)prop-2-en-1-one |
| F2659-0054 | 2-chloro-N-[2-(2,3-dihydro-1H-indol-1-yl)-2-(furan-2-yl)ethyl]-4-nitrobenzamide |
| F0466-0049 | N-(9,10-dioxo-9,10-dihydroanthracen-2-yl)-3-(2,5-dioxopyrrolidin-1-yl)benzamide |
| F1740-0134 | 2-{[1-(4-methylphenyl)-2,5-dioxopyrrolidin-3-yl]sulfanyl}-N-(4-sulfamoylphenyl)acetamide |
| F0327-0370 | N-(9,10-dioxo-9,10-dihydroanthracen-1-yl)-4-(2,5-dioxopyrrolidin-1-yl)benzamide |
| F3139-0228 | (2E)-3-(3,5-dichloro-2-hydroxyphenyl)-1-phenylprop-2-en-1-one |
| F1065-0536 | 2-{[1-(4-nitrophenyl)-2,5-dioxopyrrolidin-3-yl]sulfanyl}benzoic acid |
| F3139-0551 | (2E)-1-(5-chloro-2-hydroxyphenyl)-3-(3,4,5-trimethoxyphenyl)prop-2-en-1-one |
| F1365-1896 | ethyl (2Z)-2-{[4-(2,5-dioxopyrrolidin-1-yl)benzoyl]imino}-3-(prop-2-yn-1-yl)-2,3-dihydro-1,3-benzothiazole-6-carboxylate |
| F2524-0807 | 3-(2,5-dioxopyrrolidin-1-yl)-N-[(5Z)-6-(prop-2-en-1-yl)-10,13-dioxa-4-thia-6-azatricyclo[7.4.0.0^{3,7}]trideca-1,3(7),8-trien-5-ylidene]benzamide |
| F0709-0469 | (2E)-3-[5-(4-chloro-2-nitrophenyl)furan-2-yl]-2-[(E)-piperidine-1-carbonyl]prop-2-enenitrile |
| F3188-0038 | methyl 2-{[1-(4-ethylphenyl)-2,5-dioxopyrrolidin-3-yl]sulfanyl}benzoate |
| F6660-0797 | 4-(4-bromophenyl)-6-(4-methylphenyl)-1,2,3,4-tetrahydropyrimidine-2-thione |
| F3215-0073 | (5E)-5-{[5-(2-chloro-4-nitrophenyl)furan-2-yl]methylidene}-3-(morpholin-4-yl)-2-sulfanylidene-1,3-thiazolidin-4-one |
| F0440-0350 | 2-(2,5-dioxopyrrolidin-1-yl)-N-[4-(4-phenoxyphenyl)-1,3-thiazol-2-yl]acetamide |
| F1199-0005 | 2-{[1-(2-methyl-5-nitrophenyl)-2,5-dioxopyrrolidin-3-yl]sulfanyl}benzoic acid |
| F0417-2081 | ethyl 2-{[5-(4-chloro-3-nitrobenzamido)-1,3,4-thiadiazol-2-yl]sulfanyl}acetate |
| F3088-3663 | 5-(4-chloro-2-nitrophenyl)-N-(3-methoxyphenyl)furan-2-carboxamide |
| F3386-3752 | 4-chlorophenyl 4-methylbenzene-1-sulfonate |
| F2099-0016 | 2-chloro-N-[4-(6-methanesulfonylpyridazin-3-yl)phenyl]-4-nitrobenzamide |
| F2098-0016 | 2-chloro-N-[3-(6-methanesulfonylpyridazin-3-yl)phenyl]-4-nitrobenzamide |
| F2823-0395 | 4-chloro-N-[1-(4-methylphenyl)-4-oxo-1H,4H,5H-pyrazolo[3,4-d]pyrimidin-5-yl]-3-nitrobenzamide |
| F1589-0503 | 5-(2-chloro-4-nitrophenyl)-N-(4-sulfamoylphenyl)furan-2-carboxamide |
| F2843-0123 | 4-chloro-3-nitro-N-phenyl-N-(piperidine-1-carbonyl)benzamide |
| F1065-0450 | 2-{[1-(4-methyl-2-nitrophenyl)-2,5-dioxopyrrolidin-3-yl]sulfanyl}benzoic acid |
| F2099-0132 | 2-chloro-N-{4-[6-(ethanesulfonyl)pyridazin-3-yl]phenyl}-4-nitrobenzamide |
| F2098-0132 | 2-chloro-N-{3-[6-(ethanesulfonyl)pyridazin-3-yl]phenyl}-4-nitrobenzamide |
| F3188-0042 | methyl 2-{[2,5-dioxo-1-(2-phenylethyl)pyrrolidin-3-yl]sulfanyl}benzoate |
| F0561-0612 | 4-chloro-N-[2-(2-methylphenyl)-5-oxo-2H,4H,6H-5lambda4-thieno[3,4-c]pyrazol-3-yl]-3-nitrobenzamide |
| F3215-0287 | 5-(4-chloro-2-nitrophenyl)-2-methyl-N-(4-sulfamoylphenyl)furan-3-carboxamide |
| F1065-0770 | 2-({1-[4-chloro-3-(trifluoromethyl)phenyl]-2,5-dioxopyrrolidin-3-yl}sulfanyl)benzoic acid |
| F0561-0605 | 4-chloro-3-nitro-N-{5-oxo-2-phenyl-2H,4H,6H-5lambda4-thieno[3,4-c]pyrazol-3-yl}benzamide |
| F0834-0675 | 1-[3-(1,3-benzoxazol-2-yl)phenyl]-3-[2-(2,5-dioxopyrrolidin-1-yl)acetyl]thiourea |
| F2724-0269 | 4-chloro-N-{3-[6-(morpholin-4-yl)pyridazin-3-yl]phenyl}-3-nitrobenzamide |
| F1210-0012 | N-benzyl-2-({5,6-dimethyl-4-oxo-3-phenyl-3H,4H-thieno[2,3-d]pyrimidin-2-yl}sulfanyl)acetamide |
| F1678-0159 | 2-({5,6-dimethyl-4-oxo-3-phenyl-3H,4H-thieno[2,3-d]pyrimidin-2-yl}sulfanyl)-N-(2-phenylethyl)acetamide |
| F2688-0046 | 3-(4-chloro-3-nitrobenzamido)-1-benzofuran-2-carboxamide |
| F1537-0036 | N-(4-chloro-2-nitrophenyl)-2-[3-oxo-6-(trifluoromethyl)-3,4-dihydro-2H-1,4-benzothiazin-2-yl]acetamide |
| F3386-3753 | 4-chlorophenyl 4-bromobenzene-1-sulfonate |
| F2510-0139 | 4-oxo-6-[(pyrimidin-2-ylsulfanyl)methyl]-4H-pyran-3-yl 4-chloro-3-nitrobenzoate |
| F3115-0024 | 2-{[1-(4-methoxy-2-nitrophenyl)-2,5-dioxopyrrolidin-3-yl]sulfanyl}benzoic acid |
| F0561-0608 | 4-chloro-N-[2-(4-fluorophenyl)-5-oxo-2H,4H,6H-5lambda4-thieno[3,4-c]pyrazol-3-yl]-3-nitrobenzamide |
| F0526-2261 | 5-[(2H-1,3-benzodioxol-5-yl)methyl]-2-[3-(2,5-dioxopyrrolidin-1-yl)benzamido]-4-methylthiophene-3-carboxamide |
| F3098-1286 | 8-{[benzyl(methyl)amino]methyl}-7-hydroxy-3-phenyl-4H-chromen-4-one |
| F0561-0610 | 4-chloro-N-[2-(2,3-dimethylphenyl)-5-oxo-2H,4H,6H-5lambda4-thieno[3,4-c]pyrazol-3-yl]-3-nitrobenzamide |
| F0541-0352 | 4-chloro-N-[2-(4-methylphenyl)-5,5-dioxo-2H,4H,6H-5lambda6-thieno[3,4-c]pyrazol-3-yl]-3-nitrobenzamide |
| F2566-0077 | 6-{[(4-methyl-1,3-thiazol-2-yl)sulfanyl]methyl}-4-oxo-4H-pyran-3-yl 4-chloro-3-nitrobenzoate |
| F0561-0611 | 4-chloro-N-[2-(2,4-dimethylphenyl)-5-oxo-2H,4H,6H-5lambda4-thieno[3,4-c]pyrazol-3-yl]-3-nitrobenzamide |
| F3374-0032 | N-(4-chloro-3-nitrophenyl)-3-(morpholine-4-sulfonyl)benzamide |
| F1175-0222 | N-(1,3-benzothiazol-2-yl)-4-chloro-3-nitrobenzamide |
| F2102-0079 | [1,1'-biphenyl]-4-carbohydrazide |
| F3115-0051 | 2-{[1-(4-ethoxy-2-nitrophenyl)-2,5-dioxopyrrolidin-3-yl]sulfanyl}benzoic acid |
| F2724-0679 | 4-chloro-N-{4-[6-(morpholin-4-yl)pyridazin-3-yl]phenyl}-3-nitrobenzamide |
| F1740-0135 | 2-{[1-(4-bromophenyl)-2,5-dioxopyrrolidin-3-yl]sulfanyl}-N-(4-sulfamoylphenyl)acetamide |
| F2510-0140 | 6-{[(4-methylpyrimidin-2-yl)sulfanyl]methyl}-4-oxo-4H-pyran-3-yl 4-chloro-3-nitrobenzoate |
| F2047-0088 | 2-chloro-N-[2-(4-methoxybenzenesulfonyl)-2-(thiophen-2-yl)ethyl]-4-nitrobenzamide |
| F0561-0607 | 4-chloro-N-[2-(3-chlorophenyl)-5-oxo-2H,4H,6H-5lambda4-thieno[3,4-c]pyrazol-3-yl]-3-nitrobenzamide |
| F1015-0042 | 4-(2,5-dioxopyrrolidin-1-yl)-N-[5-(4-nitrobenzenesulfonyl)-1,3-thiazol-2-yl]benzamide |
| F0541-0156 | 4-chloro-N-{5,5-dioxo-2-phenyl-2H,4H,6H-5lambda6-thieno[3,4-c]pyrazol-3-yl}-3-nitrobenzamide |
| F2724-1089 | 4-chloro-N-[4-(6-methoxypyridazin-3-yl)phenyl]-3-nitrobenzamide |
| F1589-0518 | 5-(2-chloro-4-nitrophenyl)-N-[2-(3,4-dimethoxyphenyl)ethyl]furan-2-carboxamide |
| F3019-0012 | 1-[2-(3,4-dimethoxyphenyl)ethyl]-3-[2-(4-nitrophenyl)hydrazin-1-yl]pyrrolidine-2,5-dione |
| F2047-0205 | 2-chloro-N-[2-(4-fluoro-3-methylbenzenesulfonyl)-2-(thiophen-2-yl)ethyl]-4-nitrobenzamide |
| F2663-0042 | 8-(4-chloro-3-nitrobenzoyl)-4-(4-methylbenzenesulfonyl)-1-oxa-4,8-diazaspiro[4.5]decane |
| F2070-1681 | 4-chloro-N-{[1-(4-fluorophenyl)-1H-1,2,3,4-tetrazol-5-yl]methyl}-3-nitrobenzamide |
| F0541-0940 | 4-chloro-N-[2-(2,3-dimethylphenyl)-5,5-dioxo-2H,4H,6H-5lambda6-thieno[3,4-c]pyrazol-3-yl]-3-nitrobenzamide |
| F2510-0141 | 6-{[(4,6-dimethylpyrimidin-2-yl)sulfanyl]methyl}-4-oxo-4H-pyran-3-yl 4-chloro-3-nitrobenzoate |
| F0561-0609 | 4-chloro-N-[2-(4-methoxyphenyl)-5-oxo-2H,4H,6H-5lambda4-thieno[3,4-c]pyrazol-3-yl]-3-nitrobenzamide |
| F3385-1046 | 8-{[benzyl(methyl)amino]methyl}-7-hydroxy-3-(4-methoxyphenyl)-4H-chromen-4-one |
| F3385-0012 | 7-(2-oxo-2-phenylethoxy)-3-phenyl-4H-chromen-4-one |
| F2066-0136 | 4-{[1-(4-chloro-3-nitrobenzenesulfonyl)-3,5-dimethyl-1H-pyrazol-4-yl]sulfonyl}morpholine |
| F0541-1419 | 4-chloro-N-[2-(4-fluorophenyl)-2H,4H,6H-thieno[3,4-c]pyrazol-3-yl]-3-nitrobenzamide |
| F2070-1198 | 4-chloro-N-{[1-(3,4-difluorophenyl)-1H-1,2,3,4-tetrazol-5-yl]methyl}-3-nitrobenzamide |
| F3386-1187 | 4-chlorophenyl 4-tert-butylbenzene-1-sulfonate |
| F3222-2363 | 11-acetyl-5-{[(3-nitrophenyl)methyl]sulfanyl}-4-phenyl-8-thia-4,6,11-triazatricyclo[7.4.0.0^{2,7}]trideca-1(9),2(7),5-trien-3-one |
| F2018-4365 | N-[2-(diethylamino)ethyl]-N-{4,6-dioxa-10-thia-12-azatricyclo[7.3.0.0^{3,7}]dodeca-1(9),2,7,11-tetraen-11-yl}-4-(2,5-dioxopyrrolidin-1-yl)benzamide hydrochloride |
| F2018-4382 | N-[2-(diethylamino)ethyl]-N-{4,6-dioxa-10-thia-12-azatricyclo[7.3.0.0^{3,7}]dodeca-1(9),2,7,11-tetraen-11-yl}-3-(2,5-dioxopyrrolidin-1-yl)benzamide hydrochloride |
| F0817-0139 | 4-(2,5-dioxopyrrolidin-1-yl)-N-[11-(methylsulfanyl)-3,12-dithia-5,10-diazatricyclo[7.3.0.0^{2,6}]dodeca-1(9),2(6),4,7,10-pentaen-4-yl]benzamide |
| F0915-6513 | 2-({12-oxo-11-phenyl-7-thia-9,11-diazatricyclo[6.4.0.0^{2,6}]dodeca-1(8),2(6),9-trien-10-yl}sulfanyl)-N-(2-phenylethyl)acetamide |
| F0541-1467 | 4-chloro-N-[2-(4-methoxyphenyl)-2H,4H,6H-thieno[3,4-c]pyrazol-3-yl]-3-nitrobenzamide |
| F3139-1132 | 4-oxo-3-phenyl-4H-chromen-7-yl benzoate |
| F1740-0137 | ethyl 4-[2,5-dioxo-3-({[(4-sulfamoylphenyl)carbamoyl]methyl}sulfanyl)pyrrolidin-1-yl]benzoate |
| F3048-0088 | methyl 4-[3-({[(4-ethoxyphenyl)carbamoyl]methyl}sulfanyl)-2,5-dioxopyrrolidin-1-yl]benzoate |
| F1315-0050 | 4-hydroxy-N-(4-methylbenzenesulfonyl)-2-oxo-2H-chromene-3-carboxamide |
| F0850-4775 | 3-(2-methoxyphenyl)-4-oxo-4H-chromen-7-yl benzoate |
| F3342-0157 | benzoic acid; {3-[N-(4-fluorophenyl)4-methylbenzenesulfonamido]-2-hydroxypropyl}[(furan-2-yl)methyl]amine |
| F0614-0079 | 4-chloro-N-{4-methyl-5-oxo-5H-chromeno[4,3-b]pyridin-2-yl}-3-nitrobenzamide |
| F0239-0325 | 4-chloro-3-nitro-N-{8-thia-4,6-diazatricyclo[7.4.0.0^{2,7}]trideca-1(9),2(7),3,5-tetraen-3-yl}benzamide |
| F1529-0100 | (2E)-3-[5-(2-chloro-4-nitrophenyl)furan-2-yl]-2-(4-oxo-3,4-dihydroquinazolin-2-yl)prop-2-enenitrile |
| F0509-3783 | 4-(2,5-dioxopyrrolidin-1-yl)-N-[5-({[(2-fluorophenyl)carbamoyl]methyl}sulfanyl)-1,3,4-thiadiazol-2-yl]benzamide |
| F0507-4313 | 4-chloro-N-{[4-(4-fluorophenyl)-5-(methylsulfanyl)-4H-1,2,4-triazol-3-yl]methyl}-3-nitrobenzamide |
| F3139-1525 | 4-oxo-3-phenyl-4H-chromen-7-yl 4-methoxybenzoate |
| F2784-0548 | 4-chloro-N-[4-methyl-3-(2-oxopiperidin-1-yl)phenyl]-3-nitrobenzamide |
| F0779-0458 | 4-(2,5-dioxopyrrolidin-1-yl)-N-{5-thia-3-azatetracyclo[6.6.1.0^{2,6}.0^{11,15}]pentadeca-1(15),2(6),3,7,11,13-hexaen-4-yl}benzamide |
| F3139-1241 | 4-oxo-3-phenyl-4H-chromen-7-yl 2-methoxybenzoate |
| F1750-0122 | N-{4-[(4,6-dimethylpyrimidin-2-yl)sulfamoyl]phenyl}-3-(2,5-dioxopyrrolidin-1-yl)benzamide |
| F2224-0100 | 4-chloro-N-(2-methyl-4-{2-methyl-4-oxo-3H,4H-pyrido[2,3-d]pyrimidin-3-yl}phenyl)-3-nitrobenzamide |
| F2224-0181 | 4-chloro-N-(3-{2-methyl-4-oxo-3H,4H-pyrido[2,3-d]pyrimidin-3-yl}phenyl)-3-nitrobenzamide |
| F0779-0473 | 3-(2,5-dioxopyrrolidin-1-yl)-N-{5-thia-3-azatetracyclo[6.6.1.0^{2,6}.0^{11,15}]pentadeca-1(15),2(6),3,7,11,13-hexaen-4-yl}benzamide |
| F0817-0157 | 3-(2,5-dioxopyrrolidin-1-yl)-N-[11-(methylsulfanyl)-3,12-dithia-5,10-diazatricyclo[7.3.0.0^{2,6}]dodeca-1(9),2(6),4,7,10-pentaen-4-yl]benzamide |
| F2224-0019 | 4-chloro-N-(4-{2-methyl-4-oxo-3H,4H-pyrido[2,3-d]pyrimidin-3-yl}phenyl)-3-nitrobenzamide |
| F6782-0676 | 4-(3-chlorophenyl)-1-phenyl-1H-1,2,3-triazol-5-amine |
| F3386-4508 | 2-chlorophenyl 4-bromobenzene-1-sulfonate |
| F3139-1513 | 3-(3,4-dimethoxyphenyl)-4-oxo-4H-chromen-7-yl benzoate |
| F0808-1896 | N-(3-chloro-4-hydroxynaphthalen-1-yl)-4-fluorobenzene-1-sulfonamide |
| F3139-1236 | 4-oxo-3-phenyl-4H-chromen-7-yl 3-methoxybenzoate |
| F1750-0030 | 4-(2,5-dioxopyrrolidin-1-yl)-N-{4-[(4-methylpyrimidin-2-yl)sulfamoyl]phenyl}benzamide |
| F0327-0364 | 4-(2,5-dioxopyrrolidin-1-yl)-N-[4-(thiophen-2-yl)-5-(2,2,2-trifluoroacetyl)-1,3-thiazol-2-yl]benzamide |
| F0700-0163 | 3-(2,5-dioxopyrrolidin-1-yl)-N-[4-(thiophen-2-yl)-5-(2,2,2-trifluoroacetyl)-1,3-thiazol-2-yl]benzamide |
| F1750-0116 | N-{4-[(4,6-dimethylpyrimidin-2-yl)sulfamoyl]phenyl}-4-(2,5-dioxopyrrolidin-1-yl)benzamide |
| F3139-0282 | 3-(2-methoxyphenyl)-4-oxo-4H-chromen-7-yl 4-methoxybenzoate |
| F3139-0289 | 3-(2-methoxyphenyl)-4-oxo-4H-chromen-7-yl 3-methoxybenzoate |
| F0509-3776 | N-(5-{[2-(2,3-dihydro-1H-indol-1-yl)-2-oxoethyl]sulfanyl}-1,3,4-thiadiazol-2-yl)-4-(2,5-dioxopyrrolidin-1-yl)benzamide |
| F1243-0037 | 2-nitrophenyl N,N-diphenylcarbamate |
| F1065-0260 | 3-(1,3-benzoxazol-2-ylsulfanyl)-1-[3-(trifluoromethyl)phenyl]pyrrolidine-2,5-dione |
| F2496-1556 | 1-[5-(1-benzofuran-2-yl)-1,2-oxazol-3-yl]methyl 2-methyl benzene-1,2-dicarboxylate |
| F0808-0260 | N-(2,3-dichloro-4-hydroxynaphthalen-1-yl)-4-methylbenzene-1-sulfonamide |
| F1065-0356 | 3-[(6-ethoxy-1,3-benzothiazol-2-yl)sulfanyl]-1-phenylpyrrolidine-2,5-dione |
| F2671-0114 | 2-chloro-4-nitro-N-[1-(thiophene-2-sulfonyl)-1,2,3,4-tetrahydroquinolin-7-yl]benzamide |
| F3385-1336 | 3-(4-methoxyphenyl)-4-oxo-4H-chromen-7-yl 2-methoxybenzoate |
| F0745-0532 | 2-[2-({12-oxo-11-phenyl-7-thia-9,11-diazatricyclo[6.4.0.0^{2,6}]dodeca-1(8),2(6),9-trien-10-yl}sulfanyl)ethyl]-2,3-dihydro-1H-isoindole-1,3-dione |
| F1443-5254 | 4-(4-methylbenzenesulfonamido)-N-(1,3-thiazol-2-yl)benzene-1-sulfonamide |
| F2743-1046 | N-(4-chloro-3-nitrophenyl)-1-[(2,5-dimethylphenyl)methyl]-2-oxo-1,2-dihydropyridine-3-carboxamide |
| F3139-1535 | 3-(3,4-dimethoxyphenyl)-4-oxo-4H-chromen-7-yl 4-methoxybenzoate |
| F3385-2833 | 4-oxo-3-phenyl-4H-chromen-7-yl 2H-1,3-benzodioxole-5-carboxylate |
| F3139-1555 | 3-(3,4-dimethoxyphenyl)-4-oxo-4H-chromen-7-yl 3-methoxybenzoate |
| F1816-1188 | 2-(2,5-dioxopyrrolidin-1-yl)-N-[(2Z)-3-(prop-2-yn-1-yl)-2H,3H-naphtho[2,1-d][1,3]thiazol-2-ylidene]acetamide |
| F3385-3750 | 3-(4-methoxyphenyl)-4-oxo-4H-chromen-7-yl 2,6-dimethoxybenzoate |
| F2744-1046 | N-(4-chloro-3-nitrophenyl)-1-[(2,5-dimethylphenyl)methyl]-6-oxo-1,6-dihydropyridine-3-carboxamide |
| F3023-0416 | 1-(4-iodo-2-methylphenyl)-3-[(4-methoxyphenyl)amino]pyrrolidine-2,5-dione |
| F1814-0657 | 3-(2,5-dioxopyrrolidin-1-yl)-N-[(2Z)-3-ethyl-5,7-dimethyl-2,3-dihydro-1,3-benzothiazol-2-ylidene]benzamide |
| F1814-0653 | N-[(2Z)-7-chloro-3,4-dimethyl-2,3-dihydro-1,3-benzothiazol-2-ylidene]-3-(2,5-dioxopyrrolidin-1-yl)benzamide |
| F0709-0099 | (2Z)-N-(4-chloro-2-nitrophenyl)-2-cyano-3-(4-methylphenyl)prop-2-enamide |
| F1365-2970 | 3-(2,5-dioxopyrrolidin-1-yl)-N-[(2Z)-3-(prop-2-yn-1-yl)-6-sulfamoyl-2,3-dihydro-1,3-benzothiazol-2-ylidene]benzamide |
| F1365-1254 | 3-(2,5-dioxopyrrolidin-1-yl)-N-[(2Z)-3-(prop-2-en-1-yl)-6-sulfamoyl-2,3-dihydro-1,3-benzothiazol-2-ylidene]benzamide |
| F1365-1236 | 4-(2,5-dioxopyrrolidin-1-yl)-N-[(2Z)-3-(prop-2-en-1-yl)-6-sulfamoyl-2,3-dihydro-1,3-benzothiazol-2-ylidene]benzamide |
| F3385-3915 | 7-(2-bromoethoxy)-3-(4-methoxyphenyl)-8-methyl-4H-chromen-4-one |
| F2865-0381 | 1-(4-chloro-3-nitrobenzoyl)-4-[3-(4-fluorophenyl)-3H-[1,2,3]triazolo[4,5-d]pyrimidin-7-yl]piperazine |
| F3385-0092 | 3-(2-methoxyphenyl)-4-oxo-4H-chromen-7-yl 2H-1,3-benzodioxole-5-carboxylate |
| F2744-0928 | N-(4-chloro-3-nitrophenyl)-1-[(2-chlorophenyl)methyl]-6-oxo-1,6-dihydropyridine-3-carboxamide |
| F2743-0574 | N-(4-chloro-3-nitrophenyl)-1-[(4-chlorophenyl)methyl]-2-oxo-1,2-dihydropyridine-3-carboxamide |
| F2743-0928 | N-(4-chloro-3-nitrophenyl)-1-[(2-chlorophenyl)methyl]-2-oxo-1,2-dihydropyridine-3-carboxamide |
| F2744-1518 | N-(4-chloro-3-nitrophenyl)-1-[(3-chlorophenyl)methyl]-6-oxo-1,6-dihydropyridine-3-carboxamide |
| F2744-0574 | N-(4-chloro-3-nitrophenyl)-1-[(4-chlorophenyl)methyl]-6-oxo-1,6-dihydropyridine-3-carboxamide |
| F1575-0001 | 4-chloro-N-{4-[(difluoromethyl)sulfanyl]phenyl}-3-nitrobenzamide |
| F1365-0048 | 4-(2,5-dioxopyrrolidin-1-yl)-N-[(2Z)-6-methyl-3-(prop-2-en-1-yl)-2,3-dihydro-1,3-benzothiazol-2-ylidene]benzamide |
| F3385-0089 | 3-(4-methoxyphenyl)-4-oxo-4H-chromen-7-yl 2H-1,3-benzodioxole-5-carboxylate |
| F1816-0719 | N-[(2Z)-5,7-dimethyl-3-(prop-2-yn-1-yl)-2,3-dihydro-1,3-benzothiazol-2-ylidene]-3-(2,5-dioxopyrrolidin-1-yl)benzamide |
| F1816-0521 | N-[(2Z)-5,7-dimethyl-3-(prop-2-yn-1-yl)-2,3-dihydro-1,3-benzothiazol-2-ylidene]-4-(2,5-dioxopyrrolidin-1-yl)benzamide |
| F1359-1254 | 3-(2,5-dioxopyrrolidin-1-yl)-N-[(2Z)-3-(2-methoxyethyl)-6-sulfamoyl-2,3-dihydro-1,3-benzothiazol-2-ylidene]benzamide |
| F1814-0648 | N-[(2Z)-4,6-dichloro-3-methyl-2,3-dihydro-1,3-benzothiazol-2-ylidene]-3-(2,5-dioxopyrrolidin-1-yl)benzamide |
| F2743-0692 | N-(4-chloro-3-nitrophenyl)-1-[(2-chloro-6-fluorophenyl)methyl]-2-oxo-1,2-dihydropyridine-3-carboxamide |
| F2744-0692 | N-(4-chloro-3-nitrophenyl)-1-[(2-chloro-6-fluorophenyl)methyl]-6-oxo-1,6-dihydropyridine-3-carboxamide |
| F1359-1236 | 4-(2,5-dioxopyrrolidin-1-yl)-N-[(2Z)-3-(2-methoxyethyl)-6-sulfamoyl-2,3-dihydro-1,3-benzothiazol-2-ylidene]benzamide |
| F3385-4196 | 7-(2-bromoethoxy)-3-(4-bromophenyl)-4H-chromen-4-one |
| F1365-0594 | N-[(2Z)-6-chloro-3-(prop-2-en-1-yl)-2,3-dihydro-1,3-benzothiazol-2-ylidene]-3-(2,5-dioxopyrrolidin-1-yl)benzamide |
| F0589-0344 | 4-chloro-3-nitro-N-[2-(thiophen-2-yl)imidazo[1,2-a]pyridin-3-yl]benzamide |
| F1359-1104 | 4-(2,5-dioxopyrrolidin-1-yl)-N-[(2Z)-6-methanesulfonyl-3-(2-methoxyethyl)-2,3-dihydro-1,3-benzothiazol-2-ylidene]benzamide |
| F1366-1386 | methyl 2-[(2Z)-2-{[3-(2,5-dioxopyrrolidin-1-yl)benzoyl]imino}-6-acetamido-2,3-dihydro-1,3-benzothiazol-3-yl]acetate |
| F2865-0536 | 1-(4-chloro-3-nitrobenzoyl)-4-[3-(3-fluorophenyl)-3H-[1,2,3]triazolo[4,5-d]pyrimidin-7-yl]piperazine |
| F1815-0524 | 4-(2,5-dioxopyrrolidin-1-yl)-N-[(2Z)-4-ethoxy-3-(prop-2-en-1-yl)-2,3-dihydro-1,3-benzothiazol-2-ylidene]benzamide |
| F3385-0082 | 3-(3,4-dimethoxyphenyl)-4-oxo-4H-chromen-7-yl 2H-1,3-benzodioxole-5-carboxylate |
| F1815-0722 | 3-(2,5-dioxopyrrolidin-1-yl)-N-[(2Z)-4-ethoxy-3-(prop-2-en-1-yl)-2,3-dihydro-1,3-benzothiazol-2-ylidene]benzamide |
| F3386-3259 | naphthalen-2-yl benzenesulfonate |
| F1819-0521 | ethyl 2-[(2Z)-2-{[4-(2,5-dioxopyrrolidin-1-yl)benzoyl]imino}-5,7-dimethyl-2,3-dihydro-1,3-benzothiazol-3-yl]acetate |
| F1819-0719 | ethyl 2-[(2Z)-2-{[3-(2,5-dioxopyrrolidin-1-yl)benzoyl]imino}-5,7-dimethyl-2,3-dihydro-1,3-benzothiazol-3-yl]acetate |
| F3385-0075 | 3-(2,3-dihydro-1,4-benzodioxin-6-yl)-4-oxo-4H-chromen-7-yl 2H-1,3-benzodioxole-5-carboxylate |
| F2051-0244 | 2-chloro-N-[1-(4-fluorobenzenesulfonyl)-1,2,3,4-tetrahydroquinolin-7-yl]-4-nitrobenzamide |
| F3099-3874 | 2-(4-bromo-2-nitrophenoxy)-N-(4-chloro-3-nitrophenyl)acetamide |
| F0808-2337 | N-(3-acetyl-2-methyl-1-benzofuran-5-yl)-N-(4-chloro-3-nitrobenzenesulfonyl)acetamide |
| F1359-0198 | ethyl (2Z)-2-{[3-(2,5-dioxopyrrolidin-1-yl)benzoyl]imino}-3-(2-methoxyethyl)-2,3-dihydro-1,3-benzothiazole-6-carboxylate |
| F1359-0180 | ethyl (2Z)-2-{[4-(2,5-dioxopyrrolidin-1-yl)benzoyl]imino}-3-(2-methoxyethyl)-2,3-dihydro-1,3-benzothiazole-6-carboxylate |
| F1365-0180 | ethyl (2Z)-2-{[4-(2,5-dioxopyrrolidin-1-yl)benzoyl]imino}-3-(prop-2-en-1-yl)-2,3-dihydro-1,3-benzothiazole-6-carboxylate |
| F0651-0071 | 4-chloro-N-[(2-methyl-1H-indol-5-yl)methyl]-3-nitrobenzamide |
| F1916-0196 | 4-[4-(4-formylphenoxy)phenoxy]benzaldehyde |
| F0778-0194 | 4-chloro-N-[2-(4-methylphenyl)-2H,4H,6H-thieno[3,4-c]pyrazol-3-yl]-3-nitrobenzamide |
| F0778-0193 | 4-chloro-N-[2-(2-methylphenyl)-2H,4H,6H-thieno[3,4-c]pyrazol-3-yl]-3-nitrobenzamide |
| F1168-0081 | 4-(2-methoxynaphthalen-1-yl)-6-phenyl-1,2,3,4-tetrahydropyrimidine-2-thione |
| F0582-0076 | 4-chloro-N-[2-(1H-indol-3-ylsulfanyl)ethyl]-3-nitrobenzamide |
| F1620-0109 | 2-chloro-N-(6-methyl-1,3-benzothiazol-2-yl)-3,5-dinitrobenzamide |
| F0326-1133 | 4-chloro-N-[5-(4-methylphenyl)-1,3,4-thiadiazol-2-yl]-3-nitrobenzamide |
| F1625-0030 | 1-(4-chloro-3-nitrophenyl)-2-[(3-methylquinoxalin-2-yl)sulfanyl]ethan-1-one |
| F2098-0017 | 4-chloro-N-[3-(6-methanesulfonylpyridazin-3-yl)phenyl]-3-nitrobenzamide |
| F2099-0017 | 4-chloro-N-[4-(6-methanesulfonylpyridazin-3-yl)phenyl]-3-nitrobenzamide |
| F0326-1033 | 4-chloro-N-[5-(2-chlorophenyl)-1,3,4-thiadiazol-2-yl]-3-nitrobenzamide |
| F0778-0195 | 4-chloro-3-nitro-N-[2-(4-nitrophenyl)-2H,4H,6H-thieno[3,4-c]pyrazol-3-yl]benzamide |
| F2204-0089 | N-(4-chloro-3-nitrophenyl)-5-methyl-2,4-dioxo-3-phenyl-1H,2H,3H,4H,5H-pyrrolo[3,2-d]pyrimidine-7-carboxamide |
| F0541-0646 | 4-chloro-3-nitro-N-[2-(4-nitrophenyl)-5,5-dioxo-2H,4H,6H-5lambda6-thieno[3,4-c]pyrazol-3-yl]benzamide |
| F2099-0133 | 4-chloro-N-{4-[6-(ethanesulfonyl)pyridazin-3-yl]phenyl}-3-nitrobenzamide |
| F2098-0133 | 4-chloro-N-{3-[6-(ethanesulfonyl)pyridazin-3-yl]phenyl}-3-nitrobenzamide |
| F0095-0737 | naphthalen-2-yl 4-chlorobenzene-1-sulfonate |
| F1168-0083 | 4-[3-(benzyloxy)-4-methoxyphenyl]-6-phenyl-1,2,3,4-tetrahydropyrimidine-2-thione |
| F2722-0119 | 4-chloro-N-[2-fluoro-5-(2-methyl-4-oxo-3,4-dihydroquinazolin-3-yl)phenyl]-3-nitrobenzamide |
| F6617-4628 | N-[2-(1H-indol-3-yl)ethyl]-2-[4-(2-methylpropyl)phenyl]propanamide |
| F3097-4951 | N-(4-chloro-3-nitrophenyl)-4-(1,3-dioxo-2,3-dihydro-1H-isoindol-2-yl)benzamide |
| F2638-0069 | 4-chloro-N-[3-(2-methyl-4-oxo-3,4-dihydroquinazolin-3-yl)phenyl]-3-nitrobenzamide |
| F0589-0060 | 4-chloro-N-[2-(4-fluorophenyl)imidazo[1,2-a]pyridin-3-yl]-3-nitrobenzamide |
| F0641-0072 | ethyl 5-(4-chloro-3-nitrobenzamido)-4-oxo-3-phenyl-3H,4H-thieno[3,4-d]pyridazine-1-carboxylate |
| F0417-0064 | 4-chloro-3-nitro-N-(5-{[(phenylcarbamoyl)methyl]sulfanyl}-1,3,4-thiadiazol-2-yl)benzamide |
| F0509-2733 | 4-chloro-N-[5-({[(2-fluorophenyl)carbamoyl]methyl}sulfanyl)-1,3,4-thiadiazol-2-yl]-3-nitrobenzamide |
| F0589-0131 | 4-chloro-N-[2-(4-methoxyphenyl)imidazo[1,2-a]pyridin-3-yl]-3-nitrobenzamide |
| F2066-0144 | 1-(4-chloro-3-nitrobenzenesulfonyl)-N,3,5-trimethyl-N-phenyl-1H-pyrazole-4-sulfonamide |
| F1816-0726 | 3-(2,5-dioxopyrrolidin-1-yl)-N-[(2Z)-3-(prop-2-yn-1-yl)-2H,3H-naphtho[2,1-d][1,3]thiazol-2-ylidene]benzamide |
| F1816-0528 | 4-(2,5-dioxopyrrolidin-1-yl)-N-[(2Z)-3-(prop-2-yn-1-yl)-2H,3H-naphtho[2,1-d][1,3]thiazol-2-ylidene]benzamide |
| F2671-0110 | 4-chloro-3-nitro-N-[1-(thiophene-2-sulfonyl)-1,2,3,4-tetrahydroquinolin-7-yl]benzamide |
| F6440-4280 | N-((1-cyclopentyl-5-cyclopropyl-1H-pyrazol-3-yl)methyl)-2-(2,5-dioxopyrrolidin-1-yl)acetamide |
| F6473-3530 | 1-(1-(2-(m-tolyl)acetyl)azetidin-3-yl)pyrrolidine-2,5-dione |
| F6400-0640 | 1-(2-oxo-2-(3-(pyrrolidin-1-ylmethyl)-1,4-thiazepan-4-yl)ethyl)pyrrolidine-2,5-dione |
| F5857-5345 | 2-(2,5-dioxopyrrolidin-1-yl)-N-((1-hydroxy-2,3-dihydro-1H-inden-1-yl)methyl)acetamide |
| F3115-0061 | 3-(2-morpholinocyclohex-2-en-1-yl)-1-(p-tolyl)pyrrolidine-2,5-dione |
| F1065-0368 | 1-(4-methoxyphenyl)-3-(4-methylpiperidin-1-yl)pyrrolidine-2,5-dione |
| F3205-0010 | 1-phenyl-3-(2-(piperidin-1-yl)cyclohex-2-en-1-yl)pyrrolidine-2,5-dione |
| F3188-0100 | 1-(3,5-dimethylphenyl)-3-(2-morpholinocyclohex-2-en-1-yl)pyrrolidine-2,5-dione |
| F3023-0417 | 3-((3,4-dimethoxyphenethyl)amino)-1-methylpyrrolidine-2,5-dione |
| F3325-0021 | 1-(2-butoxyphenyl)-3-(4-methylpiperazin-1-yl)pyrrolidine-2,5-dione |
| F3205-0014 | 1-(4-methoxyphenyl)-3-(2-(piperidin-1-yl)cyclohex-2-en-1-yl)pyrrolidine-2,5-dione |
| F5823-0721 | 1-(2-((4-((1H-pyrazol-1-yl)methyl)piperidin-1-yl)sulfonyl)ethyl)piperidine-2,6-dione |
| F3238-0138 | 4-((4-bromo-3,5-dimethyl-1H-pyrazol-1-yl)sulfonyl)morpholine |
| F5831-2082 | 2-(2,6-dioxopiperidin-1-yl)-N-(1-(furan-2-yl)propan-2-yl)ethane-1-sulfonamide |
| F5831-3334 | 1-(2-((3-phenylpyrrolidin-1-yl)sulfonyl)ethyl)piperidine-2,6-dione |
| F3096-1171 | N-(3,4-dichlorophenyl)-2,6-dimethylmorpholine-4-carboxamide |
| F3083-0093 | 3-(4-bromo-3,5-dimethyl-1H-pyrazol-1-yl)-N-(4-methoxyphenyl)butanamide |
| F2733-0061 | 3-(2,5-dioxopyrrolidin-1-yl)-N-(thieno[2,3-d]pyrimidin-4-yl)benzamide |
| F5832-0313 | 2-(2,6-dioxopiperidin-1-yl)-N-((1-(4-methoxyphenyl)cyclopentyl)methyl)ethane-1-sulfonamide |
| F6416-8505 | N-(3-(2,5-dimethylfuran-3-yl)-3-hydroxypropyl)-2-(2,5-dioxopyrrolidin-1-yl)acetamide |
| F3316-0239 | N-(2-(4-chloro-3,5-dimethyl-1H-pyrazol-1-yl)ethyl)-2,4-dimethylbenzenesulfonamide |
| F1757-0424 | (4-bromo-3,5-dimethyl-1H-pyrazol-1-yl)(morpholino)methanone |
| F5832-2362 | 2-(2,5-dioxopyrrolidin-1-yl)-N-(2-(4-fluorophenyl)-2-morpholinoethyl)acetamide |
| F1800-0188 | N-(benzo[d][1,3]dioxol-5-yl)-2-(2-methyl-3,5-dioxothiomorpholino)acetamide |
| F3033-0005 | 2-(2,5-dioxopyrrolidin-1-yl)-N-mesitylacetamide |
| F1358-0095 | methyl 2-(2-(2,5-dioxopyrrolidin-1-yl)acetamido)thiophene-3-carboxylate |
| F1800-0189 | N-(2,3-dihydrobenzo[b][1,4]dioxin-6-yl)-2-(2-methyl-3,5-dioxothiomorpholino)acetamide |
| F1800-0197 | N-benzyl-2-(2-methyl-3,5-dioxothiomorpholino)acetamide |
| F1800-0163 | 2-(2-methyl-3,5-dioxothiomorpholino)-N-(p-tolyl)acetamide |
| F1800-0006 | 2-(3,5-dioxothiomorpholino)-N-phenylacetamide |
| F3188-0113 | 1-cyclopentyl-3-((4-morpholinophenyl)amino)pyrrolidine-2,5-dione |
| F1800-0045 | 2-(3,5-dioxothiomorpholino)-N-(4-fluorobenzyl)acetamide |
| F3023-0981 | 1-ethyl-3-((4-(piperidin-1-yl)phenyl)amino)pyrrolidine-2,5-dione |
| F0349-0676 | 4-bromo-N-(6-bromobenzo[d]thiazol-2-yl)-1-methyl-1H-pyrazole-5-carboxamide |
| F0700-0029 | 2-(2,5-dioxopyrrolidin-1-yl)-N-(4-(pyridin-2-yl)thiazol-2-yl)acetamide |
| F1800-0193 | 2-(2-methyl-3,5-dioxothiomorpholino)-N-(4-(trifluoromethoxy)phenyl)acetamide |
| F0466-0054 | N-(2-chloro-5-(trifluoromethyl)phenyl)-3-(2,5-dioxopyrrolidin-1-yl)benzamide |
| F2189-0119 | 1-benzyl-4-(4-bromophenyl)piperidine-2,6-dione |
| F0440-0342 | 2-(2,5-dioxopyrrolidin-1-yl)-N-(4-mesitylthiazol-2-yl)acetamide |
| F3188-0032 | ethyl 4-(1-(2-(difluoromethoxy)phenyl)-2,5-dioxopyrrolidin-3-yl)piperazine-1-carboxylate |
| F2806-0037 | N-(benzo[d]thiazol-6-yl)-4-(2,5-dioxopyrrolidin-1-yl)benzamide |
| F1786-0002 | 2,4-dichloro-6-(piperidin-1-ylsulfonyl)phenol |
| F1675-0067 | 1-((3,5-dichloro-2-hydroxyphenyl)sulfonyl)piperidine-3-carboxylic acid |
| F2019-0987 | N-(3-(dimethylamino)propyl)-2-(2,5-dioxopyrrolidin-1-yl)-N-(4-methylbenzo[d]thiazol-2-yl)acetamide hydrochloride |
| F3115-0087 | ethyl 4-(1-(4-acetamidophenyl)-2,5-dioxopyrrolidin-3-yl)piperazine-1-carboxylate |
| F3325-0024 | 2-((1-allyl-2,5-dioxopyrrolidin-3-yl)thio)nicotinic acid |
| F1799-0540 | 2-((2,5-dioxo-1-phenylpyrrolidin-3-yl)thio)acetic acid |
| F1813-1163 | N-(4,6-difluorobenzo[d]thiazol-2-yl)-4-(2,5-dioxopyrrolidin-1-yl)benzamide |
| F3316-0241 | N-(2-(4-chloro-3,5-dimethyl-1H-pyrazol-1-yl)ethyl)-2,4,6-trimethylbenzenesulfonamide |
| F3205-0026 | 2-((1-allyl-2,5-dioxopyrrolidin-3-yl)thio)benzoic acid |
| F1591-0812 | 4-(2,5-dioxopyrrolidin-1-yl)-N-(4-(morpholinosulfonyl)phenyl)benzamide |
| F2368-3114 | 4-(2,5-dioxopyrrolidin-1-yl)-N-(5-(4-(ethylthio)benzyl)-1,3,4-oxadiazol-2-yl)benzamide |
| F1374-0054 | N-(5-(2-chlorophenyl)-1,3,4-oxadiazol-2-yl)-3-(2,5-dioxopyrrolidin-1-yl)benzamide |
| F5833-3365 | 2-(2,6-dioxopiperidin-1-yl)-N-(1-(methylsulfonyl)-1,2,3,4-tetrahydroquinolin-7-yl)ethane-1-sulfonamide |
| F2019-1334 | N-(3-(dimethylamino)propyl)-3-(2,5-dioxopyrrolidin-1-yl)-N-(4-fluorobenzo[d]thiazol-2-yl)benzamide hydrochloride |
| F3023-0836 | 3-((4-ethoxyphenyl)amino)-1-(o-tolyl)pyrrolidine-2,5-dione |
| F2100-0034 | (Z)-methyl 2-((1R,4S,7R)-7-(bromomethyl)-4,7-dimethyl-3-oxobicyclo[2.2.1]heptan-2-ylidene)-2-hydroxyacetate |
| F3406-3516 | 4-(4-methyl-2-(1H-pyrrol-1-yl)thiazole-5-carbonyl)-1-phenethylpiperazine-2,6-dione |
| F3325-0002 | 3-((4-fluorophenyl)amino)-1-(4-hydroxyphenyl)pyrrolidine-2,5-dione |
| F3171-0076 | 1,4-bis(4-chloro-3,5-dimethyl-1H-pyrazol-1-yl)butane-1,4-dione |
| F3228-0103 | (E)-1-(3,5-dichloro-2-hydroxyphenyl)-3-(pyridin-2-yl)prop-2-en-1-one |
| F3111-2313 | 3-iodo-1-(p-tolyl)pyrrolidine-2,5-dione |
| F3205-0002 | 3-(benzo[d][1,3]dioxol-5-ylamino)-1-benzylpyrrolidine-2,5-dione |
| F3023-0390 | 3-((4-ethylphenyl)amino)-1-(4-methoxyphenyl)pyrrolidine-2,5-dione |
| F1175-0186 | 2-chloro-N-(3-hydroxyphenyl)-4-nitrobenzamide |
| F3145-4553 | (E)-2,4-dichloro-6-((((tetrahydrofuran-2-yl)methyl)imino)methyl)phenol |
| F1751-0035 | (E)-1-(4-ethoxyphenyl)-3-((1,3,3-trimethylindolin-2-ylidene)methyl)pyrrolidine-2,5-dione |
| F0921-4246 | (Z)-5-(3-chloro-4-hydroxybenzylidene)-3-ethyl-2-thioxoimidazolidin-4-one |
| F1593-0042 | 1-(3,4-dimethoxyphenethyl)-3-((4-morpholinophenyl)amino)pyrrolidine-2,5-dione |
| F5581-0025 | N-(2-(1H-pyrazol-1-yl)ethyl)-N-(benzo[d]thiazol-2-yl)-3-(2,5-dioxopyrrolidin-1-yl)benzamide |
| F3023-0438 | 3-(((3s,5s,7s)-adamantan-1-yl)amino)-1-(benzo[d][1,3]dioxol-5-ylmethyl)pyrrolidine-2,5-dione |
| F0882-1056 | N-(2-(benzo[d]thiazol-2-yl)phenyl)-4-(2,5-dioxopyrrolidin-1-yl)benzamide |
| F0239-0139 | 4-chloro-N-(3-cyanothiophen-2-yl)-3-nitrobenzamide |
| F1751-0009 | 1-(4-methoxyphenyl)-3-(2-phenylhydrazinyl)pyrrolidine-2,5-dione |
| F0779-0616 | N-(4-(benzo[d]thiazol-2-yl)thiazol-2-yl)-2-(2,5-dioxopyrrolidin-1-yl)acetamide |
| F3264-0150 | 2-(7'-(2-hydroxyphenyl)-2,2',5-trioxo-3',7'-dihydro-2'H,5'H-spiro[pyrrolidine-3,6'-thiopyrano[2,3-d]thiazol]-1-yl)acetic acid |
| F1065-0144 | 2-((1-(2,3-dimethylphenyl)-2,5-dioxopyrrolidin-3-yl)thio)benzoic acid |
| F0270-0021 | 2-((2,5-dioxo-1-phenylpyrrolidin-3-yl)thio)benzoic acid |
| F1277-0114 | 2-((2,5-dioxo-1-(p-tolyl)pyrrolidin-3-yl)thio)-N-phenylacetamide |
| F0806-0876 | N-(2-((2-cyanoethyl)thio)phenyl)-4-(2,5-dioxopyrrolidin-1-yl)benzamide |
| F1065-0143 | 2-((1-benzyl-2,5-dioxopyrrolidin-3-yl)thio)benzoic acid |
| F3048-0084 | methyl 4-(3-((2-((3,4-dimethylphenyl)amino)-2-oxoethyl)thio)-2,5-dioxopyrrolidin-1-yl)benzoate |
| F0779-0604 | N-(4-(benzo[d]thiazol-2-yl)thiazol-2-yl)-4-(2,5-dioxopyrrolidin-1-yl)benzamide |
| F0286-0291 | 4-chloro-N-(1,5-dimethyl-3-oxo-2-phenyl-2,3-dihydro-1H-pyrazol-4-yl)-3-nitrobenzamide |
| F0020-1956 | 2-chloro-N-(9,10-dioxo-9,10-dihydroanthracen-2-yl)acetamide |
| F3111-2122 | N-(3-chlorophenyl)-N-(1-(3-chlorophenyl)-2,5-dioxopyrrolidin-3-yl)isonicotinamide |
| F3111-3610 | 3-(2-(4-bromophenoxy)acetyl)-4-hydroxy-2H-chromen-2-one |
| F0327-0349 | N-(5-benzoyl-4-phenylthiazol-2-yl)-4-(2,5-dioxopyrrolidin-1-yl)benzamide |
| F2224-0262 | 4-chloro-N-(2-fluoro-5-(2-methyl-4-oxopyrido[2,3-d]pyrimidin-3(4H)-yl)phenyl)-3-nitrobenzamide |
| F1649-0519 | ethyl 4-(3-((5-(2-hydroxyphenyl)-1,3,4-oxadiazol-2-yl)thio)-2,5-dioxopyrrolidin-1-yl)benzoate |
| F0549-0283 | 5-amino-1-tosyl-1H-pyrazol-3-yl 4-chloro-3-nitrobenzoate |
| F0588-0121 | ethyl 3-(4-chloro-3-nitrobenzamido)-1H-indole-2-carboxylate |
